# Enantio‐ and Regioselective Palladium(II)‐Catalyzed Dioxygenation of (Aza‐)Alkenols

**DOI:** 10.1002/anie.202109312

**Published:** 2021-09-06

**Authors:** Sabrina Giofrè, Letizia Molteni, Donatella Nava, Leonardo Lo Presti, Egle Maria Beccalli

**Affiliations:** ^1^ DISFARM, Sezione di Chimica Generale e Organica “A. Marchesini” Università degli Studi di Milano Via Venezian 21 20133 Milano Italy; ^2^ Dipartimento di Chimica, Università degli Studi di Milano Via Golgi 19 20133 Milano Italy

**Keywords:** alkene difunctionalizations, alkenols, dioxygenation, enantioselectivity, hypervalent iodine, Pd catalysis

## Abstract

An oxidative Pd‐catalyzed intra‐intermolecular dioxygenation of (aza‐)alkenols has been reported, with total regioselectivity. To study the stereoselectivity, different chiral ligands as well as different hypervalent‐iodine compounds have been compared. In particular, by using a C‐6 modified pyridinyl‐oxazoline (Pyox) ligand and hypervalent iodine bearing an aromatic ring, an excellent enantio‐ and diastereoselectivity has been achieved.

## Graphical abstract

A palladium‐catalyzed intramolecular difunctionalization of (aza‐)alkenols with excellent regioselectivity is reported. A new generation of C‐6‐modified Pyox ligands together with the use of unusual hypervalent iodine reagents as oxidants enabled the generation of acyloxy‐functionalized heterocycles in excellent enantio‐ and diastereoselectivity.

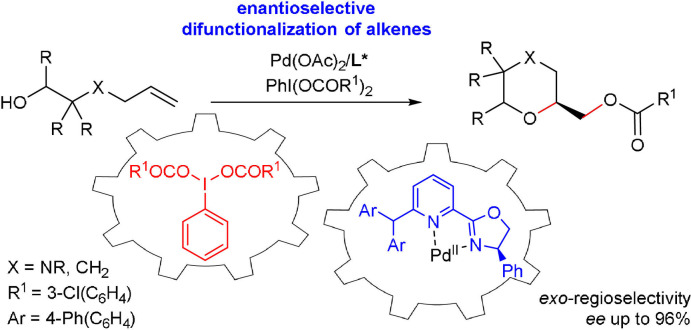

The formation of two carbon‐heteroatom bonds across an unsaturated system in *one‐pot* process have gained considerable importance as a means to achieve structural motifs in a rapid manner and to provide the main goals of the modern organic synthesis as efficiency, atom‐economy, and sustainability.^[1]^ This process do not require isolation of intermediates, change of the reaction conditions, or addition of further reagents to those initially mixed. To achieve this goal, different methods have been developed, making use of transition metal catalysts in oxidative conditions. In particular, the Pd‐catalyzed difunctionalization of alkenes^[2]^ has been widely studied due to high tolerability of different functional groups and mild reaction conditions required. In these reactions, the presence of an oxidant, such as copper salts, quinones and hypervalent iodines, is required in order to oxidize the Pd‐catalyst to its active species. Recently, difunctionalization of alkenes in transition metal free conditions have also been reported, making use of the electrophilic nature of hypervalent iodines, in intramolecular C‐N and C−O bond formations.^[3]^ Nevertheless, the limited substrate scope and the need in some cases of stoichiometric amount of the chiral iodine represent still a challenge of this emerging field. Moreover, despite several studies have been published on enantioselective intermolecular dialkoxylations,[^3c^, ^4^] less explored is the field of the asymmetric intramolecular C−O bond formation of alkenols.

In our interest in developing efficacious Pd‐catalyzed alkenes difunctionalizations,^[5]^ we recently reported the dialkoxylation and alkoxyarylation,^[6]^ processes, starting from 3‐aza‐5‐alkenols, using different oxidants species and nucleophiles. (Scheme 1 a). The two reaction typologies involved an intramolecular step with different regioselectivity, as a result of a 6‐*exo*‐ or 7‐*endo*‐*trig* cyclization step. Lately, difunctionalizations involving stereocontrol have gained considerable attention, too.

**Scheme 1:**
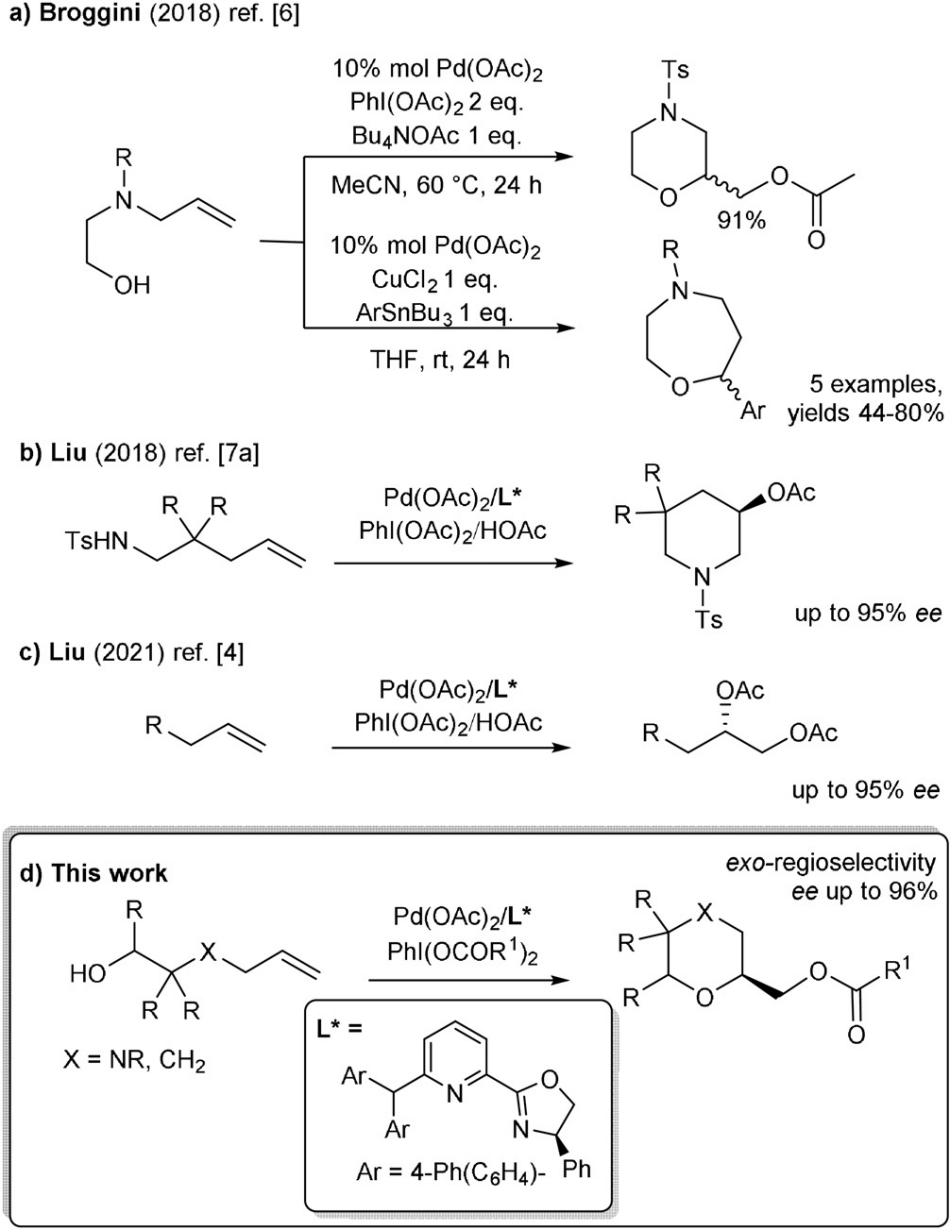
Recent difunctionalizations of unactivated alkenes.

The enantioselective Pd^II^‐catalyzed difunctionalizations of amino alkenes have been recently reported by Liu.^[7]^ In particular, an asymmetric 6‐*endo*‐aminoacetoxylation has been described as efficient method to afford enantioenriched 3‐acetoxy piperidines (Scheme 1 b).^[7a]^ Also an inter‐intermolecular dialkoxylation has been lately described on terminal alkenes as efficient way to access various chiral 1,2‐diols (Scheme 1 c).^[4]^

In the present work, we focused on the development of enantioselective cyclization of (aza‐)alkenols, for the synthesis of functionalized *O*‐containing heterocycles under mild reaction conditions and in particular, on the inter‐intramolecular alkoxyacyloxylation process (Scheme 1 d). To the best of our knowledge, only few examples of enantioselective cyclization involving the oxygen atom in Pd^II^‐catalyzed difunctionalizations has been reported so far.^[8]^ Indeed, enantioselective hydroalkoxylation have been mainly described as way to access chiral *O*‐containing heterocycles (through Cu^II^‐catalysis,^[9]^ Ti^IV^‐catalysis^[10]^ Co^II^‐catalysis,^[11]^ Ni^II^‐catalysis^[12]^ or Brønsted acid‐catalysis^[13]^).

Thus, in order to test the efficiency of different ligands in intramolecular C−O bond formation, we synthetized the already described ligands (**L1**, **L3**)^[7a]^ and the new ones (**L2**, **L4**), where specifically the steric hindrance given by the biphenyl disubstitution on C‐6 position of the **L4** may result in an increase Pd‐complex reactivity.

Using the *N*‐allyl,*N*‐Ts‐aminoethanol **1 a** as starting material, the difunctionalization process was performed in the presence of Pd(OAc)_2_ as catalyst and a modified hypervalent iodine,^[14]^ PhI(mcba)_2_ **2 a**, able to act both as oxidant and nucleophilic source, in DCM as solvent (entry 1, Table 1). The process was completely regioselective, affording the 2‐substituted *N*‐Ts‐morpholine **3 aa,** in 61 % yield, as result of 6‐*exo*‐cyclization. The reaction carried out in the presence of **L1** at room temperature in trifluoromethylbenzene afforded the product **3 aa** in good yields (78 %), but really low enantioselectivity (entry 2, Table 1). A significant improvement of the enantioselectivity was observed, decreasing the temperature from 10 °C to 0 °C and to −20 °C, with an enantiomeric ratio from 77:23 to 81:19 (entries 4–6, Table 1). The employment of the different ligands, **L1**‐**L4,** influenced slightly the enantioselectivity but it significantly increased the yields of **3 aa**, from 62 % to 77 %, changing from the less sterically‐hindered ligands **L2** to the most congested **L4** (entries 5,7–9, Table 1). Changing the solvent, we observed that, while the mixture of dichloromethane with polar solvents, such as acetonitrile, had a detrimental effect on both enantioselectivity and reactivity (entries 11,14), the mixture with the xylene (entry 12) was advantageous on both the aspects. Using the classic commercially available Pyox ligand (^H^PyOx^tBu^), no reaction was observed.

**Table 1:** Optimization of the Pd‐catalyzed enantioselective dialkoxylation 
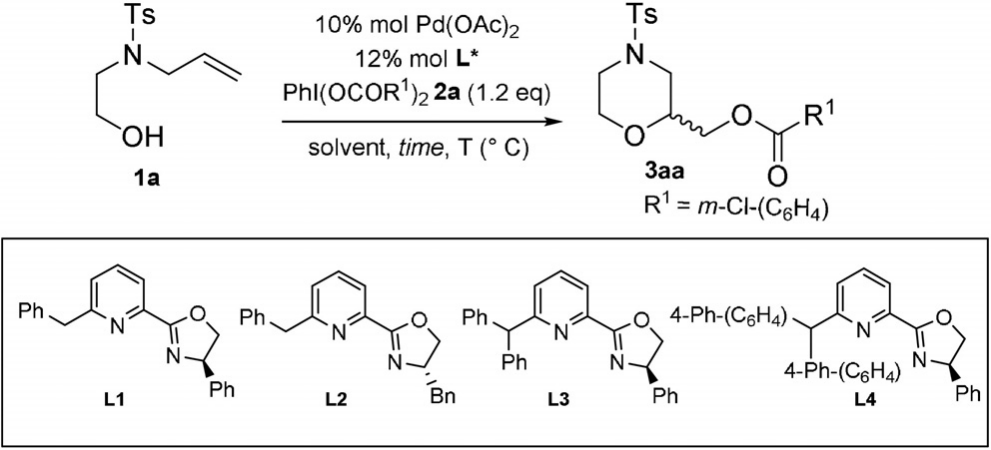

Entry	Lig.	Solvent (0.3 M)	Time (h)	T (°C)	**3 aa** %Yield	e.r. (*S:R*)
1	**‐**	DCM	24 h	rt	61 %	50:50
2	**L1**	PhCF_3_	24 h	rt	78 %	54:46
3	**L1**	PhCF_3_	30 h	10 °C	75 %	70:30
4	**L1**	DCM	30 h	10 °C	69 %	77:23
5	**L1**	DCM	48 h	0 °C	71 %	80:20
6	**L1**	DCM	48 h	‐20 °C	49 %	81:19
7	**L2**	DCM	48 h	0 °C	62 %	17:83
8	**L3**	DCM	48 h	0 °C	73 %	85:15
9	**L4**	DCM	48 h	0 °C	77 %	86:14
10	**L4**	ClPh	48 h	0 °C	82 %	71:29
11	**L4**	CH_3_CN/DCM 1:1	48 h	0 °C	55 %	74:26
**12**	**L4**	**DCM/xylene 1:2**	**48 h**	**0 °C**	**79 %**	**88:12**
13	**L5**	DCM/xylene 1:2	48 h	0 °C	‐	‐
14	**L4**	CH_3_CN/toluene 1:3	48 h	0 °C	64 %	77:23

**2 a**=PhI(mcba)_2_; **L5**=^**H**^ **PyOx^tBu^**

In order to further investigate and confirm the advantages of this protocol (Table 1, entry 12), a comparison with the classic acetoxylation conditions,^[7a]^ involving the commercially available PhI(OAc)_2_, was carried out, together with other hypervalent iodines (Scheme 2 a‐b). By using PhI(OAc)_2,_ both enantioselectivity and yields were compromised, the product **3 ab** was obtained in *er* 67:33 and 57 % yields. Switching to the bi‐*o*‐fluoro‐benzoic derivative, PhI(ofba)_2_ **2 c**, with similar feature to PhI(mcba)_2_ **2 a**, the corresponding morpholine derivative **2 ac** was obtained in satisfying yield, with complete consumption of the starting material after 24 h, even if with lower *er*. In the case of the bi‐*N*‐acetyl‐alaninate, PhI(*N*‐acetyl‐Ala)_2_ **2 d**, the results were unsatisfactory, with almost no reactivity at 0 °C and no diastereoselectivity at room temperature. We tried to explain the influence of the different hypervalent iodines on the outcome of the reaction through ^1^H NMR studies (Scheme 2 c). Thus, by mixing the ligand **L4**, Pd(OAc)_2_, and PhI(OAc)_2_ **2 b** or PhI(mcba)_2_ **2 a** in a 1:1:2 stoichiometric ratios, we analyzed the corresponding ^1^H NMR at time 0 (5 min) and after 2 h. Interestingly, while in the NMR spectra regarding PhI(OAc)_2_ the signals relative to **L4** (circle) were never disappearing, even after 24 h, in the NMR related to PhI(mcba)_2_, the **L4** signals completely disappeared after 2 h and a new stable complex was formed, characterized by a shift of the proton 6‐PyH(Ar)_2_ from 6.03 to 6.82 ppm, which was even stable after 24 h at 0 °C. As a consequence, the not complete complexation of the palladium in the first case could explain the low enantioselectivity observed with PhI(OAc)_2,_ while it is possible to hypothesize that the new species formed with PhI(mcba)_2_ **2 a** might be stabilized by π‐π interactions between the benzoate of the hypervalent iodine and the aromatic ring of the ligand, improving its performance in the asymmetric difunctionalization.^[15]^

**Scheme 2:**
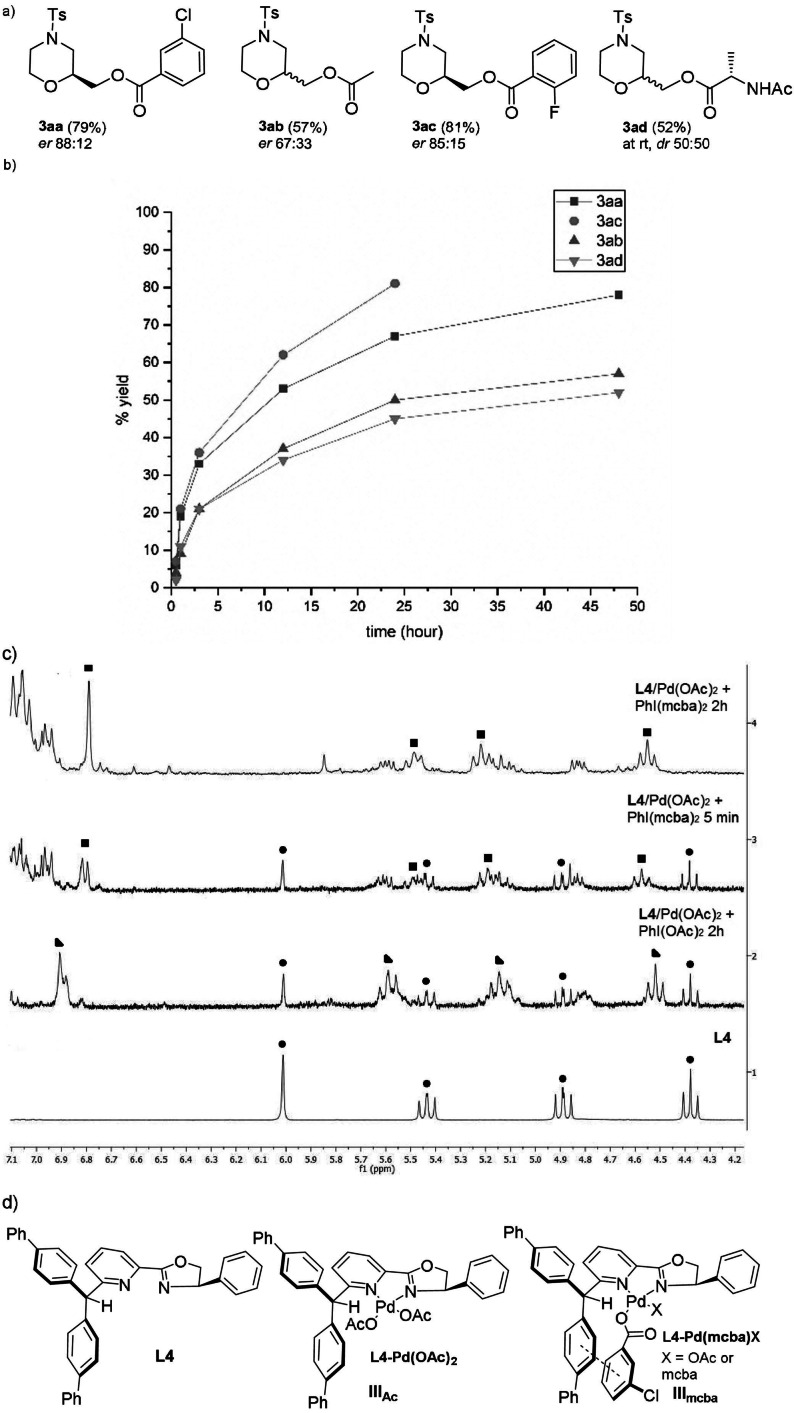
Investigations on the effect of different hypervlant iodines **2 a**–**d** on the different reactivity and enatioselectivity observed: a) products **3 aa‐3 ad**; b) hypervalent iodines effect on reaction rate; c) ^1^H NMR spectra regarding the complexation of **L4** given by Pd(OAc)_2_ in the presence of PhI(mcba)_2_ or PhI(OAc)_2_; d) proposed complexes **III_Ac_** and **III_mcba_**.

Taking the reaction conditions reported in entry 12, Table 1, as the optimized ones, different class of (aza‐)alkenyl alcohols were investigated in order to check the applicability of the method (Scheme 3). Replacing the Ts‐protecting group on the aminoethanol with the Boc‐group (**1 b**) resulted in a slightly loss of both reactivity and enantioselectivity, while the Ns‐derivative **3 da** ^[16]^ was obtained in higher yields and comparable *er*. The substrate **1 c**, bearing the di‐substitution on the α‐position to the amino group, gave the best result, in accordance with the recent contributions,^[7]^ where the Thorpe–Ingold effect given by the di‐substitution favored both cyclization and enantioselectivity. Then, starting from simple alkenols **1 e**–**i**, we observed a ligand‐induced enhanced reactivity. Indeed, the reaction performed in the absence of the ligand furnished the alkoxyacyloxylated product in really low yields and in a mixture with other undesired products. Thus, with the protocol in our hand (entry 12, Table 1), we were able to achieve the functionalized pyrans **3 ea‐na** in moderate yields and satisfying enantioselectivity, as exclusively *exo*‐products (confirmed by ^1^H‐NOESY NMR, n.O.e between the two protons, CH and CH_2_, bonded to the oxygen of the 6‐membered ring). Due to the geometry of the system, the 2,5‐*trans* diastereoselectivity was preferred in the case of compounds **3 ja‐3 la** (*trans/cis* >2.5:1). On the other hand, when the substituent was located on the carbon bonded to the oxygen, only the 2,6‐*cis* diastereoisomers were isolated, as demonstrated by compound **3 ma‐na** (*trans/cis* >1:10). When the reaction was applied on chiral *N*‐allyl aminoethanols **1 o**–**s**, the chiral center located on the substrates was the main driver of the stereoselectivity (Scheme 3 b). For this reason, the alkoxyacyloxylation was carried out in the presence of the racemic ligand, which led to the formation of the *cis*‐products as single diastereoisomers.^[17]^ The structures of the 2,5‐*cis* disubstitued morpholines **3 oa‐sa** have been confirmed by ^1^H‐NOESY NMR.

**Scheme 3:**
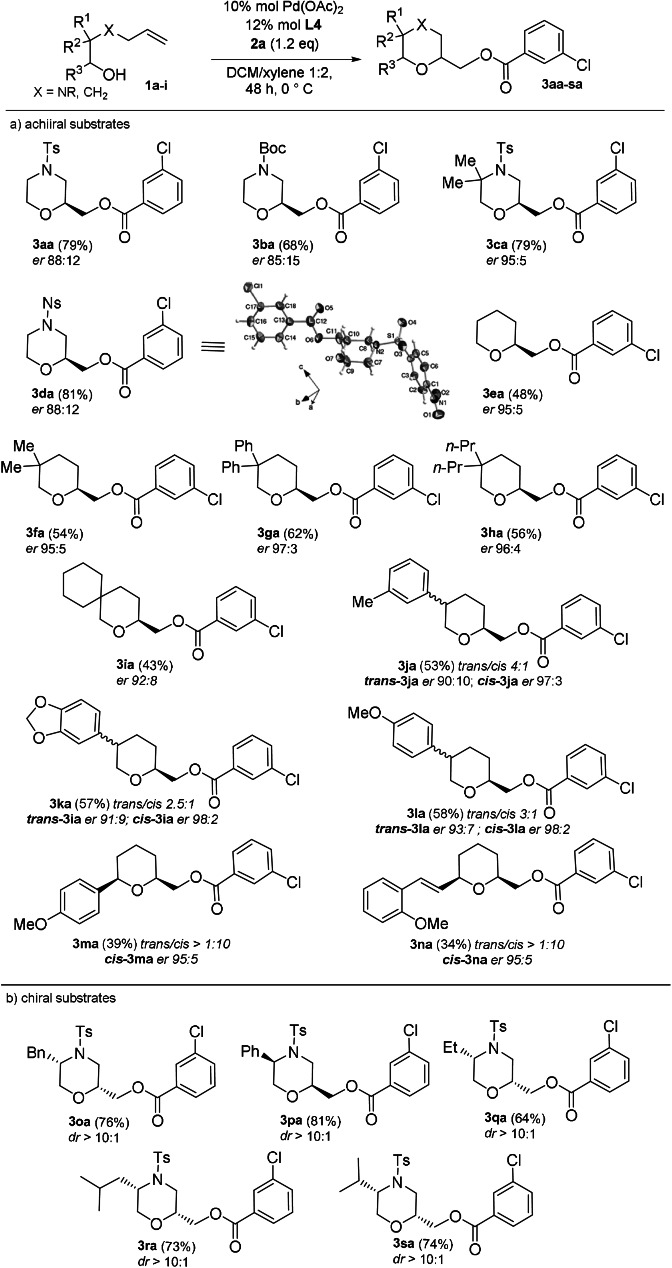
Scope of the enantioselective Pd‐catalyzed alkoxyacyloxylation on (aza‐)alkenols.

To justify the results observed, a possible mechanism of the process was proposed (Scheme 4). After the formation of Pd/L* complex, the activation of the double bond mediated by the palladium leads to the oxygen nucleophile addition with formation of the corresponding alkoxypalladated species **II**.^[18]^ The interaction of the oxidant agent with the ligand and simultaneous complexation with metal directly affects stereoselectivity observed (as elucidated above through NMR studies). The subsequent reductive elimination affords the alkoxyacyloxylated compound as exclusive *exo*‐product.

**Scheme 4:**
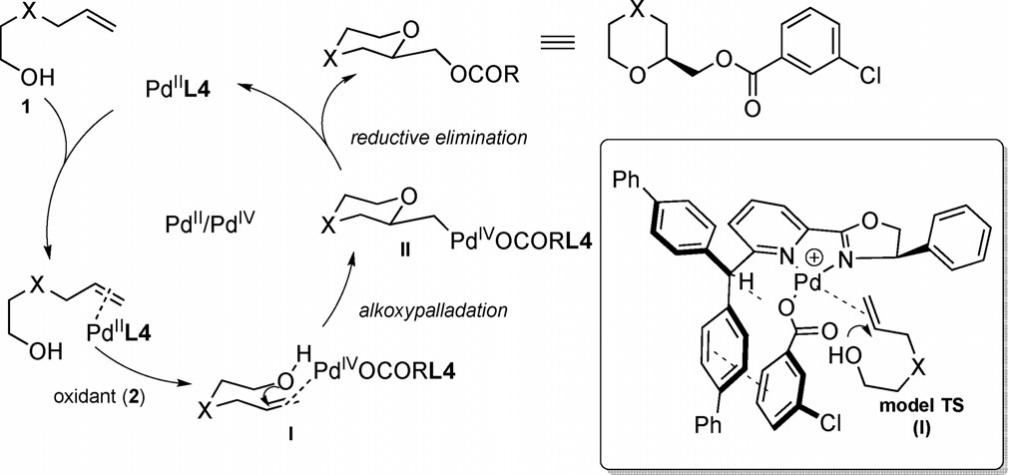
Proposed mechanism for the alkoxyacyloxylation process starting from substrates **1**.

In addition, the ester functionality inserted in the morpholines, offers the possibility of further transformations, including hydrolysis and oxidation of the alcohol to afford enantioenriched or diastereopure β‐aminoacids,^[19]^ as well as important intermediates for natural products synthesis, such as (+)‐centrolobine (Scheme 5).^[20]^

**Scheme 5:**
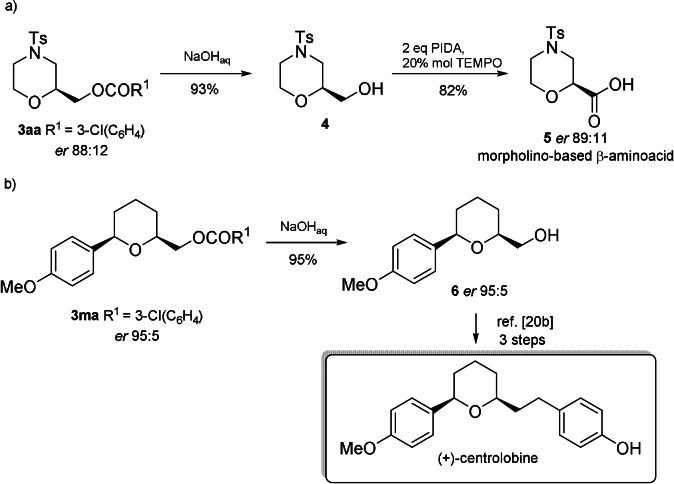
Synthetic applications of the chiral morpholine and pyran nuclei obtained.

In conclusion, we described a useful and mild procedure for the synthesis of enantioenriched acyloxy‐substituted morpholines and pyrans using (aza‐)alkenols as starting materials. The reaction makes use of a Pd‐catalyst in oxidative conditions, in combination with the new generation of C‐6 modified Pyox ligands. The use of uncommon hypervalent iodines was also crucial to afford an enhanced enantioselectivity and reactivity. The process was completely *exo*‐regioselective, starting from both achiral and chiral substrates. The achievement of intermediates important for natural products synthesis and peptide synthesis increases the synthetic utility of this method.

## Conflict of interest

The authors declare no conflict of interest.

## Supporting information

As a service to our authors and readers, this journal provides supporting information supplied by the authors. Such materials are peer reviewed and may be re‐organized for online delivery, but are not copy‐edited or typeset. Technical support issues arising from supporting information (other than missing files) should be addressed to the authors.

Supporting Information
