# Supplementary material for: Enantio‐ and Regioselective Palladium(II)‐Catalyzed Dioxygenation of (Aza‐)Alkenols
Source: Angew Chem Int Ed Engl. 2021 Sep 6;60(40):21723–7. doi: 10.1002/anie.202109312 (PMC8518864; doi:10.1002/anie.202109312)
Supplement: Supplementary file 1 — Supporting Information [file ANIE-60-21723-s001.pdf]

Angewandte Chemie
Eine Zeitschrift der Gesellschaft Deutscher Chemiker

Supporting Information

# Enantio- and Regioselective Palladium(II)-Catalyzed Dioxygenation of (Aza-)Alkenols

*Sabrina Giofrè, Letizia Molteni, Donatella Nava, Leonardo Lo Presti, and Egle Maria Beccalli**

anie_202109312_sm_miscellaneous_information.pdf

## Contents

General Information ....................2
General Procedures ....................3
General procedure for the preparation of C-6 modified Pyox ligand (GP1) ....................3
General procedure for the preparation of differently substituted hypervalent iodine (III) (GP2)....................4
General procedure for the synthesis of *N*-allyl Ts-protected aminoethanols 1 (GP3) ....................4
General procedure for the synthesis of alkenols (GP4) ....................4
General procedure for the synthesis of alkenols (GP5) ....................5
General procedure for the racemic intramolecular dialkoxylation (GP6) ....................5
General procedure for the asymmetric intramolecular dialkoxylation (GP7) ....................6
Optimization of the Enantioselective Dialkoxylation starting from the *N*-Allyl-*N*-Ts-aminoethanol 1a ....................7
NMR Studies on the Effect of Hypervalent Iodines on the Pd-ligand Complex ....................9
Interpretation of the hypothesized alkoxypalladation step through $^{1}$H NOESY NMR .................... 15
$^{1}$H NOESY NMR couplings of the corresponding diastereoisomer formed starting from alkenols (1j) and chiral aminoethanols .................... 16
Behaviour of chiral aminoethanols in the presence of ligands of opposite configuration. .................... 19
Synthetic routes to access to (+/-)-centrolobine .................... 20
Further Procedures and Analytical Data of Unknown Compounds .................... 21
X-Ray Cristallography (Prof. Leonardo Lo Presti) .................... 46
Single crystal X-ray diffraction analysis of the compound 3da .................... 46
$^{1}$H NMR and $^{13}$C NMR spectra and HPLC Data .................... 50

# General Information

Melting points were determined by the capillary method with a Büchi B-540 apparatus and are uncorrected.

Chemicals were purchased from Sigma Aldrich and FluoroChem and used without any further purification.

IR spectra were measured with a Jasco FT/IR 5300 spectrometer, using ATR Sampling.

Nuclear Magnetic Resonance Spectroscopy (NMR)

$^{1}$H NMR and $^{13}$C in open capillary tubes. NMR spectra were recorded with: AVANCE 400 Bruker spectrometer at 400 and 100 MHz, Varian Oxford 300 MHz spectrometer at 300 and 75 MHz and AVANCE 500 Bruker spectrometer at 500 and 125 MHz, respectively. Chemical shifts are given as δ values in ppm relative to residual solvent peaks ($CHCl_3$) as the internal reference, and the coupling constants *J* are reported in Hertz (Hz).

$^{13}$C NMR spectra are $^{1}$H-decoupled and the determination of the multiplicities was achieved by the APT pulse sequence or by HSQC 2D NMR. Further investigations have been made by COSY and NOESY NMR.

High Pressure Liquid Chromatography (HPLC)

The chiral HPLC analysis were carried out by using Merck LaChrom Hitachi Pump L-7100, Hewlett Packard Series 1050 DAD, and the following chiral column: Phenomenex Lux 3µ Amylose-2 (250 x 4.60 mm, 3 µm), Phenomenex Lux 3µ Amylose-2 (50 x 4.60 mm, 3 µm), OD-H (250 x 4.60 mm, 5 µm) and AkzoNobel Kromasil 5-AmyCoat column (4.6 mm i.d. × 250 mm, 5 µm).

Optical rotations were measured on a Perkin–Elmer 343 polarimeter at 20° C (concentration in g/mL).

Mass spectrometry

ESI mass spectra were recorded on a LCQ Advantage spectrometer from Thermo Finningan and values are reported in m/z.

# General Procedures

### General procedure for the preparation of C-6 modified Pyox ligand (GP1)

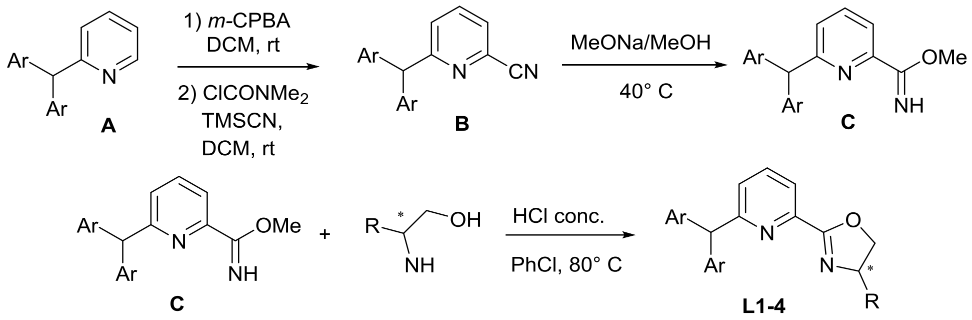


Following the procedure,[1] in a 250 mL round bottom flask, pyridine (1 eq) was dissolved in DCM (0.3 M). Then *m*-CPBA (meta-chloroperoxybenzoic acid, 1.7 eq) was added, and the mixture was stirred at room temperature. After the reaction was completed, $K_2CO_3$ (5 eq) was added. The mixture was stirred for 30 min at room temperature. After filtered through a celite pad, the filtrate was concentrated *in vacuo* to give the desired *N*-oxide, which was used without further purification. To a solution of the crude *N*-oxide in DCM (0.7 M), dimethylcarbamic chloride (1.3 eq) was added at room temperature. The mixture was stirred for 10 min, then TMSCN (1.3 eq) was added. The mixture was stirred at room temperature. After the reaction was completed, the mixture was quenched with 10% aq $K_2CO_3$, and the mixture was extracted two times with DCM. The combined organic layers were washed with brine, dried over $NaSO_4$, and filtered. After concentration, the crude residue was purified by column chromatography on silica gel or crystallization to afford product **B**.

In a 50mL round bottom flask, **B** (1 eq) was dissolved in MeOH (1.5 M). Then NaOMe (0.3 eq) was added. The reaction mixture was stirred at 40° C overnight. After that, the solvent was removed *in vacuo*, and the residue was dissolved in EtOAc. The solution was washed with water and brine, then the organic phase was separated and dried over $NaSO_4$. After filtration, the filtrate was concentrated *in vacuo* to give the imine **C**, which was used without any further purification. The crude imine (1 eq) and (*R*)-2-amino-2-phenyl ethan-1-ol (1 eq) or (*S)*-phenylalaninol were weighted into a 100 mL round bottom flask, then dissolved in PhCl (0.5 M). Concentrated HCl (2 drops) was added to the solution and the mixture was heated to 80° C under nitrogen atmosphere. After the reaction was completed, the organic solvent was removed *in vacuo*. The residue was purified by column chromatography on silica gel or crystallography to afford ligand **L1-L4**.

---

[1] C. Chen, P. M. Pflgger, P. Chen, G. Liu*, Angew. Chem. Int. Ed.* **2019**, 58, 2392–2396

## General procedure for the preparation of differently substituted hypervalent iodine (III) (GP2)

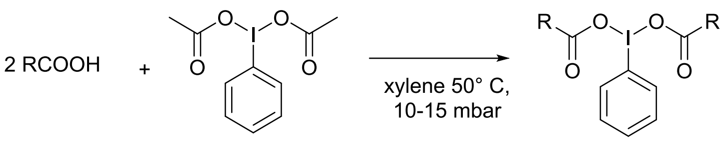


Following the procedure,[2] to a round-bottom flask, $PhI(OAc)_2$ (10 mmol, 1.0 eq.) and the corresponding acid (20 mmol, 2 eq) were dissolved in xylene (0.2 M) and the flask was heated to 50° C under reduced pressure (about 10.15 mbar) using a diaphragm pump. When the xylene was removed, a mixture of n-hexane/AcOEt 3:1 was used to wash the solid. The white solid was then filtered and dried *in vacuo*. The corresponding hypervalent iodine (III) **2a-d** was obtained and used directly in the following reaction without any further purification.

## General procedure for the synthesis of *N*-allyl Ts-protected aminoethanols 1 (GP3)

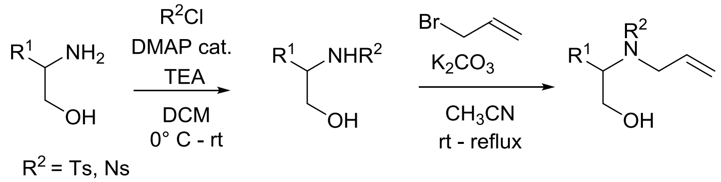


To a solution of aminoalcohol (10 mmol, 1 eq), DMAP (2 mmol, 0.1 eq) and TEA (20 mmol, 2 eq) in DCM (2 M) at 0 °C, a solution of the corresponding sulfonyl chloride was added dropwise (10.5 mmol, 1.05 eq) in DCM (0.8 M) and the resulting mixture was stirred at room temperature overnight. The mixture was washed with water (3 x 20 mL) and brine (1 x 20 mL), then dried over $Na_2SO_4$, and concentrated at reduced pressure. The crude product was purified by silica gel column chromatography to afford the corresponding protected aminoalcohol.

In a two-neck round-bottom flask the corresponding sulfonyl-protected aminophenol (1 eq) was dissolved in $CH_3CN$ (0.2 M) and then $K_2CO_3$ (1.2 eq) was added. A solution of allyl bromide (1.2 eq) in $CH_3CN$ (1 M) was added dropwise over 10 min. The reaction was refluxed overnight. Then the mixture was filtered to remove $K_2CO_3$, the solvent was evaporated *in vacuo*, the resulting crude product was rinsed with AcOEt and washed with brine. The organic phase was dried over $Na_2SO_4$ and the solvent evaporated under reduced pressure. The crude product was purified by flash chromatography on silica gel to afford compounds **1a,c,d,l-p.**

## General procedure for the synthesis of alkenols (GP4)

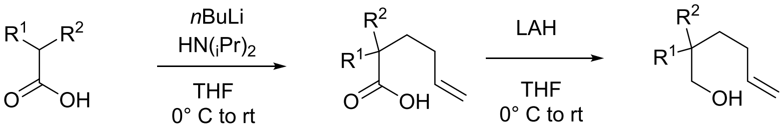


According to the reported procedure,[3] 2.5 M solution of n-BuLi in hexane (4.77 mL, 11.9 mmol) was added to a stirred solution of diisopropyl amine (1.75 mL, 12.45 mmol) in THF (10 mL) at 0°C under argon atmosphere. After 30 min, the corresponding acid (0.5 mL, 5.4 mmol) was added to the reaction mixture at 0°C. After 30 min, 4-bromobutene (0.65 mL, 6.4 mmol) was added to the mixture at 0°C and then warmed to rt. After 10 h, the mixture diluted with diethyl ether (8

[2] Y.Wang, L. Zhang, Y. Yang, P. Zhang, Z. Du, C. Wang, *J. Am. Chem. Soc.* **2013**, 135 (48), 18048-18051

[3] K. Kwon, J. S. Ham, H. Y. Kim, V. Sampath, H.-Y. Lee, *Asian J. Org. Chem.* **2017**, *6*, 1594.

mL) and saturated $NH_4Cl$ solution (10 mL) was added. The aqueous layer was extracted with diethyl ether (10 mL x 3). Combined organic layer was dried over $MgSO_4$, filtered and concentrated in vacuo. 1.0 M solution of LAH in THF (13mL, 13 mmol) was added to a solution of crude acid in dry THF (13 mL) at 0°C and then warmed to rt. After 1 h, water (0.5 mL), 20% NaOH (1 mL) and water (1.5 mL) were added to the mixture. The white solid was filtered over celite pad and the filtrate was concentrated in vacuo. The crude was purified by flash column chromatography on silica gel ($Et_2O$/n-Hex) to afford the corresponding alcohol.

## General procedure for the synthesis of alkenols (GP5)

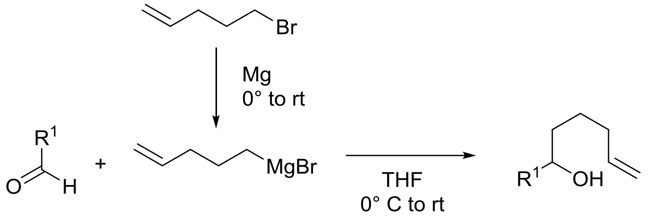


According to a revisited procedure of the one described in literature[4], to a dry, air free round-bottom flask were added sequentially magnesium turnings (146 mg, 6.07 mmol) and dry THF (7 mL). 5-Bromopent-1-ene (0.6 mL, 5.06 mmol) was added slowly to the stirring magnesium. After the mixture was stirred at ambient temperature for 1 h, dry THF (7 mL) was added, and the solution was cooled to 0 °C. The corresponding aldehyde (4.21 mmol) was added dropwise, and the mixture was allowed to warm to ambient temperature. After being stirred for 1-2 h, the reaction mixture was quenched with saturated aqueous $NH_4Cl$, and subsequently extracted with EtOAc (2 x 20 mL). The combined organic layers were washed with brine, dried over $Na_2SO_4$, and concentrated in vacuo to yield the corresponding alcohol which was purified by flash chromatography (purification only if necessary).

## General procedure for the racemic intramolecular dialkoxylation (GP6)

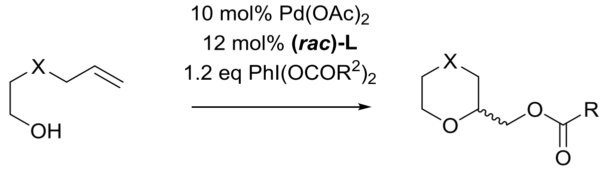


In a 5 mL glass vial $Pd(OAc)_2$ (0.025 mmol, 10 mol%) and the *rac*-ligand **L3** (0.03 mmol, 12 mol%) were added and dissolved in the corresponding solvent (0.3 M). The complex was preformed, stirring the mixture at rt for 15 min. Then, compound **1** (0.3 mmol, 1 eq) and $PhI(mcba)_2$ **2a** (0.45 mmol, 1.2 eq) were added subsequently and the reaction was cooled to 0° C. The reaction mixture was stirred at the reported temperature for 48 h.

Once the reaction had occurred as completely as possible, the solvent was evaporated *in vacuo*. The crude product was rinsed with EtOAc, washed with a saturated solution of $NaHCO_3$ (until pH = 8) and with brine. The organic phase was dried over $Na_2SO_4$, filtered and the solvent evaporated under reduced pressure. The crude product was purified by flash chromatography on silica gel.

[4] N. Yoneda, Y. Fujii, A. Matsumoto, K. Asano, S. Matsubara. *Nature Communications*., **2017**, 8 (1), 1–7

**General procedure for the asymmetric intramolecular dialkoxylation (GP7)**

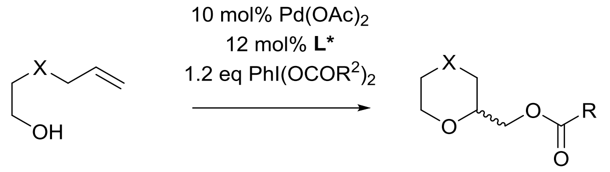


In a 5 mL glass vial $Pd(OAc)_2$ (0.025 mmol, 10 mol%) and the ligand **L1-L6** (0.03 mmol, 12 mol%) were added and dissolved in the corresponding solvent (0.3 M). The complex was preformed, stirring the mixture at rt for 15 min. Then, compound **1** (0.3 mmol, 1 eq) and $PhI(mcba)_2$ **2a** (0.45 mmol, 1.2 eq) were added subsequently and the reaction was cooled to 0° C. The reaction mixture was stirred at the reported temperature for 48 h, for substrates.

Once the reaction had occurred as completely as possible, the reaction mixture was immediately filtered through a plug of silica gel (3 cm) and washed with EtOAc/n-Hex 9:1. Then the collected fractions were evaporated *in vacuo*. The crude product was rinsed with EtOAc, washed with a saturated solution of $NaHCO_3$ (until pH = 8) and with brine. The organic phase was dried over $Na_2SO_4$, filtered and the solvent evaporated under reduced pressure. The crude product was purified by flash chromatography on silica gel.

# Optimization of the Enantioselective Dialkoxylation starting from the *N*-Allyl-*N*-Ts-aminoethanol 1a

The reactions were carried out according to the general procedure (GP7). After the reaction occurred as completely as possible, the reaction mixture was rinsed with EtOAc, filtered through a short silica pad and then the solvent evaporated. *N*-methyl aniline was used as internal standard for determining the yields through NMR. The enantiomeric ratio was obtained by analysing the crude mixture by chiral HPLC.

**Screening of Temperature**

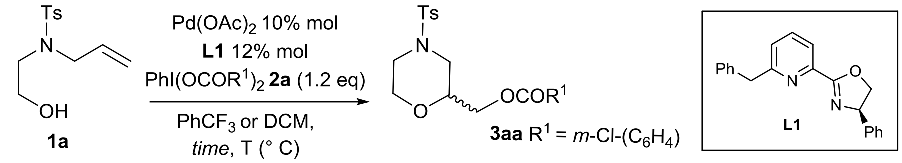


| Entry | Ligand | Solvent (0.3 M) | Time (h) | T°C | Yields % **3aa** | e.r. (*S*:*R*) |
|---|---|---|---|---|---|---|
| 1 | L1 | $PhCF_3$ | 24 h | rt | 78% | 54:46 |
| 2 | L1 | $PhCF_3$ | 30 h | 10 °C | 75% | 70:30 |
| 3 | L1 | DCM | 30 h | 10 °C | 69% | 77:23 |
| 4 | L1 | DCM | 48 h | -20 °C | 49% | 81:19 |
| 5 | L1 | DCM | 48 h | 0 °C | 71% | 80:20 |
| 6 | L1 | DCM | 48 h | -30 °C | 37% | 81:19 |

**Screening of Ligands**

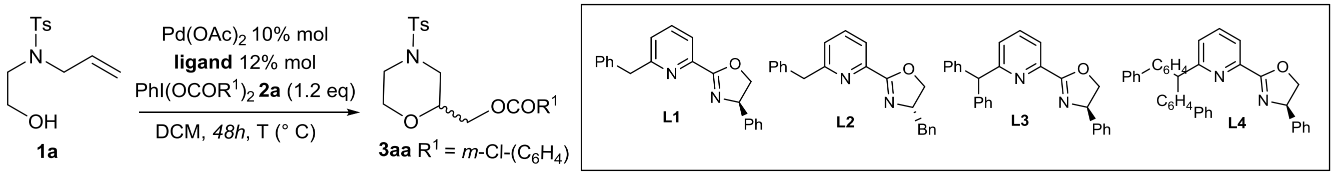


| Entry | Ligand | Time (h) | T°C | Yields % **3a** | e.r. (*S*:*R*) |
|---|---|---|---|---|---|
| 1 | - | 24 h | rt | 61% | 50:50 |
| 2 | L1 | 48 h | 0 °C | 71% | 80:20 |
| 3 | L2 | 48 h | 0 °C | 62% | 17:83 |
| 4 | L3 | 48 h | 0 °C | 73% | 85:15 |
| 5 | L4 | 48 h | 0 °C | 77% | 86:14 |

## Screening of Bases and Acid

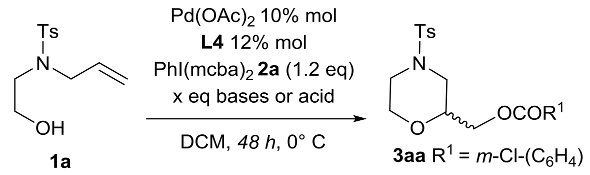


| Entry | Additives | Yields % **3aa** | e.r. (*S:R*) |
|---|---|---|---|
| 1 | - | 77% | 86:14 |
| 2 | 0.5 eq $Cs_2CO_3$ | 58% | 75:25 |
| 3 | 0.5 eq $K_2CO_3$ | 67% | 84:16 |
| 4 | 0.5 eq NaOAc | 74% | 85:15 |
| 5 | 1 eq NaOAc | 71% | 80:20 |
| 8 | 15% mol (*R*)-binaphtyl phosphoric acid | - | - |

## Screening of Solvents

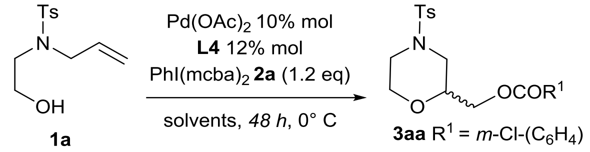


| Entry | Solvent | Yields % **3aa** | e.r. (*S:R*) |
|---|---|---|---|
| 1 | DCM | 77% | 86:14 |
| 2 | ClPh | 82% | 71:29 |
| 3 | $CH_3CN$/DCM 1:1 | 55% | 74:26 |
| 4 | DCM/xylene 1:2 | 79% | 88:12 |
| 5 | $CH_3CN$/toluene 1:3 | 64% | 77:23 |

**Screening of Hypervalent Iodines**

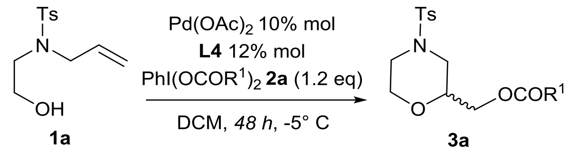


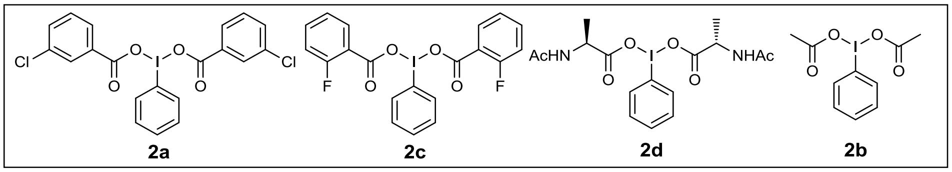


| Entry | Ligand | I(III) | T°C | Yields % **3aa** | e.r. (*S:R*) |
|---|---|---|---|---|---|
| 1 | **L4** | 2a | 0 °C | 79% | 88:12 |
| 2 | **L4** | **2c** | 0 °C | 81% | 85:15 |
| 3 | **L4** | **2d** | 0 °C | - | - |
| 4 | **L4** | **2d** | rt | 52% | 50:50 |
| 5 | L4 | **2b** | 0 °C | 57% | 67:33 |

# NMR Studies on the Effect of Hypervalent Iodines on the Pd-ligand Complex

Working in an open-air system, $Pd(OAc)_2$ (2 mg, 0.01 mmol), **L4** (5 mg, 0.01 mmol) and/or the different Hypervalent Iodines (2 equiv.) were placed into a vials. Then $CDCl_3$ was added, the vials were shacked and each mixture was directly transferred into an NMR tube. $^1$H NMR spectrum was acquired at different time and storing conditions for each hypervalent iodine.

(**Note**: the signals of the ligand were disappeared after 2 hours for both PhI(mcba)$_2$ and PhI(ofba)$_2$ while for PhI(OAc)$_2$ and PhI(*N*-Ac-Ala)$_2$ after 24 hours were still present. The replacement of the OAc, coordinated to Pd, with the aromatic mcba and ofba may stabilize the ligand-Pd complex increasing reactivity and enantioselectivity.

After 24 hours at room temperature the signals arising from $^1$H NMR spectrum of PhI(mcba)$_2$ got broader, probably due to the instability of the complex at rt, while storing the NMR tubes for 24 hours at 0° C didn't change significantly the spectrum, supporting the decrease of enantioselectivity at higher temperature.

The –CH bonded to the C6 position of the Pyridine shifts from 6.06 ppm to 6.78 ppm along with the complex formation.

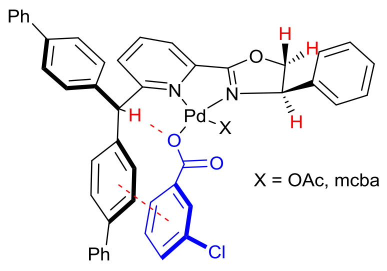
Ph
Ph
H
N
Pd
X
O
O
N
O
H
H
H
Cl
X = OAc, mcba

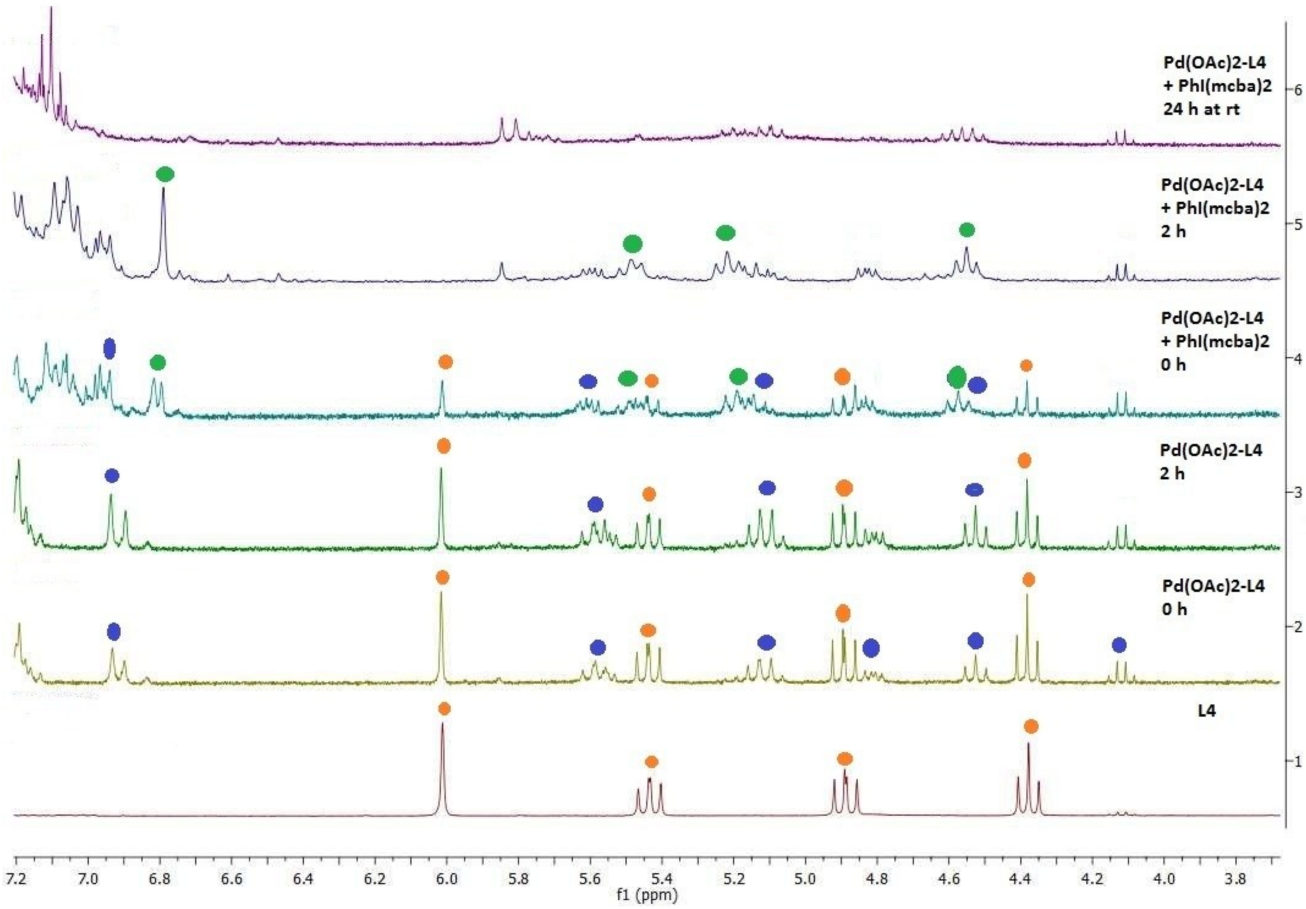
Pd(OAc)2-L4
+ PhI(mcba)2
24 h at rt
Pd(OAc)2-L4
+ PhI(mcba)2
2 h
Pd(OAc)2-L4
+ PhI(mcba)2
0 h
Pd(OAc)2-L4
2 h
Pd(OAc)2-L4
0 h
L4
f1 (ppm)

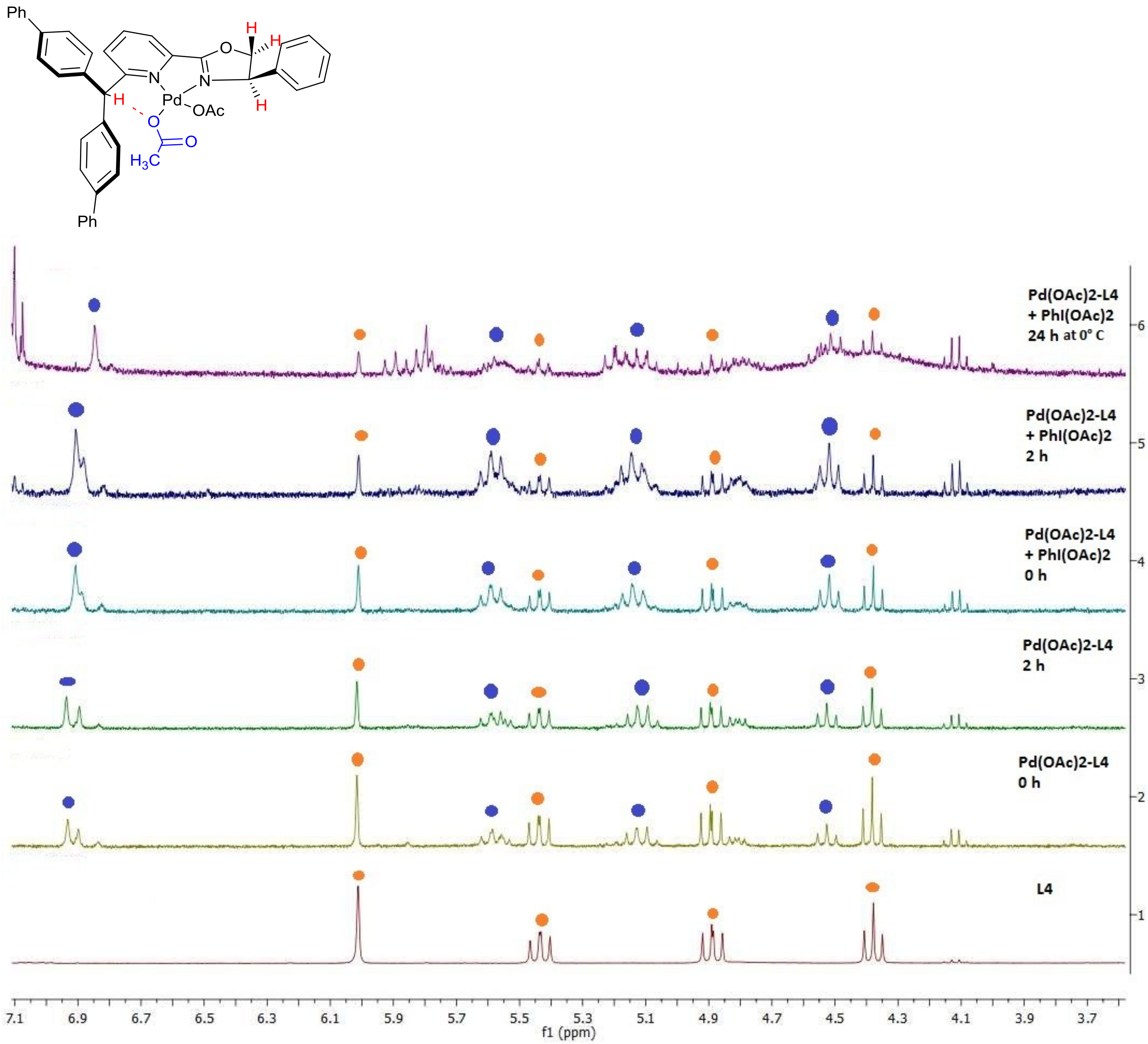
Ph
Pd
N
N
O
OAc
H
H
H
H
O
$H_3C$
O
Ph
Pd(OAc)2-L4
+ PhI(OAc)2
24 h at 0° C
Pd(OAc)2-L4
+ PhI(OAc)2
2 h
Pd(OAc)2-L4
+ PhI(OAc)2
0 h
Pd(OAc)2-L4
2 h
Pd(OAc)2-L4
0 h
L4
7.1 6.9 6.7 6.5 6.3 6.1 5.9 5.7 5.5 5.3 5.1 4.9 4.7 4.5 4.3 4.1 3.9 3.7
f1 (ppm)

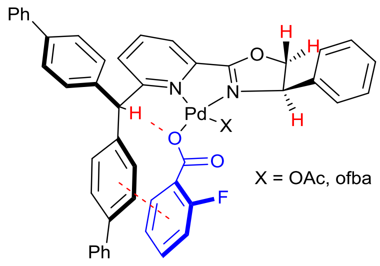
Ph
O
N
N
Pd
X
H
H
H
H
O
O
F
Ph
X = OAc, ofba

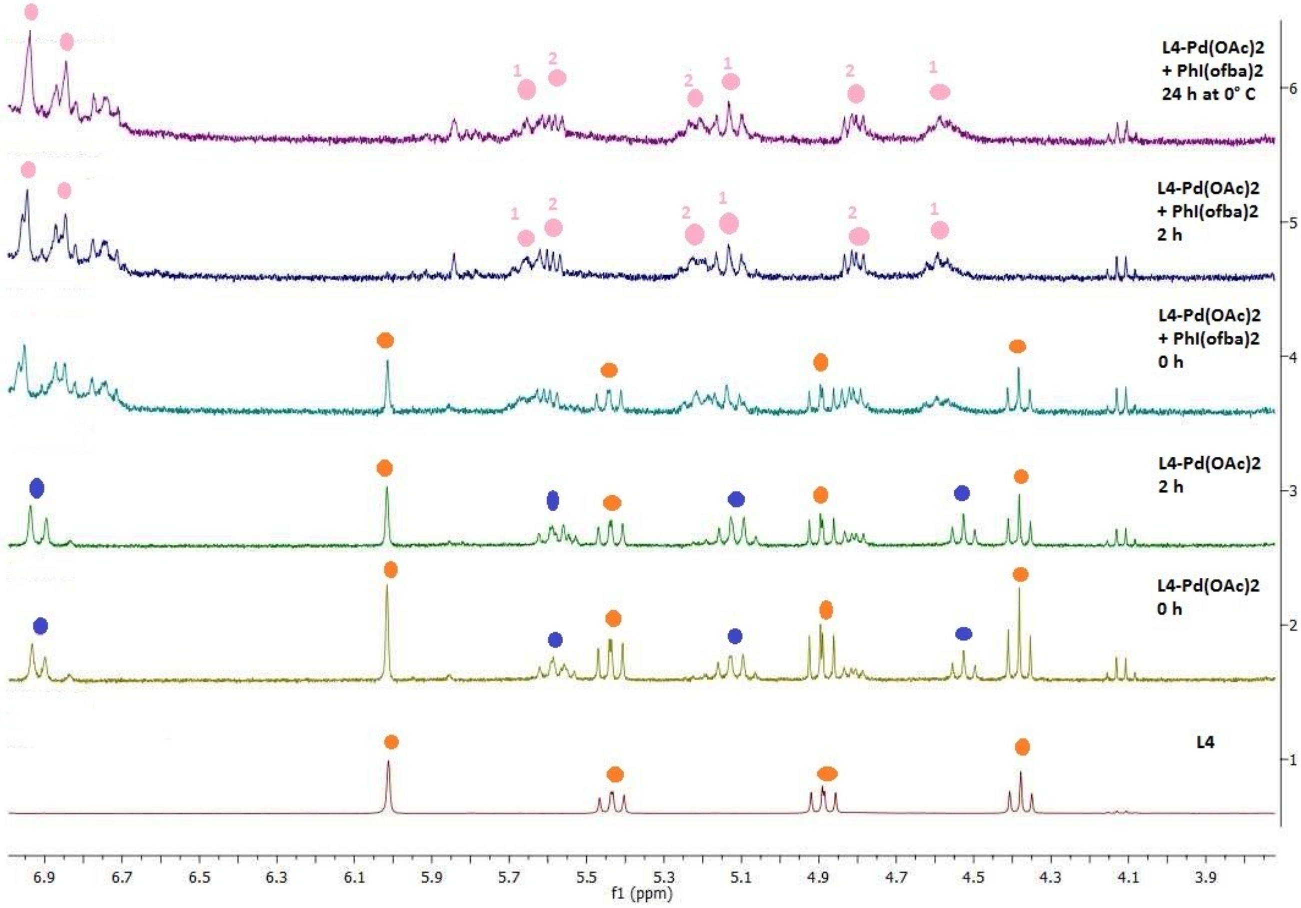
L4-Pd(OAc)2
+ PhI(ofba)2
24 h at 0° C
L4-Pd(OAc)2
+ PhI(ofba)2
2 h
L4-Pd(OAc)2
+ PhI(ofba)2
0 h
L4-Pd(OAc)2
2 h
L4-Pd(OAc)2
0 h
L4
f1 (ppm)

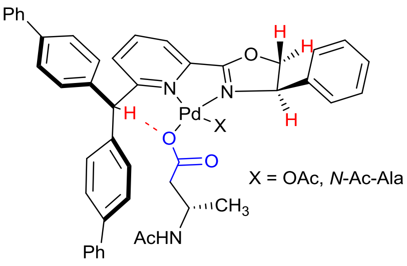
Ph
Ph
N
Pd
X
N
O
H
H
H
H
O
O
CH3
AcHN
X = OAc, N-Ac-Ala

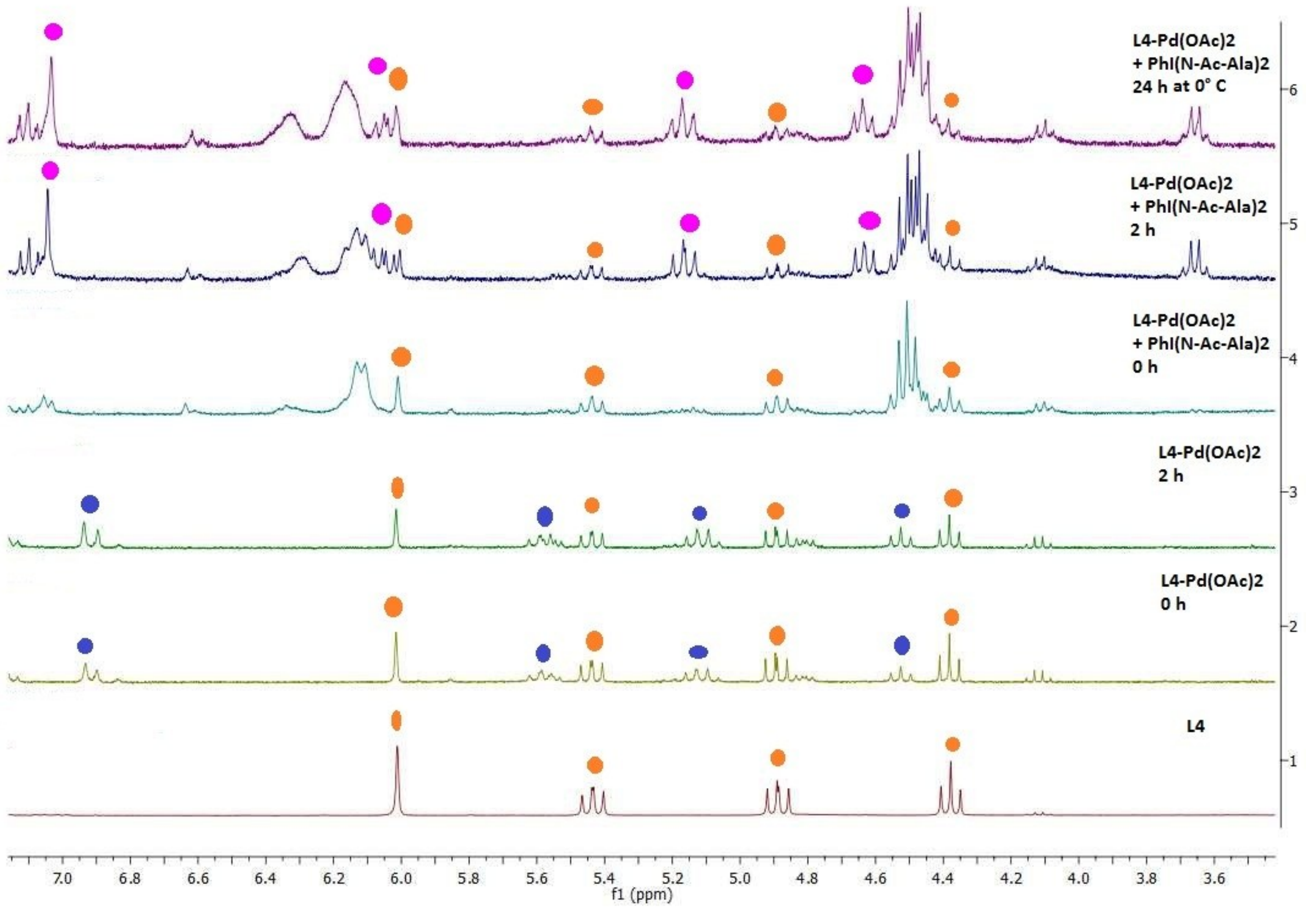
L4-Pd(OAc)2
+ PhI(N-Ac-Ala)2
24 h at 0° C
L4-Pd(OAc)2
+ PhI(N-Ac-Ala)2
2 h
L4-Pd(OAc)2
+ PhI(N-Ac-Ala)2
0 h
L4-Pd(OAc)2
2 h
L4-Pd(OAc)2
0 h
L4
6
5
4
3
2
1
7.0 6.8 6.6 6.4 6.2 6.0 5.8 5.6 5.4 5.2 5.0 4.8 4.6 4.4 4.2 4.0 3.8 3.6
f1 (ppm)

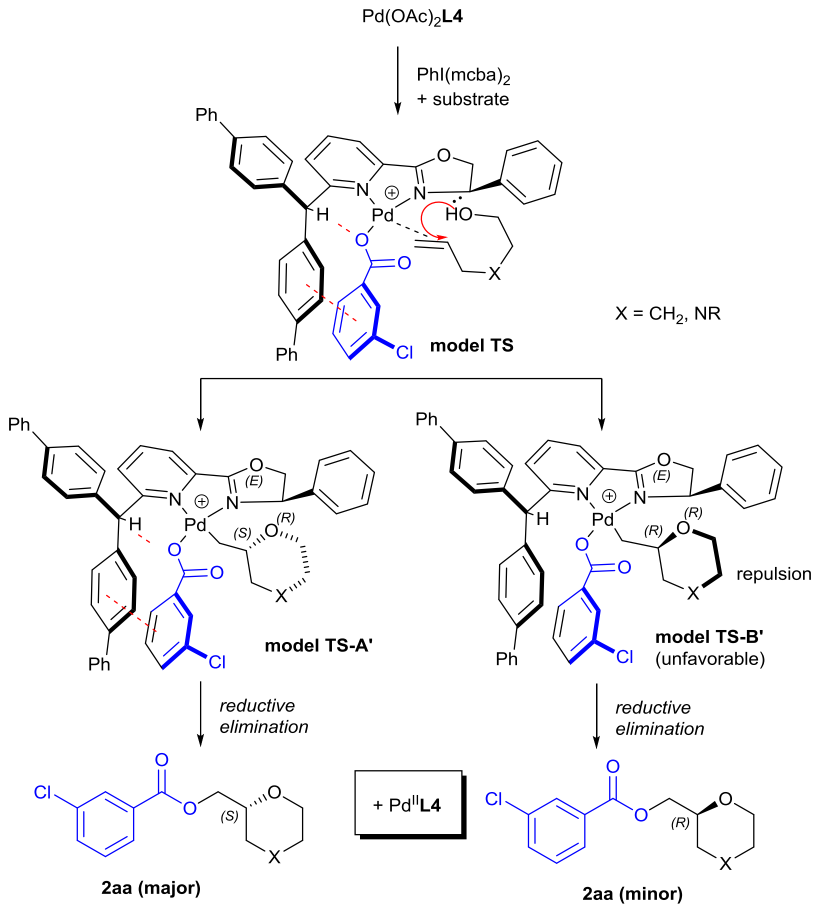

Pd(OAc)2L4
PhI(mcba)2
+ substrate
Ph
O
N
Pd
H
HO
O
O
X
Ph
Cl
model TS
X = CH2, NR
(E)
(S)
(R)
model TS-A'
(R)
model TS-B'
(unfavorable)
repulsion
reductive elimination
+ PdIIL4
2aa (major)
2aa (minor)

# Interpretation of the hypothesized alkoxypalladation step through $^1$H NOESY NMR

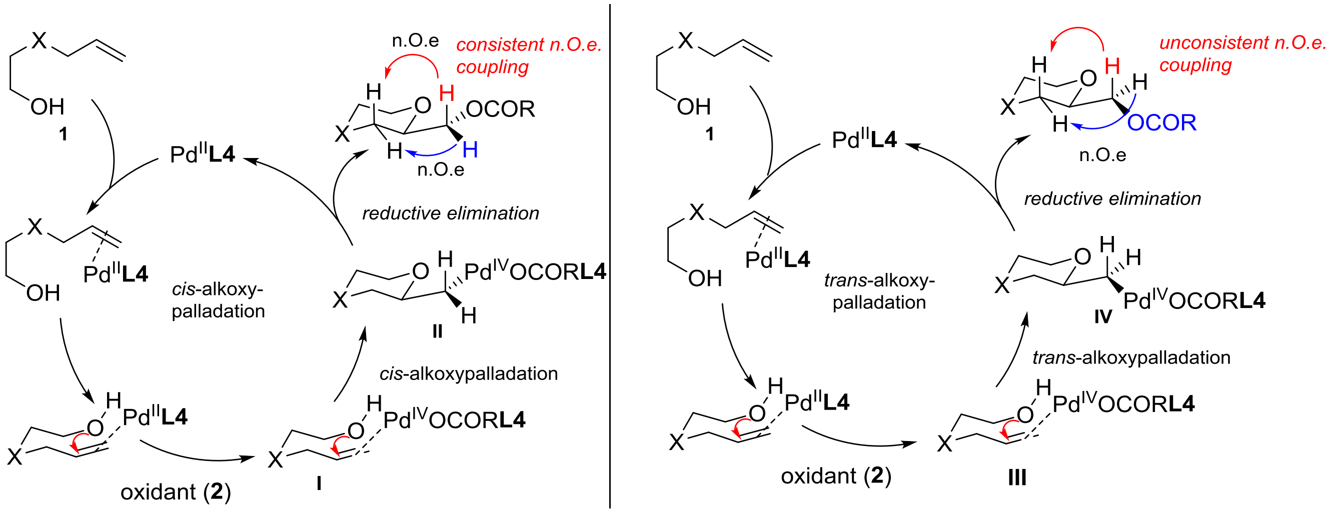


Compound **3ga**

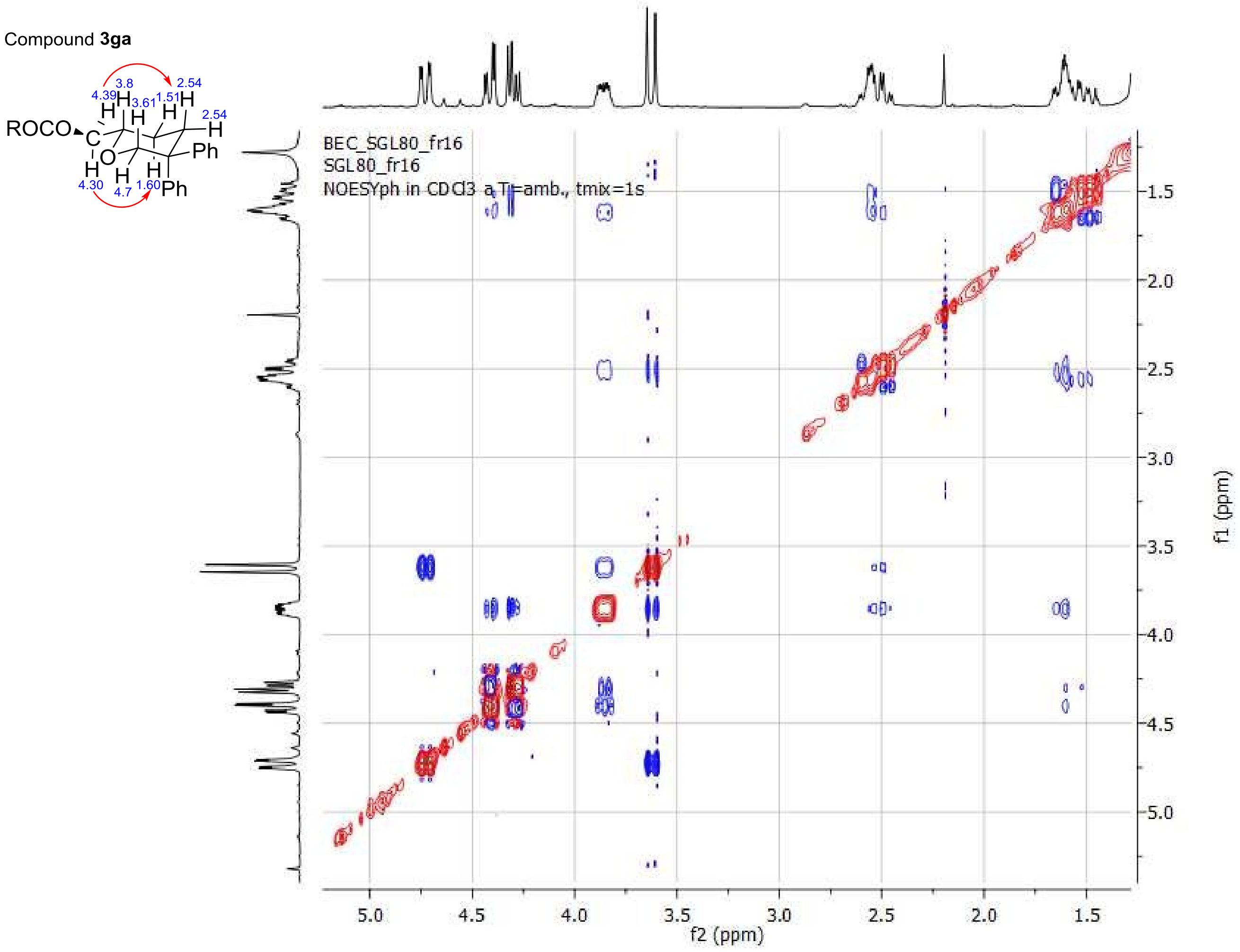

# $^{1}$H NOESY NMR couplings of the corresponding diastereoisomer formed starting from alkenols (1j) and chiral aminoethanols

NMR attributions in ppm

l-i = low-intensity

m-i = medium-intensity

h-i = high-intensity

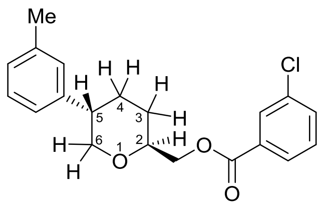


| $^{1}$H NMR value | H on C2 | $H_{axial}$ on C3 | $H_{equatorial}$ on C3 | $H_{axial}$ on C4 | $H_{equatorial}$ on C4 | $H_{equatorial}$ on C5 | $H_{axial}$ on C6 | $H_{equatorial}$ on C6 | CH (3-MeAryl) | $CH_2$-COOR | 3-Me |
|---|---|---|---|---|---|---|---|---|---|---|---|
| | 3.80 | 1.6 | 1.89 | 1.86 | 2.12 | 2.85 | 3.49 | 4.10 | 7.05 | 4.41 | 2.37 |
| NOESY coupling | 1.86 (m-i)<br>3.49 (h-i) | 1.86 (m-i)<br>1.89 (h-i)<br>2.12 (m-i)<br>2.85 (l-i)<br>4.41 (h-i) | 1.6 (h-i)<br>2.12 (m-i)<br>3.80 (m-i)<br>4.41 (m-i) | 2.12 (h-i)<br>3.49 (l-i)<br>3.80 (l-i)<br>7.05 (m-i) | 1.6 (m-i)<br>1.86 (h-i)<br>1.89 (m-i)<br>2.85 (l-i) | 1.6 (l-i)<br>2.12 (m-i)<br>4.10 (m-i)<br>7.05 (m-i) | 1.86 (l-i)<br>3.80 (m-i)<br>4.10 (h-i)<br>7.05 (m-i) | 2.85 (m-i)<br>3.49 (h-i)<br>7.05 (l-i) | 1.86 (l-i)<br>2.12 (h-i)<br>2.85 (m-i)<br>3.49 (m-i)<br>4.10 (m-i) | 1.6 (m-i)<br>1.89 (m-i) | 7.05 (m-i) |

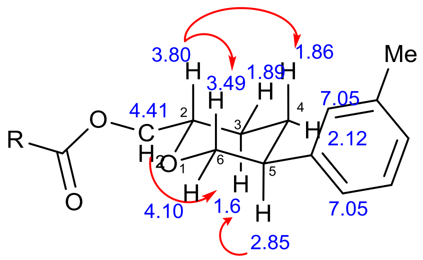


2,5-trans-disubstituted tetrahydropyran

Figure S1. a) $^{1}$H-NOESY couplings for the 2,5-trans-disubstituted tetrahydropyran structure, ***trans*-3ja**;

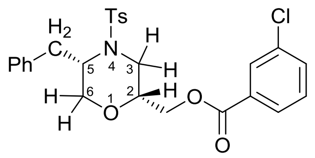

| $^1$H NMR value | H on C2 | $H_{axial}$ on C3 | $H_{equatorial}$ on C3 | $H_{equatorial}$ on C5 | $H_{axial}$ on C6 | $H_{equatorial}$ on C6 | $CH_2$-Ph | $CH_2$-COOR |
|---|---|---|---|---|---|---|---|---|
| | 3.80 | 3.19 | 3.72 | 4.07 | 3.52 | 3.76 | 2.79, 3.07 | 4.39 |
| NOESY coupling | 3.52 (l-i) | 2.79 (l-i)<br>3.07 (l-i)<br>4.39 (l-i) | 4.39 (m-i) | - | 3.80 (m-i) | 3.07 (l-i) | 3.19 (l-i)<br>3.76 (m-i for 3.07) | 3.19 (l-i)<br>3.72 (l-i) |

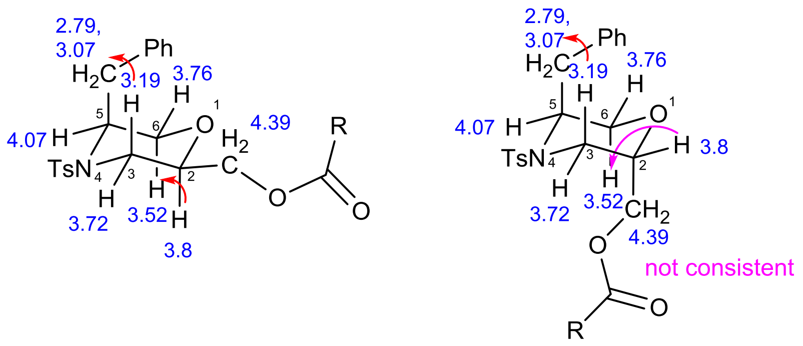


a) 2,5-cis-disubstituted morpholine  b) 2,5-trans-disubstituted morpholine

Figure S2. a) $^1$H-NOESY couplings for the 2,5-cis-disubstituted morpholine structure,**3oa**; b) contradictory $^1$H-NOESY couplings for the 2,5-trans-disubstituted morpholine structure, **3oa**.

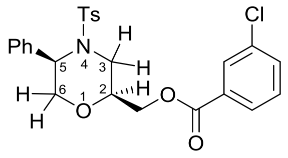

| $^1$H NMR value | H on C2 | $H_{equatorial}$ on C6 | $H_{axial}$ on C6 | $H_{axial}$ on C5 | $H_{equatorial}$ on C3 | $H_{axial}$ on C3 | $CH_2$-COOR |
|---|---|---|---|---|---|---|---|
| | 3.79 | 4.39 | 3.9 | 5.00 | 3.69 | 3.15 | 4.38, 4.29 |
| NOESY coupling | 3.69 (l-i)<br>3.9 (m-i)<br>4.29 (m-i)<br>4.38 (m-i) | 5.00 (l-i) | 3.79 (m-i)<br>5.00 (m-i) | 3.9 (h-i)<br>7.51 (m-i)<br>7.63 (m-i) | 3.79 (m-i)<br>4.29 (m-i)<br>4.38 (m-i)<br>7.63 (l-i) | 4.38 (l-i)<br>7.51 (m-i) | 3.15 (l-i)<br>3.79 (h-i) |

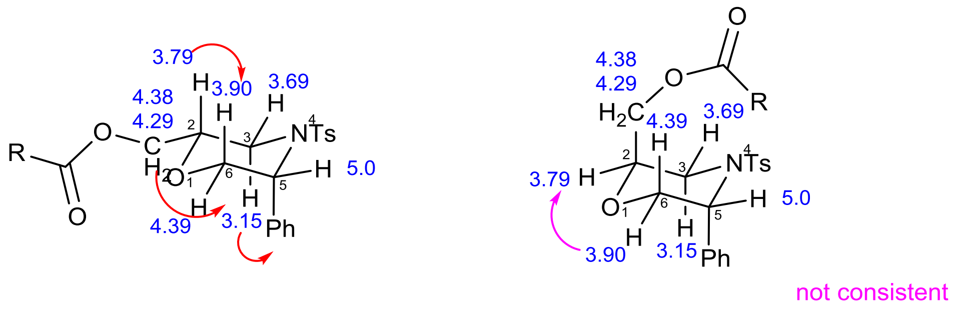


a) 2,5-trans-disubstituted morpholine  b) 2,5-cis-disubstituted morpholine

Figure S3. a) $^1$H-NOESY couplings for the 2,5-trans-disubstituted morpholine structure, **3pa**; b) contradictory $^1$H-NOESY couplings for the 2,5-cis-disubstituted morpholine structure, **3pa**.

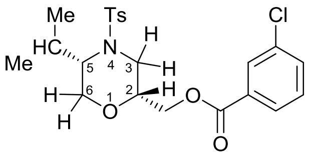

| $^1$H NMR value | H on C2 | $H_{axial}$ on C6 | $H_{equatorial}$ on C6 | $H_{equatorial}$ on C5 | $H_{axial}$ on C3 | $H_{equatorial}$ on C3 | CH-$Me_2$ | $CH_2$-COOR | $Me_2$ |
|---|---|---|---|---|---|---|---|---|---|
| | 3.46 | 3.35 | 3.96 | 3.40 | 3.09 | 3.74 | 2.25 | 4.23 | 0.95 |
| NOESY coupling | 3.35 (l-i)<br>3.74 (l-i) | 3.46 (m-i)<br>3.74 (l-i)<br>3.96 (h-i) | 0.95 (l-i)<br>3.40 (h-i) | 0.95 (h-i)<br>2.25 (m-i)<br>3.35 (l-i)<br>3.46 (m-i)<br>3.96 (h-i) | 0.95 (l-i)<br>2.25 (l-i)<br>3.46 (m-i)<br>3.74 (h-i)<br>4.23 (m-i) | 3.46 (l-i)<br>4.23 (l-i) | 0.95 (h-i)<br>3.09 (l-i)<br>3.40 (l-i)<br>3.96 (l-i) | 3.09 (l-i)<br>3.74 (l-i) | 2.25 (h-i)<br>3.09 (l-i)<br>3.40 (m-i)<br>3.96 (l-i) |

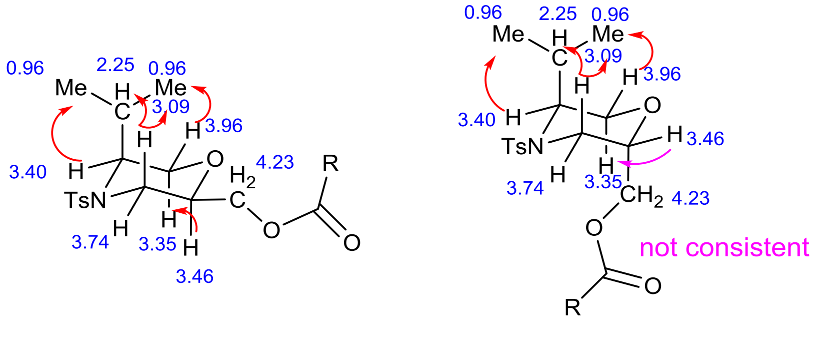


a) 2,5-cis-disubstituted morpholine  b) 2,5-trans-disubstituted morpholine

Figure S4. a) $^1$H-NOESY couplings for the 2,5-cis-disubstituted morpholine structure, **3sa**; b) contradictory $^1$H-NOESY couplings for the 2,5-trans-disubstituted morpholine structure, **3sa**.

# Behaviour of chiral aminoethanols in the presence of ligands of opposite configuration.

In order to check and observe the reactivity of chiral substrates in the presence of the chiral ligands of opposite configuration (L2, L3), a reaction in the presence of the corresponding ligand (L2 and L3, 12 mol%) was carried out according to **GP7**. In the presence of **L2**, starting from **1r** and **1s** the corresponding product was obtained in good yield at 0° C in 48 h, with a diastereoisomeric ratio of 1 to 6 between (2*S,5S*) and (2*R,5S*) according to the **GP6.** However, in the presence of **L4** the product **3ra** and **3sa** were obtained with really low yields, with the starting material still present in the reaction mixture. Opposite behavior was observed for the derivative **1p**, where in this case the higher reactivity was observed with ligand **L4.**

Basically, the use of the chiral ligand featuring the proper configuration to lead to the *anti*-disubstituted product did not afford the expected result, for this reason the reaction on the chiral aminoethanol **1o-s** was carried out in the presence of the ***rac*-L4.**

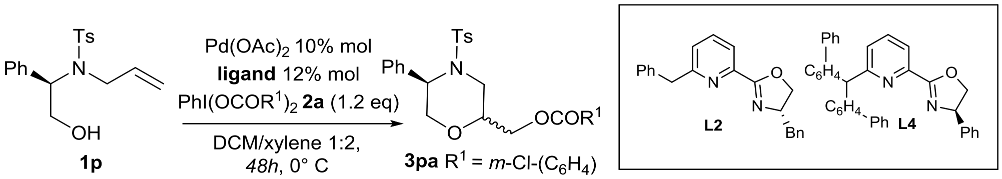


| Entry | Ligand | Time (h) | T°C | *dr* (2S,5R)/(2R,5R) | 3pa/1p |
|---|---|---|---|---|---|
| 1 | L2 | 48 h | 0 °C | 4/1 | 1/1.3 |
| 2 | L4 | 48 h | 0 °C | 9/1 | 12/1 |

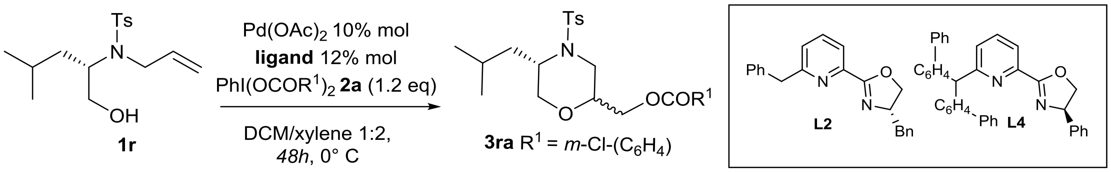


| Entry | Ligand | Time (h) | T°C | *dr* (2S,5S)/(2R,5S) | 3ra/1r |
|---|---|---|---|---|---|
| 1 | L2 | 48 h | 0 °C | 1/6 | 10/1 |
| 2 | L4 | 48 h | 0 °C | 1/3 | 1/1.6 |

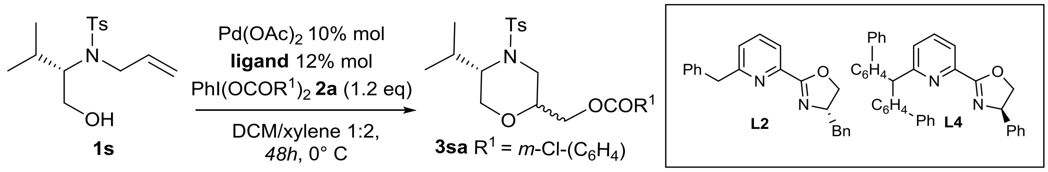


| Entry | Ligand | Time (h) | T°C | *dr* (2S,5S)/(2R,5S) | 3sa/1s |
|---|---|---|---|---|---|
| 1 | L2 | 48 h | 0 °C | 1/6 | 10/1 |
| 2 | L4 | 48 h | 0 °C | 1/2.5 | 1/1 |

## *Starting materials which did not give the desired product.*

The following substrates were not reactive in the optimized conditions. In particular, while the methyl-substituted Ts-aminoalkenes, the homoallylic derivative and the 5-hexenoic acid were not reactive, the remaining ones were mostly characterized by degradation.

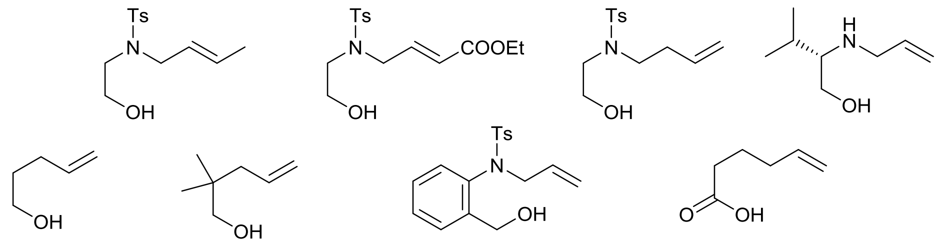


# Synthetic routes to access to (+/-)-centrolobine

In contrast to the protocols reported in literature for the synthesis of (+/-)-centrolobine, our method e) offers a shorter pathway, only 6 steps compare to the ones described by Carreno and Aidhen, route a) and c) respectively. On the other hand, the possibility to start from easily accessible substrates opens up a straightforward synthesis of centrolobine analogues.

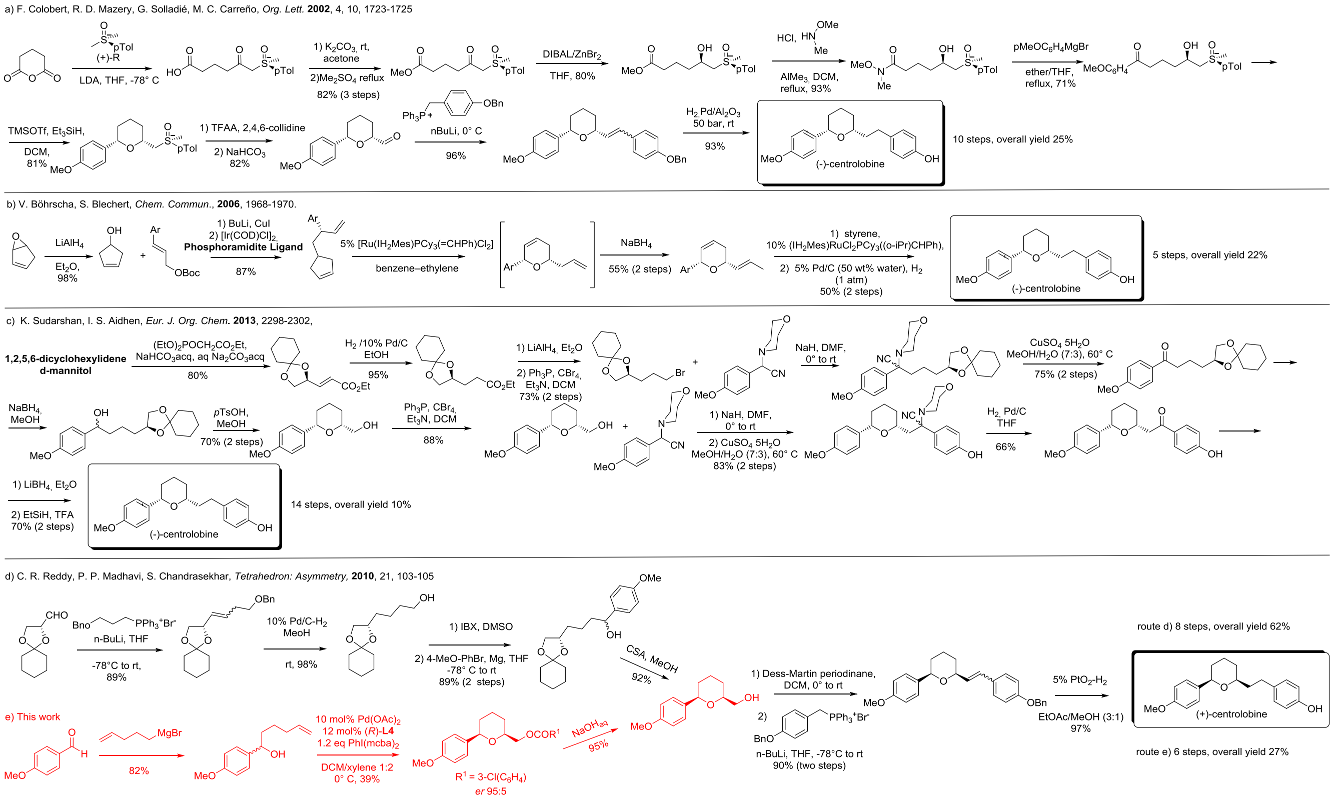

# Further Procedures and Analytical Data of Unknown Compounds

## Synthesis of 2-(di([1,1'-biphenyl]-4-yl)methyl)pyridine

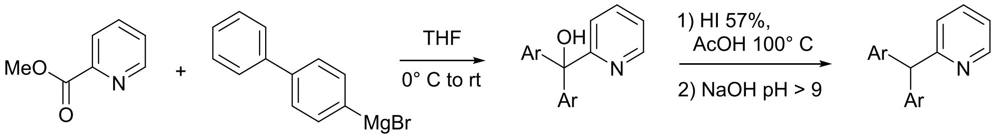


A 250 mL oven-dried flask was charged with magnesium turnings (691 mg, 28.8 mmol, 5 equiv) and THF (26 mL) under a nitrogen atmosphere, followed by adding a small amount of (1,1'-biphenyl)-4-yl bromide. After the observation of gas evolution, a solution of (1,1'-biphenyl)-4-yl bromide (Ar-Br, 3.99 g, 17 mmol, 3 equiv) in THF (16 mL) was added dropwise. The reaction mixture was heated at reflux for 5 h. The ArMgBr solution was then cooled with an ice-water bath, a solution of methyl picolinate (794 mg, 5.8 mmol, 1.00 equiv) in THF (15 mL) was added carefully. After stirring at room temperature for 1.5 hours, the reaction mixture was quenched with aqueous $NH_4Cl$ and extracted with $Et_2O$. The combined organic layers were dried over $NaSO_4$. After concentration, the residue was purified by column chromatography on silica gel (Hex/AcOEt 3:1), affording di([1,1'-biphenyl]-4-yl)(pyridin-2-yl)methanol as a white solid (2.04 g, 4.93 mmol, 85%). In a 50 mL round bottle flask, the obtained alcohol (2.04 g, 4.93 mmol) was dissolved in HOAc (14 mL), then HI (3.3 mL, 57 wt% aq) was added and the mixture was heated to 100 °C for 4 h. The mixture was cooled to 0 °C and basified to pH > 9 with NaOH (2 M aq), then diluted with ethyl acetate (45 mL), followed by washing with $NaHSO_3$ aq and brine. The combined organic layers were dried over $NaSO_4$, and concentrated. The residue was purified by column chromatography on silica gel (Hex:AcOEt 1.5:1) giving the desired product 2-(di([1,1'-biphenyl]-4-yl)methyl)pyridine as a pale white solid (1.64 g, 83%).

## 2-(di([1,1'-biphenyl]-4-yl)methyl)pyridine

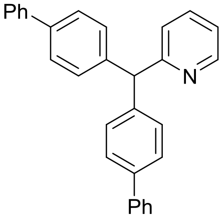


$R_f$ = 0.36 (n-Hex/EtOAc 2:1); stained with $KMnO_4$

M.p. 179° C

IR = 3027, 1585, 1483, 1139, 744 $cm^{-1}$

$^1$H NMR (300 MHz, $CDCl_3$) δ 8.94 – 8.57 (m, 1H), 7.73 – 7.54 (m, 9H), 7.45 (t, $J$ = 7.4 Hz, 4H), 7.40 – 7.29 (m, 6H), 7.26 – 7.13 (m, 2H), 5.81 (s, 1H).

$^{13}$C NMR (75 MHz, $CDCl_3$) δ 163.1 (s), 149.7 (d), 141.8 (s), 140.9 (s), 139.5 (s), 136.5 (d), 129.8 (d), 128.7 (d), 127.2 (d), 127.2 (d), 127.1 (d), 127.1 (d), 123.8 (d), 121.6 (d), 58.8 (d).

MS (ESI): m/z 398.26 $[M+H]^{+}$[i]

## ($R$)-2-(6-benzylpyridin-2-yl)-4-phenyl-4,5-dihydrooxazole (L1)

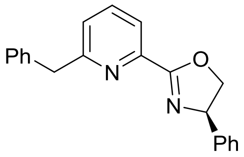


Compound **L1** was prepared according to the general procedure (GP1) and isolated as white solid (overall yield 69%) after flash chromatography (n-Hex/AcOEt 6:1 → 4:1) for the first purification step and (n-Hex/AcOEt 3:1) for the second one.

The data are in good agreement with those reported in the literature.[5]

## ($S$)-4-benzyl-2-(6-benzylpyridin-2-yl)-4,5-dihydrooxazole (L2)

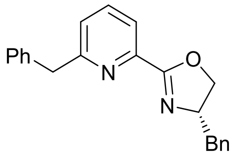


Compound **L2** was prepared according to the general procedure (GP1) and isolated as yellow oil (overall yield 58%) after flash chromatography (Hex:AcOEt 8:1) for the first purification step and (Hex:AcOEt 2:1) for the second one.

$R_f$ = 0.14 (n-Hex/EtOAc 2:1); stained with $KMnO_4$

IR = 1645, 1491, 1449, 1107, 695 $cm^{-1}$

$^1$H NMR (300 MHz, $CDCl_3$) δ 7.92 (d, $J$ = 7.7 Hz, 1H), 7.67 (t, $J$ = 7.8 Hz, 1H), 7.41 – 7.22 (m, 10H), 7.14 (d, $J$ = 7.8 Hz, 1H), 4.82 – 4.59 (m, 1H), 4.48 (t, $J$ = 9.0 Hz, 1H), 4.37 – 4.22 (m, 3H), 3.35 (dd, $J$ = 13.7, 4.9 Hz, 1H), 2.78 (dd, $J$ = 13.7, 9.2 Hz, 1H).

$^{13}$C NMR (75 MHz, $CDCl_3$) δ 163.3 (s), 161.6 (s), 146.2 (s), 139.0 (s), 137.9 (s), 137.0 (d), 129.4 (d), 129.2 (d), 128.7 (d), 128.6 (d), 126.6 (d), 126.6 (d), 125.2 (d), 121.7 (d), 72.6 (t), 68.1 (d), 44.7 (t), 41.7 (t).

MS (ESI): m/z 329.20 $[M+H]^+$

$[\alpha]^{D}_{20}$: +9° (c: 0.01 in $CHCl_3$)

## ($R$)-2-(6-benzhydrylpyridin-2-yl)-4-phenyl-4,5-dihydrooxazole (L3)

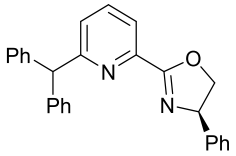


Compound **L3** was prepared according to the general procedure (GP1) and isolated as white solid (overall yield 63%) after flash chromatography (Hex:AcOEt 8:1) for the first purification step and (Hex:AcOEt 4:1) for the second one

[5] X. Qi, C. Chen, C. Hou, L. Fu, P. Chen, G. Liu, *J. Am. Chem. Soc.*, **2018**, 140 (24), 7415-7419

The data are in good agreement with those reported in the literature.[1]

## (*R*)-2-(6-(di([1,1'-biphenyl]-4-yl)methyl)pyridin-2-yl)-4-phenyl-4,5-dihydrooxazole (L4)

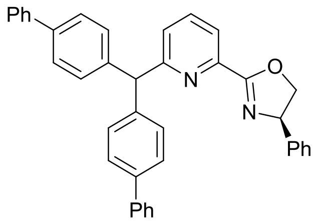

Compound **L4** was prepared according to the general procedure (GP1) and isolated as white solid (overall yield 66%) after crystallization (n-Hex/AcOEt).

$R_f$ = 0.26 (n-Hex/EtOAc 2:1); stained with $KMnO_4$

M.p. 228° C

IR = 1637, 1486, 1110, 747, 732 $cm^{-1}$

$^1$H NMR (300 MHz, $CDCl_3$) δ 8.12 (d, $J$ = 7.6 Hz, 1H), 7.79 (t, $J$ = 7.8 Hz, 1H), 7.66 – 7.53 (m, 7H), 7.51 – 7.24 (m, 14H), 6.04 (s, 1H), 5.46 (dd, $J$ = 10.2, 8.7 Hz, 1H), 4.92 (dd, $J$ = 10.3, 8.7 Hz, 1H), 4.41 (t, $J$ = 8.6 Hz, 1H).

$^{13}$C NMR (75 MHz, $CDCl_3$) δ 164.1 (s), 163.4 (s), 146.6 (s), 141.9 (s), 141.6 (s), 140.8 (s), 139.5 (s), 137.0 (d), 137.0 (d), 129.8 (d), 128.7 (d), 127.7 (d), 127.21 (d), 127.0 (d), 126.8 (d), 126.1 (d), 126.1 (d), 122.5 (d), 75.46 (t), 70.3 (d), 58.7 (d)

MS (ESI): m/z 543,31 $[M+H]^+$

$[\alpha]^D_{20}$: -17° (c: 0.01 in $CHCl_3$)

## phenyl-λ3-iodanediyl bis(3-chlorobenzoate) (2a)

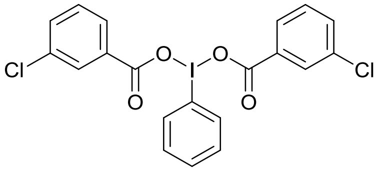

Compound **2a** was prepared according to the general procedure (GP2) and isolated as white solid (yield 97%).

The data are in good agreement with those reported in the literature.[6]

## phenyl-λ3-iodanediyl bis(2-fluorobenzoate) (2c)

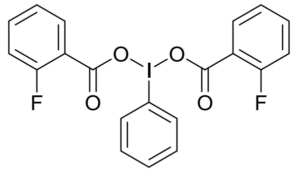

[6] K. Muñiz B. García C. Martínez, A. Piccinelli, *Chem Eur J,* **2017**, 23(7), 1539-1545

Compound **2c** was prepared according to the general procedure (GP2) and isolated as white solid (yield 95%).

The data are in good agreement with those reported in the literature. [6]

## phenyl-λ3-iodanediyl (2*S*,2'*S*)-bis(2-acetamidopropanoate) (2d)

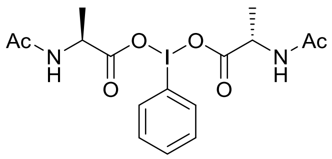


Compound **2d** was prepared according to the general procedure (GP2) and isolated as white solid (yield 83%).

The data are in good agreement with those reported in the literature.[7]

## *N*-allyl-*N*-(2-hydroxyethyl)-4-methylbenzenesulfonamide (1a)

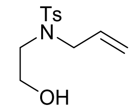


Compound **1a** was prepared according to the general procedure (GP3) and isolated as a colourless gum (overall yield 79%) after flash chromatography (Hex/AcOEt 2:1 → 1:1).

The data are in good agreement with those reported in the literature.[8]

## Synthesis of *tert*-butyl allyl(2-hydroxyethyl)carbamate (1b)

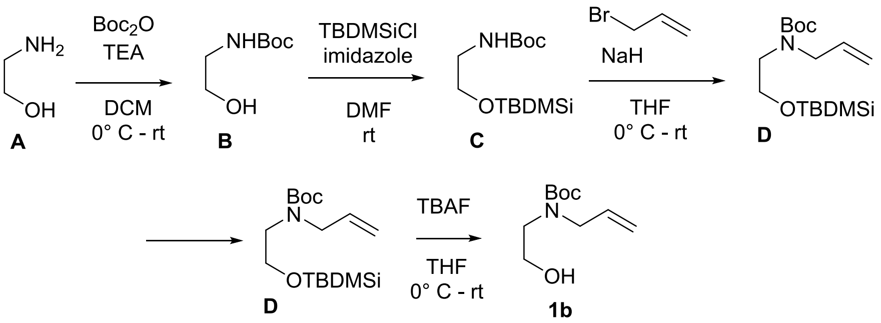


To a two-neck round-bottom flask 2-aminoethanol **A** (610 mg, 10 mmol, 1 eq) was added and dissolved in DCM (12 mL, 0.2 M). After cooling to 0° C, di-*tert*-butyl dicarbonate (2616 mg, 12 mmol, 1.2 eq) was added, followed by trimethylamine (1.6 mL, 12 mmol, 1.2 eq). The solution was allowed to warm to rt and stirred for 24 h. The reaction mixture was washed with saturated solution of $NH_4Cl$ until pH~7 and with brine (20 mL). Then, the organic phase was dried over $Na_2SO_4$ and the solvent evaporated under reduced pressure. The crude product was purified by flash chromatography on silica gel (n-Hex/EtOAc 1:1) affording the compound **B** (1384 mg, 8.6 mmol, 86%) as a colourless oil.

---

[7] *Zhurnal Organicheskoi Khimii,* **1975,** 11 (6), 1259-1263

[8] M. Poornachandran, R. Raghunathan, Tetrahedron **2008**, 64 (27), 6461-6474

A solution of *N*-protected aminoalcohol **B** (8.6 mmol, 1 eq), tert-butyldimethylsilyl chloride (8.6 mmol, 1 eq) and imidazole (8.6 mmol, 1 eq) in DMF (0.2 M) was stirred at room temperature overnight. The reaction was taken up with brine (40 mL), extracted with $Et_2O$ (3 x 40 mL), dried over $Na_2SO_4$ and concentrated at reduced pressure. The crude product was purified by silica gel column chromatography gel (n-Hex/EtOAc 4:1) to afford **C** (1521 mg, 5.3 mmol, 62%).

To a mixture of sodium hydride (7.95 mmol, 1.5 eq) in THF (16 mL) at 0° C under nitrogen atmosphere, a solution of **C** (1521 mg, 5.3 mmol, 1 eq) in THF (16 mL) was dropped. The reaction mixture was stirred for 30 min at room temperature, then cooled at 0 °C. A solution of allyl bromide (5.56 mmol, 1.05 eq) in THF (5 mL) was dropped and the mixture was stirred at room temperature overnight. The mixture was concentrated at reduced pressure, washed with water (20 mL), extracted with $Et_2O$ (3 x 60 mL), dried on $Na_2SO_4$, and concentrated at reduced pressure. The crude product was purified by silica gel column chromatography (Hex:AcOEt 6:1) to afford **D** (1003 mg, 3.07 mmol, 58%).

A mixture of the corresponding **D** (1003 mg, 3.07 mmol, 1 eq) and tetrabutylammonium fluoride (3.07 mmol, 1.2 eq) in THF (0.2 M) was stirred at room temperature for 2 h. The solvent was evaporated under reduced pressure, water was added (10 mL) and the reaction mixture was extracted with DCM (3 x 20 mL). Then the organic phase was dried over $Na_2SO_4$, and the solvent concentrated at reduced pressure. The crude product was purified by silica gel column chromatography (Hex:AcOEt 3:1 → 1.5:1) to afford **1b** (530 mg, 2.64 mmol, 85%) as a colourless gum.

## ***tert*-butyl allyl(2-hydroxyethyl)carbamate (1b)**

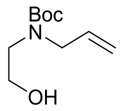

The data are in good agreement with those reported in the literature.[9]

## ***N*-allyl-*N*-(1-hydroxy-2-methylpropan-2-yl)-4-methylbenzenesulfonamide (1c)**

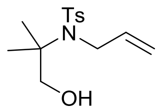

Compound **1c** was prepared according to the general procedure (GP3) and isolated as a colourless gum (overall yield 47%) after flash chromatography (Hex/AcOEt 3:1).

The data are in good agreement with those reported in the literature.[10]

## ***N*-allyl-*N*-(2-hydroxyethyl)-4-nitrobenzenesulfonamide (1d)**

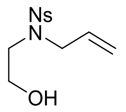

[9] M. Schuster, J. Pernerstorfer, S. Blechert, *Angew Chem, Int Ed,* **1996**, 35 (17) 1979-1980

[10] H. Ohno, Y. Takeoka, Y. Kadoh, K. Miyamura, T. Tanaka, *Journal of Organic Chemistry* **2004**, 69(13), 4541-4544

Compound **1d** was prepared according to the general procedure (GP3) and isolated as a pale-yellow solid (overall yield 62%) after flash chromatography (Hex/AcOEt 4:1 → 2:1).

The data are in good agreement with those reported in the literature.[11]

## hex-5-en-1-ol (1e)

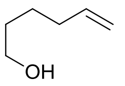


Compound **1e** was purchased from Sigma Aldrich. CAS: 821-41-0.

## 2,2-dimethylhex-5-en-1-ol (1f)

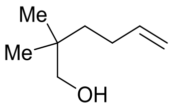


Compound **1f** was prepared according to the general procedure (GP4) and isolated as a colourless oil (overall yield 63%) after flash chromatography (n-Hex → n-Hex:$Et_2O$ 1:2).

The data are in good agreement with those reported in the literature.[3]

## 2,2-dimethylhex-5-en-1-ol (1g)

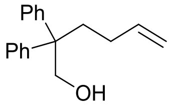


Compound **1g** was prepared according to the general procedure (GP4) and isolated as a colourless oil (overall yield 68%) after flash chromatography (n-Hex → n-Hex:$Et_2O$ 1:2).

The data are in good agreement with those reported in the literature.[12] CAS: 167300-48-3.

## Synthesis of 2,2-dipropylhex-5-en-1-ol (1h)

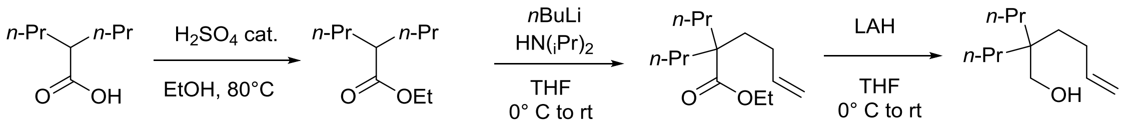


To a solution of valproic acid (800 mg, 5.2 mmol) in EtOH (5 ml), $H_2SO_4$ (55 μL, 1.04 mmol, 20 mol%) was added. The reaction was heated at 80° C overnight and then the solvent evaporated. The crude residue was rinsed with EtOAc and washed with $NaHCO_3$ until pH = 7/8. The organic layer was dried over $Na_2SO_4$, filtered and concentrated in vacuo to afford ethyl valproate, used in the next step without any further purification. 2.5 M solution of n-BuLi in hexane (3.8 mL, 9.24 mmol) was added to a stirred solution of diisopropyl amine (1.4 mL, 9.66 mmol) in THF (10 mL) at 0°C under argon

[11] S. Gazzola, E. M. Beccalli, T. Borelli, C. Castellano, D. Diamante, G. Broggini, *Synlett* **2018**, 29 (4), 503-508

[12] S. Fujita, M. Abe, M. Shibuya, Y. Yamamoto, *Org. Lett.* **2015**, 17, 3822

atmosphere. After 30 min, ethyl valproate (735 mg, 4.2 mmol) was added to the reaction mixture at 0°C. After 30 min, 4-bromobutene (0.5 mL, 5.04 mmol) was added to the mixture at 0°C and then warmed to rt. After 10 h, the mixture diluted with diethyl ether (8 mL) and saturated $NH_4Cl$ solution (10 mL) was added. The aqueous layer was extracted with diethyl ether (10 mL x 3). Combined organic layer was dried over $Na_2SO_4$, filtered and concentrated in vacuo. 1.0 M solution of LAH in THF (11mL, 10.92 mmol) was added to a solution of crude acid in dry THF (11 mL) at 0°C and then warmed to rt. After 1 h, water (0.5 mL), 20% NaOH (1 mL) and water (1.5 mL) were added to the mixture. The white solid was filtered over celite pad and the filtrate was concentrated in vacuo. The crude was purified by flash column chromatography on silica gel (n-Hex → n-Hex:$Et_2O$ 1:2) to afford alcohol (670 mg, 3.90 mmol, 75%).

## 2,2-dipropylhex-5-en-1-ol (1h)

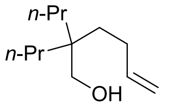


$R_f$ = 0.49 (n-Hex/$Et_2O$ 1:2); stained with Molybdenum

IR = 3356, 2956, 1456, 1033, 907 $cm^{-1}$

$^1H$ NMR (300 MHz, CDCl3) δ 5.81 (ddt, $J$ = 16.8, 10.1, 6.5 Hz, 1H), 5.16 – 4.78 (m, 2H), 3.36 (s, 2H), 2.10 – 1.81 (m, 2H), 1.42 – 1.07 (m, 12H), 0.89 (t, $J$ = 6.4 Hz, 7H).

$^{13}C$ NMR (75 MHz, CDCl3) δ 139.5 (d), 113.9 (t), 67.1 (t), 39.7 (s), 36.5 (t), 33.3 (t), 27.5 (t), 16.2 (t), 15.0 (q)·

MS (ESI): m/z 186.43 $[M+H]^+$ 207.36 $[M+Na]^+$ 392.31 $[2M+Na]^+$

## (1-(but-3-en-1-yl)cyclohexyl)methanol (1i)

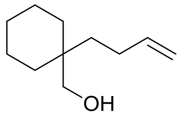


Compound **1i** was prepared according to the general procedure (GP4) and isolated as a light-yellow oil (overall yield 56%).

The data are in good agreement with those reported in the literature.[13]

## 2-(*m*-tolyl)hex-5-en-1-ol (1j)

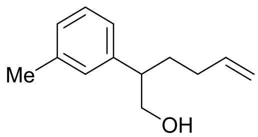


Compound **1j** was prepared according to the general procedure (GP4) and isolated as a colourless oil (overall yield 59%) after flash chromatography (n-Hex → n-Hex/$Et_2O$ 1:2).

$R_f$ = 0.42 (n-Hex/$Et_2O$ 1:2); stained with Molybdenum

[13] D. L. J. Clive, M. P. Pham, R. Subedi, *J. Am. Chem. Soc.* **2007**, *129, 9*, 2713–2717

IR = 3343, 2924, 1640, 1607, 1453, 1033, 704 $cm^{-1}$

$^{1}H$ NMR (300 MHz, $CDCl_3$) δ 7.34 – 7.14 (m, 1H), 7.04 (dt, *J* = 14.8, 3.8 Hz, 3H), 5.93 – 5.54 (m, 1H), 5.12 – 4.74 (m, 2H), 3.85 – 3.55 (m, 2H), 2.77 (dtd, *J* = 9.5, 7.5, 5.7 Hz, 1H), 2.35 (s, 3H), 2.16 – 1.90 (m, 2H), 1.86 – 1.56 (m, 2H).

$^{13}C$ NMR (75 MHz, $CDCl_3$) δ 142.0 (s), 138.4 (d), 138.2 (s), 128.9 (d), 128.5 (d), 127.5 (d), 125.0 (d), 114.7 (t), 67.4 (t), 48.0 (d), 31.3 (t), 31.2 (t), 21.4 (q).

MS (ESI): m/z 191.46 $[M+H]^+$

## 2-(benzo[d][1,3]dioxol-5-yl)hex-5-en-1-ol (1k)

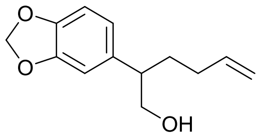


Compound **1k** was prepared according to the general procedure (GP4) and isolated as a yellow oil (overall yield 76%).

$R_f$ = 0.23 (n-Hex/$Et_2O$ 2:1); stained with Molybdenum

IR = 3383, 2925, 1639, 1486, 1242, 908, 729 $cm^{-1}$

$^{1}H$ NMR (300 MHz, $CDCl_3$) δ 6.83 – 6.52 (m, 3H), 5.91 (s, 2H), 5.74 (ddt, J = 16.9, 10.3, 6.6 Hz, 1H), 4.94 (ddd, J = 10.3, 6.3, 1.3 Hz, 2H), 3.84 – 3.51 (m, 2H), 2.80 – 2.52 (m, 1H), 2.04 – 1.47 (m, 4H).

$^{13}C$ NMR (75 MHz, $CDCl_3$) δ 147.9 (s), 146.3 (s), 138.3 (d), 136.0 (s), 121.3 (d), 114.7 (t), 108.3 (d), 107.9 (d), 100.9 (t), 67.4 (t), 47.7 (d), 31.3 (t), 31.3 (t)

MS (ESI): m/z 219.48 $[M-H]^-$

## 2-(4-methoxyphenyl)hex-5-en-1-ol (1l)

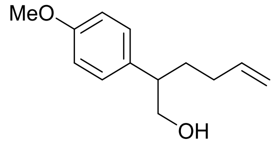


Compound **1l** was prepared according to the general procedure (GP4) and isolated as a white solid (overall yield 67%) after flash chromatography (n-Hex → n-Hex/$Et_2O$ 1:2).

M.p. 44-46° C

$R_f$ = 0.21 (n-Hex/$Et_2O$ 2:1); stained with Molybdenum

IR = 3442, 2924, 1611, 1513, 1249, 1029, 920, 813 $cm^{-1}$

$^{1}H$ NMR (400 MHz, $CDCl_3$) δ 7.19 – 7.06 (m, 2H), 6.95 – 6.83 (m, 2H), 5.79 (ddt, *J* = 17.0, 10.3, 6.6 Hz, 1H), 5.06 – 4.88 (m, 2H), 3.82 (s, 3H), 3.72 (ddd, *J* = 18.6, 10.7, 6.9 Hz, 2H), 2.90 – 2.68 (m, 1H), 2.11 – 1.89 (m, 2H), 1.89 – 1.55 (m, 2H).

$^{13}C$ NMR (101 MHz, $CDCl_3$) δ 158.8 (s), 138.4 (d), 133.9 (s), 129.0 (d), 114.7 (t), 114.1 (d), 67.6 (t), 55.3 (q), 47.2 (d), 31.4 (t), 31.3 (t).

MS (ESI): m/z 207.44 $[M+H]^+$

## 1-(4-methoxyphenyl)hex-5-en-1-ol (1m)

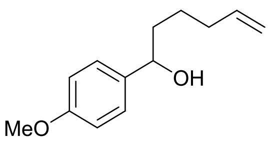


Compound **1m** was prepared according to the general procedure (GP5) and isolated as a colourless oil (yield 82%) after flash chromatography (n-Hex → n-Hex/$Et_2O$ 1:2).

The data are in good agreement with those reported in the literature.[14]

## (E)-1-(2-methoxyphenyl)octa-1,7-dien-3-ol (1n)

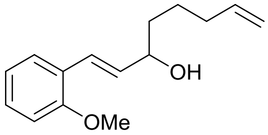


Compound **1n** was prepared according to the general procedure (GP5) and isolated as a yellow oil (yield 67%) after flash chromatography (n-Hex → n-Hex/$Et_2O$ 1:2).

$R_f$ = 0.24 (n-Hex/$Et_2O$ 2:1); stained with Molybdenum

IR = 3367, 2934, 1597, 1488, 1240, 1027, 972, 748 $cm^{-1}$

$^1$H NMR (300 MHz, $CDCl_3$) δ 7.44 (dd, $J$ = 7.6, 1.6 Hz, 1H), 7.30 – 7.12 (m, 1H), 7.08 – 6.72 (m, 3H), 6.23 (dd, $J$ = 16.0, 7.0 Hz, 1H), 5.82 (ddt, $J$ = 16.9, 10.2, 6.7 Hz, 1H), 5.15 – 4.75 (m, 2H), 4.43 – 4.17 (m, 1H), 3.85 (s, 3H), 2.33 – 1.87 (m, 2H), 1.87 – 1.30 (m, 4H).

$^{13}$C NMR (75 MHz, $CDCl_3$) δ 156.8 (s), 138.6 (d), 133.1 (d), 128.7 (d), 126.9 (d), 125.7 (s), 125.2 (d), 120.6 (d), 114.6 (t), 110.9 (d), 73.4 (d), 55.4 (q), 36.75 (t), 33.6 (t), 24.8 (t).

MS (ESI): m/z 255.49 $[M+Na]^+$, 487.42 $[2M+Na]^+$

## (*S*)-*N*-allyl-*N*-(1-hydroxy-3-phenylpropan-2-yl)-4-methylbenzenesulfonamide (1o)

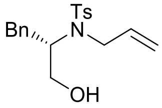


Compound **1o** was prepared according to the general procedure (GP3) and isolated as a colourless gum (overall yield 72%) after flash chromatography (Hex/AcOEt 2:1).

The data are in good agreement with those reported in the literature.[15]

---

[14] P.A. Barlett, K.L. McLaren, P.C. Ting, *J. Am. Chem. Soc.* **1988**, 110, 1634-1635

[15] M. Poornachandran, R. Raghunathan, *Tetrahedron: Asymmetry* **2008**, 19 (18), 2177-2183

### (*R*)-*N*-allyl-*N*-(2-hydroxy-1-phenylethyl)-4-methylbenzenesulfonamide (1p)

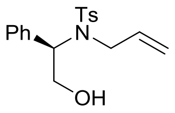


Compound **1p** was prepared according to the general procedure (GP3) and isolated as a colourless oil (overall yield 64%) after flash chromatography (Hex/AcOEt 3:1).

The data are in good agreement with those reported in the literature.[16]

### (*S*)-*N*-allyl-*N*-(1-hydroxybutan-2-yl)-4-methylbenzenesulfonamide (1q)

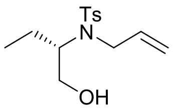


Compound **1q** was prepared according to the general procedure (GP3) and isolated as a colourless oil (overall yield 67%) after flash chromatography (Hex/AcOEt 3:1).

The data are in good agreement with those reported in the literature.[15]

### (*S*)-*N*-allyl-*N*-(1-hydroxy-4-methylpentan-2-yl)-4-methylbenzenesulfonamide (1r)

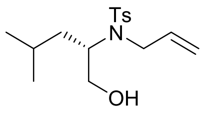


Compound **1r** was prepared according to the general procedure (GP3) and isolated as a white solid (overall yield 59%) after flash chromatography (Hex/AcOEt 3:1).

The data are in good agreement with those reported in the literature.[16]

### (*S*)-*N*-allyl-*N*-(1-hydroxy-3-methylbutan-2-yl)-4-methylbenzenesulfonamide (1s)

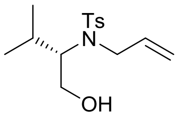


Compound **1s** was prepared according to the general procedure (GP3) and isolated as a colourless oil (overall yield 59%) after flash chromatography (Hex/AcOEt 3:1).

The data are in good agreement with those reported in the literature.[16]

---

[16] F.C. Sequeira, S. R. Chemler, *Org. Lett.*, **2012**, 141 (7), 4482-4485

## (S) (4-tosylmorpholin-2-yl)methyl 3-chlorobenzoate (3aa)

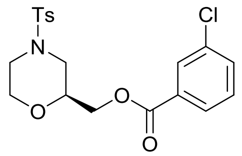


Compound **3aa** was prepared according to the general procedure (GP7) and isolated as colourless oil (yield 79%) after flash chromatography (Hex/AcOEt 2:1).

$R_f$ = 0.23 (n-Hex/EtOAc 2:1); stained with $KMnO_4$

IR = 2918, 1721, 1347, 1261, 1161, 744 $cm^{-1}$

$^1H$ NMR (400 MHz, $CDCl_3$) δ 7.97 (t, $J$ = 1.9 Hz, 1H), 7.90 (dt, $J$ = 7.8, 1.3 Hz, 1H), 7.65 (d, $J$ = 8.3 Hz, 2H), 7.55 (ddd, $J$ = 8.0, 2.1, 1.1 Hz, 1H), 7.42 – 7.31 (m, 3H), 4.40 – 4.22 (m, 2H), 4.01 – 3.87 (m, 2H), 3.79 – 3.63 (m, 2H), 3.59 – 3.45 (m, 1H), 2.51 – 2.40 (m, 4H), 2.34 – 2.20 (m, 1H).

$^{13}C$ NMR (101 MHz, $CDCl_3$) δ 165.1, 144.2, 134.7, 133.4, 132.2, 131.5, 129.9, 129.9, 128.0, 73.2, 66.0, 65.1, 47.6, 45.5, 21.6.

MS (ESI): m/z 410.21 $[M+H]^+$

From GP7. *Reaction conditions*: $Pd(OAc)_2$ 10 mol%, **L4** 12 mol% in xylene/DCM 2:1 (0.3 M) T = 0° C. *er* 88:12. HPLC (Amylose column 20cm, 5μm, l = 230 nm, n-Hex/iPrOH = 70:30, 1 mL/min): tR = 8.4 min (major), 10.8 min (minor).

$[\alpha]^D_{20}$: -21° (c: 0.001 in $CHCl_3$)

## 4-tosylmorpholin-2-yl)methyl acetate (3ab)

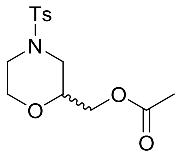


Compound **3ab** was prepared according to the general procedure (GP7) and isolated as colourless oil (yield 57%) after flash chromatography (n-Hex:AcOEt 2:1).

The data are in good agreement with those reported in the literature.[11]

From GP7. *Reaction conditions*: $Pd(OAc)_2$ 10 mol%, **L4** 12 mol% in xylene/DCM 2:1 (0.3 M) T = 0° C. *er* 67:33. HPLC (Amylose column 20 cm, 3μm, l = 230 nm, n-Hex/iPrOH = 70:30, 0.5 mL/min): tR = 20.1 min (major), 24.9 min (minor).

## (*S*)-(4-tosylmorpholin-2-yl)methyl 2-fluorobenzoate (3ac)

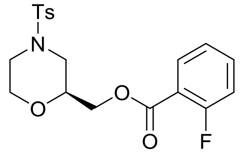


Compound **3ac** was prepared according to the general procedure (GP7) and isolated as colourless oil (yield 81%) after flash chromatography (n-Hex:AcOEt 3:1).

$R_f$ = 0.29 (n-Hex/EtOAc 2:1); stained with $KMnO_4$

IR = 1708, 1297, 1162, 991, 752 $cm^{-1}$

$^1H$ NMR (400 MHz, $CDCl_3$) δ δ 7.92 (td, $J$ = 7.6, 1.8 Hz, 1H), 7.67 (d, $J$ = 8.2 Hz, 2H), 7.61 – 7.49 (m, 1H), 7.37 (d, $J$ = 8.0 Hz, 2H), 7.29 – 7.05 (m, 2H), 4.33 (qd, $J$ = 11.7, 5.2 Hz, 2H), 4.02 – 3.86 (m, 2H), 3.82 – 3.66 (m, 2H), 3.63 – 3.49 (m, 1H), 2.54 – 2.41 (m, 4H), 2.38 – 2.20 (m, 1H).

$^{13}C$ NMR (101 MHz, $CDCl_3$) δ [164.0, 163.9] (s), 160.2 (s), 144.1 (s), [134.9, 134.8] (d), 132.2 (s), 131.9 (s), 129.8 (d), 127.9 (d), [124.1, 124.0] (d), [117.2, 116.9] (d), 72.9 (d), 66.0 (t), 64.9 (t), 47.6 (t), 45.4 (t), 21.6 (q).

MS (ESI): m/z 394.26 $[M+H]^+$

$[\alpha]^D{}_{20}$: -43° (c: 0.001 in $CHCl_3$)

From GP7. *Reaction conditions*: $Pd(OAc)_2$ 10 mol%, **L4** 12 mol% in xylene/DCM 2:1 (0.3 M) T = 0° C. *er* 83:17. HPLC (Amylose column 20 cm, 3µm, l = 230 nm, n-Hex/iPrOH = 70:30, 0.5 mL/min): tR = 43.5 min (minor), 49.8 min (major).

$[\alpha]^D{}_{20}$: -32° (c: 0.01 in $CHCl_3$)

## (4-tosylmorpholin-2-yl)methyl acetyl-*L*-alaninate (3ad)

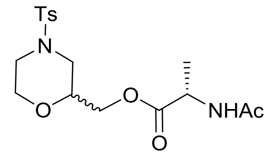


Compound **3ad** was prepared according to the general procedure (GP7) and isolated as colourless gum (yield 52%) after flash chromatography (n-Hex:AcOEt 2:1 → 1:2).

$R_f$ = 0.05 (n-Hex/EtOAc 1:2)

IR = 2921, 1743, 1656, 1339, 1161, 752 $cm^{-1}$

$^1H$ NMR (300 MHz, $CDCl_3$) δ 7.63 (d, $J$ = 8.0 Hz, 2H), 7.35 (d, $J$ = 8.3 Hz, 2H), 6.04 (d, $J$ = 5.6 Hz, NH), 4.58 (pd, $J$ = 7.2, 1.8 Hz, 1H), 4.25 – 4.03 (m, 2H), 3.99 – 3.87 (m, 1H), 3.85 – 3.42 (m, 4H), 2.49 – 2.35 (m, 3H), 2.19 (dd, $J$ = 21.5, 10.4 Hz, 2H), 2.00 (d, $J$ = 4.1 Hz, 3H), 1.37 (d, $J$ = 7.2 Hz, 3H).

$^{13}C$ NMR (75 MHz, $CDCl_3$) δ 172.7 (s), 169.5 (s), 144.1 (s), 132.2 (s), 129.8 (d), 127.8 (d), [72.9, 72.8] (t), 65.8 (t), 64.8 (t), 48.0 (d), [47.24, 47.18] (t), 45.4 (t), 23.0 (q), 21.5 (q), 18.4 (q).

MS (ESI): m/z 407.33 $[M+Na]^+$

From GP7. *Reaction conditions*: $Pd(OAc)_2$ 10 mol%, **L4** 12 mol% in DCM (0.3 M) T = rt. Racemic. (The yields were improved up to 59% performing the reaction at 40° C, without ligand according to procedure GP6. HPLC (Amylose column 5 cm, 3µm, l = 230 nm, n-Hex/iPrOH = 70:30, 1 mL/min): tR = 9.6 min, 20.6 min.

## ($S$)-*tert*-butyl 2-(((3-chlorobenzoyl)oxy)methyl)morpholine-4-carboxylate (3ba)

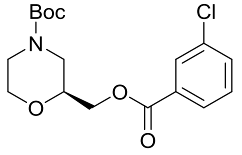


Compound **3ba** was prepared according to the general procedure (GP7) and isolated as colourless oil (yield 68%) after flash chromatography (n-Hex/AcOEt 1:1).

$R_f$ = 0.36 (n-Hex/EtOAc 2:1); stained with $KMnO_4$

$^1$H NMR (300 MHz, $CDCl_3$) δ 8.09 – 8.00 (m, 1H), 7.95 (d, J = 7.8 Hz, 1H), 7.55 (d, J = 8.0 Hz, 1H), 7.40 (t, J = 7.9 Hz, 1H), 4.46 – 4.22 (m, 2H), 4.17 – 3.69 (m, 4H), 3.58 (td, J = 11.7, 2.6 Hz, 1H), 3.13 – 2.67 (m, 2H), 1.49 (s, J = 7.7 Hz, 9H).

$^{13}$C NMR (75 MHz, $CDCl_3$) δ 165.5 (s), 155.1 (s), 135.0 (s), 133.8 (s), 133.6 (d), 131.9 (s), 130.2 (d), 128.3 (d), 80.8 (s), 73.6 (d), 66.9 (t), 65.63 (2 signals – (d) + (t)), 46.1 (t – overlapping), 43.5 (t – overlapping), 28.8 (q).

MS (ESI): m/z 378.19 [M+Na]$^+$

$[\alpha]^D{}_{20}$: -18° (c: 0.001 in $CHCl_3$)

From GP7. *Reaction conditions*: $Pd(OAc)_2$ 10 mol%, **L4** 12 mol% in xylene/DCM 2:1 (0.3 M) T = 0° C. *er* 85:15. HPLC (Chiralcel OD-H, 3 µm, l = 230 nm, n-Hex/iPrOH = 90:10, 1 mL/min): tR = 6.2 min (major), 7.2 min (minor).

## *(S)*-(5,5-dimethyl-4-tosylmorpholin-2-yl)methyl 3-chlorobenzoate (3ca)

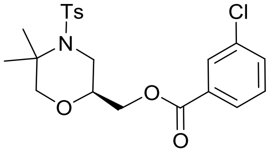


Compound **3ca** was prepared according to the general procedure (GP7) and isolated as colourless oil (yield 79%) after flash chromatography (n-Hex/AcOEt 4:1).

$R_f$ = 0.34 (n-Hex/EtOAc 2:1); stained with $KMnO_4$

$^1$H NMR (300 MHz, $CDCl_3$) δ 8.02 – 7.99 (m, 1H), 7.95 – 7.88 (m, 1H), 7.70 (d, $J$ = 8.3 Hz, 2H), 7.59 – 7.53 (m, 1H), 7.40 (t, $J$ = 7.8 Hz, 1H), 7.30 (d, $J$ = 7.9 Hz, 2H), 4.39 (dd, $J$ = 5.0, 1.6 Hz, 2H), 3.96 – 3.78 (m, 2H), 3.39 (s, 2H), 3.14 (dd, $J$ = 12.6, 10.6 Hz, 1H), 2.42 (s, 3H), 1.34 (s, 3H), 1.20 (s, 3H).

$^{13}$C NMR (75 MHz, $CDCl_3$) δ 165.0 (s), 143.4 (s), 138.9 (s), 134.6 (s), 133.2 (d), 131.4 (s), 129.8 (d), 129.7 (d), 127.8 (d), 127.1 (d), 77.6 (s), 74.2 (d), 64.9 (t), 57.0 (t), 44.0 (t), 24.4 (q), 21.5 (q), 20.1 (q).

MS (ESI): m/z 460.48 [M+Na]$^+$, 897.08 [2M+Na]$^+$

$[\alpha]^D{}_{20}$: -11° (c: 0.001 in $CHCl_3$)

From GP7. *Reaction conditions*: $Pd(OAc)_2$ 10 mol%, **L4** 12 mol% in xylene/DCM 2:1 (0.3 M) T = 0° C. *er* 94:6. HPLC (Amylose column 20cm, 3µm, l = 230 nm, n-Hex/iPrOH = 70:30, 1 mL/min): tR = 20.4 min (major), 23.8 min (minor).

## (4-nosylmorpholin-2-yl)methyl 3-chlorobenzoate (3da)

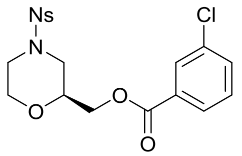


Compound **3da** was prepared according to the general procedure (GP7) and isolated as pale-yellow solid (yield 81%) after flash chromatography (n-Hex:AcOEt 4:1).

$R_f$ = 0.25 (n-Hex/EtOAc 2:1); stained with $KMnO_4$

M.p.: 74-76°C

$^1$H NMR (400 MHz, $CDCl_3$) δ 8.41 (d, *J* = 9.0 Hz, 2H), 8.01 – 7.92 (m,3), 7.92 – 7.84 (m, 1H), 7.55 (ddd, *J* = 8.0, 2.1, 1.1 Hz, 1H), 7.39 (t, *J* = 7.9 Hz, 1H), 4.33 (dd, *J* = 5.1, 1.8 Hz, 2H), 4.09 – 3.85 (m, 2H), 3.82 – 3.55 (m, 3H), 2.54 (td, *J* = 11.4, 3.4 Hz, 1H), 2.36 (dd, *J* = 11.2, 10.4 Hz, 1H).

$^{13}$C NMR (101 MHz, $CDCl_3$) δ 164.9 (s), 150.5 (s), 141.4 (s), 134.6 (s), 133.4 (d), 131.2 (s), 129.8 (d), 129.7 (d), 128.9 (d), 127.8 (d), 124.5 (d), 73.1 (d), 65.9 (t), 64.7 (t), 47.4 (t), 45.3 (t), 29.6 (t).

MS (ESI): m/z 441.10 $[M+H]^+$

$[\alpha]^D_{20}$: -8° (c: 0.001 in $CHCl_3$)

From GP7. *Reaction conditions*: $Pd(OAc)_2$ 10 mol%, **L4** 12 mol% in xylene/DCM 2:1 (0.3 M) T = 0° C. *er* 89:11. HPLC (Amylose column 20cm, 5µm, l = 230 nm, n-Hex/iPrOH = 70:30, 1 mL/min): tR = 16.4 min (major), 22.3 min (minor).

## (*S*)-(tetrahydro-2H-pyran-2-yl)methyl 3-chlorobenzoate (3ea)

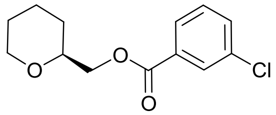


Compound **3ea** was prepared according to the general procedure (GP7) and isolated as a colourless oil (yield 48%) after flash chromatography (n-Hex → n-Hex:$Et_2O$ 1:2).

$R_f$ = 0.63 (n-Hex/$Et_2O$ 1:2); stained with Molybdenum

$^1$H NMR (300 MHz, $CDCl_3$) δ 8.06 (s, *J* = 1.8 Hz, 1H), 7.97 (d, *J* = 7.7 Hz, 1H), 7.61 – 7.50 (m, 1H), 7.40 (t, *J* = 7.9 Hz, 1H), 4.32 (dd, *J* = 5.1, 2.3 Hz, 2H), 4.06 (dd, *J* = 13.3, 2.2 Hz, 1H), 3.79 – 3.59 (m, 1H), 3.55 – 3.44 (m, 1H), 2.00 – 1.80 (m, 2H), 1.78 – 1.38 (m, 4H).

$^{13}$C NMR (75 MHz, $CDCl_3$) δ 165.4 (s), 134.5 (s), 133.0 (d), 131.9 (s), 129.8 (d), 129.7 (d), 127.9 (d), 75.4 (d), 68.5 (t), 68.2 (t), 28.0 (t), 25.8 (t), 23.0 (t).

MS (ESI): m/z 254.23 $[M+H]^+$

$[\alpha]^D_{20}$: +11° (c: 0.001 in $CHCl_3$)

From GP7. *Reaction conditions*: $Pd(OAc)_2$ 10 mol%, **L4** 12 mol% in xylene/DCM 2:1 (0.3 M) T = 0° C. *er* 93:7. HPLC (Amylose column 20cm, 3µm, l = 230 nm, n-Hex/iPrOH = 70:30, 1 mL/min): tR = 5.1 min (major), 6.0 min (minor).

## *(S)*-(5,5-dimethylltetrahydro-2H-pyran-2-yl)methyl 3-chlorobenzoate (3fa)

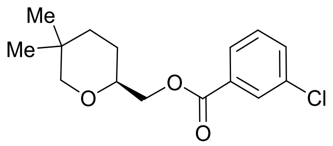

Compound **3fa** was prepared according to the general procedure (GP7) and isolated as a colourless oil (yield 54%) after flash chromatography (n-Hex → n-Hex:$Et_2O$ 1:2).

$R_f$ = 0.61 (n-Hex/$Et_2O$ 1:2); stained with Molybdenum

IR = 2938, 1722, 1251, 746 $cm^{-1}$

$^1$H NMR (300 MHz, $CDCl_3$) δ 8.06 (t, $J$ = 1.7 Hz, 1H), 8.00 – 7.91 (m, 1H), 7.55 (ddd, $J$ = 8.0, 2.1, 1.1 Hz, 1H), 7.40 (t, $J$ = 7.9 Hz, 1H), 4.35 (ddd, $J$ = 18.1, 11.6, 5.1 Hz, 2H), 3.71 – 3.44 (m, 2H), 3.23 (d, $J$ = 11.5 Hz, 1H), 1.72 – 1.38 (m, 4H), 1.05 (s, 3H), 0.88 (s, 3H).

$^{13}$C NMR (75 MHz, $CDCl_3$) δ 134.5 (s), 133.0 (d), 129.8 (d), 129.7 (d), 127.9 (d), 78.2 (t), 75.4 (d), 68.0 (t), 36.1 (t), 27.1 (q), 24.3 (t), 23.3 (q).

MS (ESI): m/z 283.84 $[M+H]^+$

$[\alpha]^D_{20}$: +8° (c: 0.001 in $CHCl_3$)

From GP7. *Reaction conditions*: $Pd(OAc)_2$ 10 mol%, **L4** 12 mol% in xylene/DCM 2:1 (0.3 M) T = 0° C. *er* 95:5. HPLC (Amylose column 20cm, 3μm, l = 230 nm, n-Hex/iPrOH = 90:10, 1 mL/min): tR = 9.3 min (major), 11.9 min (minor).

## *(S)*-(5,5-diphenyltetrahydro-2H-pyran-2-yl)methyl 3-chlorobenzoate (3ga)

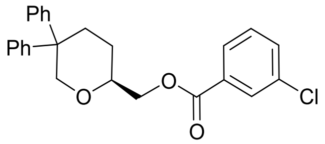

Compound **3ga** was prepared according to the general procedure (GP7) and isolated as a colourless oil (yield 62%) after flash chromatography (n-Hex → n-Hex:$Et_2O$ 1:2).

$R_f$ = 0.55 (n-Hex/$Et_2O$ 1:2); stained with Molybdenum

IR = 2950, 1721, 1253, 1097, 747 $cm^{-1}$

$^1$H NMR (300 MHz, $CDCl_3$) δ 7.98 (t, $J$ = 1.7 Hz, 1H), 7.93 – 7.81 (m, 1H), 7.55 (ddd, $J$ = 8.0, 2.1, 1.1 Hz, 1H), 7.51 – 7.12 (m, 11H), 4.73 (dd, $J$ = 12.1, 2.6 Hz, 1H), 4.41 (dd, $J$ = 11.7, 3.2 Hz, 1H), 4.30 (dd, $J$ = 11.7, 5.3 Hz, 1H), 3.94 – 3.76 (m, 1H), 3.63 (d, $J$ = 12.1 Hz, 1H), 2.69 – 2.39 (m, 2H), 1.70 – 1.47 (m, 2H).

$^{13}$C NMR (75 MHz, $CDCl_3$) δ 165.4 (s), 146.2 (s), 145.3 (s), 134.5 (s), 133.1 (d), 131.8 (s), 129.7 (d), 129.7 (d), 128.9 (d), 128.4 (d), 128.1 (d), 128.0 (d), 127.0 (d), 126.5 (d), 125.9 (d), 75.3 (d), 74.9 (t), 67.6 (t), 45.8 (s), 34.2 (d), 24.0 (d).

MS (ESI): 429.38 m/z $[M+H]^+$

$[\alpha]^D_{20}$: +91° (c: 0.001 in $CHCl_3$)

From GP7. *Reaction conditions*: $Pd(OAc)_2$ 10 mol%, **L4** 12 mol% in xylene/DCM 2:1 (0.3 M) T = 0° C. *er* 97:3 HPLC (OD-H column 20cm, 5µm, l = 230 nm, n-Hex/iPrOH = 95:5, 1 mL/min): tR = 9.3 min (major), 11.9 min (minor).

## (*S*)-(5,5-dipropyltetrahydro-2H-pyran-2-yl)methyl 3-chlorobenzoate (3ha)

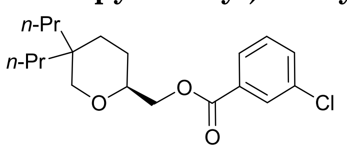


Compound **3ha** was prepared according to the general procedure (GP7) and isolated as a colourless oil (yield 56%) after flash chromatography (n-Hex → n-Hex:$Et_2O$ 1:2).

$R_f$ = 0.69 (n-Hex:$Et_2O$ 1:2); stained with Molybdenum

IR = 2955, 1724, 1252, 1073, 747 $cm^{-1}$

$^1H$ NMR (400 MHz, $CDCl_3$) δ 8.06 (t, *J* = 1.8 Hz, 1H), 7.97 (dt, *J* = 7.8, 1.3 Hz, 1H), 7.55 (ddd, *J* = 8.0, 2.1, 1.1 Hz, 1H), 7.40 (t, *J* = 7.9 Hz, 1H), 4.38 (dd, *J* = 11.6, 3.5 Hz, 1H), 4.30 (dd, *J* = 11.6, 6.4 Hz, 1H), 3.71 (dd, *J* = 11.3, 2.6 Hz, 1H), 3.67 – 3.58 (m, 1H), 3.17 (d, *J* = 11.3 Hz, 1H), 1.76 – 1.03 (m, 12H), 0.92 (dt, *J* = 19.0, 7.2 Hz, 6H).

$^{13}C$ NMR (101 MHz, $CDCl_3$) δ 165.4 (s), 134.5 (s), 133.0 (d), 132.0 (s), 129.8 (d), 129.6 (d), 127.9 (d), 75.9 (t), 75.5 (d), 68.0 (t), 39.4 (t), 34.7 (s), 33.8 (t), 32.8 (t), 23.7 (t), 16.3 (t), 15.8 (t), 15.0 (q), 15.0 (q).

MS (ESI): m/z 339.31 $[M+H]^+$, 361.37 $[M+Na]^+$

$[\alpha]^D_{20}$: -18° (c: 0.001 in $CHCl_3$)

From GP7. *Reaction conditions*: $Pd(OAc)_2$ 10 mol%, **L4** 12 mol% in xylene/DCM 2:1 (0.3 M) T = 0° C. *er* 95:5. HPLC (amylose column 20cm, 3µm, l = 230 nm, n-Hex/iPrOH = 95:5, 1 mL/min): tR = 5.5 min (major), tR = 7.2 min (major).

## (*S*)-(2-oxaspiro[5.5]undecan-3-yl)methyl 3-chlorobenzoate (3ia)

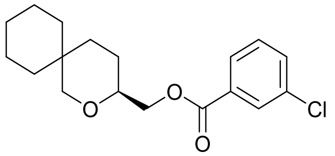


Compound **3ia** was prepared according to the general procedure (GP7) and isolated as a colourless oil (yield 43%) after flash chromatography (n-Hex → n-Hex/$Et_2O$ 2:1).

$R_f$ = 0.50 (-Hex/$Et_2O$ 2:1); stained with Molybdenum

$^1H$ NMR (300 MHz, $CDCl_3$) δ 8.00 (dt, *J* = 19.0, 6.5 Hz, 2H), 7.66 – 7.49 (m, 1H), 7.40 (t, *J* = 7.9 Hz, 1H),4.43 – 4.20 (m, 2H), 4.19 – 3.94 (m, 1H), 3.69 – 3.43 (m, 1H), 3.20 (t, *J* = 11.1 Hz, 1H), 2.32 – 0.68 (m, 14H).

$^{13}C$ NMR (75 MHz, $CDCl_3$) δ 165.8 (s), 151.7 (s), 133.4 (d), 132.3 (s), 130.2 (d), 130.0 (d), 128.3 (d), 75.8 (d), 72.3 (t), 68.5 (t), 31.7 (s), 30.8 (t), 30.4 (t), 28.6 (t), 27.3 (t), 27.0 (t), 24.6 (t), 24.3 (t).

$[\alpha]^D_{20}$: +8° (c: 0.001 in $CHCl_3$)

From GP7. *Reaction conditions*: $Pd(OAc)_2$ 10 mol%, **L4** 12 mol% in xylene/DCM 2:1 (0.3 M) T = 0° C. *er* 92:8. HPLC (OD-H column 20cm, 5µm, l = 230 nm, n-Hex/iPrOH = 95:5, 1 mL/min): tR = 4.3 min (major), tR = 4.8 min (minor).

### ((*2S,5S*)-5-(m-tolyl)tetrahydro-2H-pyran-2-yl)methyl 3-chlorobenzoate (*trans*-3ja)

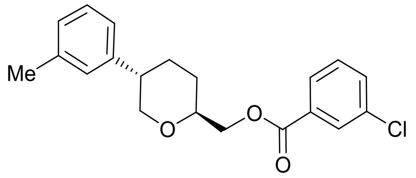


Compound ***trans*-3ja** was prepared according to the general procedure (GP7) and isolated as a colourless oil (yield 43%) after flash chromatography (n-Hex → n-Hex:$Et_2O$ 1:2).

$R_f$ = 0.58 (n-Hex:$Et_2O$ 1:2); stained with Molybdenum

IR = 2926, 1722, 1252, 747 cm-1

$^1$H NMR (300 MHz, $CDCl^3$) δ 8.08 (t, *J* = 1.9 Hz, 1H), 8.00 (dt, *J* = 7.7, 1.1 Hz, 1H), 7.56 (ddd, *J* = 8.0, 2.0, 1.0 Hz, 1H), 7.42 (t, *J* = 7.9 Hz, 1H), 7.27 – 7.17 (m, 1H), 7.12 – 6.99 (m, 3H), 4.40 (dd, *J* = 5.0, 2.8 Hz, 2H), 4.10 (ddd, *J* = 11.2, 4.3, 2.1 Hz, 1H), 3.87 – 3.74 (m, 1H), 3.50 (t, *J* = 11.3 Hz, 1H), 2.86 (tt, *J* = 11.6, 4.0 Hz, 1H), 2.37 (s, *J* = 3.5 Hz, 3H), 2.23 – 2.04 (m, 1H), 1.92 – 1.80 (m, 2H), 1.74 – 1.56 (m, 1H).

$^{13}$C NMR (75 MHz, $CDCl_3$) δ 141.9 (s), 138.2 (s), 134.5 (s), 133.1 (d), 131.9 (s), 129.8 (d), 129.7 (d), 128.5 (d), 128.2 (d), 127.9 (d), 127.6 (d), 124.3 (d), 75.2 (d), 73.7 (t), 68.0 (t), 42.5 (d), 30.1 (t), 28.1 (t), 21.5 (q).

MS (ESI): 367.65 m/z $[M+Na]^+$

$[\alpha]^D_{20}$: +7° (c: 0.001 in $CHCl_3$)

From GP7. *Reaction conditions*: $Pd(OAc)_2$ 10 mol%, **L4** 12 mol% in xylene/DCM 2:1 (0.3 M) T = 0° C. *er* 10:90. HPLC (OD-H column 20cm, 5µm, l = 230 nm, n-Hex/iPrOH = 95:5, 1 mL/min): tR = 6.7 min (minor), tR = 8.6 min (major).

### ((*2S,5R*)-5-(m-tolyl)tetrahydro-2H-pyran-2-yl)methyl 3-chlorobenzoate (*cis*-3ja)

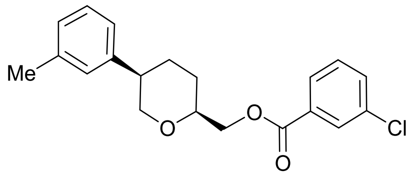


Compound ***cis*-3ja** (yield 11%) was prepared according to the general procedure (GP7) and isolated in mixture with ***trans*-3ia** as a colourless oil after flash chromatography (n-Hex → n-Hex:$Et_2O$ 1:2).

$R_f$ = 0.52 (n-Hex/$Et_2O$ 1:2); stained with Molybdenum

IR = 2924, 1724, 1252, 747 $cm^{-1}$

$^1$H NMR (300 MHz, $CDCl_3$) δ 8.08 (dt, *J* = 5.0, 1.7 Hz, 1H), 7.99 (t, *J* = 7.2 Hz, 1H), 7.56 (d, *J* = 8.0 Hz, 1H), 7.45 – 7.32 (m, 2H), 7.23 (t, *J* = 7.4 Hz, 1H), 7.05 (t, *J* = 7.6 Hz, 2H), 4.50 – 4.40 (m, 2H), 4.36 – 4.27 (m, 1H), 3.99 – 3.87 (m, 1H), 3.50 (t, *J* = 11.3 Hz, 1H), 2.93 – 2.83 (m, 1H), 2.35 (s, 3H), 2.19 – 1.95 (m, 2H), 1.71 – 1.52 (m, 2H).

$^{13}C$ NMR (75 MHz, $CDCl_3$) δ 165.4 (s), 143.7 (s), 137.8 (s), 134.5 (s), 133.1 (d), 131.8 (s), 129.8 (d), 129.7 (d), 129.0 (d), 128.2 (d), 127.9 (d), 126.9 (d), 125.3 (d), 74.5 (d), 70.1 (t), 67.3 (t), 38.5 (d), 29.1 (t), 23.3 (t), 21.6 (q).

MS (ESI): 367.41 m/z $[M+Na]^+$

$[\alpha]^D{}_{20}$: +20° (c: 0.001 in $CHCl_3$)

From GP7. *Reaction conditions*: $Pd(OAc)_2$ 10 mol%, **L4** 12 mol% in xylene/DCM 2:1 (0.3 M) T = 0° C. *er* 96:3. HPLC (OD-H column 20cm, 5µm, l = 230 nm, n-Hex/iPrOH = 95:5, 1 mL/min): tR = 5.7 min (major), tR = 6.4 min (minor).

## ((*2S,5S*)-5-(benzo[d][1,3]dioxol-5-yl)tetrahydro-2H-pyran-2-yl)methyl 3-chlorobenzoate (3ka)

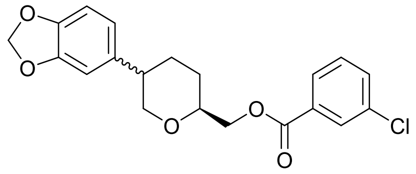


Compound **3ka** was prepared according to the general procedure (GP7) and isolated as a *trans/cis* mixture (ratio 2.5:1) colourless gum (yield 57%) after flash chromatography (n-Hex → n-Hex/$Et_2O$ 2:1).

$R_f$ = 0.36 (n-Hex/$Et_2O$ 2:1); stained with Molybdenum

IR = 2923, 1722, 1487, 1252, 1038, 748 $cm^{-1}$

The $^1H$ NMR signals of the major and minor diastereoisomers, where not otherwise stated, collapse together. The unit of the integral has been attributed, considering the two cis/trans-diastereoisomer, regardless of the predominance of the *trans*-isomer.

$^1H$ NMR (400 MHz, $CDCl_3$) δ 8.12 – 8.04 (m, 2H), 8.03 – 7.94 (m, 2H), 7.64 – 7.53 (m, 2H), 7.48 – 7.36 (m, 2H), 6.78 (dd, $J$ = 8.0, 2.1 Hz, 2H), 6.74 – 6.64 (m, 4H), 5.95 (s, $J$ = 0.8 Hz, 4H), 4.46 – 4.33 (m, 4H), 4.31 – 4.21 (m, 1H, signal of the *cis*-diastereoisomer), 4.06 (ddd, $J$ = 11.2, 4.3, 2.1 Hz, 1H, signal of the *trans*-diastereoisomer), 3.93 – 3.84 (m, 2H, signal of the *cis*-diastereoisomer), 3.83 – 3.73 (m, 1H, signal of the *trans*-diastereoisomer), 3.42 (t, $J$ = 11.3 Hz, 1H, signal of the *trans*-diastereoisomer), 2.87 – 2.75 (m, 2H), 2.19 – 1.92 (m, 4H), 1.91 – 1.49 (m, 4H).

*trans*-diastereoisomer: $^{13}C$ NMR (75 MHz, $CDCl_3$) δ 165.8 (s), 148.1 (s), 138.1 (s), 136.2 (s), 134.9 (s), 133.4 (d), 132.3 (s), 130.2 (d), 130.2 (d), 128.3 (d), 120.6 (d), 108.8 (d), 108.1 (d), 101.3 (t), 75.6 (d), 74.3 (t), 68.3 (t), 42.6 (d), 30.6 (t), 28.5 (t).

*cis*-diastereoisomer: $^{13}C$ NMR (75 MHz, $CDCl_3$) δ 169.0 (s), 146.7 (s), 139.7 (s), 135.1 (s), 134.0 (d), 130.6 (d), 128.7 (d), 121.2 (d), 109.4 (d), 108.4 (d), 101.2 (t), 74.9 (d), 70.7 (t), 67.8 (t), 38.8 (d), 29.8 (t), 23.6 (t).

MS (ESI): m/z 375.22 $[M+H]^+$, 770.87 $[2M+Na]^+$

$[\alpha]^D{}_{20}$: -8° (c: 0.001 in $CHCl_3$)

From GP7. *Reaction conditions*: $Pd(OAc)_2$ 10 mol%, **L4** 12 mol% in xylene/DCM 2:1 (0.3 M) T = 0° C. *trans/cis* ratio 2.5:1; *trans er* 91:9; *cis er* 2:98. HPLC (OD-H column 20cm, 5µm, l = 230 nm, n-Hex/iPrOH = 95:5, 1 mL/min): ***trans-3ka*** tR= 11.0 min (minor), tR = 12.4 min (major); ***cis-3ka*** tR= 9.2 min (major), tR = 9.8 min (minor).

## ((2S,5S)-5-(4-methoxyphenyl)tetrahydro-2H-pyran-2-yl)methyl 3-chlorobenzoate (3la)

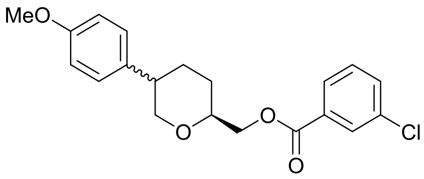

Compound **3la** was prepared according to the general procedure (GP7) and isolated as a *trans/cis* mixture (ratio 3:1) (yields 58%) after flash chromatography (n-Hex → n-Hex/$Et_2O$ 2:1).

$R_f$ = 0.35 (n-Hex/$Et_2O$ 2:1); stained with Molybdenum

IR = 2924, 1722, 1513, 1280, 1247, 1084, 747 $cm^{-1}$

The $^1H$ NMR signals of the major and minor diastereoisomers, where not otherwise stated, collapse together. The unit of the integral has been attributed, considering the two cis/trans-diastereoisomer, regardless of the predominance of the *trans*-isomer.

$^1H$ NMR (300 MHz, $CDCl_3$) δ 8.20 – 7.88 (m, 4H), 7.66 – 7.50 (m, 2H), 7.49 – 7.33 (m, 2H), 7.15 (d, $J$ = 8.4 Hz, 4H), 6.88 (d, $J$ = 8.5 Hz, 4H), 4.48 – 4.33 (m, 4H), 4.32 – 4.15 (m, 1H, signal of the *cis*-diastereoisomer), 4.15 – 3.96 (m, 1H, signal of the *trans*-diastereoisomer), 3.96 – 3.69 (m, 8H, including H of the -OMe and 2H of 6-membered cycle of the *cis*-diastereoisomer), 3.44 (t, $J$ = 11.3 Hz, 2H), 2.96 – 2.70 (m, 4H), 2.24 – 1.94 (m, 2H), 1.95 – 1.43 (m, 6H)

*trans*-diastereoisomer: $^{13}C$ NMR (75 MHz, $CDCl_3$) δ 165.8 (s), 158.8 (s), 135.0 (s), 134.9 (s), 133.4 (d), 132.3 (s), 130.2 (d), 130.1 (d), 128.6 (d), 128.3 (d), 114.4 (d), 75.6 (d), 74.3 (t), 68.4 (t), 55.7 (q), 42.0 (d), 30.6 (t), 28.5 (t).

*cis*-diastereoisomer: $^{13}C$ NMR (75 MHz, $CDCl_3$) δ 165.8 (s), 158.8 (s), 135.0 (s), 134.9 (s), 134.0 (d), 131.3 (d), 130.6 (d), 128.6 (d), 128.3 (d), 114.0 (d), 74.9 (d), 70.7 (t), 67.7 (t), 55.7 (q), 38.2 (d), 29.7 (t), 23.6 (t).

MS (ESI): m/z 383.40 $[M+Na]^+$

$[\alpha]^D_{20}$: +8° (c: 0.001 in $CHCl_3$)

From GP7. *Reaction conditions*: $Pd(OAc)_2$ 10 mol%, **L4** 12 mol% in xylene/DCM 2:1 (0.3 M) T = 0° C. *trans/cis* ratio 3:1; *trans er* 93:7; *cis er* 98:2. HPLC (OD-H column 20cm, 5µm, l = 230 nm, n-Hex/iPrOH = 95:5, 1 mL/min): ***trans-3la*** 8.1 min (minor), tR = 9.5 min (major); ***cis-3la*** tR= 7.0 min (major), tR = 7.4 min (minor).

## ((*2S,6R*)-6-(4-methoxyphenyl)tetrahydro-2H-pyran-2-yl)methyl 3-chlorobenzoate (3ma)

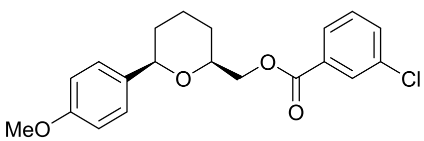

Compound **3ma** was prepared according to the general procedure (GP7) and isolated only as *cis*-diastereoisomer and colourless oil (yield 39%) after flash chromatography (n-Hex → n-Hex/$Et_2O$ 2:1).

$R_f$ = 0.40 (n-Hex/$Et_2O$ 2:1); stained with Molybdenum

$^{1}H$ NMR (300 MHz, $CD_3CN$) δ 8.05 – 7.91 (m, 2H), 7.65 (ddd, *J* = 8.1, 2.2, 1.1 Hz, 1H), 7.51 (t, *J* = 7.9 Hz, 1H), 7.33 – 7.24 (m, 2H), 6.93 – 6.84 (m, 2H), 4.45 – 4.38 (m, 1H), 4.38 – 4.33 (m, 2H), 3.97 – 3.85 (m, 1H), 3.78 (s, J = 2.3 Hz, 3H), 3.66 – 3.57 (m, 8H), 1.99 – 1.93 (m, 1H), 1.87 – 1.65 (m, 3H), 1.61 – 1.35 (m, 2H).

$^{13}C$ NMR (75 MHz, $CDCl_3$) δ 171.3 (s), 165.6 (s), 159.3 (s), 136.0 (s), 133.4 (d), 131.9 (d), 130.8 (s), 130.5 (d), 129.5 (d), 128.1 (d), 127.6 (d), 113.9 (d), 79.4 (d), 76.1 (d), 69.7 (t), 55.2 (q), 33.5 (t), 27.5 (t), 23.6 (t).

MS (ESI): m/z 383.25 $[M+Na]^+$

$[\alpha]^{D}_{20}$: +18° (c: 0.001 in $CHCl_3$)

From GP7. *Reaction conditions*: $Pd(OAc)_2$ 10 mol%, **L4** 12 mol% in xylene/DCM 2:1 (0.3 M) T = 0° C. *er* 95:5. HPLC (OD-H column 20cm, 5μm, l = 230 nm, n-Hex/iPrOH = 95:5, 1 mL/min): tR = 7.0 min (minor), tR = 7.7 min (major).

## ((*2S,6R)*-6-((*E*)-2-methoxystyryl)tetrahydro-2H-pyran-2-yl)methyl 3-chlorobenzoate (1n)

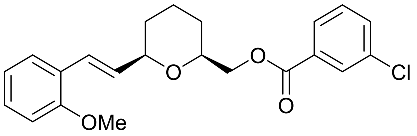

Compound **1n** was prepared according to the general procedure (GP7) and isolated only as *cis*-diastereoisomer and colourless gum (yield 34%) after flash chromatography (n-Hex → n-Hex/$Et_2O$ 2:1).

$R_f$ = 0.43 (n-Hex/$Et_2O$ 2:1); stained with Molybdenum

IR = 2931, 1723, 1289, 1244, 1072, 746 $cm^{-1}$

$^{1}H$ NMR (300 MHz, $CDCl_3$) δ 8.12 – 8.02 (m, 1H), 8.02 – 7.94 (m, 1H), 7.62 – 7.31 (m, 3H), 7.23 – 7.13 (m, 1H), 7.00 – 6.78 (m, 3H), 6.26 (dd, *J* = 16.2, 6.2 Hz, 1H), 4.42 – 4.28 (m, 2H), 4.08 (dd, *J* = 10.8, 6.3 Hz, 1H), 3.86 – 3.75 (m, 4H), 2.13 (dd, *J* = 14.6, 7.1 Hz, 1H), 2.06 – 1.86 (m, 1H), 1.85 – 1.36 (m, 4H).

$^{13}C$ NMR (75 MHz, $CDCl_3$) δ 168.9 (s), 167.8 (s), 134.7 (s), 132.9 (d), 132.5 (s), 131.0 (d), 129.8 (d), 129.6 (d), 128.5 (d), 127.8 (d), 126.9 (d), 125.1 (d), 120.6 (d), 110.8 (d), 78.8 (d), 75.4 (d), 68.2 (t), 55.4 (q), 31.6 (t), 27.7 (t), 23.1 (t).

MS (ESI): m/z 409.49 $[M+Na]^+$

$[\alpha]^{D}_{20}$: +14° (c: 0.001 in $CHCl_3$)

From GP7. *Reaction conditions*: $Pd(OAc)_2$ 10 mol%, **L4** 12 mol% in xylene/DCM 2:1 (0.3 M) T = 0° C. *er* 95:5. HPLC (OD-H column 20cm, 5μm, l = 230 nm, n-Hex/iPrOH = 95:5, 1 mL/min): tR = 12.3 min (major), tR = 17.3 min (minor).

## ((*2R,5S*)-5-benzyl-4-tosylmorpholin-2-yl)methyl 3-chlorobenzoate (3oa)

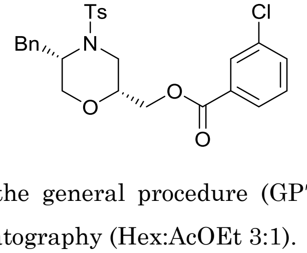

Compound **3oa** was prepared according to the general procedure (GP7) and isolated as a colourless oil and only diastereoisomer (yield 79%) after flash chromatography (Hex:AcOEt 3:1).

$R_f$ = 0.31 (n-Hex/EtOAc 2:1); stained with $KMnO_4$

IR = 2922, 1724, 1253, 1158, 745, 676 $cm^{-1}$

$^1$H NMR (300 MHz, $CDCl_3$) δ 8.08 – 8.00 (m, 1H), 7.96 (d, $J$ = 7.8 Hz, 1H), 7.71 – 7.51 (m, 3H), 7.43 (t, $J$ = 7.9 Hz, 1H), 7.35 – 7.16 (m, 7H), 4.39 (d, $J$ = 4.8 Hz, 2H), 4.14 – 3.99 (m, 1H), 3.88 – 3.62 (m, 3H), 3.52 (dd, $J$ = 11.7, 2.4 Hz, 1H), 3.26 – 2.99 (m, 2H), 2.79 (dd, $J$ = 13.2, 5.1 Hz, 1H), 2.43 (s, 3H).

$^{13}$C NMR (75 MHz, $CDCl_3$) δ 165.0 (s), 143.6 (s), 137.6 (s), 137.3 (s), 134.7 (s), 133.3 (d), 131.4 (s), 129.9 (d), 129.8 (d), 129.8 (d), 129.4 (d), 128.7 (d), 127.8 (d), 127.2 (d), 126.7 (d), 73.1 (d), 67.4 (t), 65.1 (t), 54.1 (d), 42.2 (t), 34.2 (t), 21.5 (q).

MS (ESI): m/z 500.32 [M+H]

From GP4. HPLC (Cellulose column 20cm, 5µm, l = 210 nm, n-Hex/iPrOH = 90:10, 0.5 mL/min): tR = 33.8 min.

$[\alpha]^D{}_{20}$: -12° (c: 0.001 in $CHCl_3$)

## ((*2S,5R)*-5-phenyl-4-tosylmorpholin-2-yl)methyl 3-chlorobenzoate (3pa)

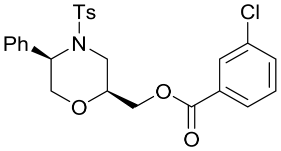

Compound **3pa** was prepared according to the general procedure (GP7) and and isolated as a colourless oil and only diastereoisomer (yield 81%) after flash chromatography (Hex:AcOEt 4:1).

$R_f$ = 0.34 (n-Hex/EtOAc 2:1); stained with $KMnO_4$

$^1$H NMR (300 MHz, $CDCl_3$) δ 8.02 – 7.89 (m, 1H), 7.89 – 7.80 (m, 1H), 7.73 – 7.46 (m, 4H), 7.43 – 7.21 (m, 5H), 5.00 (d, $J$ = 3.0 Hz, 1H), 4.46 – 4.27 (m, 3H), 3.95 – 3.61 (m, 3H), 3.15 (dd, $J$ = 13.7, 11.4 Hz, 1H), 2.42 (s, 3H).

$^{13}$C NMR (75 MHz, $CDCl_3$) δ 165.3 (s), 144.0 (s), 137.8 (s), 137.6 (s), 135.0 (s), 133.7 (d), 131.7 (s), 130.2 (d), 130.1 (d), 128.9 (d), 128.8 (d), 128.3 (d), 128.2 (d), 127.6 (d), 72.9 (d), 69.2 (t), 65.4 (t), 54.8 (d), 42.7 (t), 21.9 (q).

MS (ESI): m/z 485.92 $[M+H]^+$, 508.12 $[M+Na]^+$

$[\alpha]^D{}_{20}$: -40° (c: 0.001 in $CHCl_3$)

## (*2R,5S*)-5-ethyl-4-tosylmorpholin-2-yl)methyl 3-chlorobenzoate (3qa)

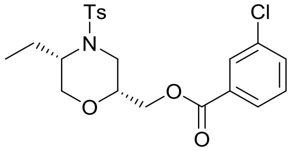

Compound **3qa** was prepared according to the general procedure (GP6) and isolated as a colourless oil and only diastereoisomer (yield 67%) after flash chromatography (Hex:AcOEt 3:1).

$R_f$ = 0.31 (n-Hex/EtOAc 2:1); stained with $KMnO_4$

IR = 2966, 1726, 1255, 1161, 748, 677 $cm^{-1}$

$^1$H NMR (300 MHz, $CDCl_3$) δ 8.00 (s, 1H), 7.92 (d, *J* = 7.8 Hz, 1H), 7.73 (d, *J* = 8.2 Hz, 2H), 7.57 (d, *J* = 7.9 Hz, 1H), 7.42 (t, *J* = 7.8 Hz, 1H), 7.33 (d, *J* = 8.0 Hz, 2H), 4.29 (d, *J* = 4.9 Hz, 2H), 3.86 – 3.48 (m, 5H), 3.07 (dd, *J* = 13.4, 11.2 Hz, 1H), 2.45 (s, 3H), 1.77 – 1.54 (m, 2H), 0.91 (t, *J* = 7.4 Hz, 3H).

$^{13}$C NMR (75 MHz, $CDCl_3$) δ 165.0 (s), 143.6 (s), 137.9 (s), 134.6 (s) 133.3 (d), 129.9 (s), 129.8 (d), 129.8 (d), 127.8 (d), 127.0 (d), 72.6 (d), 68.1 (t), 65.1 (t), 54.3 (d), 42.0 (t), 21.6 (q), 21.0 (t), 10.8 (q).

MS (ESI): m/z 438.32 $[M+H]^+$, 460.30 $[M+Na]^+$

$[\alpha]^D{}_{20}$: +13° (c: 0.001 in $CHCl_3$)

## (*2R,5S*)-5-isobutyl-4-tosylmorpholin-2-yl)methyl 3-chlorobenzoate (3ra)

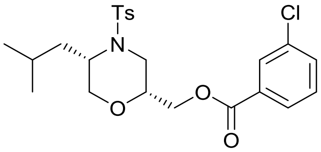

Compound **3ra** was prepared according to the general procedure (GP6) and isolated as a colourless oil and only diastereoisomer (yield 75%) after flash chromatography (Hex:AcOEt 4:1).

$R_f$ = 0.33 (n-Hex/EtOAc 2:1); stained with $KMnO_4$

IR = 2956, 1724, 1253, 1158, 1086, 748, 676 $cm^{-1}$

$^1$H NMR (300 MHz, $CDCl_3$) δ 8.00 (t, *J* = 1.7 Hz, 1H), 7.96 – 7.89 (m, 1H), 7.73 (d, *J* = 8.3 Hz, 2H), 7.57 (ddd, *J* = 8.0, 2.0, 1.0 Hz, 1H), 7.41 (t, *J* = 7.9 Hz, 1H), 7.33 (d, *J* = 8.1 Hz, 2H), 4.30 (dd, *J* = 4.6, 2.4 Hz, 2H), 3.98 – 3.87 (m, 1H), 3.80 – 3.53 (m, 4H), 3.10 (dd, *J* = 13.1, 10.8 Hz, 1H), 2.45 (s, *J* = 3.9 Hz, 3H), 1.64 – 1.35 (m, 2H), 0.95 – 0.87 (m, 6H).

$^{13}$C NMR (75 MHz, $CDCl_3$) δ 164.9 (s), 143.6 (s), 137.8 (s), 134.6 (s), 133.3 (d), 129.9 (d), 129.8 (d), 129.7 (d), 127.8 (d), 127.2 (d), 117.5 (s), 72.5 (d), 68.9 (t), 65.1 (t), 51.0 (d), 41.9 (t), 36.9 (t), 24.8 (q), 22.6 (q), 22.4 (q).

MS (ESI): m/z 466.21 [M+H]

$[\alpha]^D{}_{20}$: +21° (c: 0.001 in $CHCl_3$)

## ((*2R,5S*)-5-isopropyl-4-tosylmorpholin-2-yl)methyl 3-chlorobenzoate (3sa)

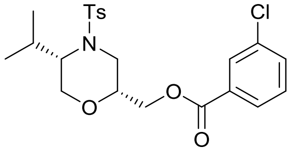

Compound **3sa** was prepared according to the general procedure (GP6) and isolated as a colourless oil and only diastereoisomer (yield 77%) after flash chromatography (Hex:AcOEt 4:1).

$R_f$ = 0.38 (n-Hex/EtOAc 2:1); stained with $KMnO_4$

$^1$H NMR (300 MHz, $CDCl_3$) δ 8.02 – 7.96 (m, 1H), 7.92 (d, *J* = 7.8 Hz, 1H), 7.74 (d, *J* = 8.2 Hz, 2H), 7.62 – 7.53 (m, 1H), 7.42 (t, *J* = 7.9 Hz, 1H), 7.33 (d, *J* = 8.3 Hz, 2H), 4.25 (d, *J* = 5.0 Hz, 2H), 3.95 (d, *J* = 11.7 Hz, 1H), 3.76 (dd, *J* = 14.6, 2.6

Hz, 1H), 3.55 – 3.28 (m, 3H), 3.10 (dd, $J$ = 14.5, 11.4 Hz, 1H), 2.45 (s, 3H), 2.27 (qd, $J$ = 13.4, 6.7 Hz, 1H), 0.98 (dd, $J$ = 11.8, 6.7 Hz, 6H).

$^{13}$C NMR (75 MHz, $CDCl_3$) δ 164.9 (s), 143.6 (s), 138.4 (s), 134.6 (s), 133.3 (d), 131.3 (s), 130.0 (d), 129.8 (d), 129.7 (d), 127.8 (d), 127.0 (d), 72.0 (d), 66.4 (t), 65.0 (t), 59.0 (d), 42.6 (t), 25.4 (d), 21.6 (q), 19.9 (q), 19.8 (q).

MS (ESI): m/z 474.46 $[M+Na]^+$, 925.04 $[2M+Na]^+$

$[\alpha]^D_{20}$: -13° (c: 0.001 in $CHCl_3$)

## Synthesis of derivative 4

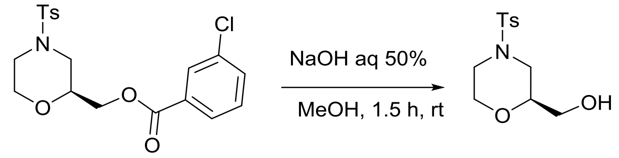


To a solution of the compound **S1** (40 mg, 0.1 mmol) in MeOH (2 mL, 0.05 M), an aqueous solution 50% m/m NaOH was added. The solution was stirred for 1.5 h at room temperature. Then, the organic solvent was evaporated and the aqueous residue was rinsed with EtOAc. The organic phase was extracted and washed with brine. The organic phase was dried over $NaSO_4$, filtered and the solvent evaporated *in vacuo*. The crude residue could be purified either by crystallization in EtOAc/n-Hex (yields 94%) or by flash chromatography on silica gel (n-Hex/EtOAc 2:1 → 1:1) affording the compound **4** (24 mg, 0.091 mmol, 91%) as a white gum.

## (*S*)-(4-tosylmorpholin-2-yl)methanol (4)

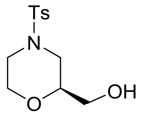


$R_f$ = 0.26 (n-Hex/EtOAc 1:1); stained with $KMnO_4$

IR: 3466, 2872, 1598, 1336, 1100, 981, 813, 753 $cm^{-1}$

$^1$H-NMR (300 MHz, $CDCl_3$) δ: 7.64 (d, $J$ = 8.2 Hz, 2H), 7.35 (d, $J$ = 8.1 Hz, 2H), 4.05 – 3.84 (m, 1H), 3.79 – 3.42 (m, 6H), 2.49 – 2.34 (m, 4H), 2.33 – 2.18 (m, 1H).

$^{13}$C-NMR (75 MHz, $CDCl_3$) δ: 1443.0 (s), 132.1 (s), 129.8 (d), 127.9 (d), 75.3 (d), 65.9 (t), 63.4 (t), 46.9 (t), 45.5 (t), 21.5 (q).

MS (ESI): 294.19 m/z $[M+Na]^+$

$[\alpha]^D_{20}$: -20° (c: 0.001 in $CHCl_3$)

89:11 *er* HPLC (Amylose column 20cm, 3µm, l = 230 nm, n-Hex/iPrOH = 70:30, 1 mL/min): tR = 17.4 min (major 25.8 min (minor).

## Synthesis of derivative 5

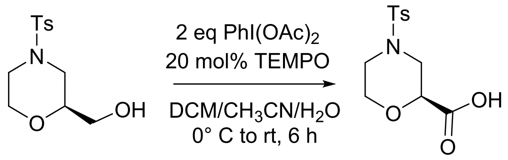


To a solution of the compound **S2** (20 mg, 0.074 mmol) in a mixture of DCM/$CH_3CN$/$H_2O$ 2:2:1 (3 mL, 0.025 M), PhI$(OAc)_2$ (48 mg, 0.148 mmol) was added at 0° C, followed by the addition of TEMPO (2.3 mg, 0.0148 mmol, 20 mol%). The solution was stirred for 6 h at room temperature. Then, the organic solvent was evaporated and the aqueous residue was rinsed with EtOAc (10 mL). An aqueous solution of NaOH (2M) was added until pH = 9, and the aqueous phase was extracted. To the aqueous phase, EtOAc (20 mL) was added, followed by the addition of 2M HCl until pH = 2. The organic phase was extracted and washed with brine. Then the organic phase was dried over $NaSO_4$, filtered and the solvent evaporated *in vacuo*, affording the compound **7** (16 mg, 0.059 mmol, 82%) as a white gum.

## (*S*)-4-tosylmorpholine-2-carboxylic acid (5)

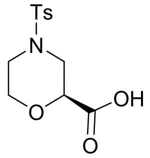


$R_f$ = 0.11 (n-Hex/EtOAc 1:1); flame-like

$^1$H NMR (300 MHz, CDCl*3*) δ 7.65 (d, *J* = 8.3 Hz, 2H), 7.36 (d, *J* = 8.0 Hz, 2H), 4.27 (dd, *J* = 9.8, 3.1 Hz, 1H), 4.08 (dt, *J* = 11.7, 2.9 Hz, 1H), 3.94 – 3.68 (m, 2H), 3.54 – 3.44 (m, 1H), 2.69 – 2.35 (m, 4H).

$^{13}$C NMR (75 MHz, $CDCl_3$) δ 169.8 (s), 144.3 (s), 132.0 (s), 129.9 (d), 127.8 (d), 73.0 (d), 65.9 (t), 46.9 (t), 45.1 (t), 21.5 (q).

MS (ESI): 284.91 m/z $[M-H]^-$

$[\alpha]^D{}_{20}$: -17° (c: 0.001 in $CHCl_3$)

90:10 *er* HPLC (AD-H column 20cm, 5μm, l = 230 nm, n-Hex/EtOH = 85:15 + 0.1% TFA, 1 mL/min): tR = 21.8 min (minor 28.2 min (major).

## Synthesis of derivative 6

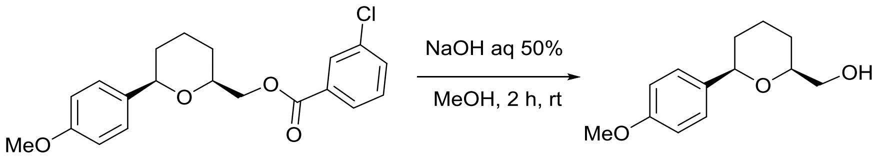


To a solution of the compound **3ma** (20 mg, 0.05 mmol) in MeOH (1 mL, 0.05 M), an aqueous solution 50% m/m NaOH was added. The solution was stirred for 2 h at room temperature. Then, the organic solvent was evaporated and the aqueous residue was rinsed with EtOAc. The organic phase was extracted and washed with brine. The organic phase was dried over $NaSO_4$, filtered and the solvent evaporated *in vacuo*. The crude residue could be purified by flash chromatography on silica gel (n-Hex/EtOAc 4:1) affording the compound **6** (11 mg, 0.047 mmol, 95%) as a colourless oil.

### *((2S,6R)*-6-(4-methoxyphenyl)tetrahydro-2H-pyran-2-yl)methanol (6)

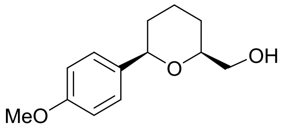


$R_f$ = 0.41 (n-Hex/$Et_2O$ 2:1); stained with molybdenum

The data are in agreement with those reported in literature.[17]

$[\alpha]^D_{20}$: +35° (c: 0.001 in $CHCl_3$)

95:5 *er* HPLC (Amylose column 20cm, 3μm, l = 230 nm, n-Hex/iPrOH = 90:10, 1 mL/min): tR = 10.3. min (major) 12.4 min (minor).

[17] C. R. Reddy, P. P. Madhavi, S. Chandrasekhar, *Tetrahedron: Asymmetry,* **2010,** 21, 103–105

# X-Ray Cristallography (Prof. Leonardo Lo Presti)

## Single crystal X-ray diffraction analysis of the compound 3da

**Sample specs:**

Crystallization method: slow evaporation from n–hexane:EtOAc 1:1

Sample description: needle, colourless, transparent, with dimensions ≈ 0.475 x 0.050 x 0.050 mm.

Mounting: on a glass fibre, with two–component epoxy glue.

Comments: The sample shows pleochroism (colourless to green). It was cut from a larger agglomerate through a thin iron needle and polished by mechanical ablation in a perfluorinated oil drop.

**Instrumental specs:**

Device: Bruker AXS Smart APEX 3-circle diffractometer (Unimi-CNR)

Source: normal focus sealed tube

Detector: APEX-II CCD

Experiment temperature: 298(2) K

Cryostat: not used

Wavelength: graphite-monochromated Mo K α (0.71073 Å).

Data collection extent: full sphere within $\sin\vartheta/\lambda = 0.59$ Å$^{-1}$

Data collections specs: Detector-to-sample distance: 50 mm,

ω-scan, $2\vartheta = \omega$ i = 0 deg , ω-sweep 180 deg, $\Delta\omega = 0.25$ deg, t / frame = 60 s, 1 run (φ = 0 deg)

ω-scan, $2\vartheta = \omega$ i = -30 deg , ω-sweep 180 deg, $\Delta\omega = 0.25$ deg, t / frame = 90 s, 4 runs (φ = 0, 90, 180, 270 deg).

Measured reflections: 2263; 1152 with $I > 2\sigma(I)$

Maximum resolution (2ϑ): 49.41 deg

Completeness: 89.8 %

**Data reduction programs:**

Integration: SAINT+

Reduction: SADABS, XPREP

Structure solution and refinement: shelxs (direct methods), shelxl

**Unit cell, lattice and crystal system**:

Bravais lattice: Monoclinic, primitive

Space group: $P2_1$, nº 4, acentric, polar, chiral

Point group: 2 ($C_2$)

Laue group: 2/m, number 2

Unit cell (Å, deg, Å 3 ): a = 5.5093(18), b = 15.969(5), c = 11.255(3), α = 90.00, β = 99.692(18), γ = 90.00, V = 976.1(5) as estimated from 409 intense reflections among 4.5 and 36.2 deg in 2ϑ (final integration result).

Formula units in cell (Z): 2

Formula units in the asymmetric unit (Z'): 1

Number of electrons in cell ($F_{000}$): 456

<u>Computed density:</u> 1.500 g/cm$^3$

<u>Linear absorption coefficient (μ)</u>: 0.347 mm$^{-1}$

**Main statistical results:**

Final stats for the spherical atom model (shelxl):

Scale factor: 0.301(1)

Secondary extinction coefficient: none

$<\Delta/\sigma> = 0.000$

R1(F) = 0.0585 for 1152 $F_o > 4\sigma(F_o)$, 0.1399 for all the 3232 independent data

wR($F^2$)= 0.1648 for all the independent data

Weighting scheme: WGHT 0.0796

Goodness-of-fit: 0.946

$\Delta\rho$ MAX/MIN = +0.23/ –0.25 e/Å$^3$

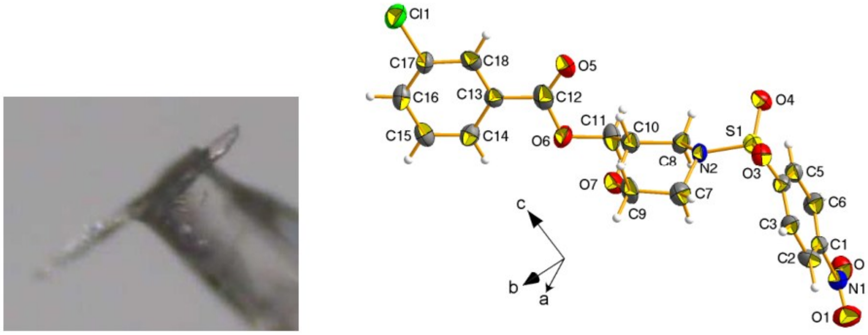


(a) (b)

***Figure S5***. (a) Sample used for the present structural determination. (b) Asymmetric unit of **3da**, with the atom-numbering scheme and crystallographic reference system. Thermal ellipsoids at RT were drawn at the 30 % probability level. Atoms are represented with the usual CPK colour code (C: grey; N: blue; O: red; H: white; Cl: light green; S: yellow).

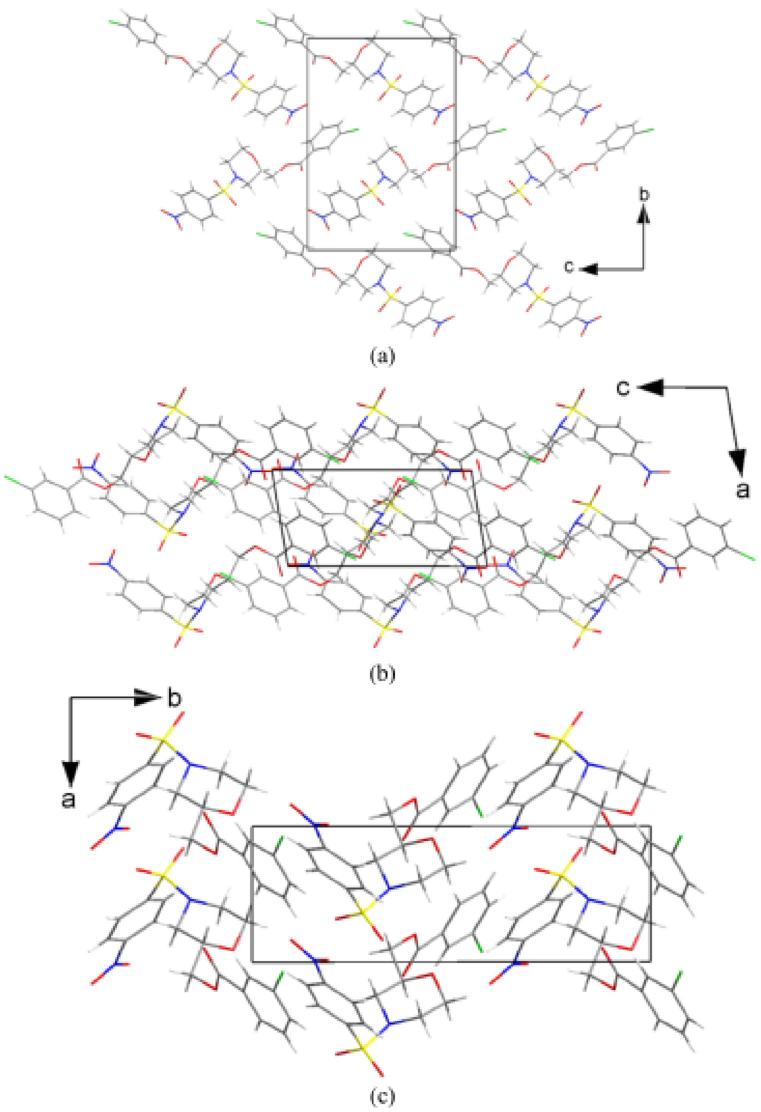


***Figure S6***. Wires–stick representation of the crystal packing of **3da** at RT, as seen along the a (a), b (b) and c (c) cell axes. Colour code as in Figure S6.

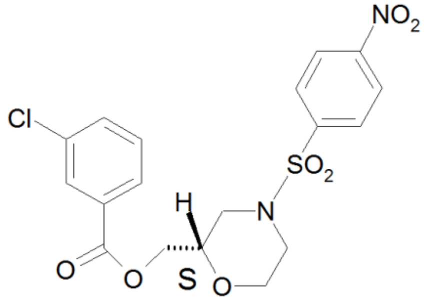


***Figure S7***. Molecular structure of **3da**, as retrieved from the X-ray diffraction experiment. The configurational descriptor at the unique chiral centre C10 (see Figure S6) is shown.

The compound is chiral and crystallizes in the acentric chiral space group $2_1$, with 1 formula in the asymmetric unit and 2 molecule per cell. Even though the coverage statistics of centrosymmetric reflection pairs is not full due to the poor scattering power of the specimen, anomalous dispersion effects suggest that the most probable absolute configuration at the asymmetric carbon C10 is S. The corresponding Flack parameter (Acta Crystallogr. A Found. Adv. 1983, 39, 876–881) reads 0.1(2) from the classical fit to all intensities and 0.27(6) from 314 selected quotients according to the Parson's method (Parson et al., Acta Crystallogr. B Struct. Sci. Cryst. Eng. Mater. 2013, 69, 249–259).

Figure S6 shows the main packing motifs in the (*b*,*c*), (*a*,*c*) and (*a*,*b*) planes. **3da** lacks strong hydrogen bond (HB) donors and adopts an elongate zig–zag conformation (compare Figure S7a and Figure S7c), likely to maximize the surface contact area and take thus advantage of van der Waals interactions. Therefore, molecules form slanted rows along *b*, whose individual elements are related by the $2_1$ axis. No stacking interactions among aromatic rings are recognizable.

The morpholine ring adopts a perfect chair conformation, with puckering parameters according to Cremer & Pople (J. Amer. Chem. Soc. 1975, 97, 1354–1358) of Q = 0.56(1) Å, θ = 3 (13) deg, φ = 44(20) deg.

For further information, see crystallographic data, which is available free of charge from the Cambridge Crystallographic Data Centre (www.ccdc.cam.ac.uk/data_request/cif) under the deposition number 2078892.

## $^{1}H$ NMR and $^{13}C$ NMR spectra and HPLC Data

## $^{1}$H NMR and $^{13}$C NMR spectra

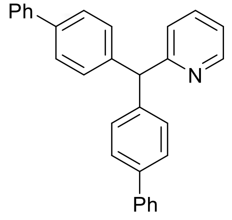


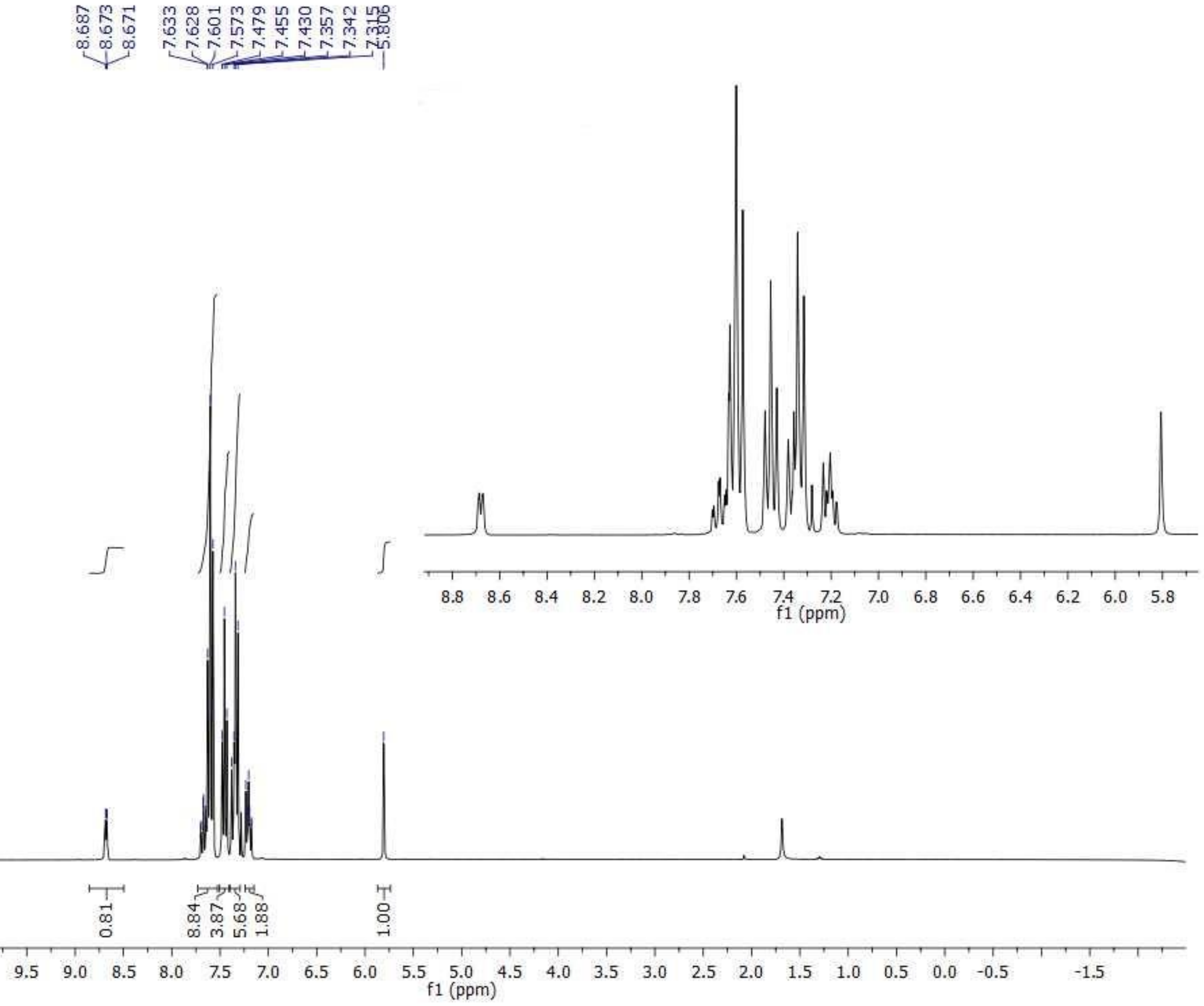

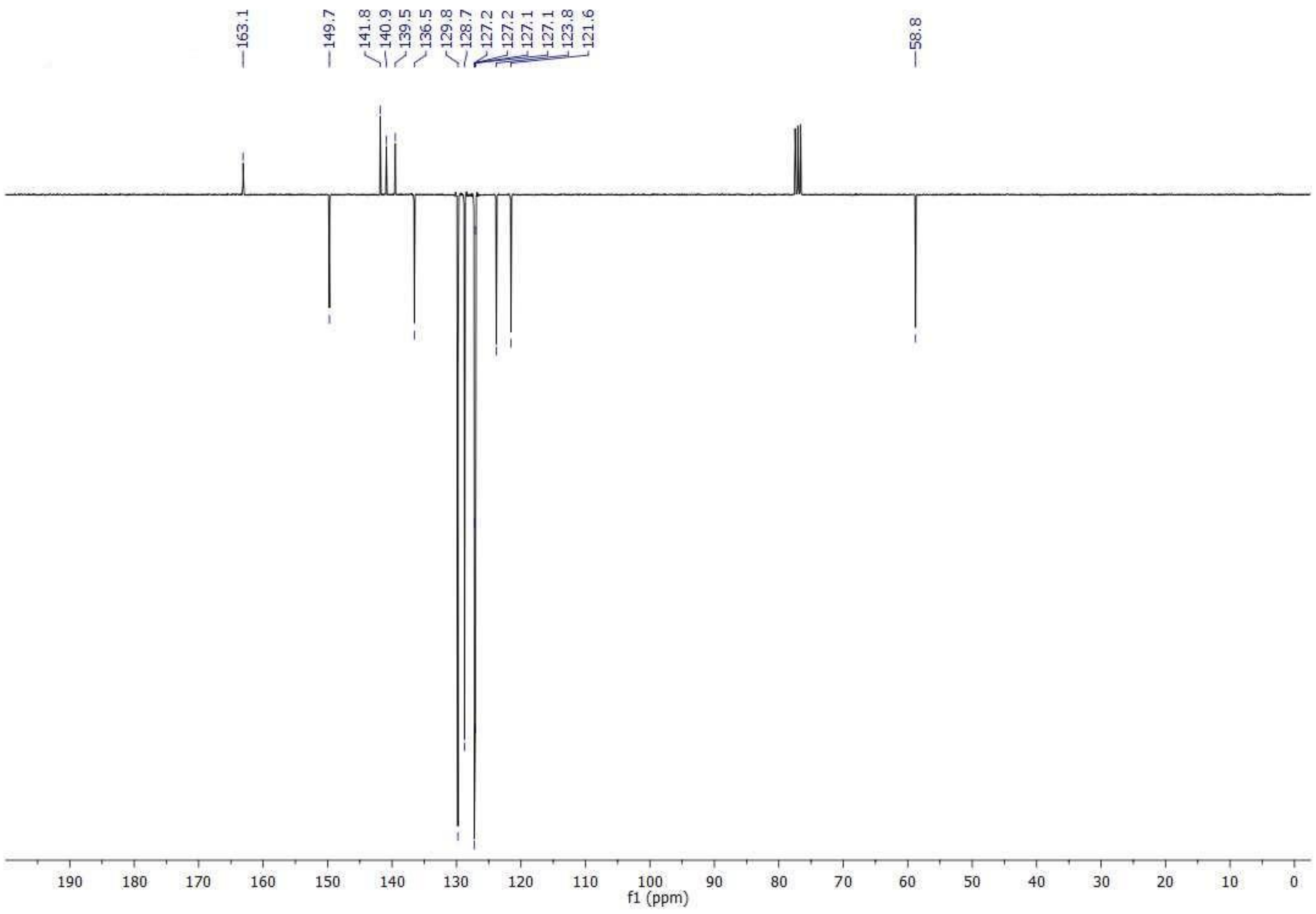
163.1
149.7
141.8
140.9
139.5
136.5
129.8
128.7
127.2
127.2
127.1
127.1
123.8
121.6
58.8
190 180 170 160 150 140 130 120 110 100 90 80 70 60 50 40 30 20 10 0
f1 (ppm)

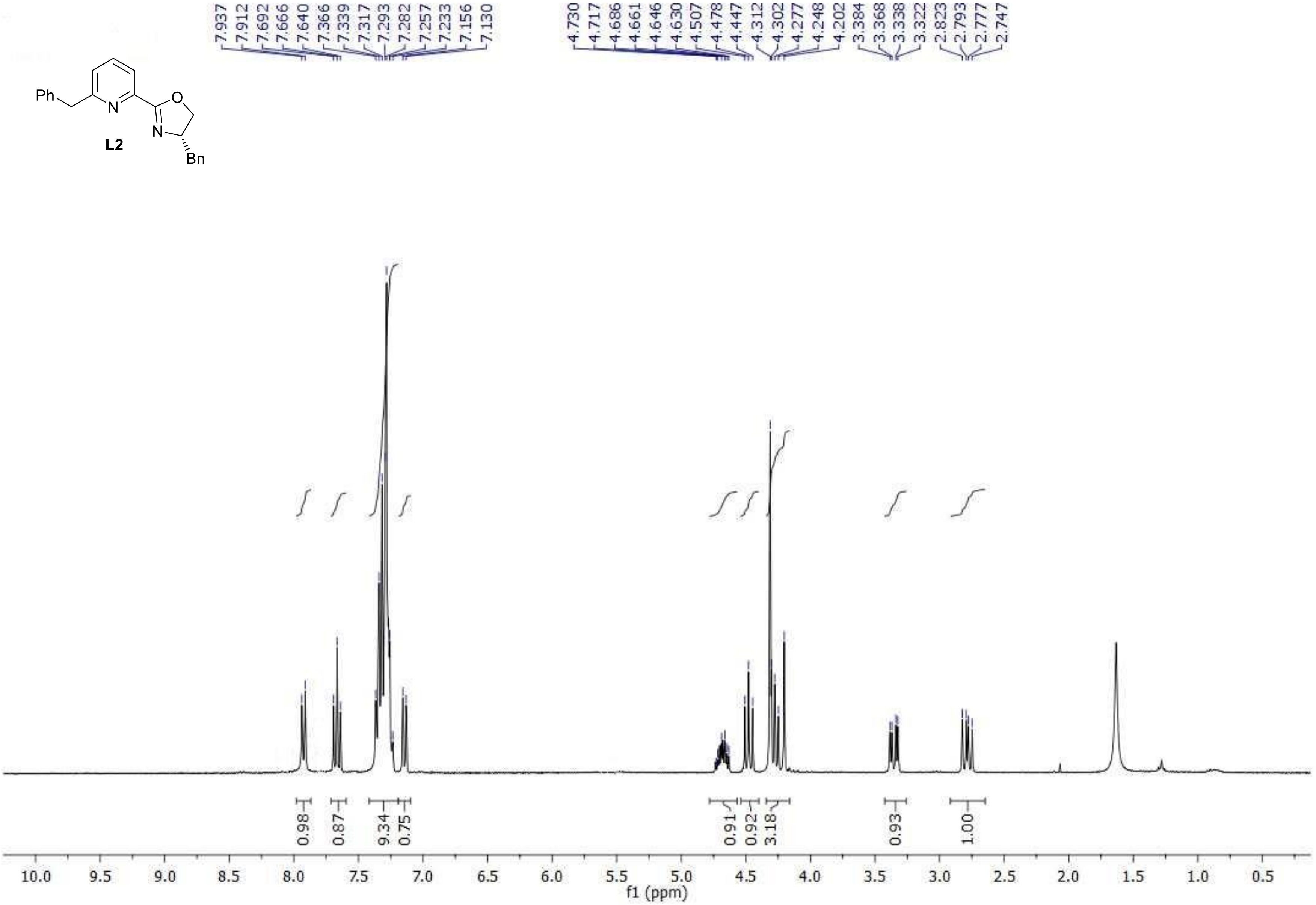
L2
Ph
N
O
N
Bn
7.937 7.912 7.692 7.666 7.640 7.366 7.339 7.317 7.293 7.282 7.257 7.233 7.156 7.130
4.730 4.717 4.686 4.661 4.646 4.630 4.507 4.478 4.447 4.312 4.302 4.277 4.248 4.202 3.384 3.368 3.338 3.322 2.823 2.793 2.777 2.747
0.98 0.87 9.34 0.75 0.91 0.92 3.18 0.93 1.00
10.0 9.5 9.0 8.5 8.0 7.5 7.0 6.5 6.0 5.5 5.0 4.5 4.0 3.5 3.0 2.5 2.0 1.5 1.0 0.5
f1 (ppm)

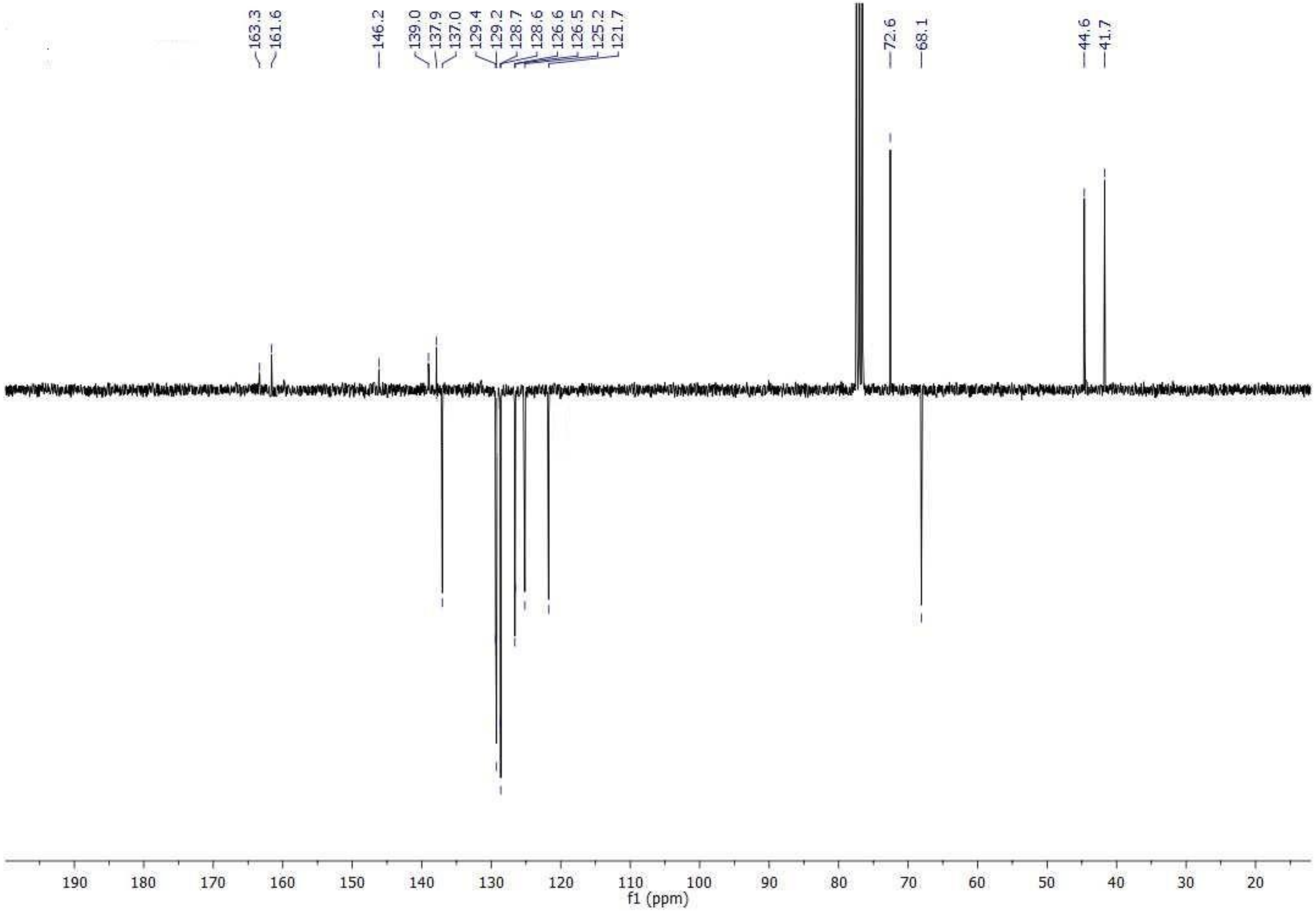
163.3
161.6
146.2
139.0
137.9
137.0
129.4
129.2
128.7
128.6
126.6
126.5
125.2
121.7
72.6
68.1
44.6
41.7
190
180
170
160
150
140
130
120
110
100
90
80
70
60
50
40
30
20
f1 (ppm)

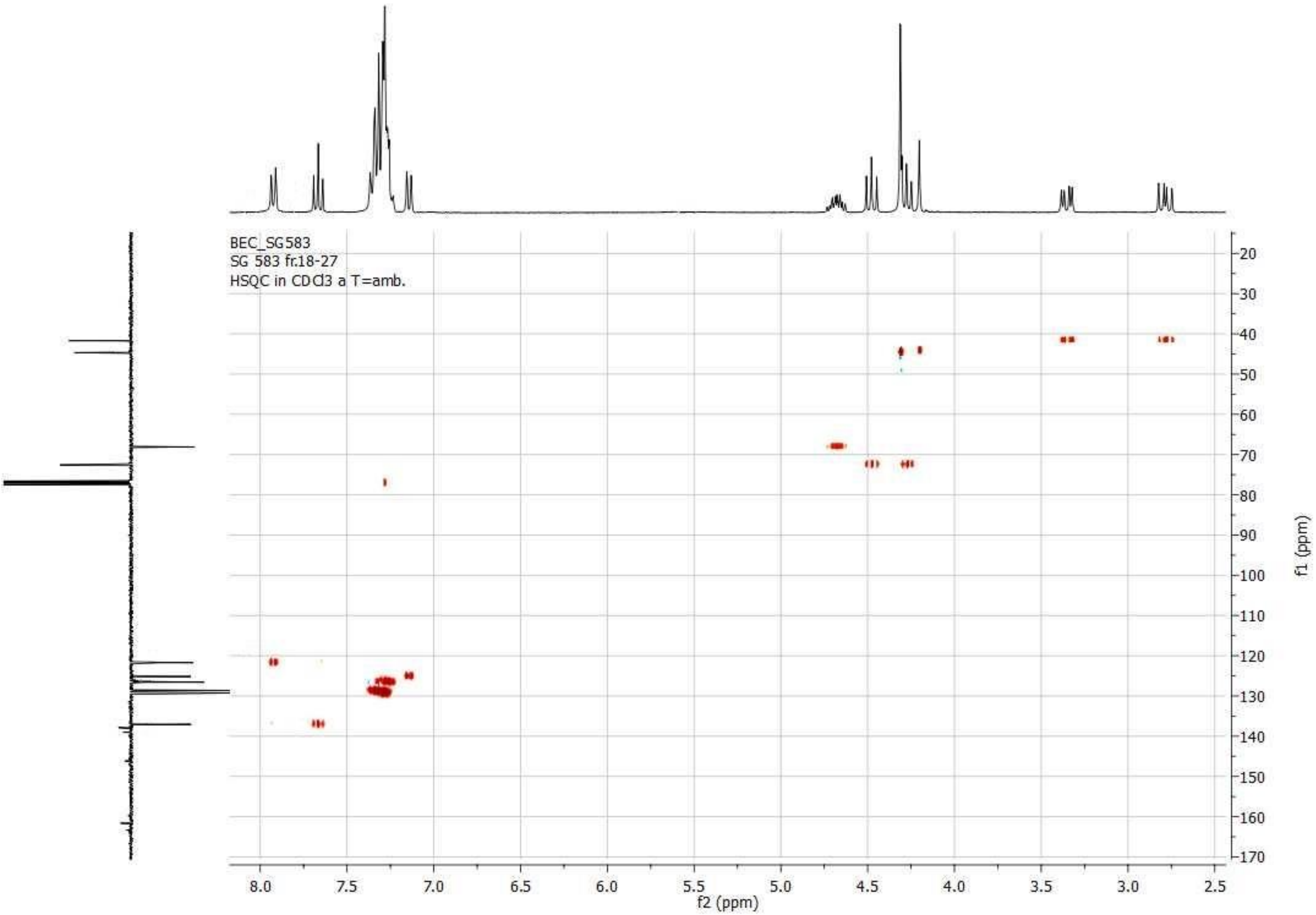

BEC_SG583
SG 583 fr.18-27
HSQC in CDCl3 a T=amb.
f2 (ppm)
f1 (ppm)

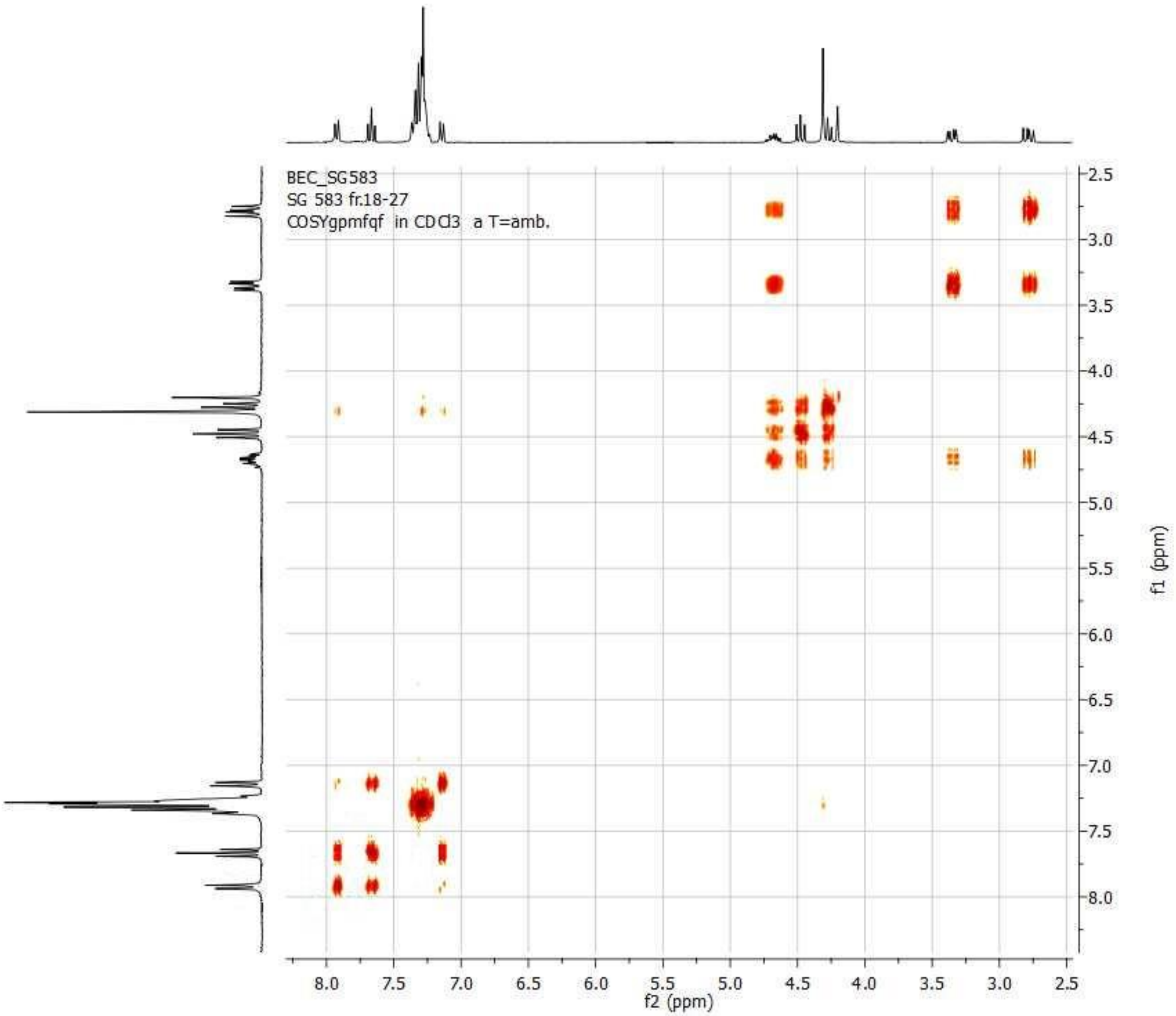
BEC_SG583
SG 583 fr.18-27
COSYgpmfqf in CDCl3 a T=amb.
f1 (ppm)
f2 (ppm)

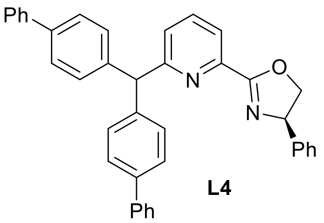
Ph
N
O
N
Ph
Ph

**L4**

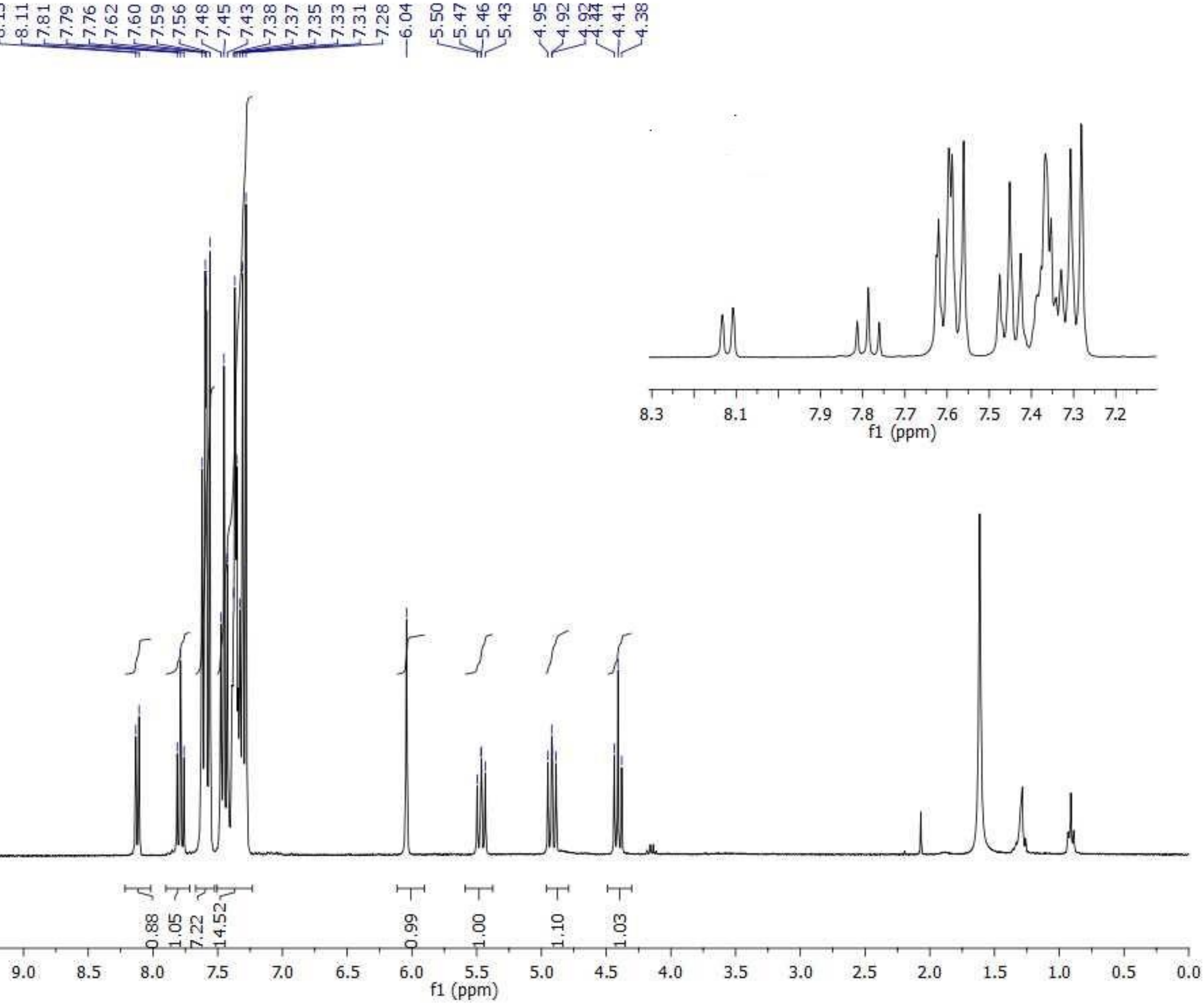
8.13
8.11
7.81
7.79
7.76
7.62
7.60
7.59
7.56
7.48
7.45
7.43
7.38
7.37
7.35
7.33
7.31
7.28
6.04
5.50
5.47
5.46
5.43
4.95
4.92
4.92
4.44
4.41
4.38
0.88
1.05
7.22
14.52
0.99
1.00
1.10
1.03
11.0 10.5 10.0 9.5 9.0 8.5 8.0 7.5 7.0 6.5 6.0 5.5 5.0 4.5 4.0 3.5 3.0 2.5 2.0 1.5 1.0 0.5 0.0
f1 (ppm)
8.3 8.1 7.9 7.8 7.7 7.6 7.5 7.4 7.3 7.2
f1 (ppm)

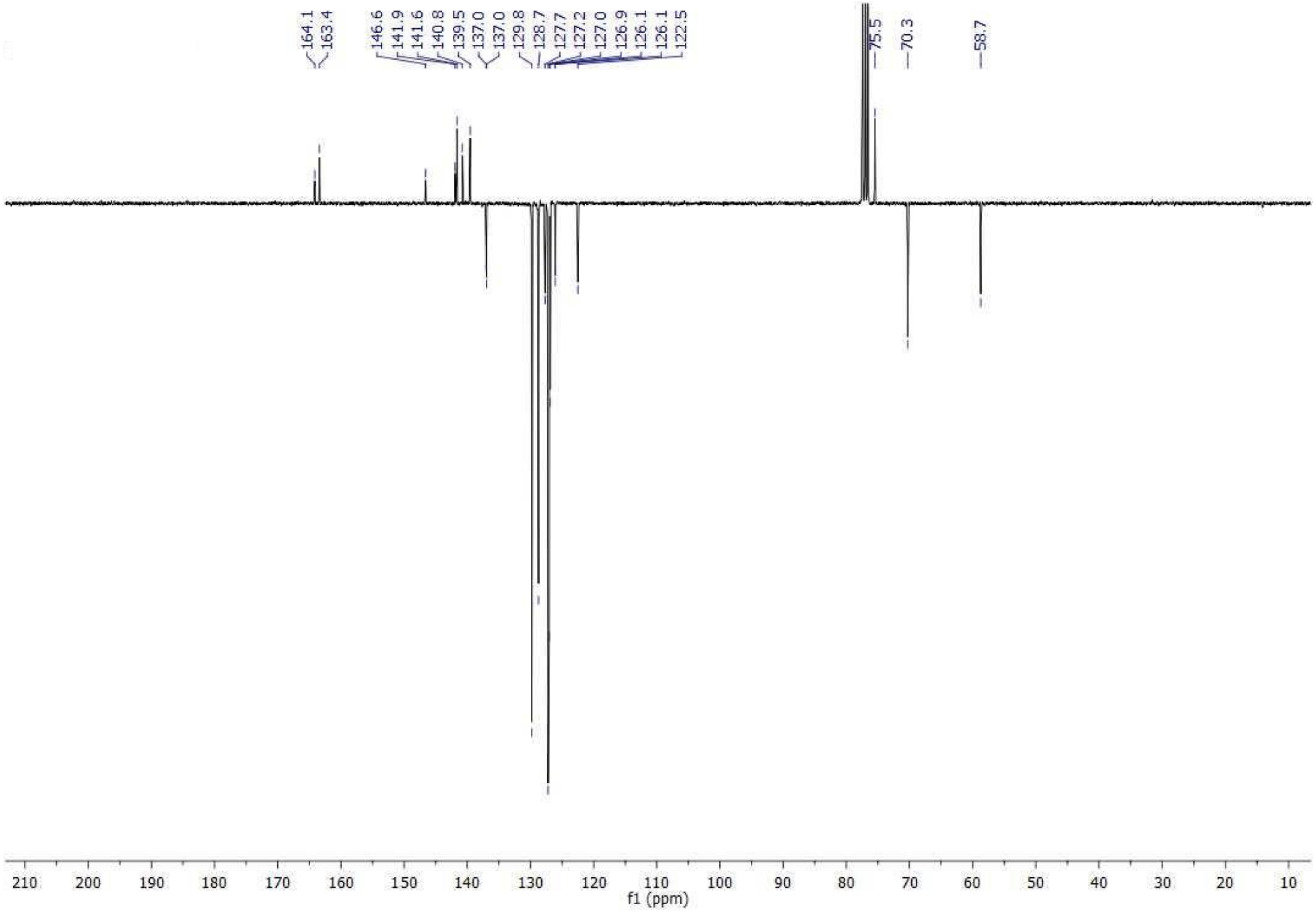
164.1
163.4
146.6
141.9
141.6
140.8
139.5
137.0
137.0
129.8
128.7
127.7
127.2
127.0
126.9
126.1
126.1
122.5
75.5
70.3
58.7
210 200 190 180 170 160 150 140 130 120 110 100 90 80 70 60 50 40 30 20 10
f1 (ppm)

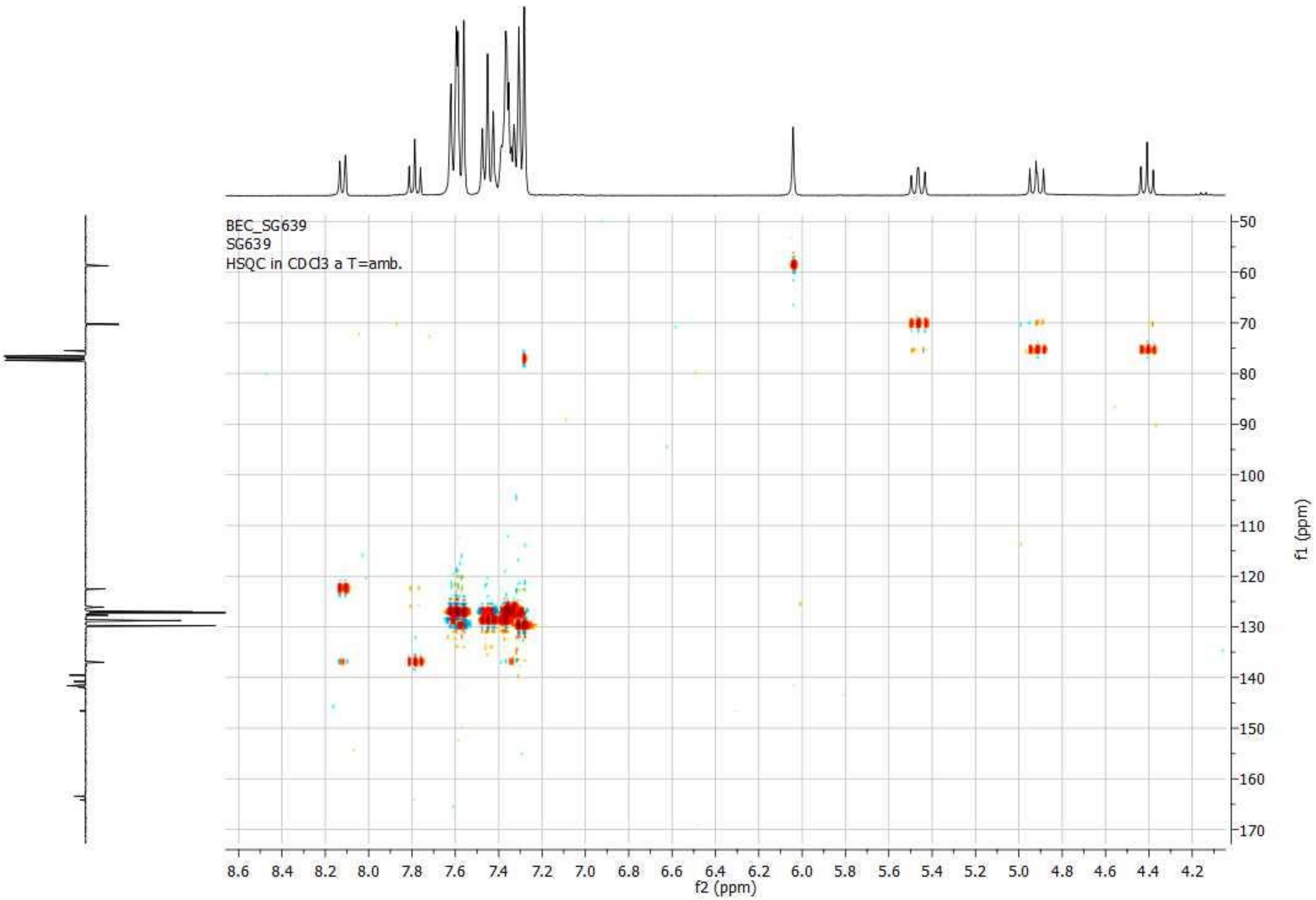

BEC_SG639
SG639
HSQC in CDCl3 a T=amb.
f2 (ppm)
f1 (ppm)

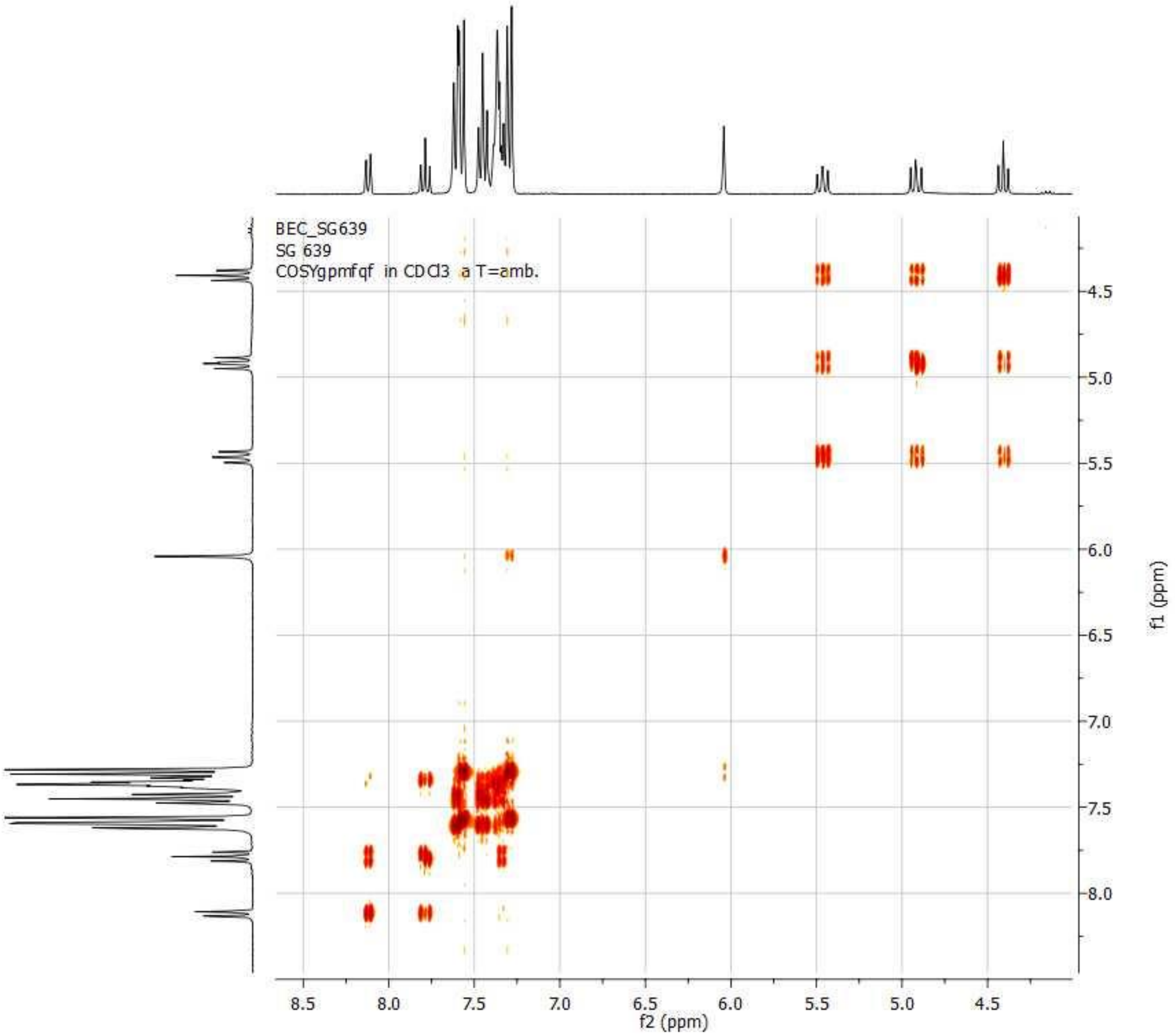

BEC_SG639
SG 639
COSYgpmfqf in CDCl3 a T=amb.
f2 (ppm)
f1 (ppm)

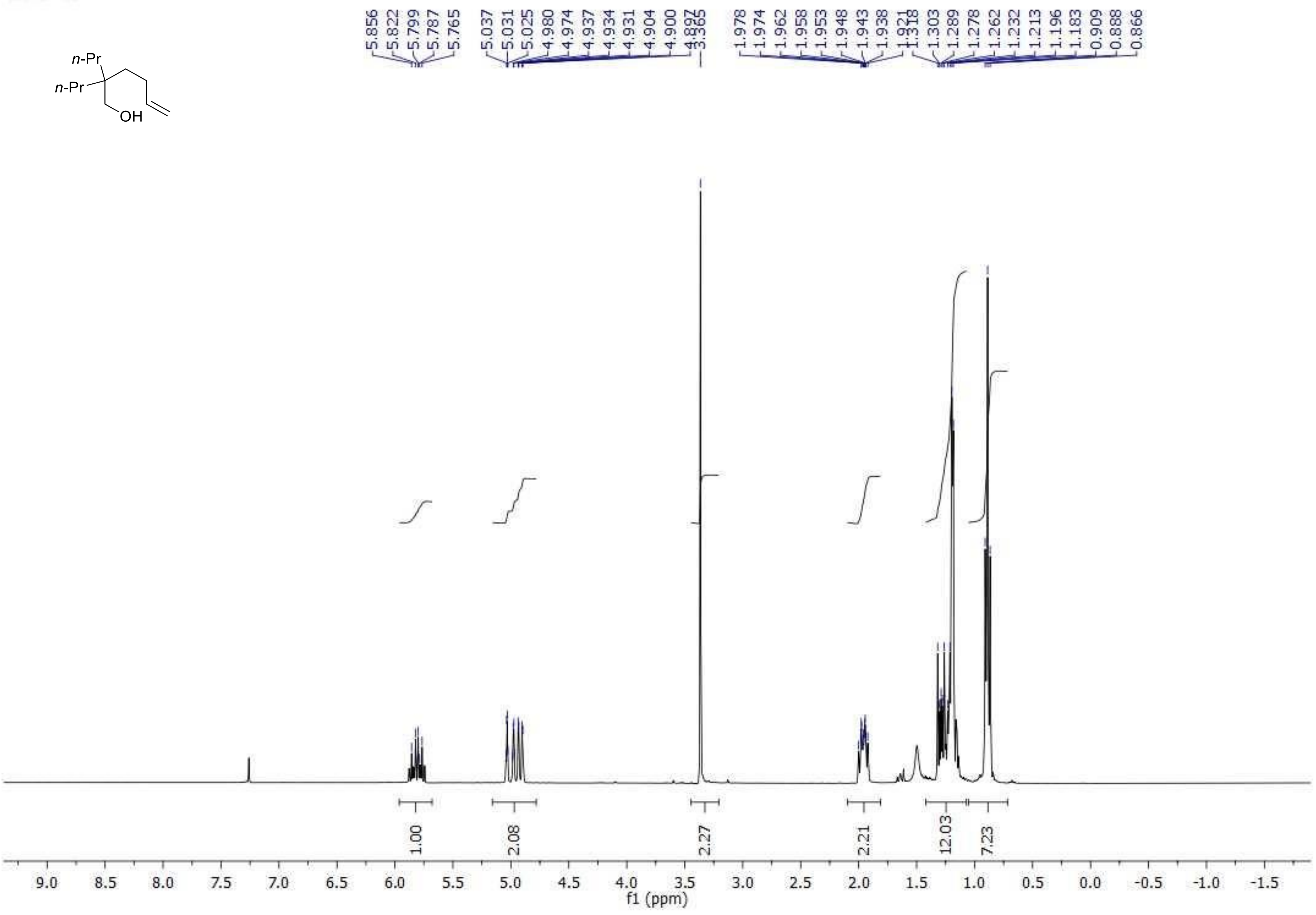

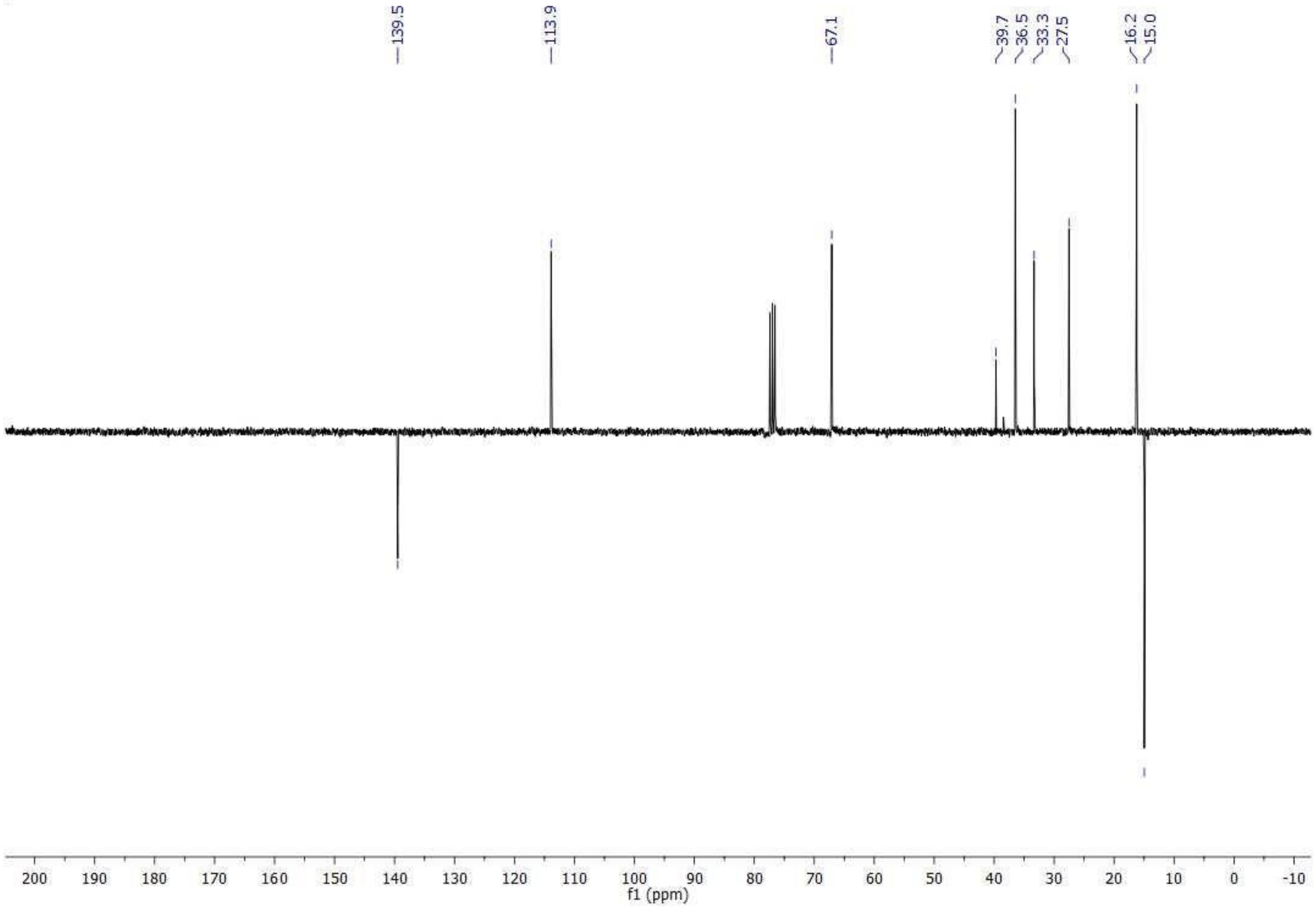

139.5
113.9
67.1
39.7
36.5
33.3
27.5
16.2
15.0
200
190
180
170
160
150
140
130
120
110
100
90
80
70
60
50
40
30
20
10
0
-10
f1 (ppm)

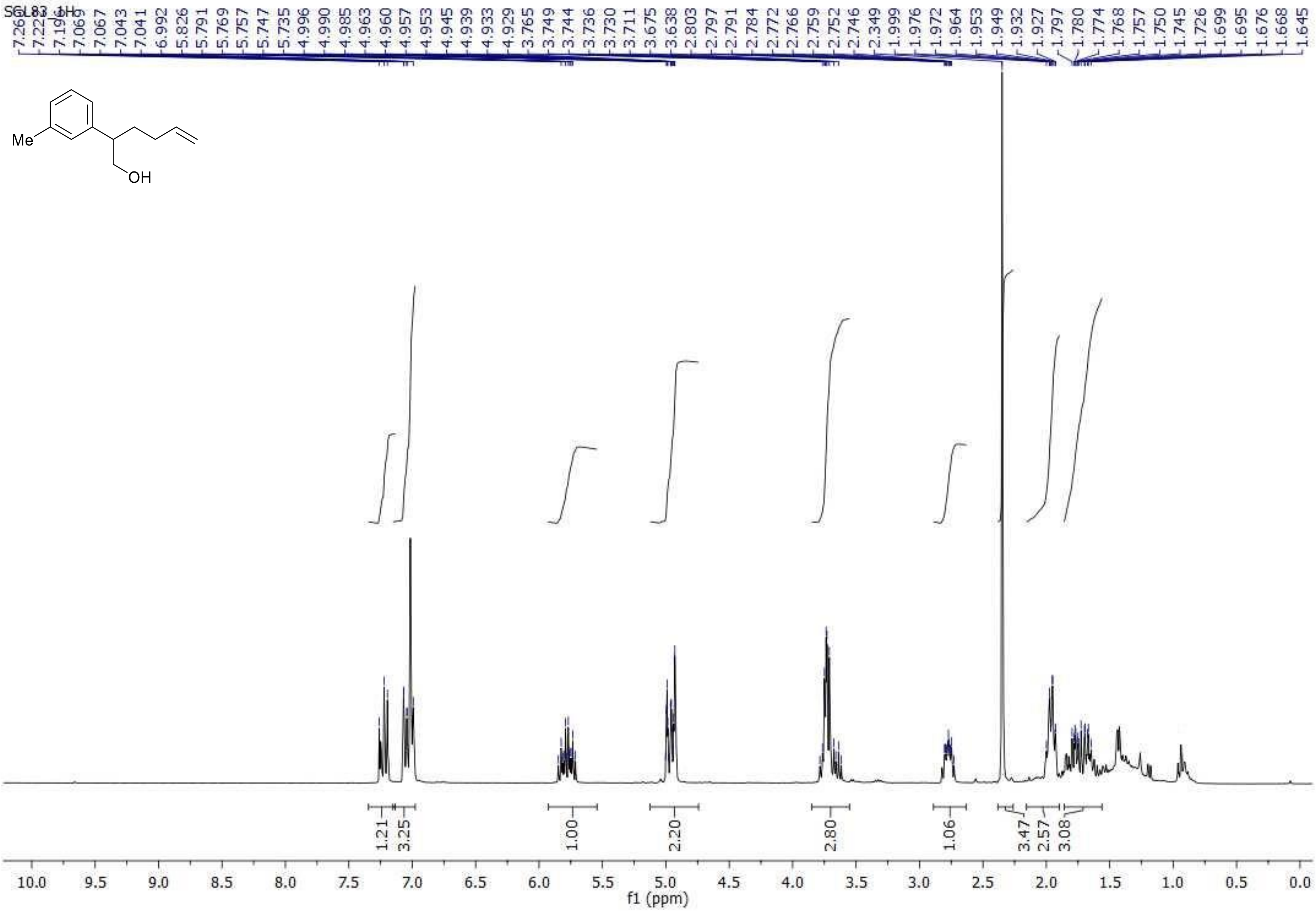
SGL83_1H
Me
OH
f1 (ppm)

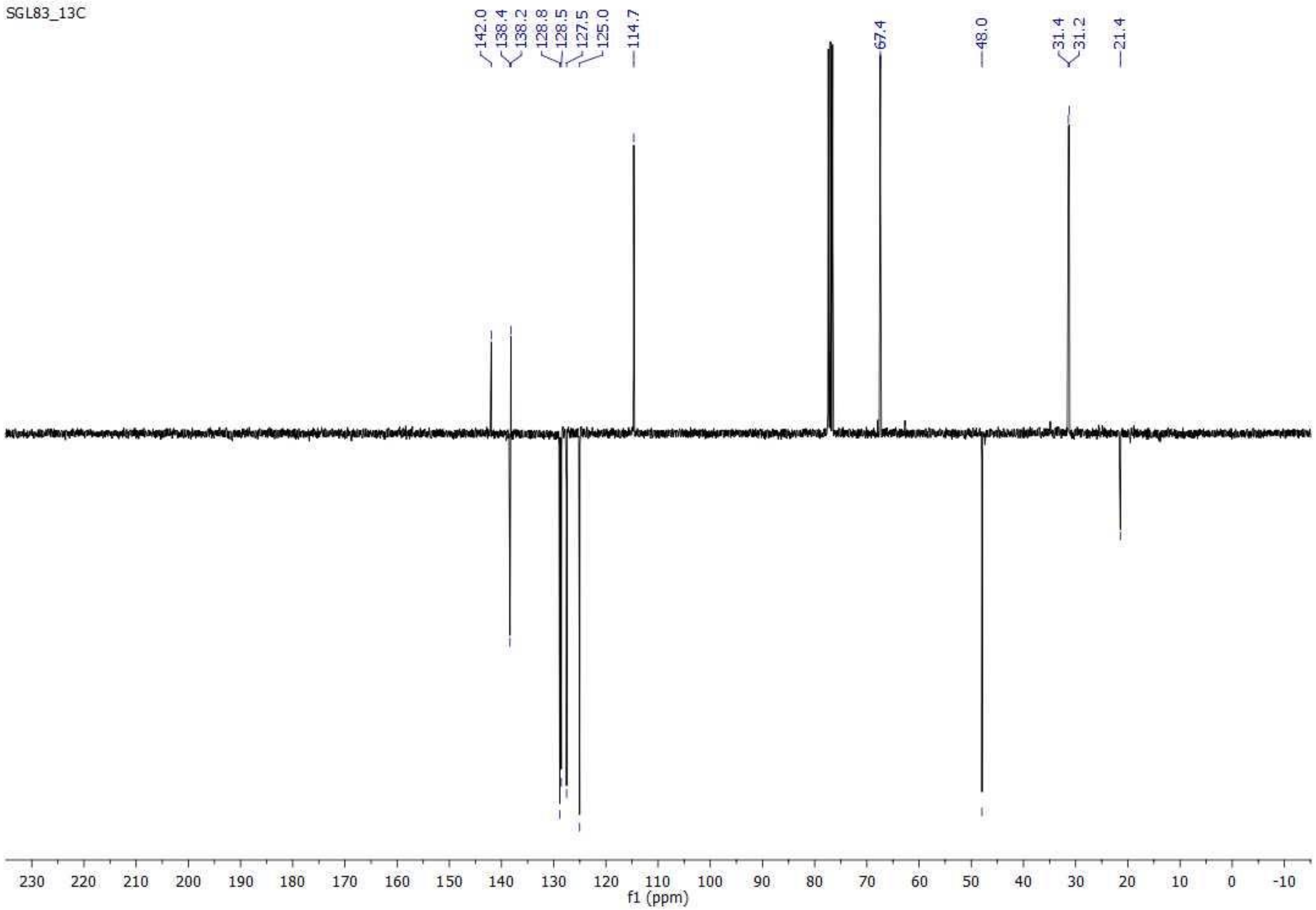

SGL83_13C
142.0
138.4
138.2
128.8
128.5
127.5
125.0
114.7
67.4
48.0
31.4
31.2
21.4
230 220 210 200 190 180 170 160 150 140 130 120 110 100 90 80 70 60 50 40 30 20 10 0 -10
f1 (ppm)

SGL116_1H

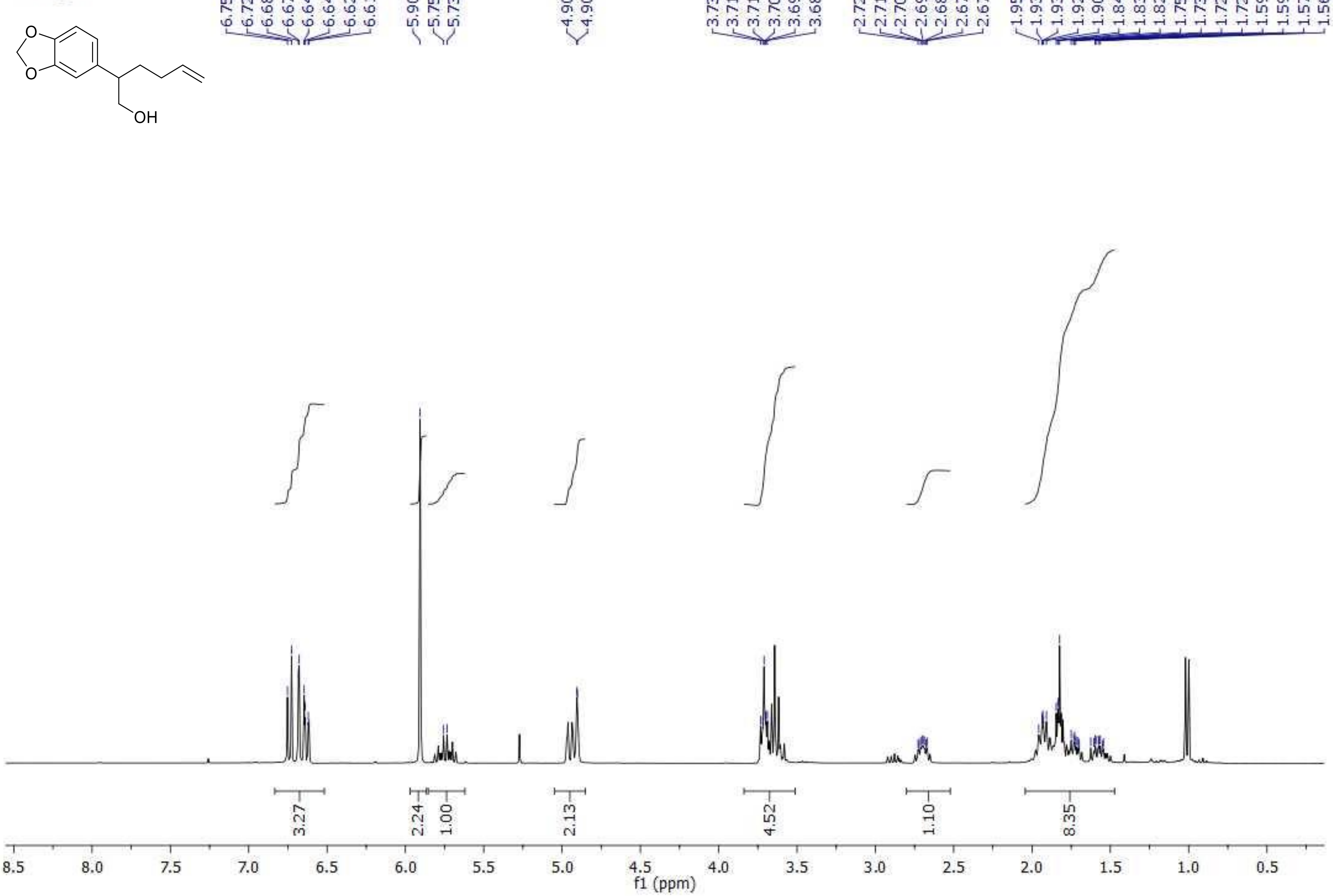
OH
O
O
6.753
6.727
6.683
6.678
6.646
6.641
6.620
6.614
5.906
5.756
5.734
4.905
4.902
3.733
3.719
3.711
3.703
3.698
3.689
2.727
2.711
2.703
2.696
2.687
2.675
2.670
1.957
1.936
1.931
1.926
1.908
1.845
1.835
1.824
1.750
1.734
1.728
1.721
1.597
1.592
1.576
1.564
3.27
2.24
1.00
2.13
4.52
1.10
8.35
8.5
8.0
7.5
7.0
6.5
6.0
5.5
5.0
4.5
4.0
3.5
3.0
2.5
2.0
1.5
1.0
0.5
f1 (ppm)

SGL116_13C

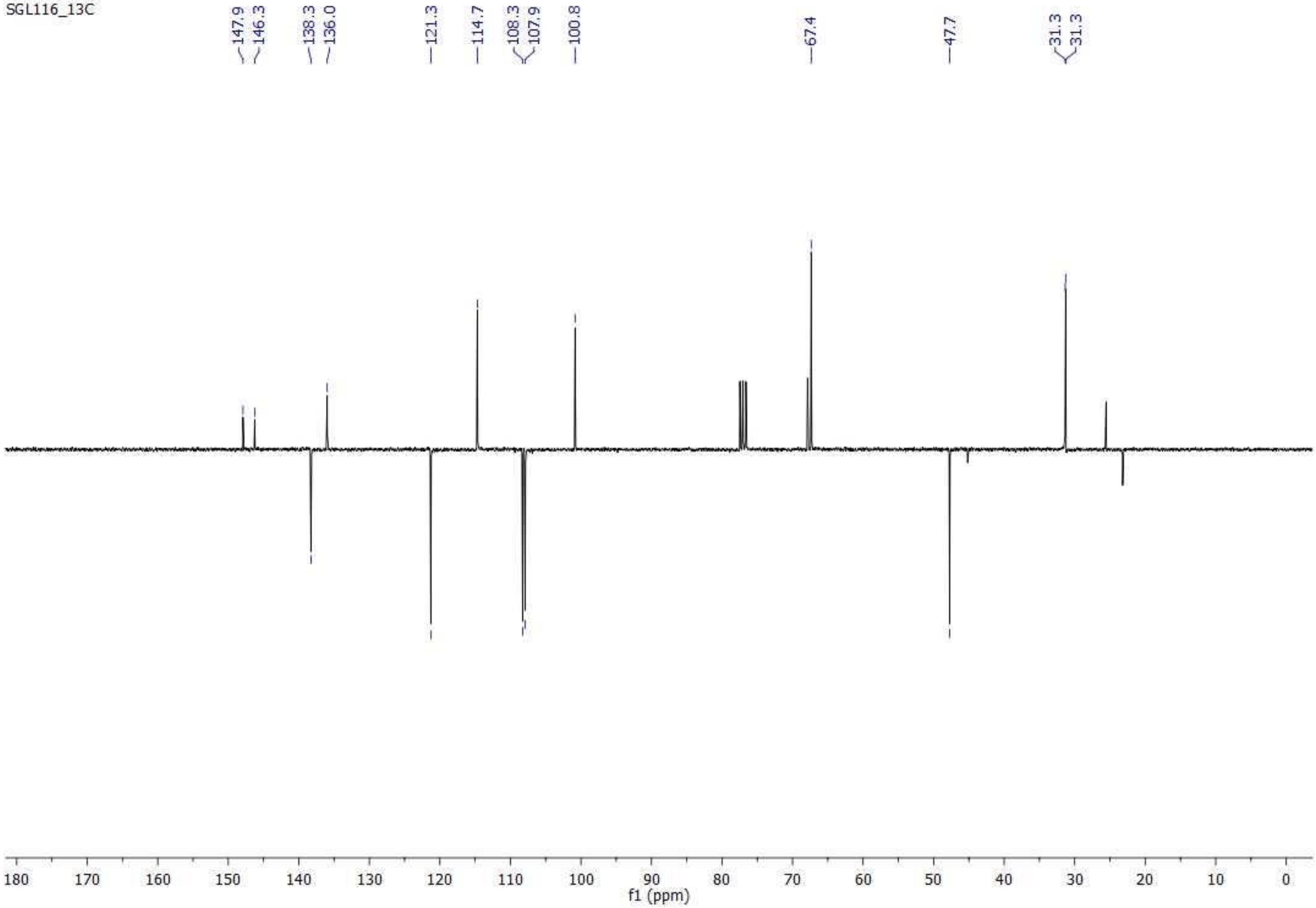

147.9
146.3
138.3
136.0
121.3
114.7
108.3
107.9
100.8
67.4
47.7
31.3
31.3
180
170
160
150
140
130
120
110
100
90
80
70
60
50
40
30
20
10
0
f1 (ppm)

BEC_SGL137
SGL137
1H in CDCl3 a T=300K

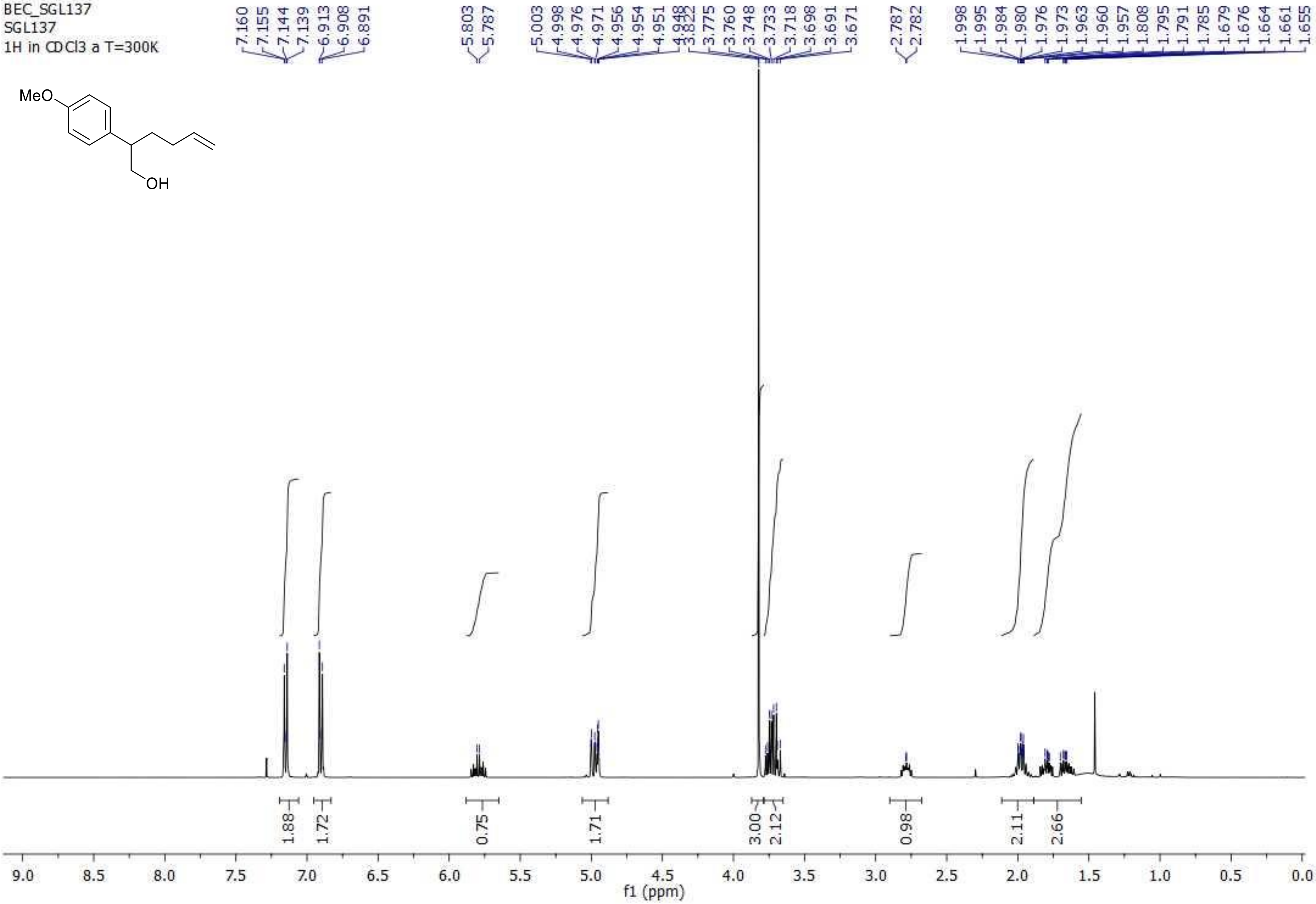

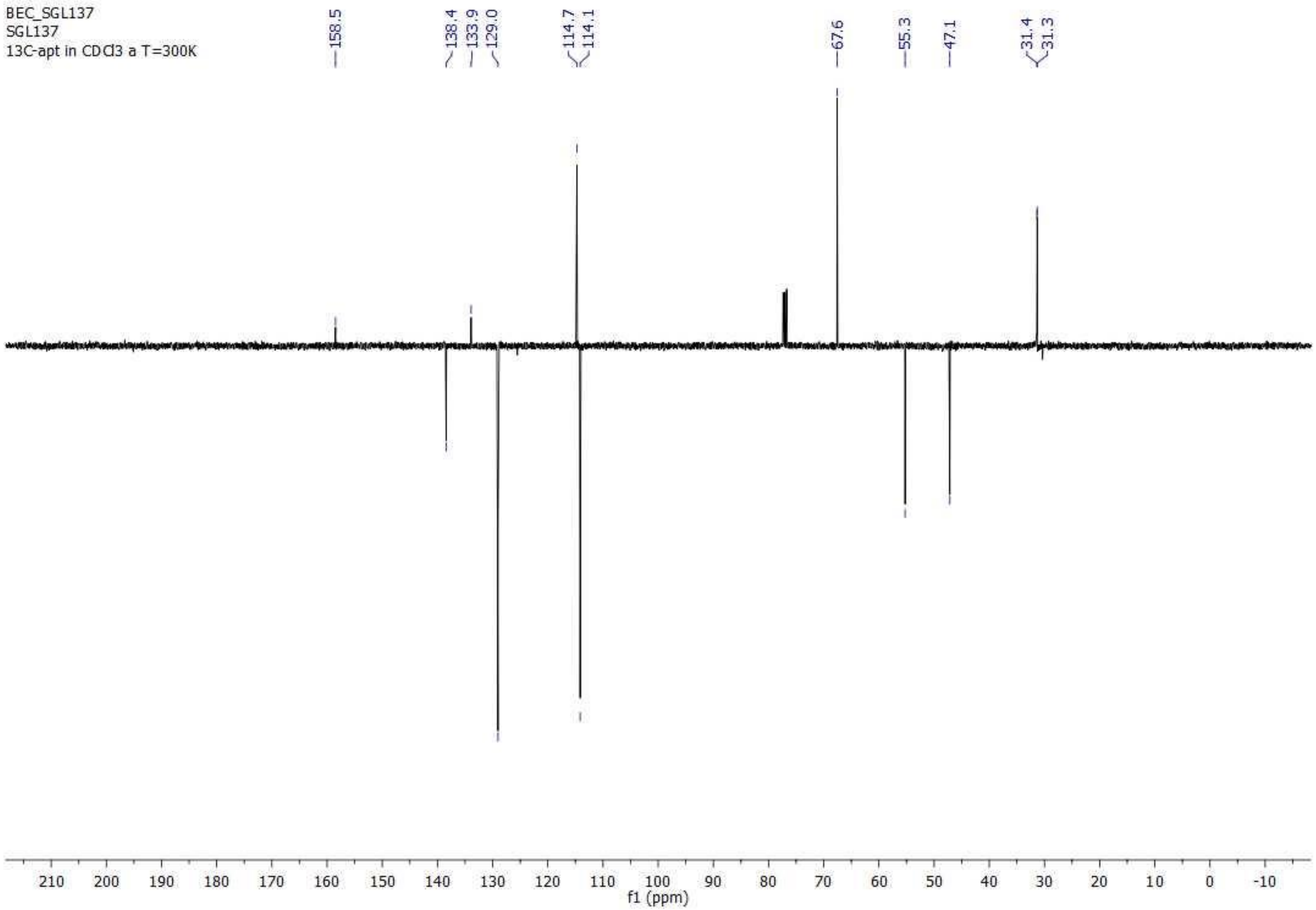
BEC_SGL137
SGL137
13C-apt in CDCl3 a T=300K
158.5
138.4
133.9
129.0
114.7
114.1
67.6
55.3
47.1
31.4
31.3
210
200
190
180
170
160
150
140
130
120
110
100
90
80
70
60
50
40
30
20
10
0
-10
f1 (ppm)

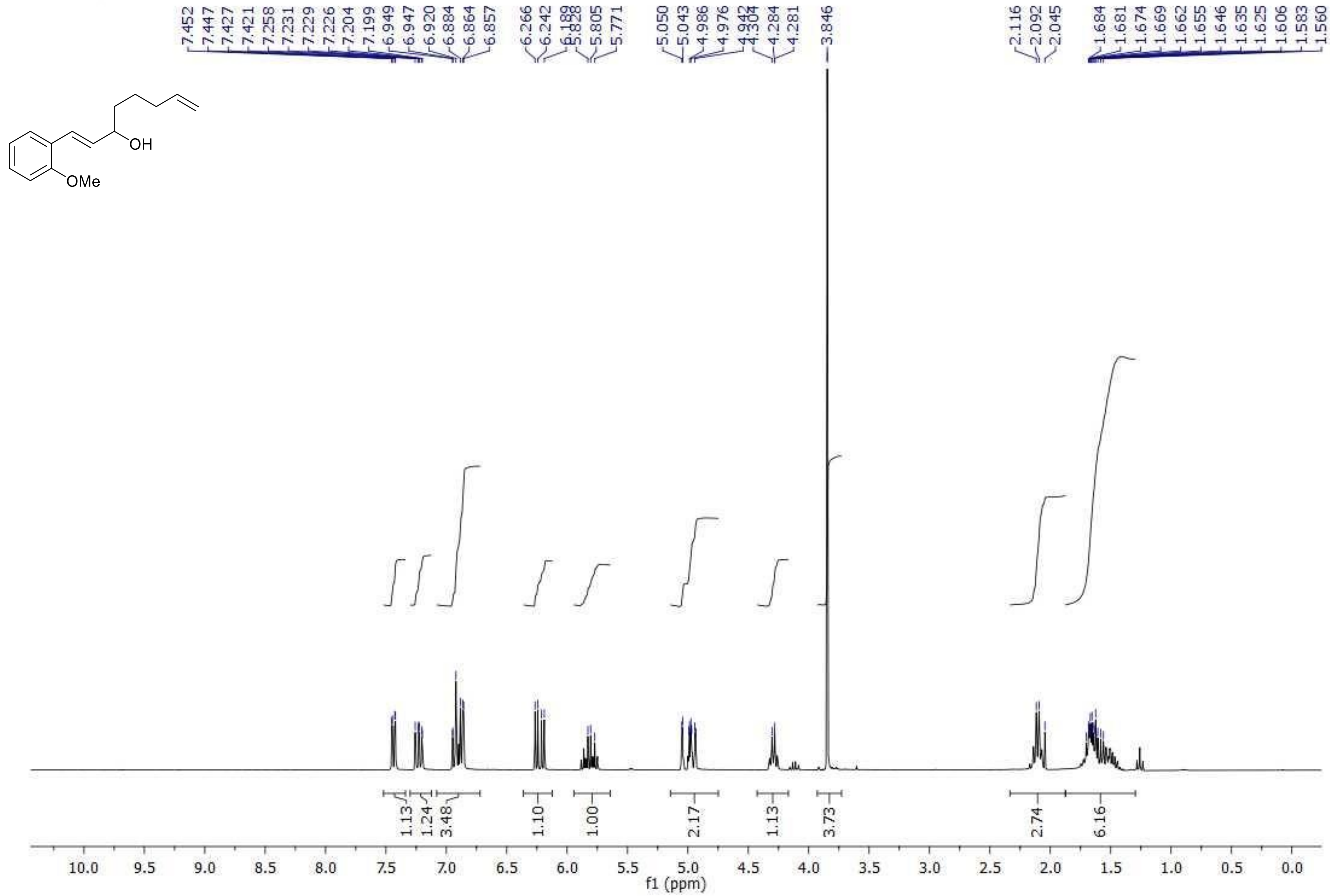

OH
OMe
7.452
7.447
7.427
7.421
7.258
7.231
7.229
7.226
7.204
7.199
6.949
6.947
6.920
6.884
6.864
6.857
6.266
6.242
6.189
5.828
5.805
5.771
5.050
5.043
4.986
4.976
4.942
4.304
4.284
4.281
3.846
2.116
2.092
2.045
1.684
1.681
1.674
1.669
1.662
1.655
1.646
1.635
1.625
1.606
1.583
1.560
1.13
1.24
3.48
1.10
1.00
2.17
1.13
3.73
2.74
6.16
10.0
9.5
9.0
8.5
8.0
7.5
7.0
6.5
6.0
5.5
5.0
4.5
4.0
3.5
3.0
2.5
2.0
1.5
1.0
0.5
0.0
f1 (ppm)

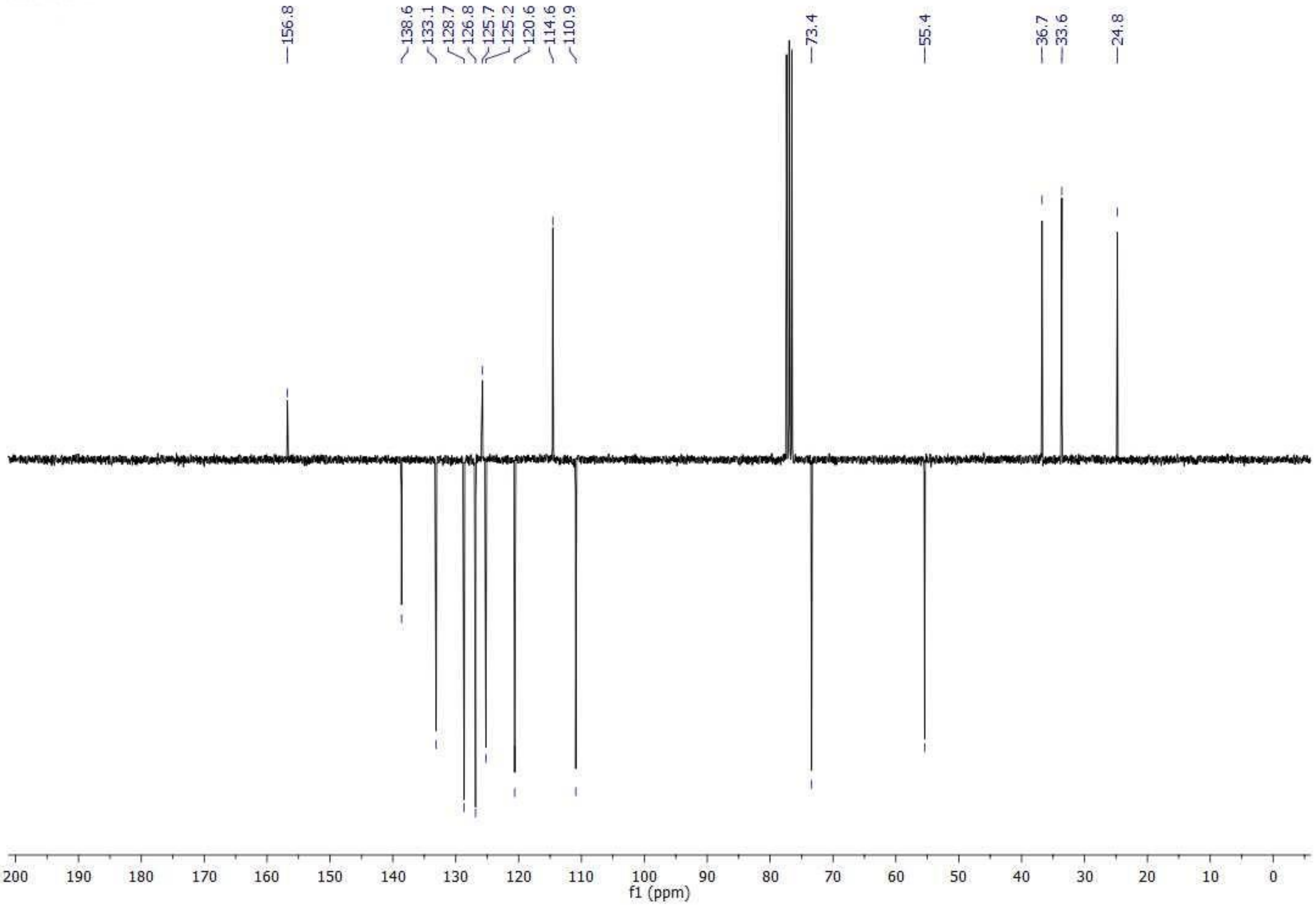
156.8
138.6
133.1
128.7
126.8
125.7
125.2
120.6
114.6
110.9
73.4
55.4
36.7
33.6
24.8
200
190
180
170
160
150
140
130
120
110
100
90
80
70
60
50
40
30
20
10
0
f1 (ppm)

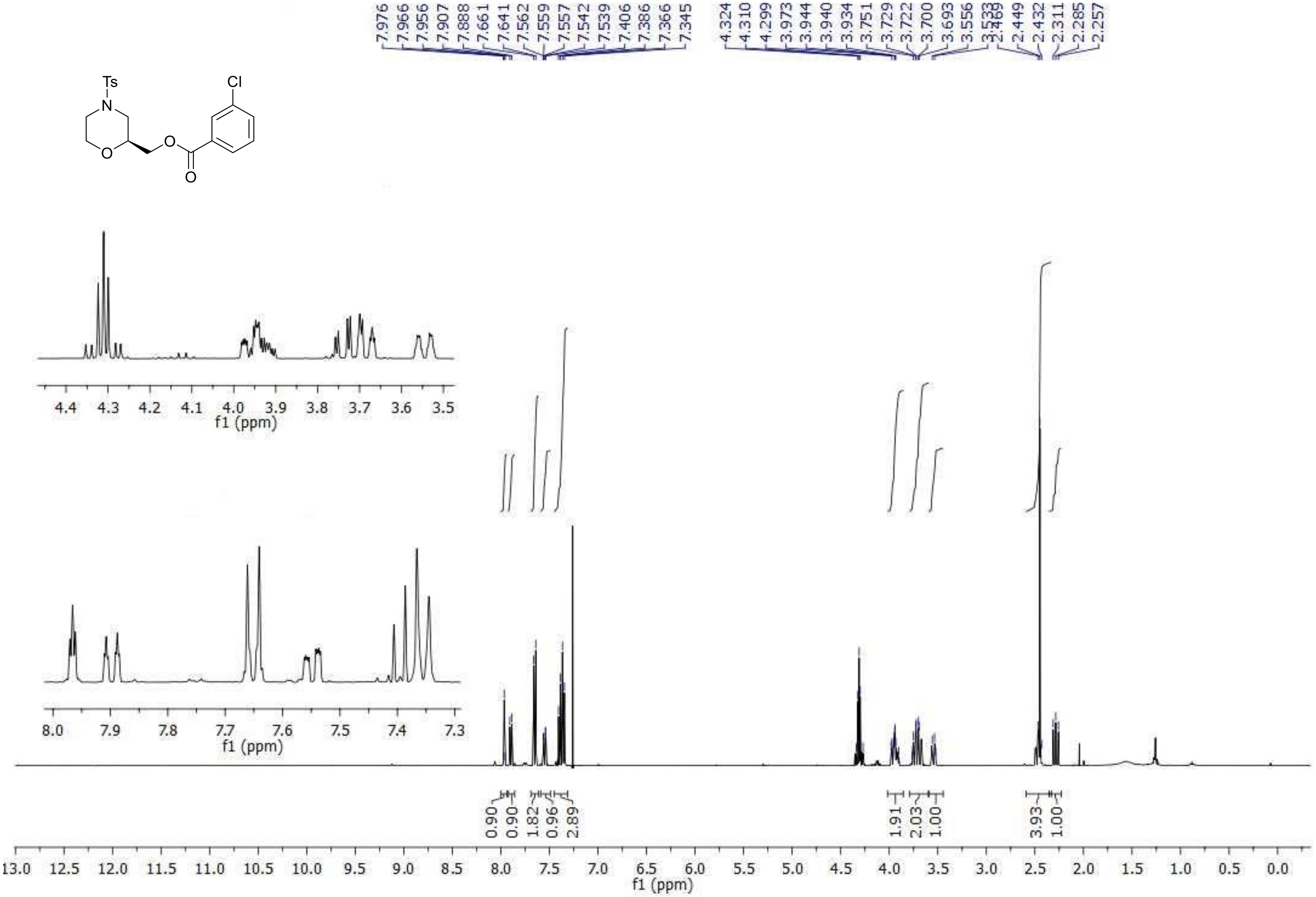

Ts
N
O
O
O
Cl
7.976 7.966 7.956 7.907 7.888 7.661 7.641 7.562 7.559 7.557 7.542 7.539 7.406 7.386 7.366 7.345
4.324 4.310 4.299 3.973 3.944 3.940 3.934 3.751 3.729 3.722 3.700 3.693 3.556 3.533 2.469 2.449 2.432 2.311 2.285 2.257
0.90 0.90 1.82 0.96 2.89
1.91 2.03 1.00
3.93 1.00
f1 (ppm)

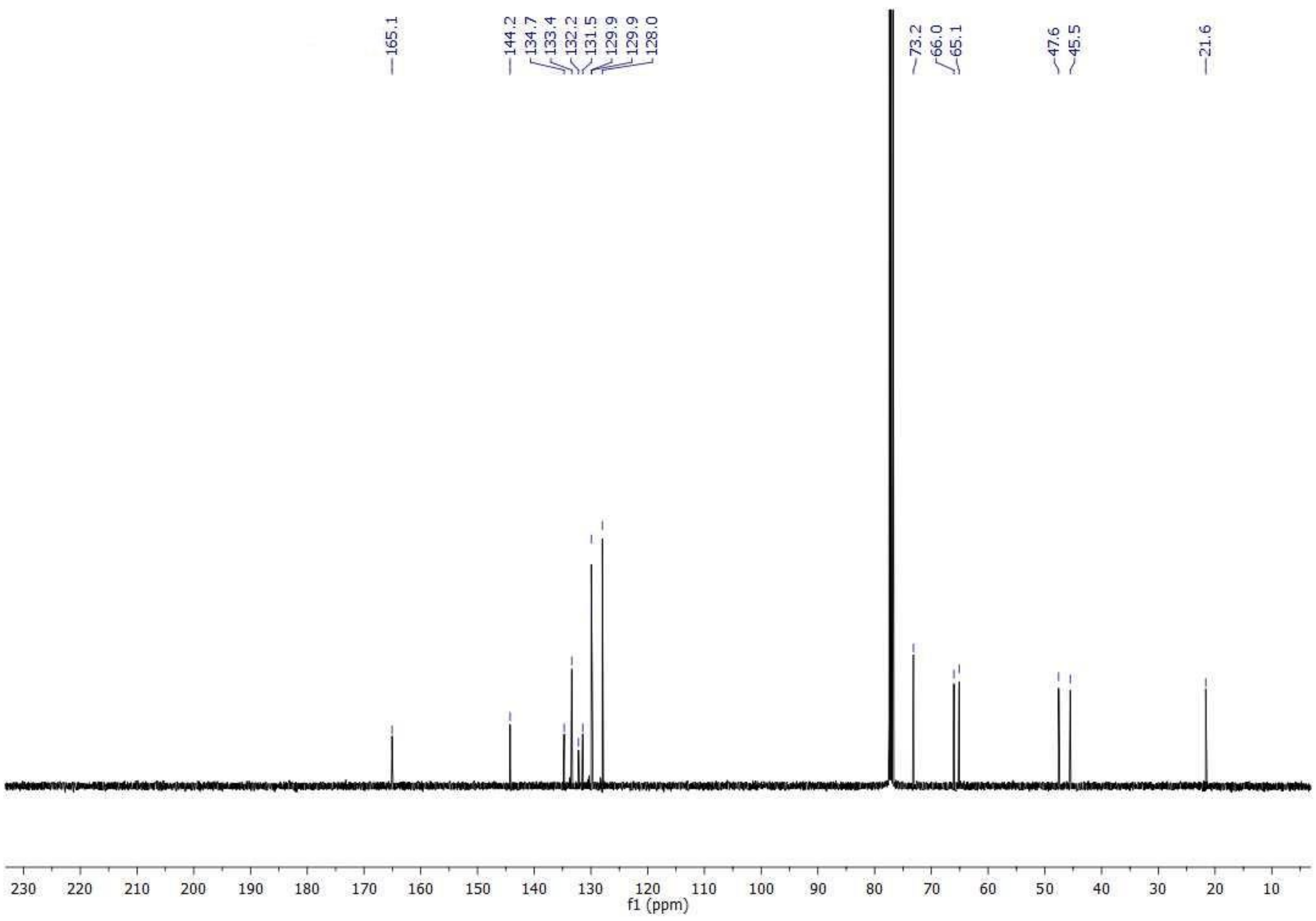
165.1
144.2
134.7
133.4
132.2
131.5
129.9
129.9
128.0
73.2
66.0
65.1
47.6
45.5
21.6
230 220 210 200 190 180 170 160 150 140 130 120 110 100 90 80 70 60 50 40 30 20 10
f1 (ppm)

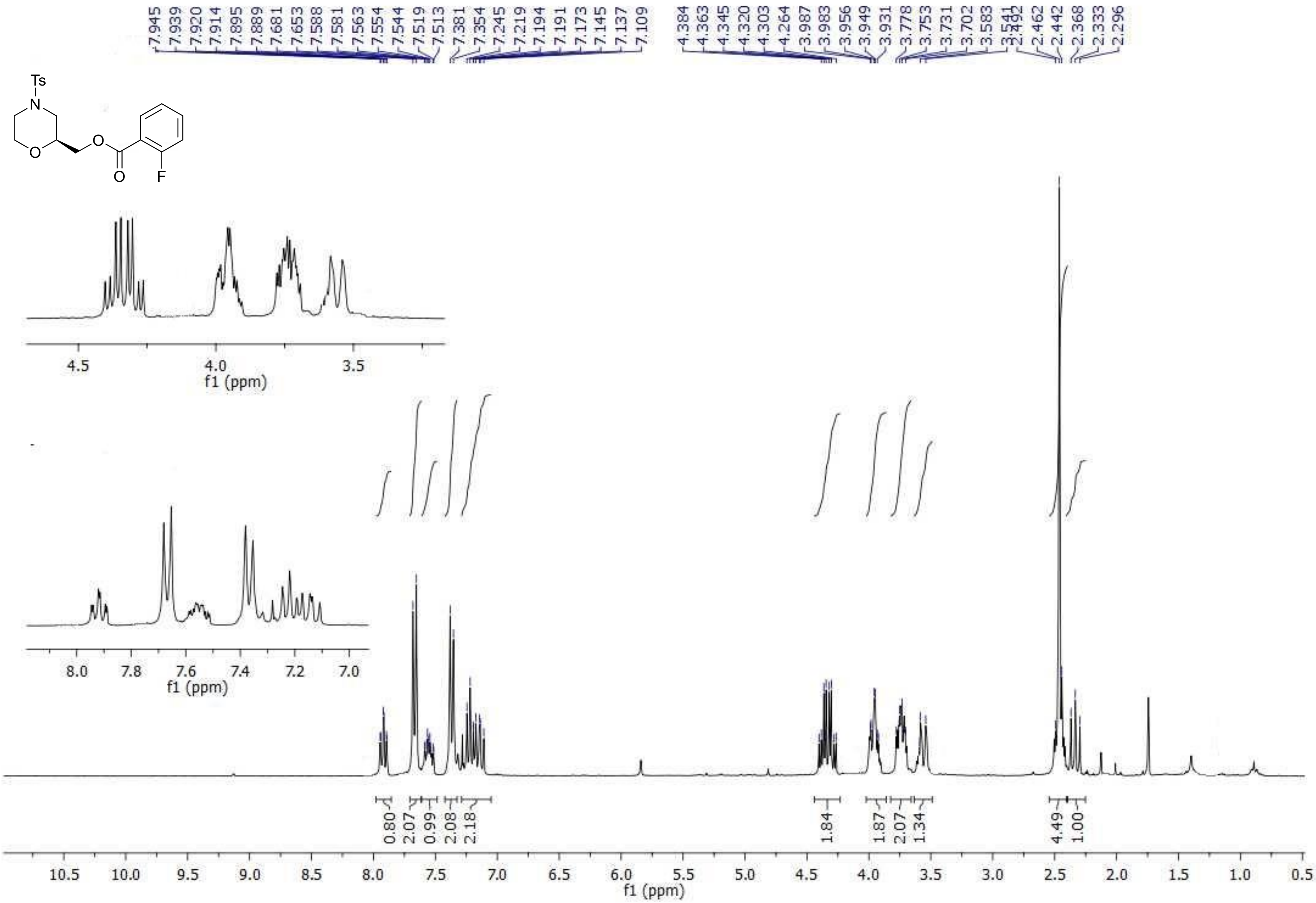

7.945 7.939 7.920 7.914 7.895 7.889 7.681 7.653 7.588 7.581 7.563 7.554 7.544 7.519 7.513 7.381 7.354 7.245 7.219 7.194 7.191 7.173 7.145 7.137 7.109
4.384 4.363 4.345 4.320 4.303 4.264 3.987 3.983 3.956 3.949 3.931 3.778 3.753 3.731 3.702 3.583 3.541 3.492 2.462 2.442 2.368 2.333 2.296
Ts
N
O
O
O
F
f1 (ppm)
0.80 2.07 0.99 2.08 2.18 1.84 1.87 2.07 1.34 4.49 1.00

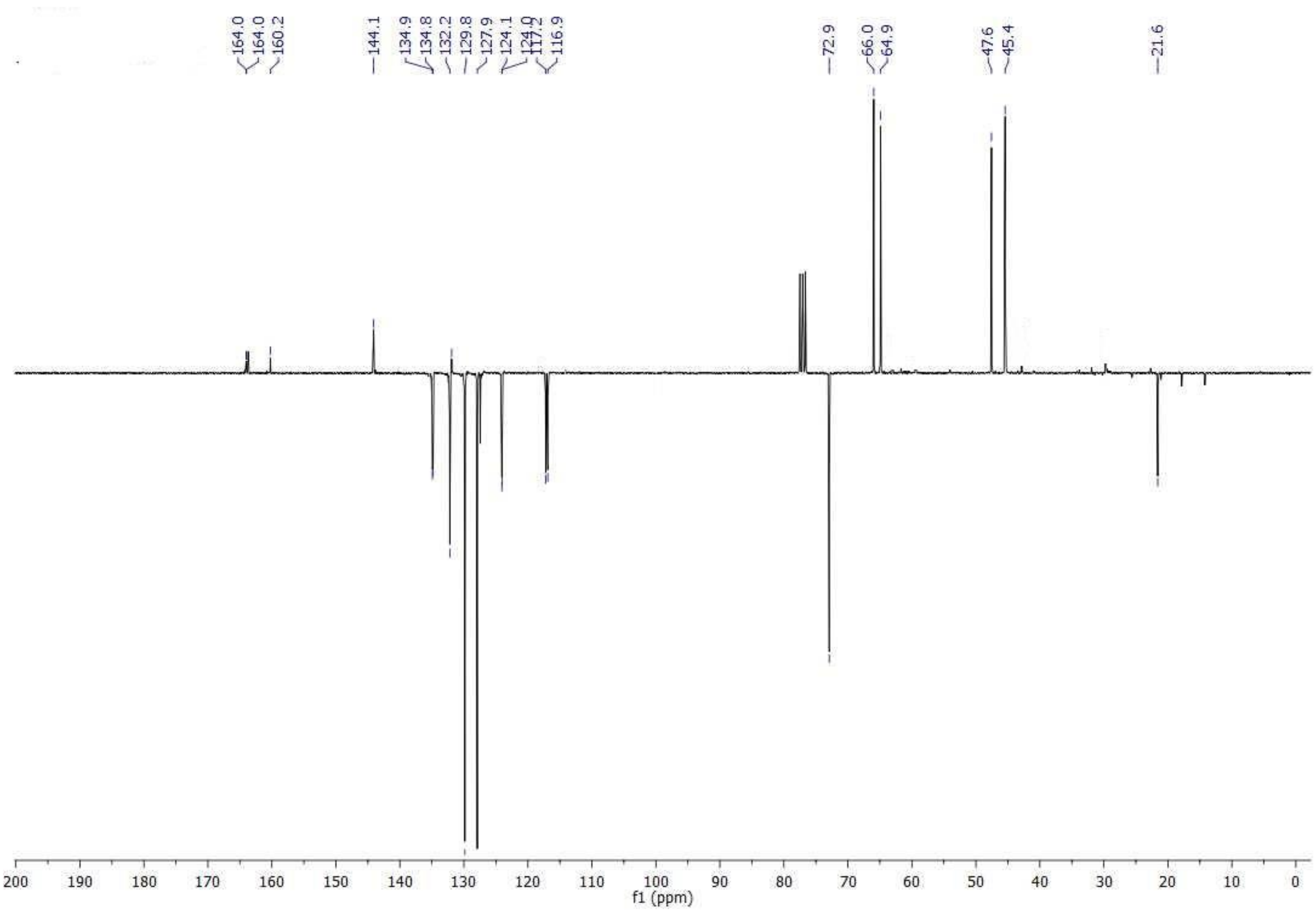

164.0
164.0
160.2
144.1
134.9
134.8
132.2
129.8
127.9
124.1
124.0
117.2
116.9
72.9
66.0
64.9
47.6
45.4
21.6
200 190 180 170 160 150 140 130 120 110 100 90 80 70 60 50 40 30 20 10 0
f1 (ppm)

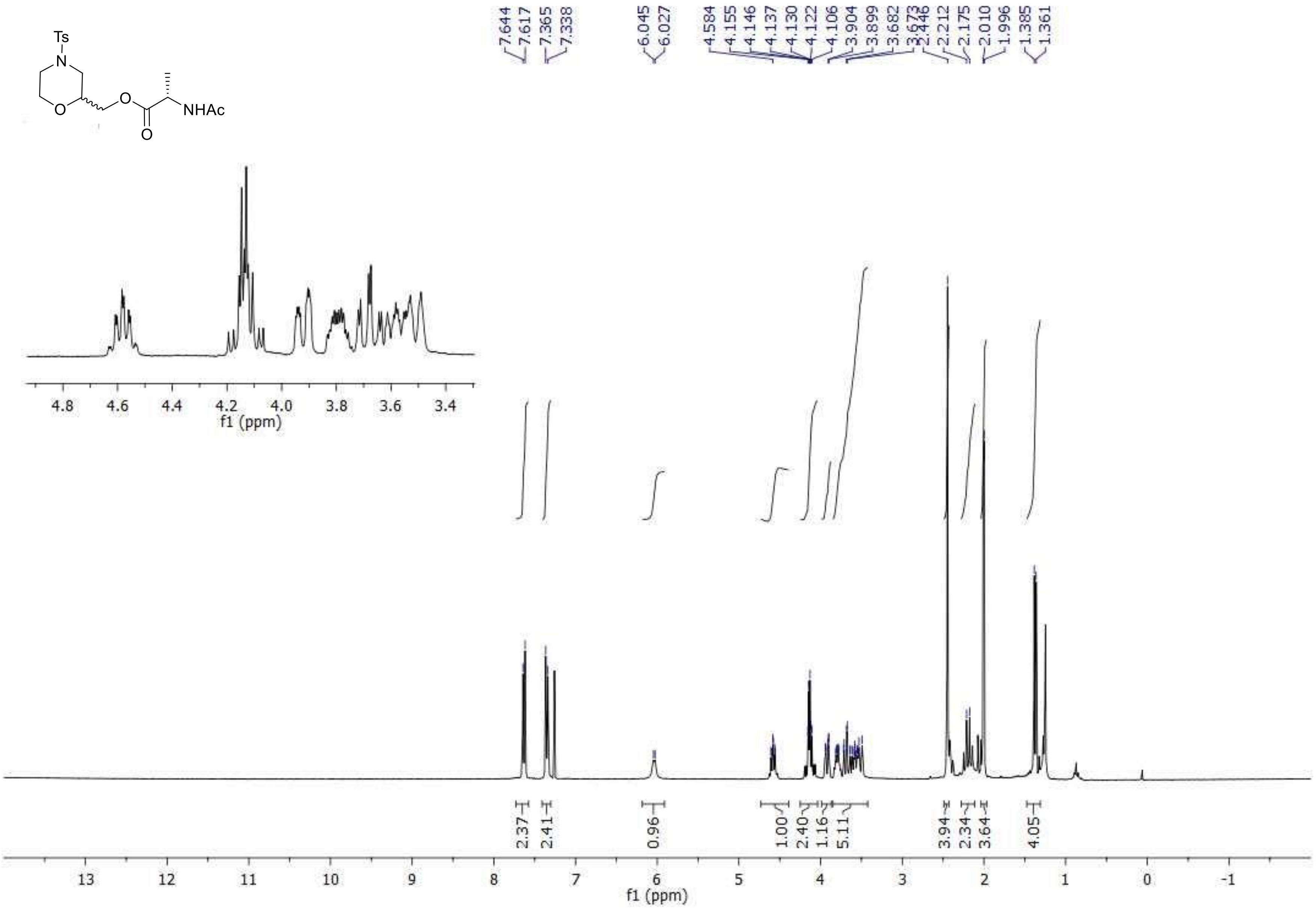

Ts
N
O
O
O
NHAc
7.644
7.617
7.365
7.338
6.045
6.027
4.584
4.155
4.146
4.137
4.130
4.122
4.106
3.904
3.899
3.682
3.673
2.446
2.212
2.175
2.010
1.996
1.385
1.361
f1 (ppm)
2.37
2.41
0.96
1.00
2.40
1.16
5.11
3.94
2.34
3.64
4.05
f1 (ppm)

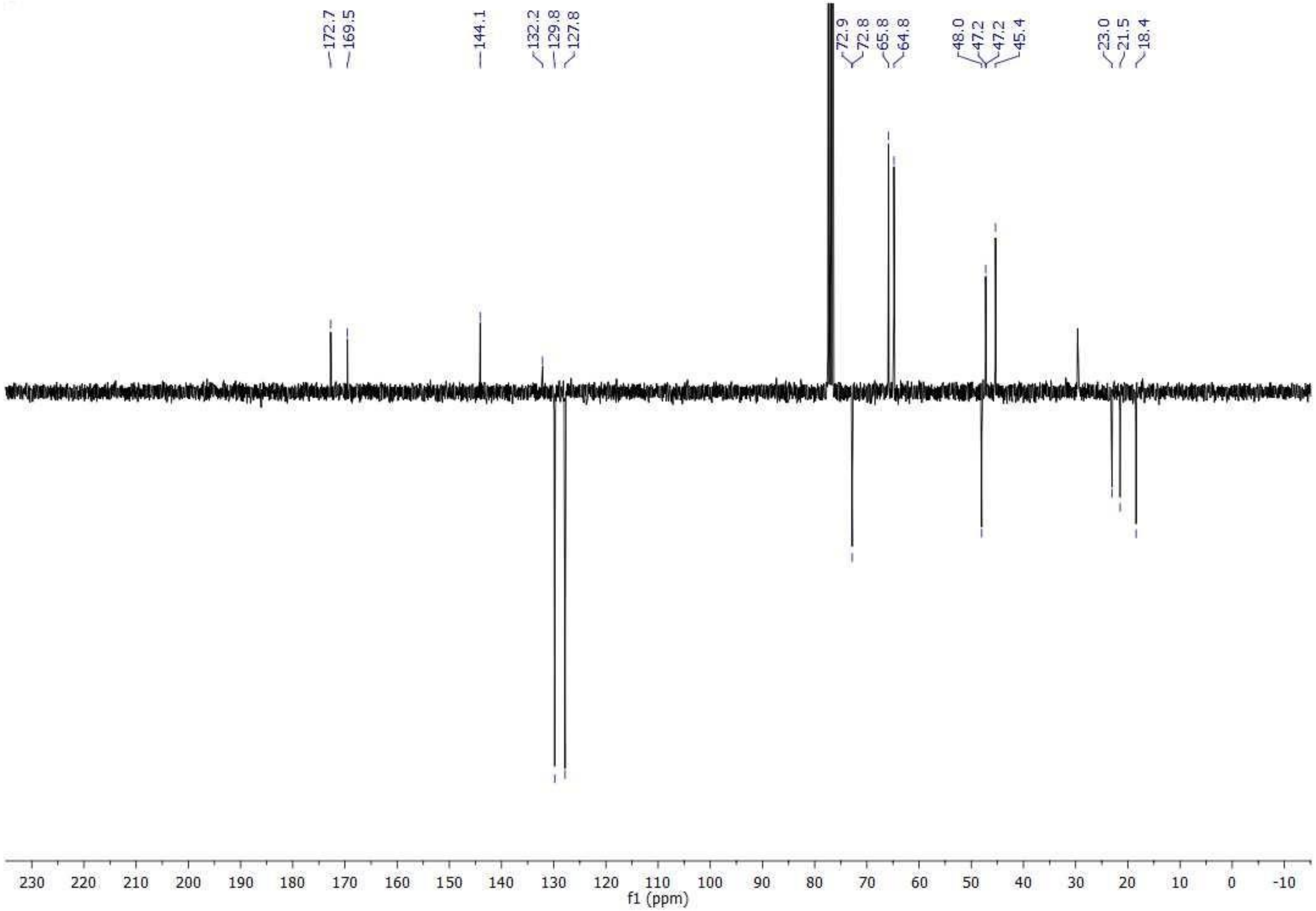

172.7
169.5
144.1
132.2
129.8
127.8
72.9
72.8
65.8
64.8
48.0
47.2
47.2
45.4
23.0
21.5
18.4
230 220 210 200 190 180 170 160 150 140 130 120 110 100 90 80 70 60 50 40 30 20 10 0 -10
f1 (ppm)

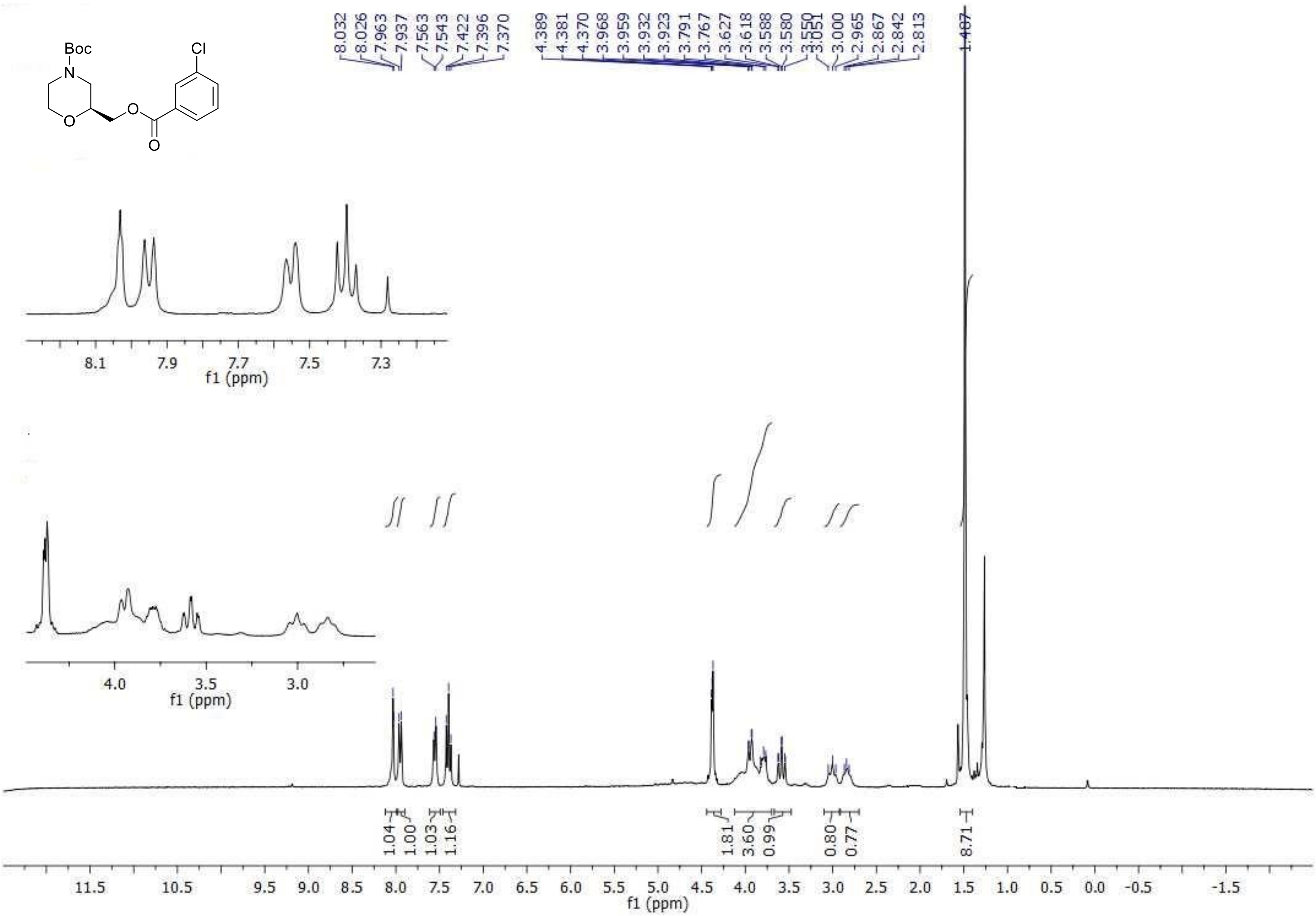

Boc
N
O
O
O
Cl
8.032
8.026
7.963
7.937
7.563
7.543
7.422
7.396
7.370
4.389
4.381
4.370
3.968
3.959
3.932
3.923
3.791
3.767
3.627
3.618
3.588
3.580
3.550
3.051
3.000
2.965
2.867
2.842
2.813
1.487
8.1
7.9
7.7
7.5
7.3
f1 (ppm)
4.0
3.5
3.0
f1 (ppm)
1.04
1.00
1.03
1.16
1.81
3.60
0.99
0.80
0.77
8.71
11.5
10.5
9.5
9.0
8.5
8.0
7.5
7.0
6.5
6.0
5.5
5.0
4.5
4.0
3.5
3.0
2.5
2.0
1.5
1.0
0.5
0.0
-0.5
-1.5
f1 (ppm)

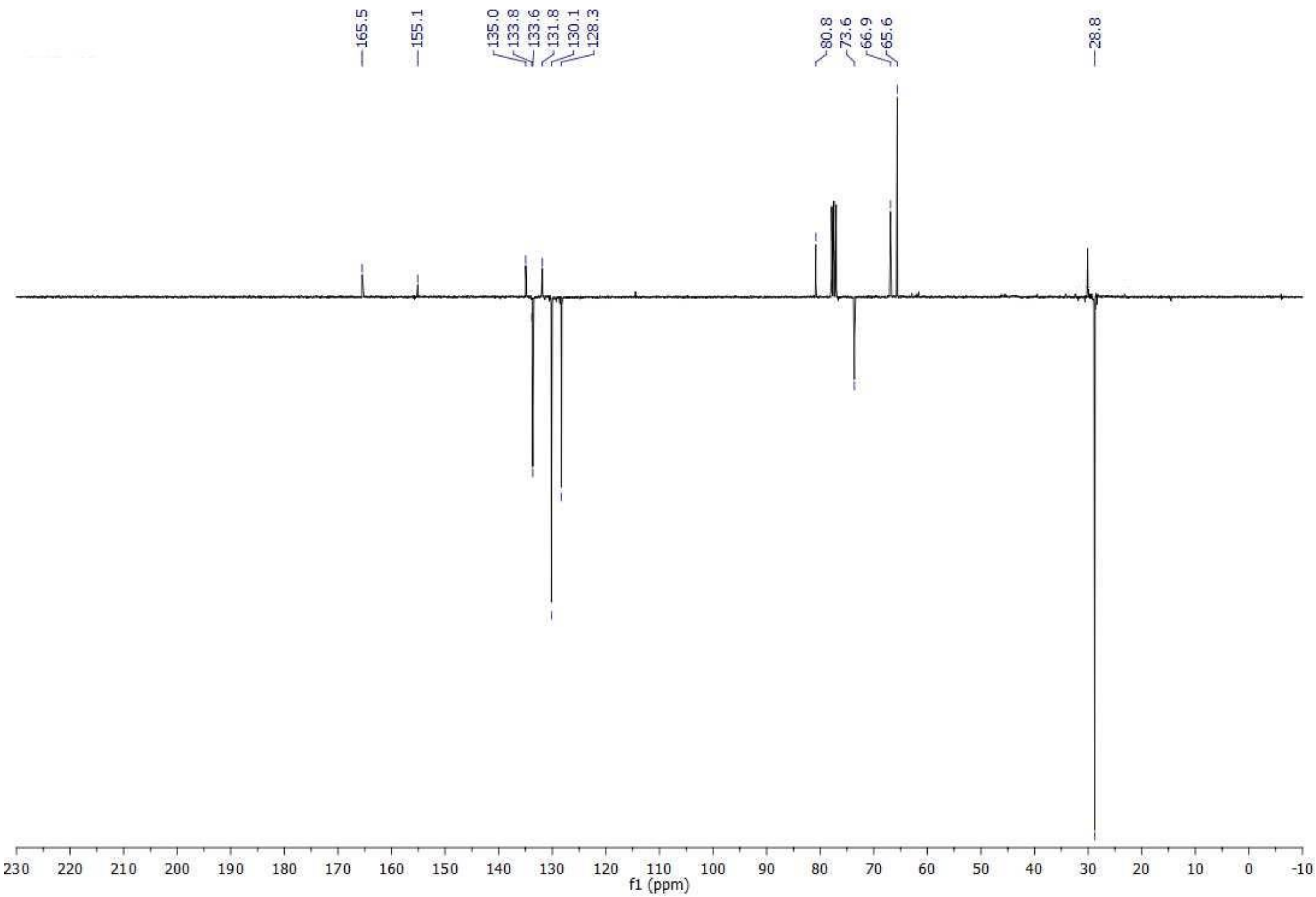
165.5
155.1
135.0
133.8
133.6
131.8
130.1
128.3
80.8
73.6
66.9
65.6
28.8
230 220 210 200 190 180 170 160 150 140 130 120 110 100 90 80 70 60 50 40 30 20 10 0 -10
f1 (ppm)

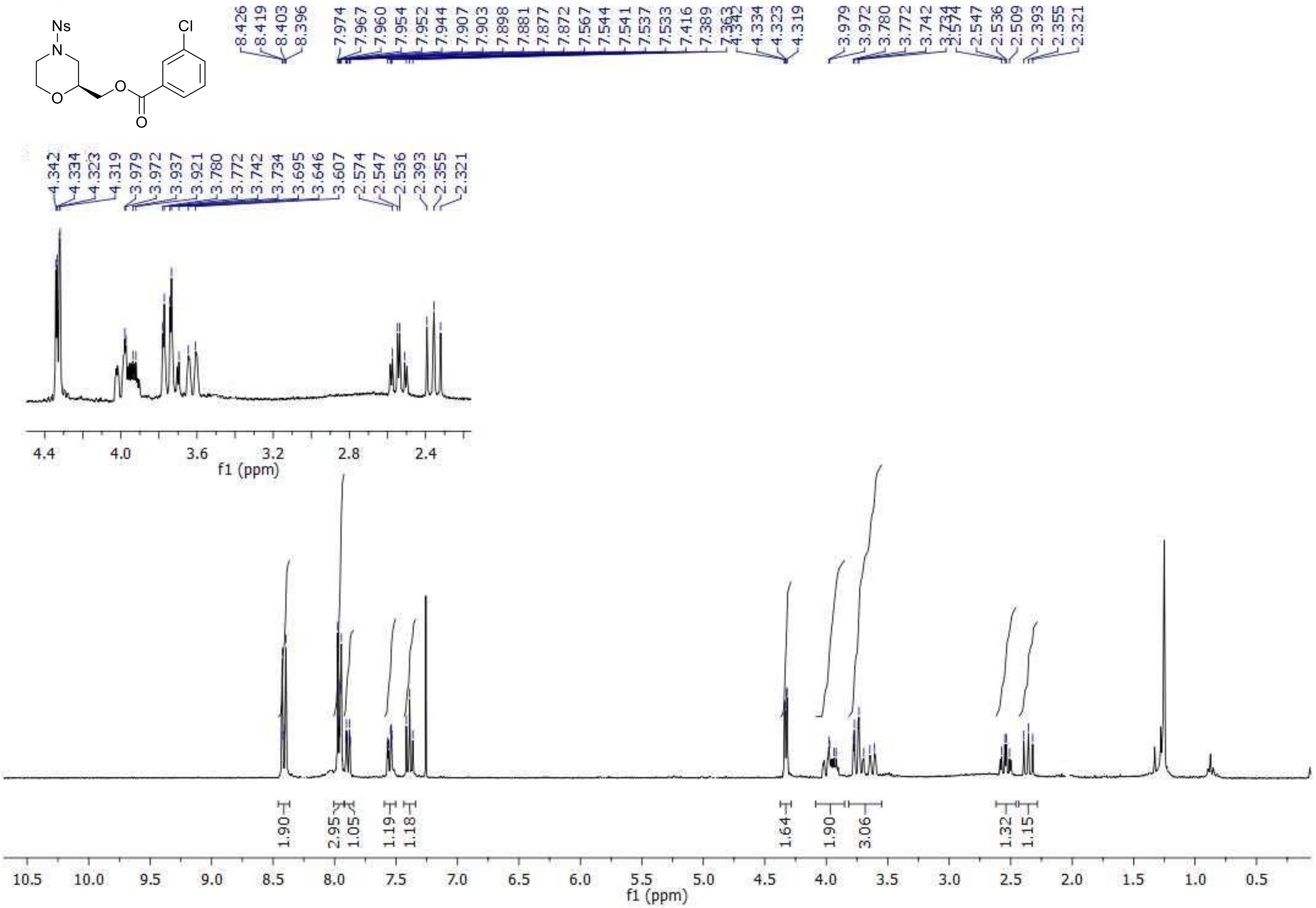
Ns
N
O
O
O
Cl
8.426
8.419
8.403
8.396
7.974
7.967
7.960
7.954
7.952
7.944
7.907
7.903
7.898
7.881
7.877
7.872
7.567
7.544
7.541
7.537
7.533
7.416
7.389
7.363
4.342
4.334
4.323
4.319
3.979
3.972
3.780
3.772
3.742
3.734
2.574
2.547
2.536
2.509
2.393
2.355
2.321
4.342
4.334
4.323
4.319
3.979
3.972
3.937
3.921
3.780
3.772
3.742
3.734
3.695
3.646
3.607
2.574
2.547
2.536
2.393
2.355
2.321
4.4
4.0
3.6
3.2
2.8
2.4
f1 (ppm)
1.90
2.95
1.05
1.19
1.18
1.64
1.90
3.06
1.32
1.15
10.5
10.0
9.5
9.0
8.5
8.0
7.5
7.0
6.5
6.0
5.5
5.0
4.5
4.0
3.5
3.0
2.5
2.0
1.5
1.0
0.5
f1 (ppm)

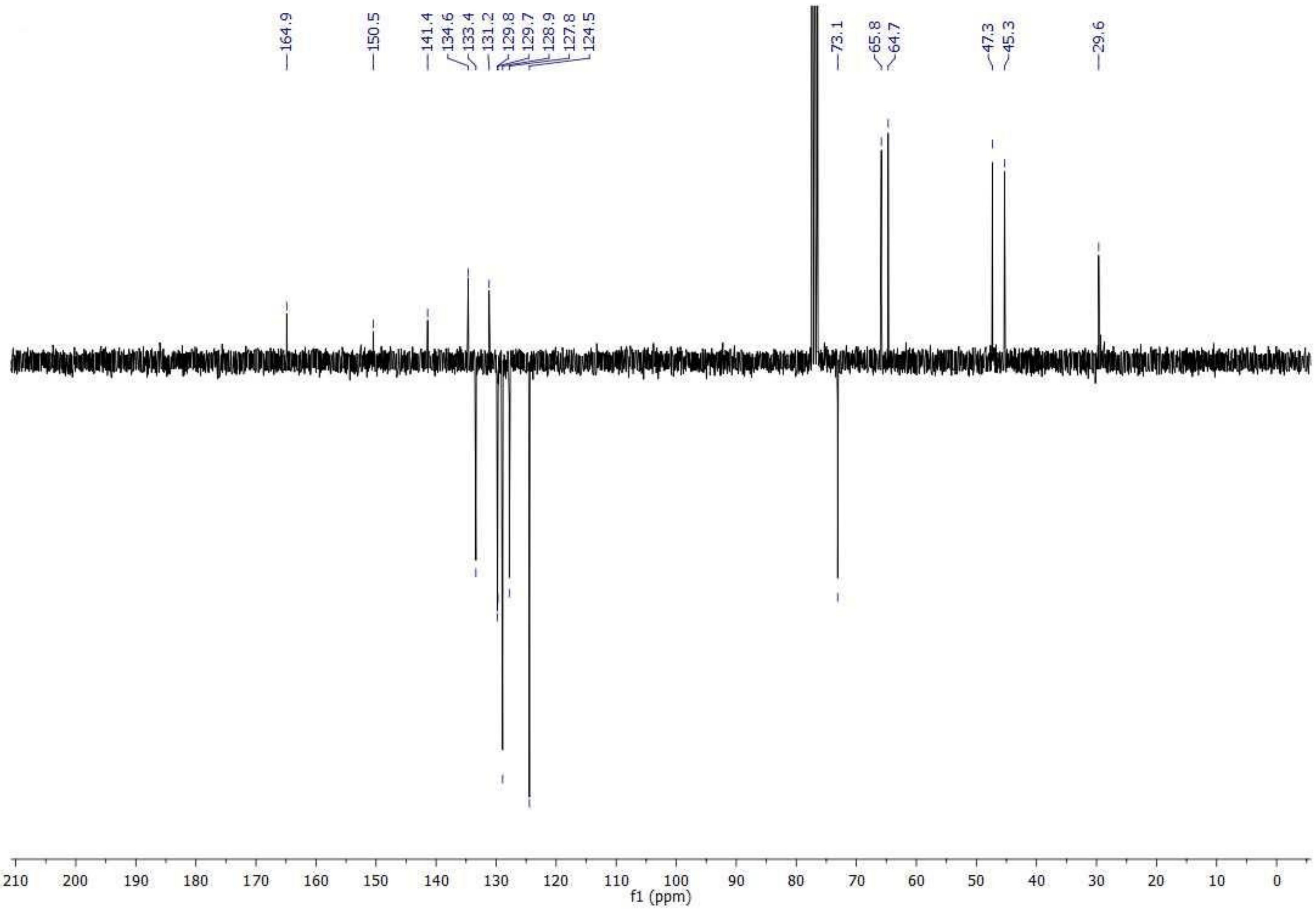

164.9
150.5
141.4
134.6
133.4
131.2
129.8
129.7
128.9
127.8
124.5
73.1
65.8
64.7
47.3
45.3
29.6
210 200 190 180 170 160 150 140 130 120 110 100 90 80 70 60 50 40 30 20 10 0
f1 (ppm)

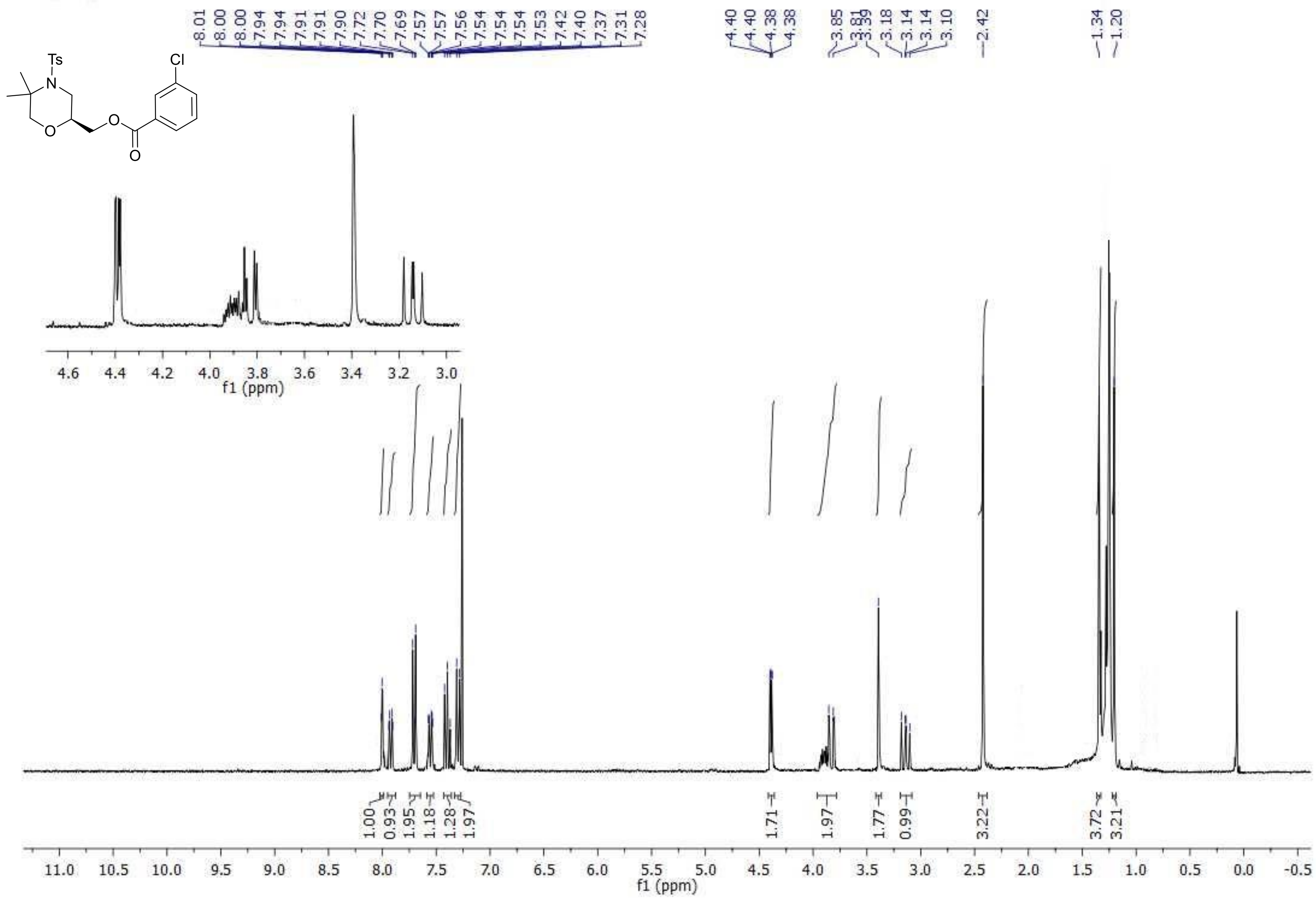

8.01
8.00
8.00
7.94
7.94
7.91
7.91
7.90
7.72
7.70
7.69
7.57
7.57
7.56
7.54
7.54
7.54
7.53
7.42
7.40
7.37
7.31
7.28
4.40
4.40
4.38
4.38
3.85
3.81
3.39
3.18
3.14
3.14
3.10
2.42
1.34
1.20
Ts
N
O
O
O
Cl
4.6 4.4 4.2 4.0 3.8 3.6 3.4 3.2 3.0
f1 (ppm)
1.00
0.93
1.95
1.18
1.28
1.97
1.71
1.97
1.77
0.99
3.22
3.72
3.21
11.0 10.5 10.0 9.5 9.0 8.5 8.0 7.5 7.0 6.5 6.0 5.5 5.0 4.5 4.0 3.5 3.0 2.5 2.0 1.5 1.0 0.5 0.0 -0.5
f1 (ppm)

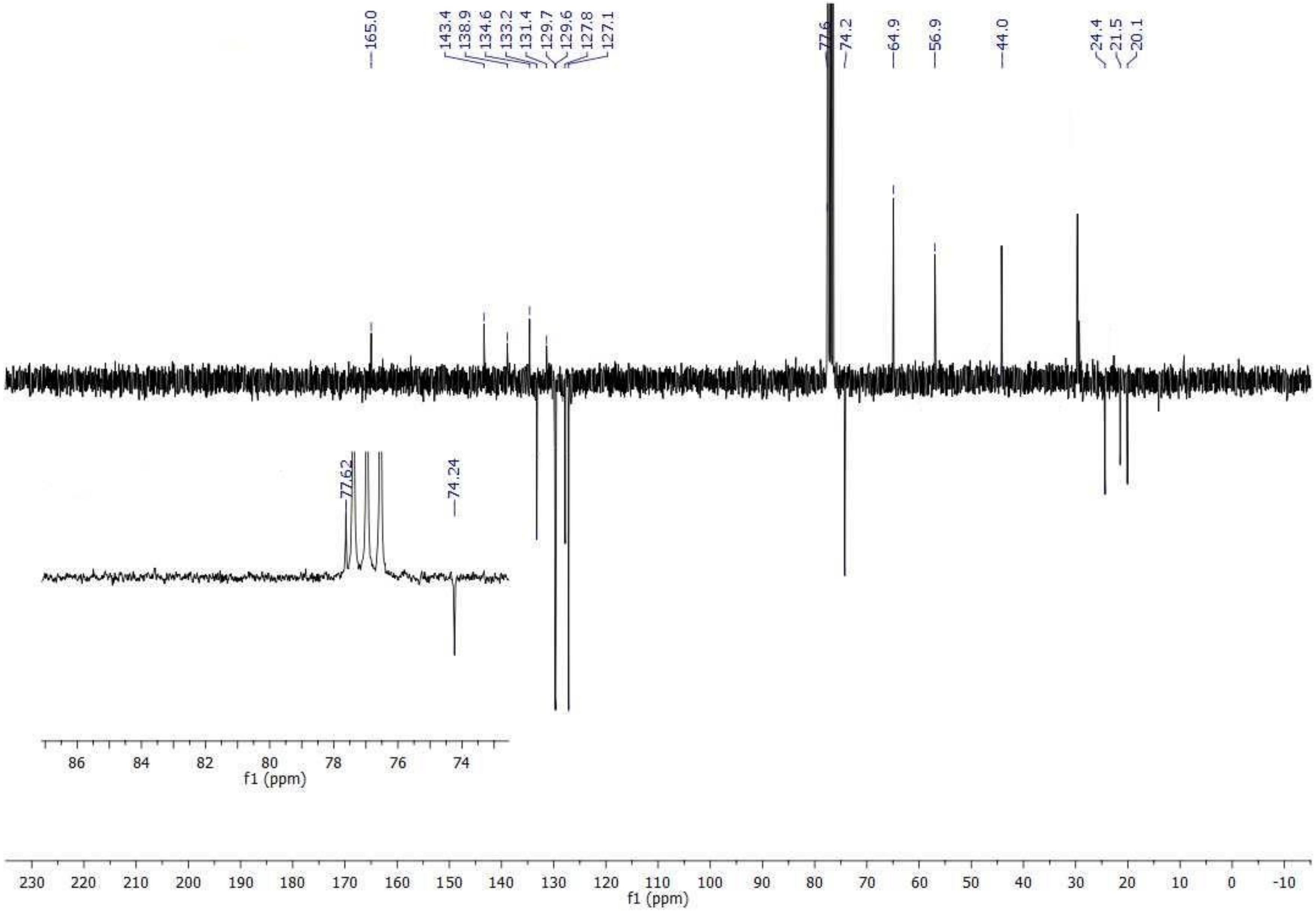

165.0
143.4
138.9
134.6
133.2
131.4
129.7
129.6
127.8
127.1
77.6
74.2
64.9
56.9
44.0
24.4
21.5
20.1
77.62
74.24
86 84 82 80 78 76 74
f1 (ppm)
230 220 210 200 190 180 170 160 150 140 130 120 110 100 90 80 70 60 50 40 30 20 10 0 -10
f1 (ppm)

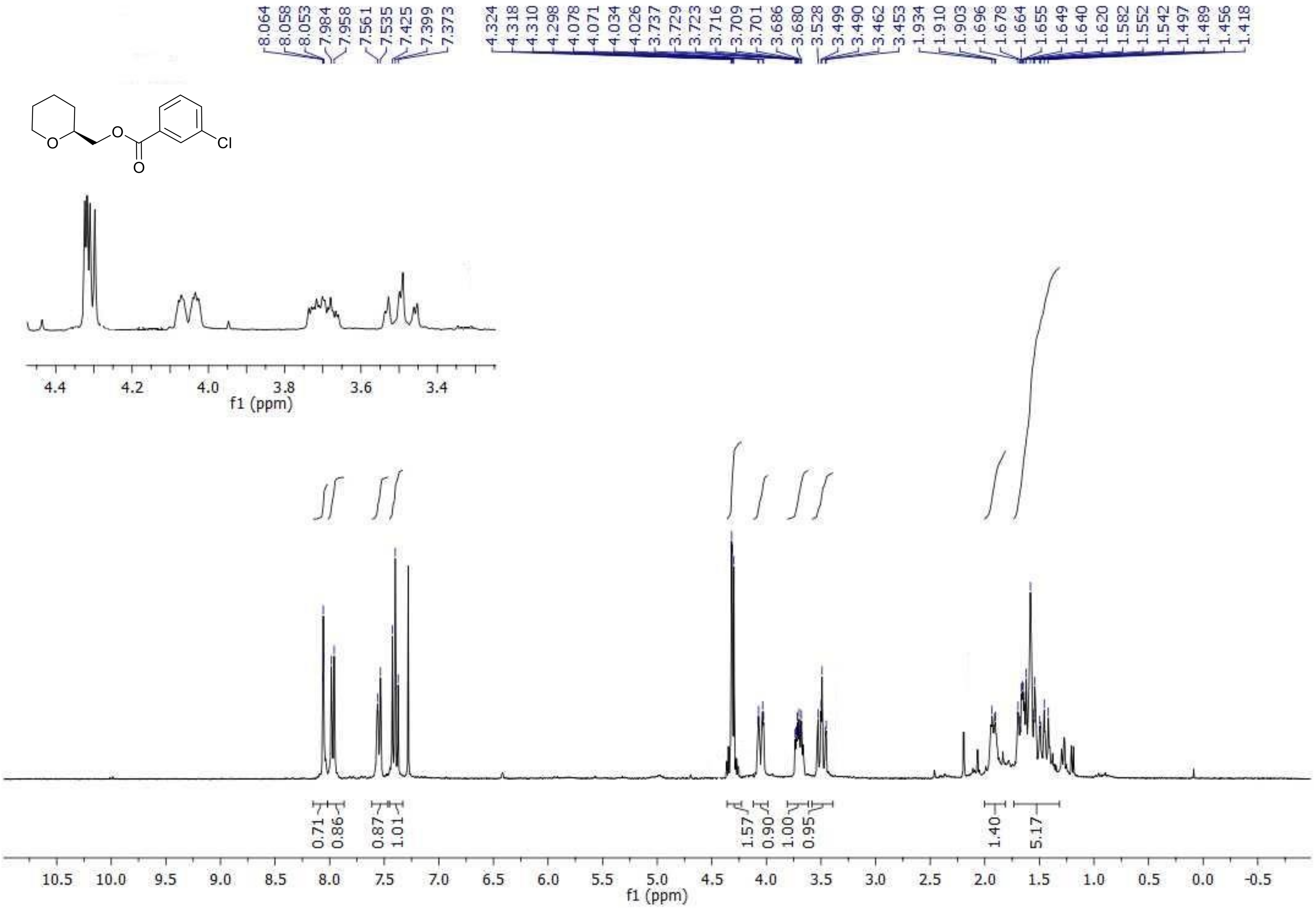

Cl
O
f1 (ppm)

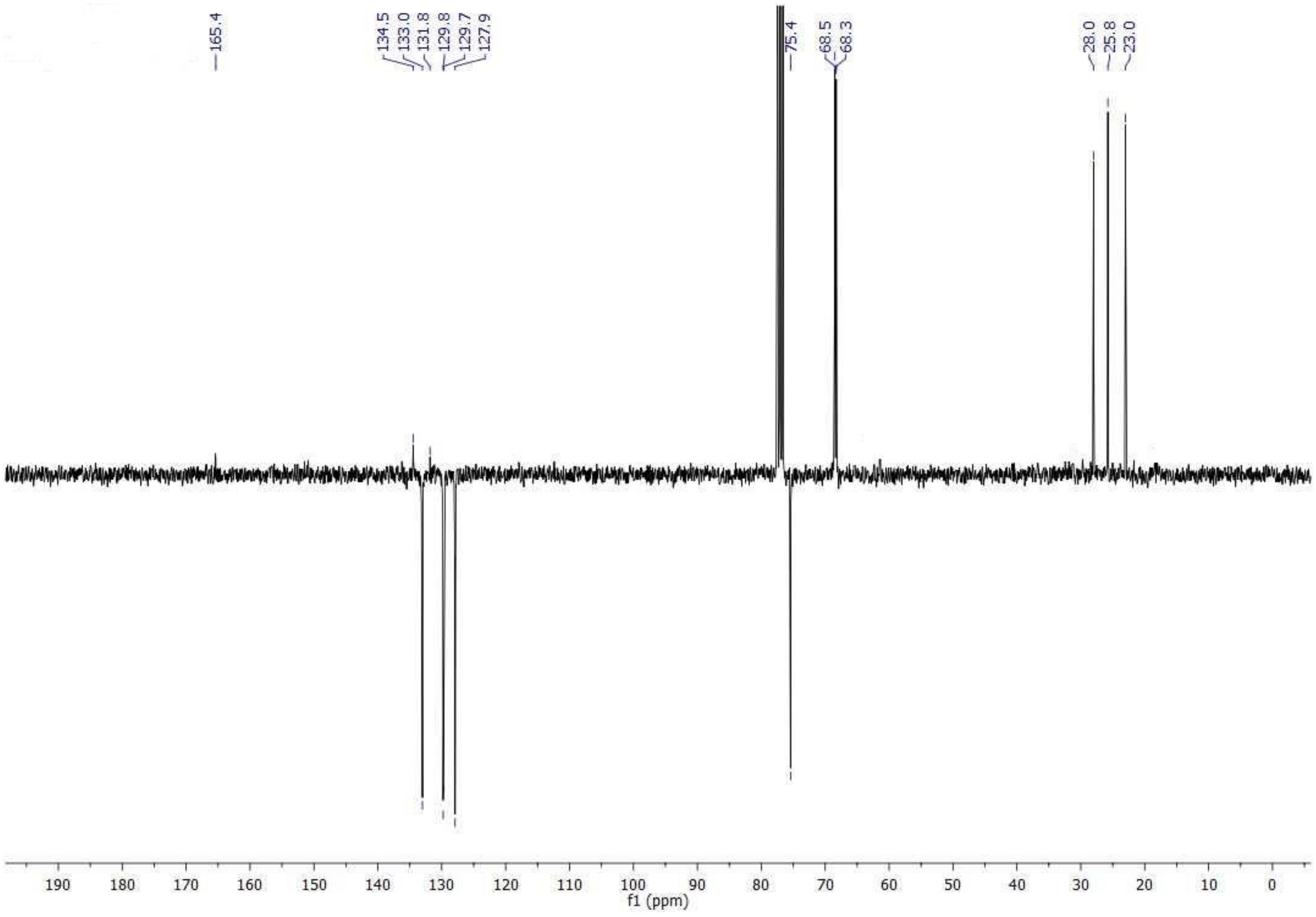

165.4
134.5
133.0
131.8
129.8
129.7
127.9
75.4
68.5
68.3
28.0
25.8
23.0
190
180
170
160
150
140
130
120
110
100
90
80
70
60
50
40
30
20
10
0
f1 (ppm)

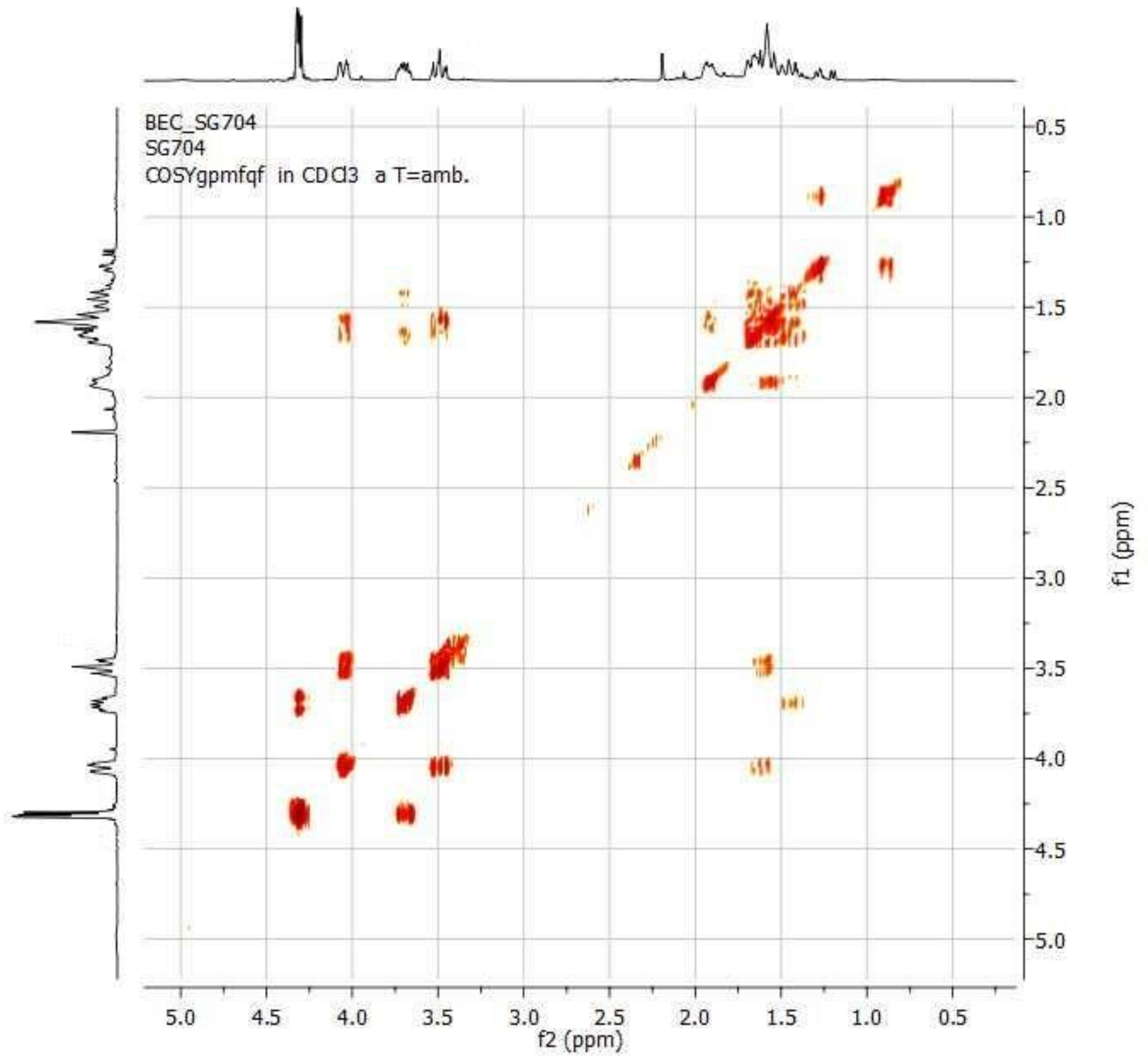
BEC_SG704
SG704
COSYgpmfqf in CDCl3 a T=amb.
f2 (ppm)
f1 (ppm)
5.0
4.5
4.0
3.5
3.0
2.5
2.0
1.5
1.0
0.5

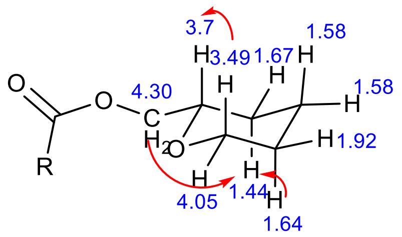
3.7
H3.49
1.67
H
1.58
H
1.58
H
O
O
4.30
C
H2O
H1.92
R
H
4.05
H
1.44
H
1.64

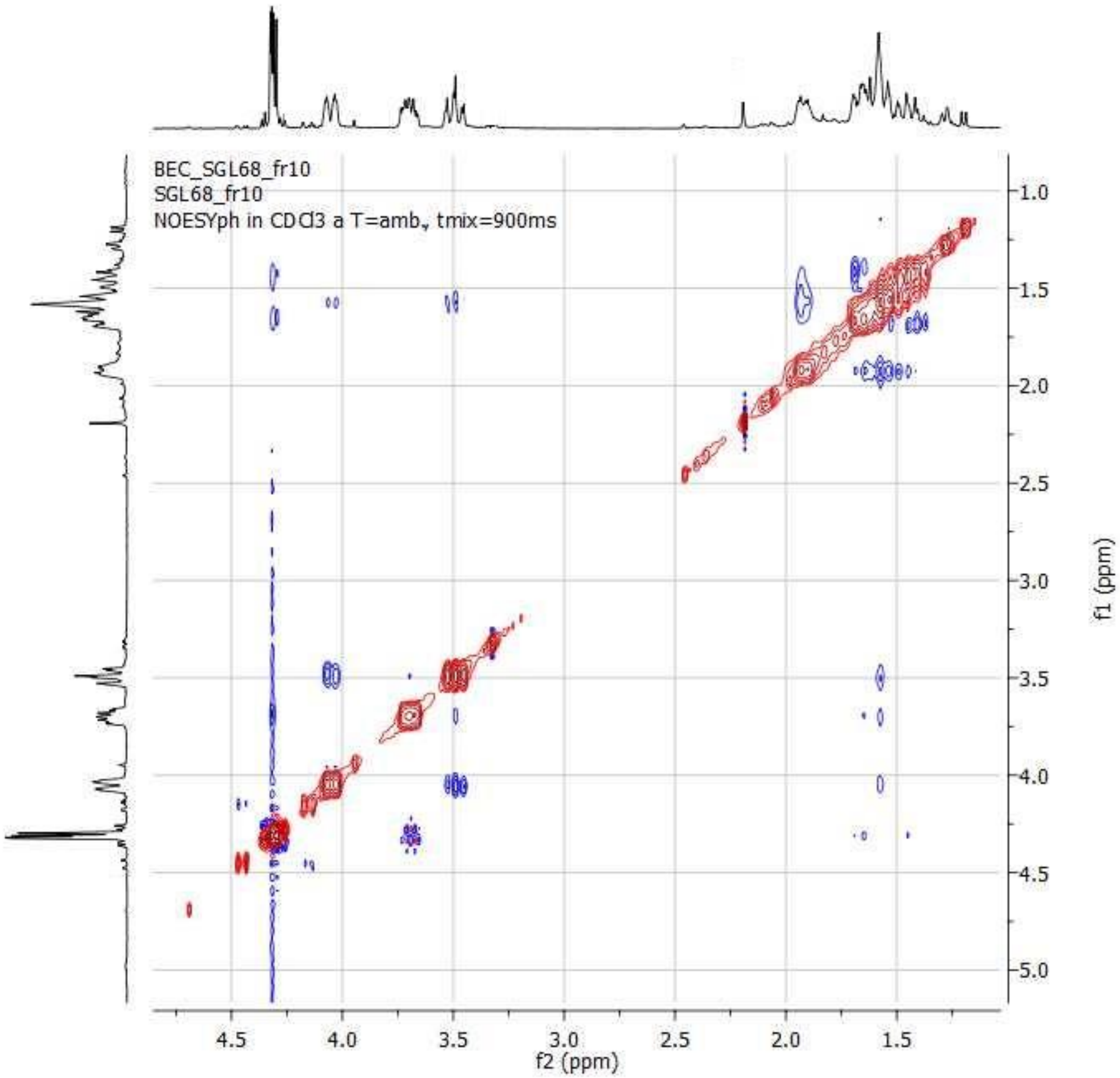
BEC_SGL68_fr10
SGL68_fr10
NOESYph in CDCl3 a T=amb., tmix=900ms
f2 (ppm)
f1 (ppm)

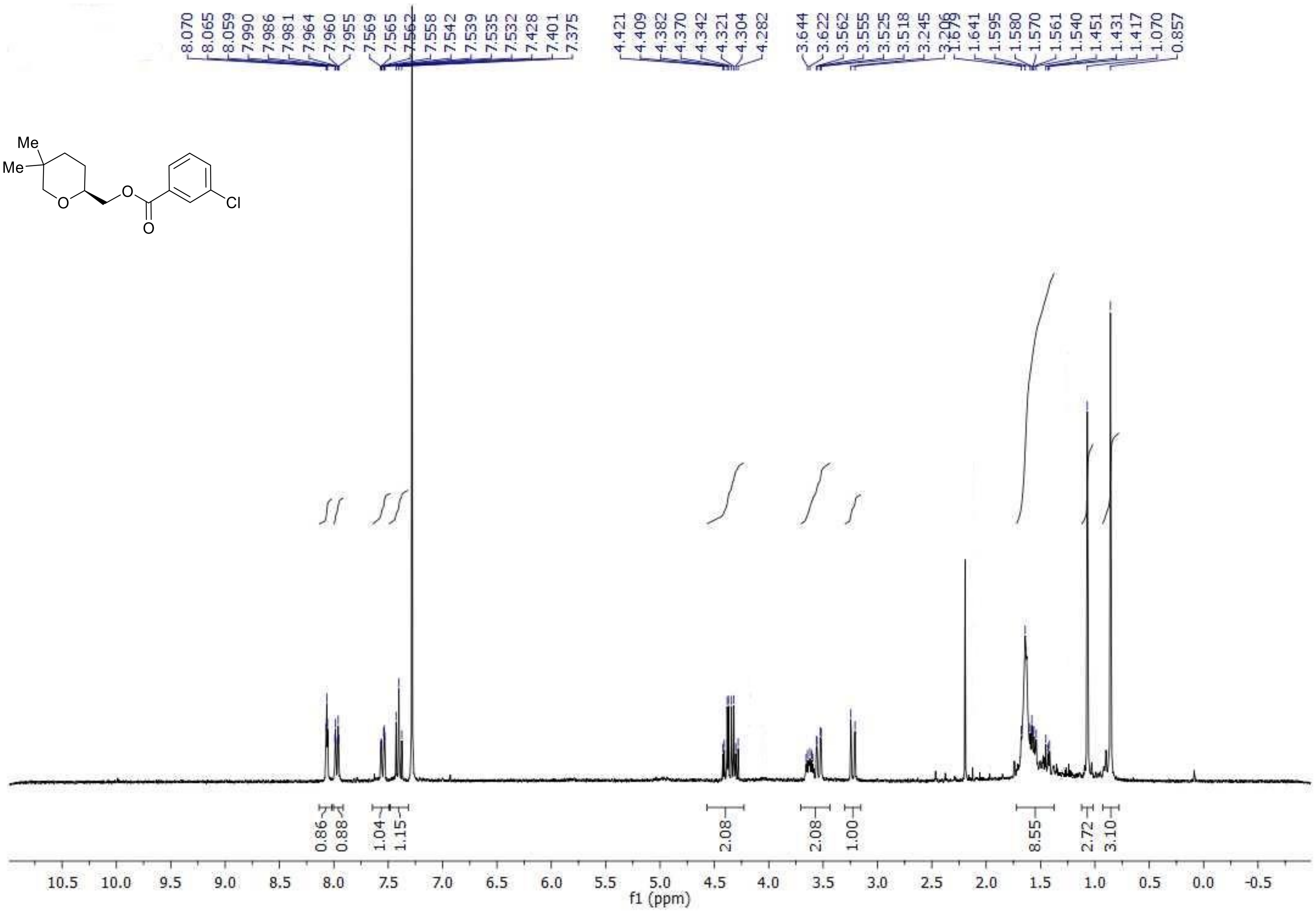

8.070
8.065
8.059
7.990
7.986
7.981
7.964
7.960
7.955
7.569
7.565
7.562
7.558
7.542
7.539
7.535
7.532
7.428
7.401
7.375
4.421
4.409
4.382
4.370
4.342
4.321
4.304
4.282
3.644
3.622
3.562
3.555
3.525
3.518
3.245
3.206
1.679
1.641
1.595
1.580
1.570
1.561
1.540
1.451
1.431
1.417
1.070
0.857
Me
Me
O
O
O
Cl
0.86
0.88
1.04
1.15
2.08
2.08
1.00
8.55
2.72
3.10
10.5
10.0
9.5
9.0
8.5
8.0
7.5
7.0
6.5
6.0
5.5
5.0
4.5
4.0
3.5
3.0
2.5
2.0
1.5
1.0
0.5
0.0
-0.5
f1 (ppm)

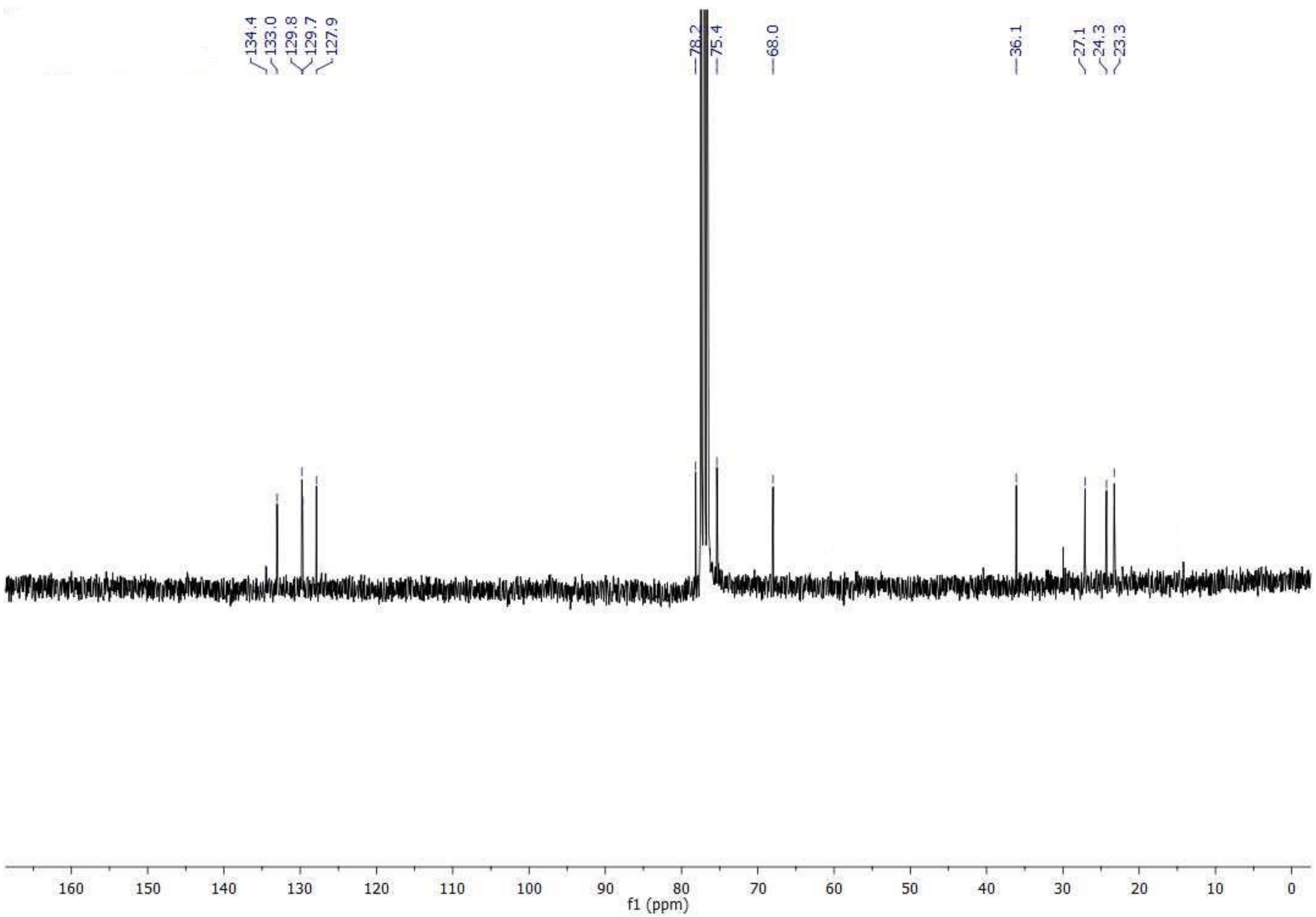
134.4
133.0
129.8
129.7
127.9
78.2
75.4
68.0
36.1
27.1
24.3
23.3
160
150
140
130
120
110
100
90
80
70
60
50
40
30
20
10
0
f1 (ppm)

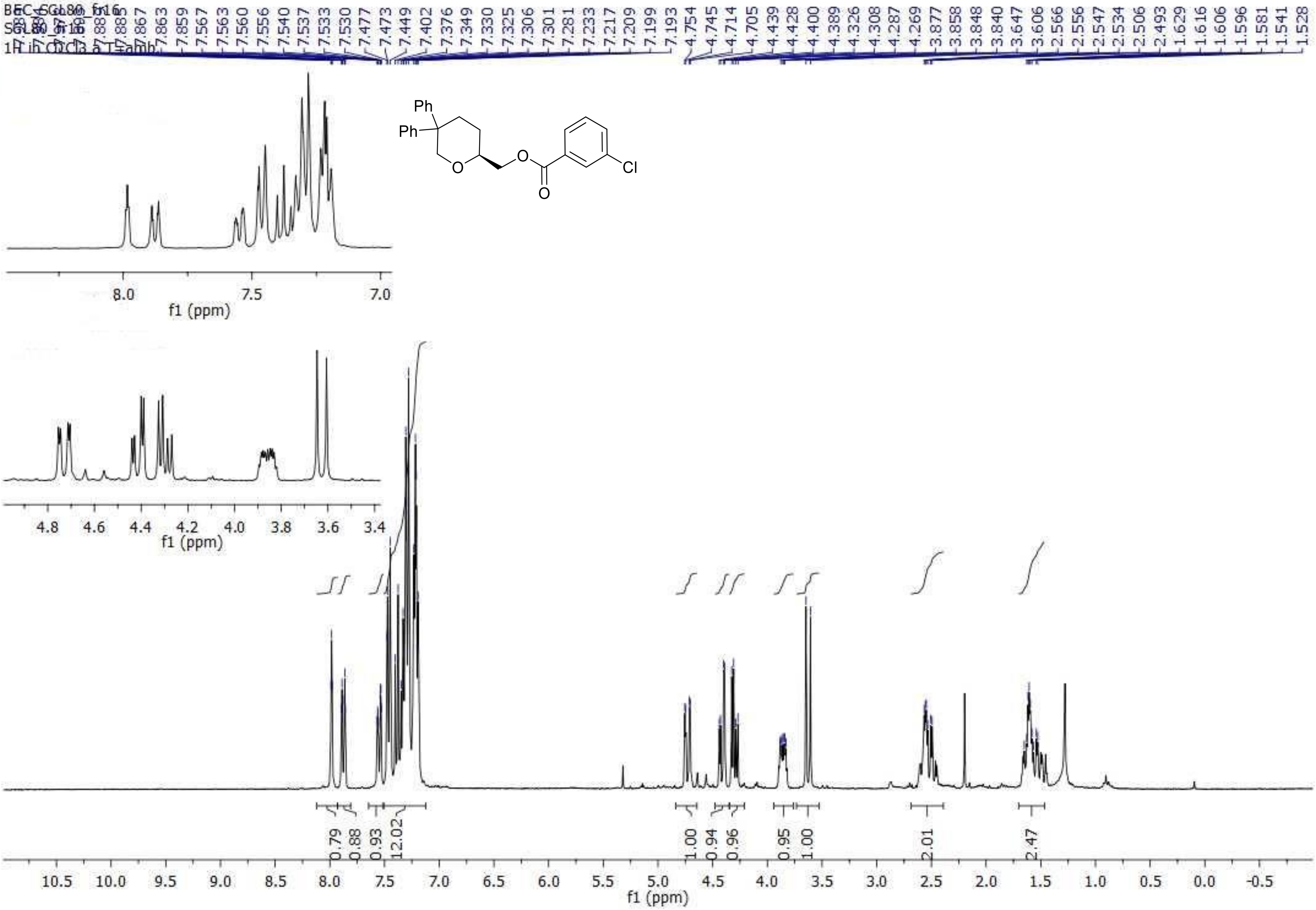

SGL80_fr16
7.867 7.863 7.859 7.567 7.563 7.560 7.556 7.540 7.537 7.533 7.530 7.477 7.473 7.449 7.402 7.376 7.349 7.330 7.325 7.306 7.301 7.281 7.233 7.217 7.209 7.199 7.193
4.754 4.745 4.714 4.705 4.439 4.428 4.400 4.389 4.326 4.308 4.287 4.269 3.877 3.858 3.848 3.840 3.647 3.606 2.566 2.556 2.547 2.534 2.506 2.493 1.629 1.616 1.606 1.596 1.581 1.541 1.528
Ph
Ph
O
O
O
Cl
f1 (ppm)
f1 (ppm)
0.79
0.88
0.93
12.02
1.00
0.94
0.96
0.95
1.00
2.01
2.47
f1 (ppm)

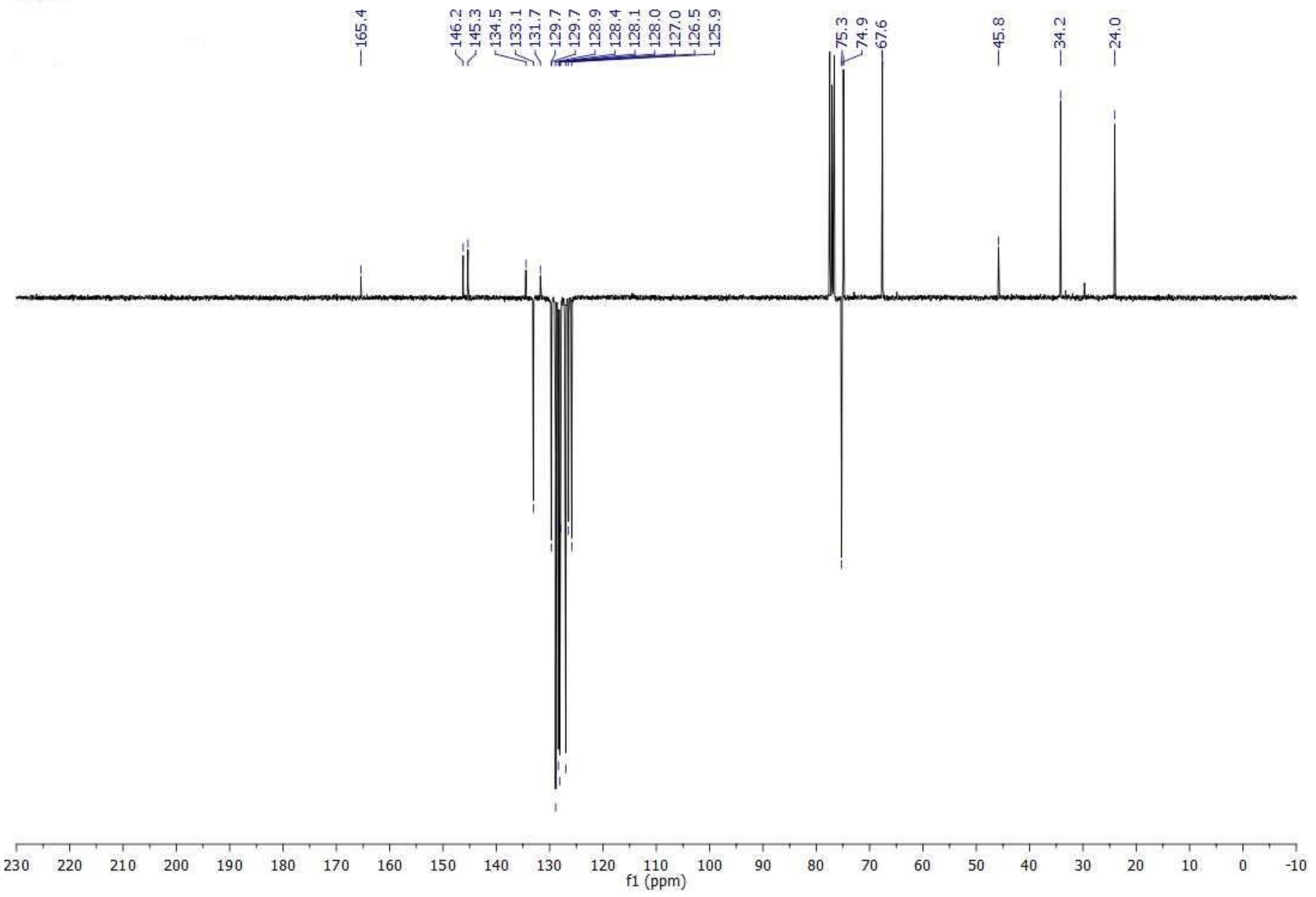

165.4
146.2
145.3
134.5
133.1
131.7
129.7
129.7
128.9
128.4
128.1
128.0
127.0
126.5
125.9
75.3
74.9
67.6
45.8
34.2
24.0
230 220 210 200 190 180 170 160 150 140 130 120 110 100 90 80 70 60 50 40 30 20 10 0 -10
f1 (ppm)

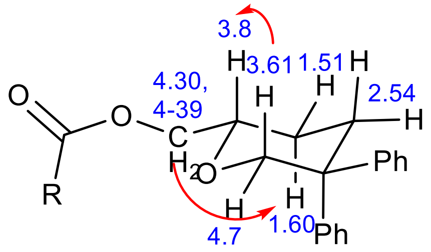
3.8
3.61
1.51
4.30,
4-39
2.54
Ph
4.7
1.60
Ph
R

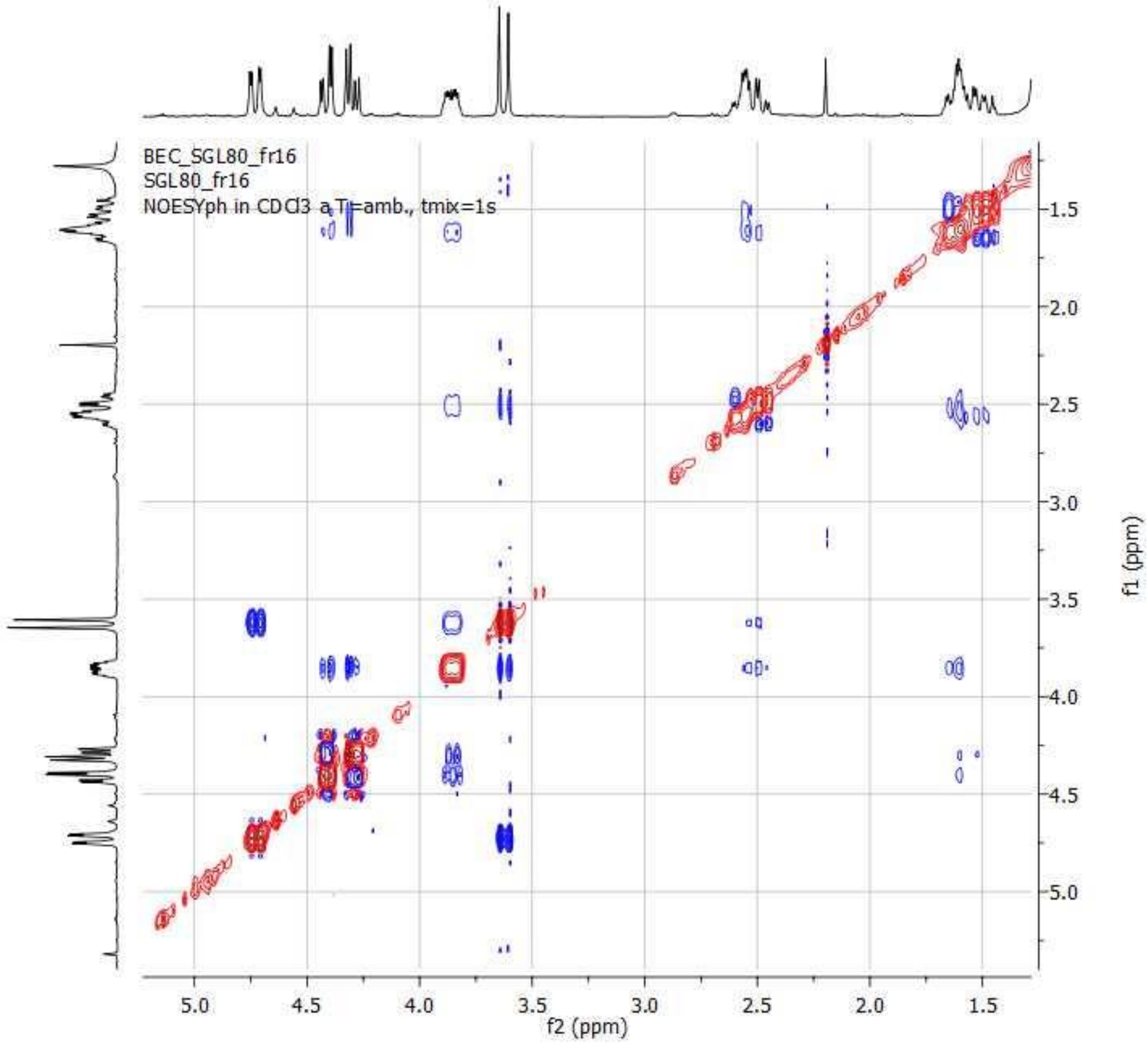
BEC_SGL80_fr16
SGL80_fr16
NOESYph in CDCl3 at T=amb., tmix=1s
f2 (ppm)
f1 (ppm)

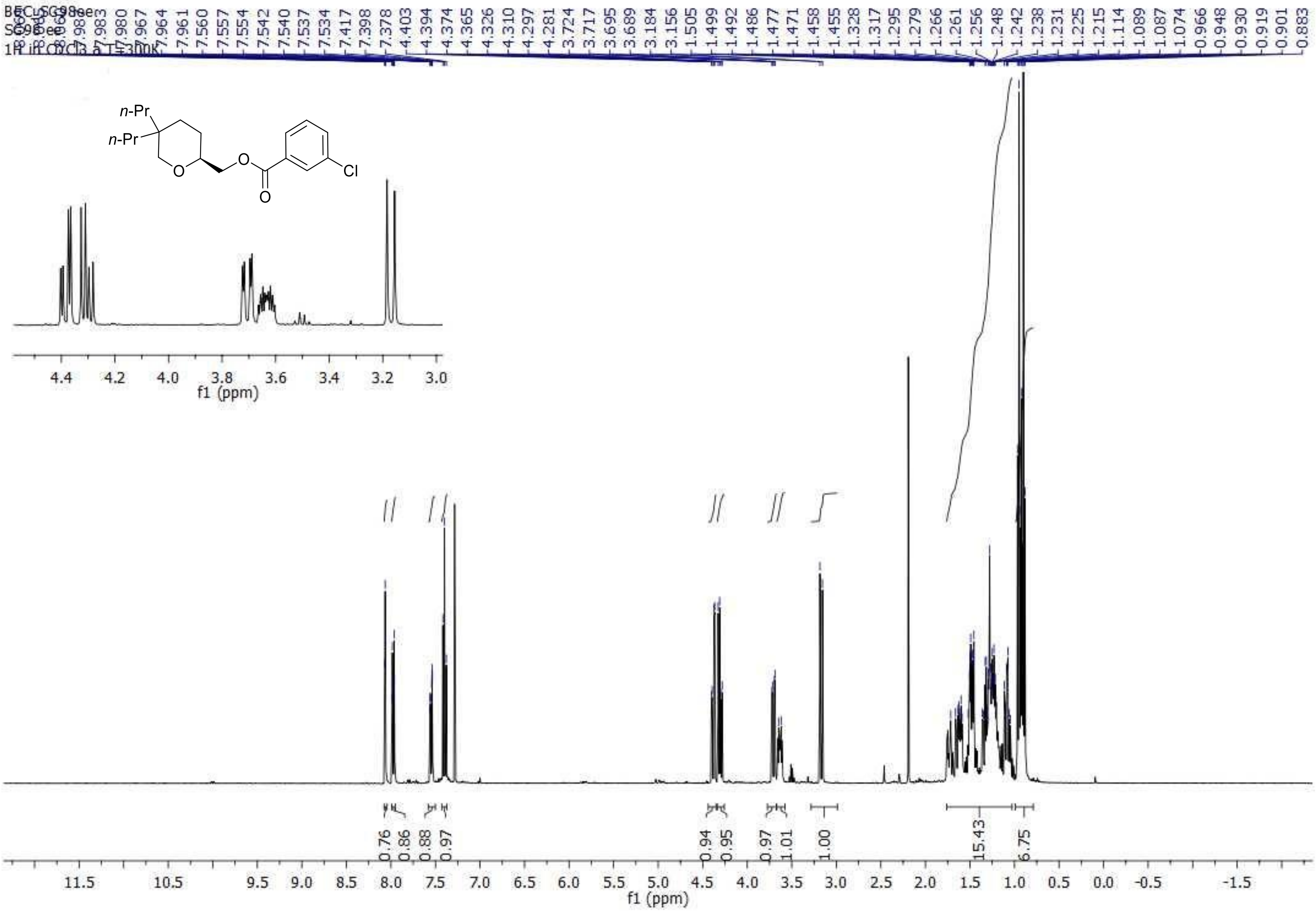

SG98ee
7.983
7.980
7.967
7.964
7.961
7.560
7.557
7.554
7.542
7.540
7.537
7.534
7.417
7.398
7.378
4.403
4.394
4.374
4.365
4.326
4.310
4.297
4.281
3.724
3.717
3.695
3.689
3.184
3.156
1.505
1.499
1.492
1.486
1.477
1.471
1.458
1.455
1.328
1.317
1.295
1.279
1.266
1.261
1.256
1.248
1.242
1.238
1.231
1.225
1.215
1.114
1.089
1.087
1.074
0.966
0.948
0.930
0.919
0.901
0.883
n-Pr
n-Pr
O
O
O
Cl
f1 (ppm)
0.76
0.86
0.88
0.97
0.94
0.95
0.97
1.01
1.00
15.43
6.75
f1 (ppm)

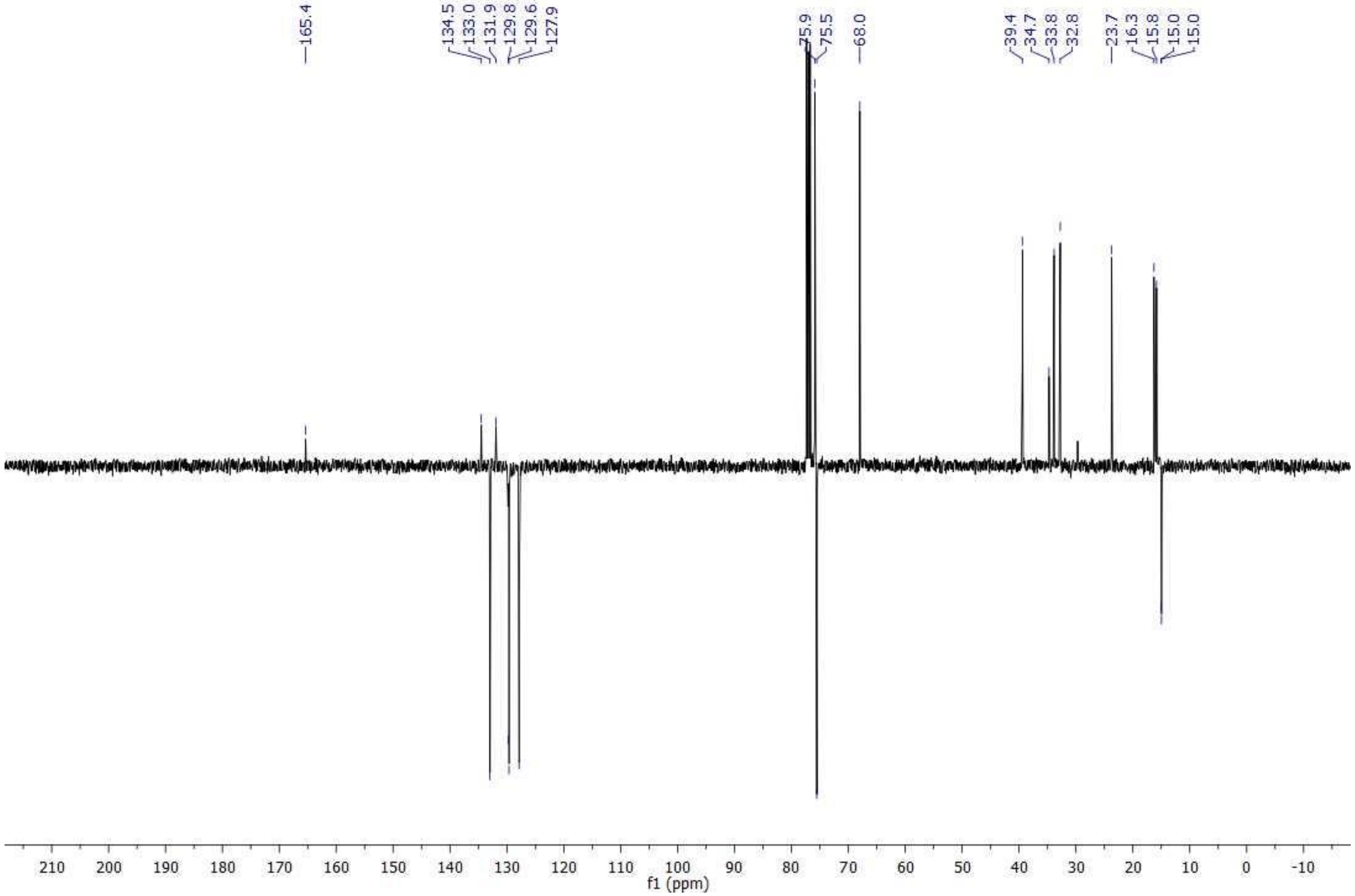

165.4
134.5
133.0
131.9
129.8
129.6
127.9
75.9
75.5
68.0
39.4
34.7
33.8
32.8
23.7
16.3
15.8
15.0
15.0
210 200 190 180 170 160 150 140 130 120 110 100 90 80 70 60 50 40 30 20 10 0 -10
f1 (ppm)

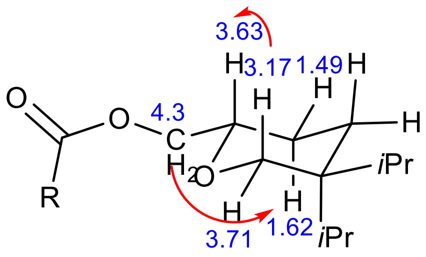
3.63
3.17
1.49
4.3
3.71
1.62
iPr
iPr
R

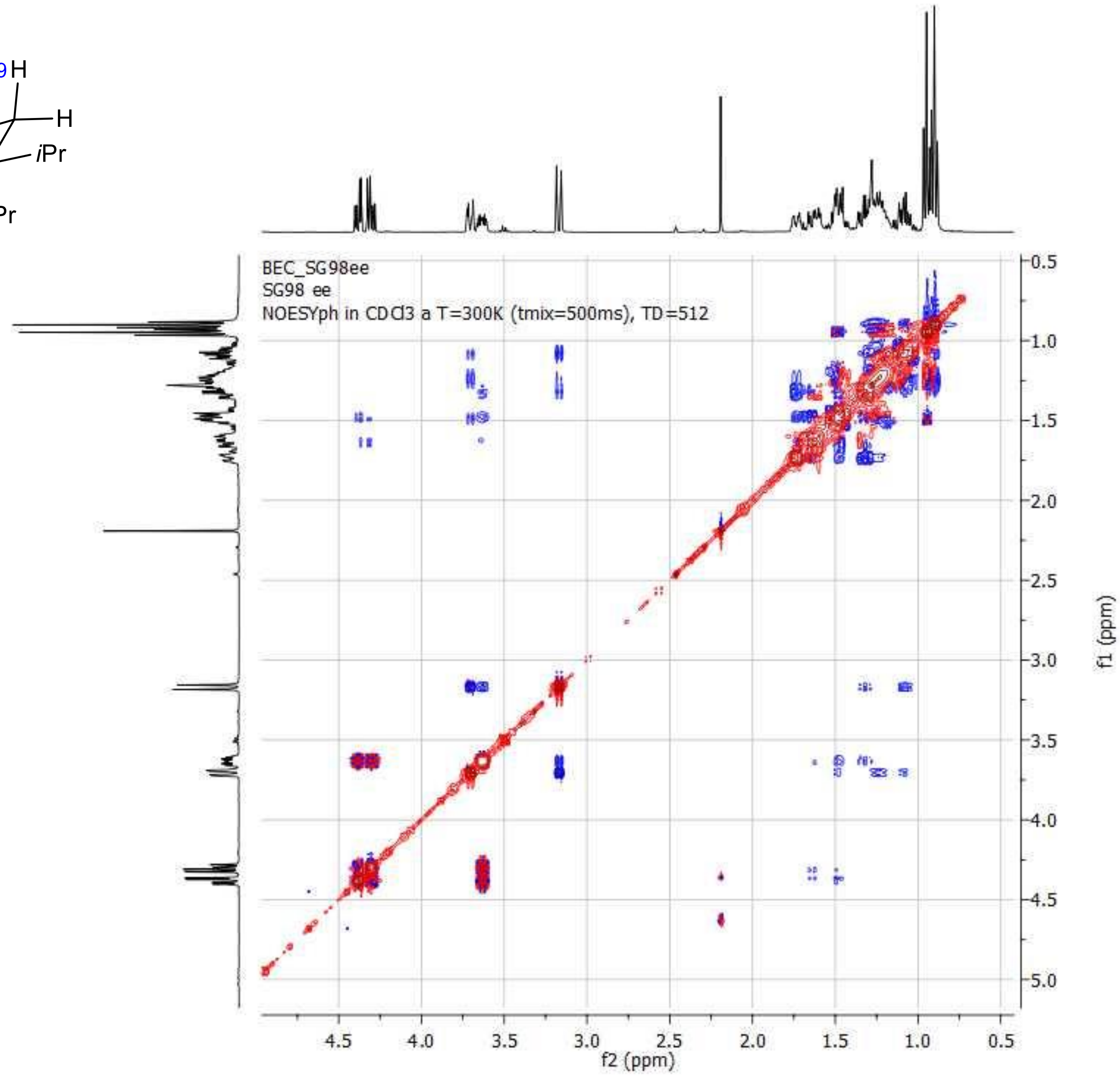
BEC_SG98ee
SG98 ee
NOESYph in CDCl3 a T=300K (tmix=500ms), TD=512
f1 (ppm)
f2 (ppm)

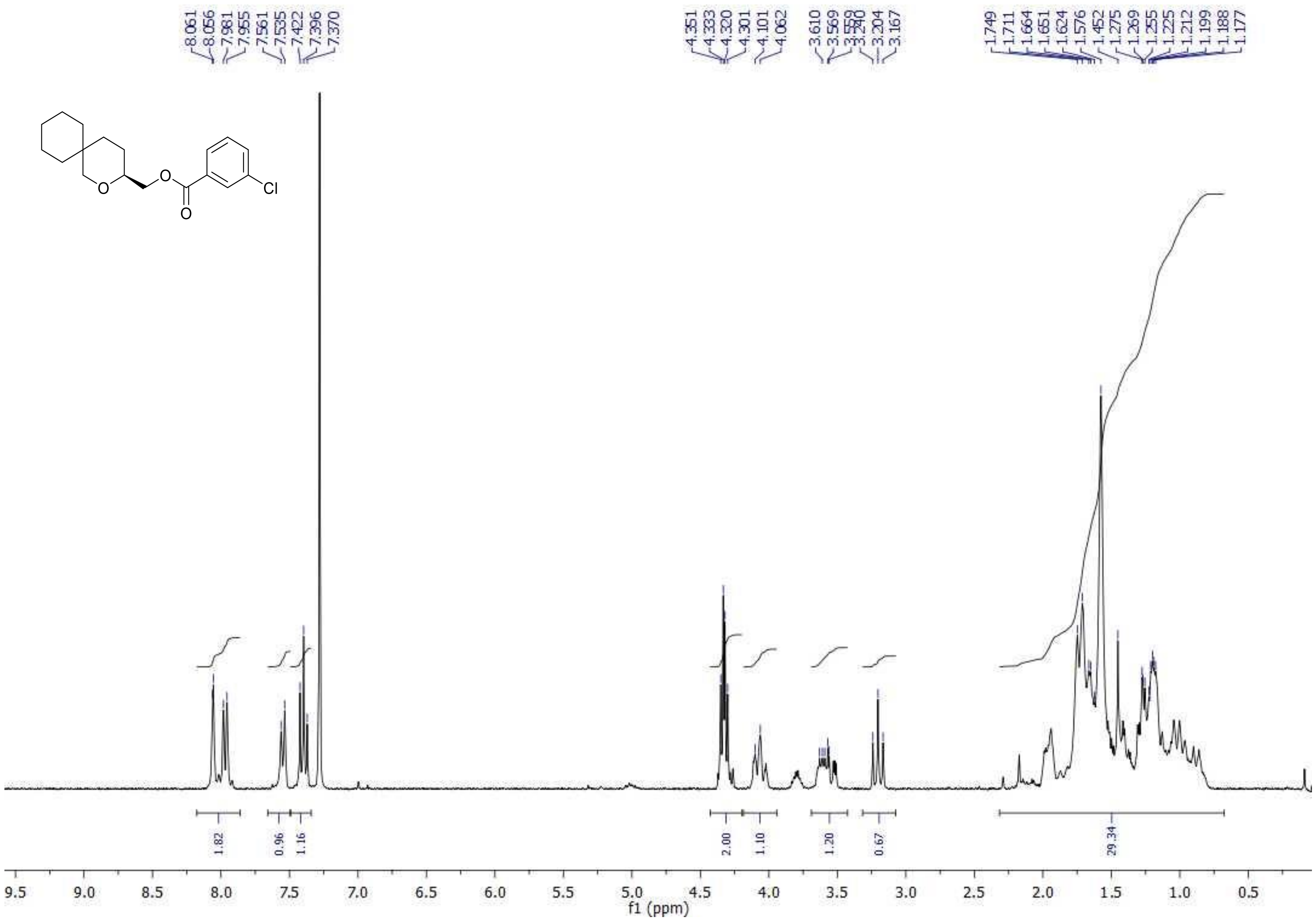

8.061
8.056
7.981
7.955
7.561
7.535
7.422
7.396
7.370
4.351
4.333
4.320
4.301
4.101
4.062
3.610
3.569
3.559
3.540
3.204
3.167
1.749
1.711
1.664
1.651
1.624
1.576
1.452
1.275
1.269
1.255
1.225
1.212
1.199
1.188
1.177
O
O
O
Cl
1.82
0.96
1.16
2.00
1.10
1.20
0.67
29.34
9.5
9.0
8.5
8.0
7.5
7.0
6.5
6.0
5.5
5.0
4.5
4.0
3.5
3.0
2.5
2.0
1.5
1.0
0.5
f1 (ppm)

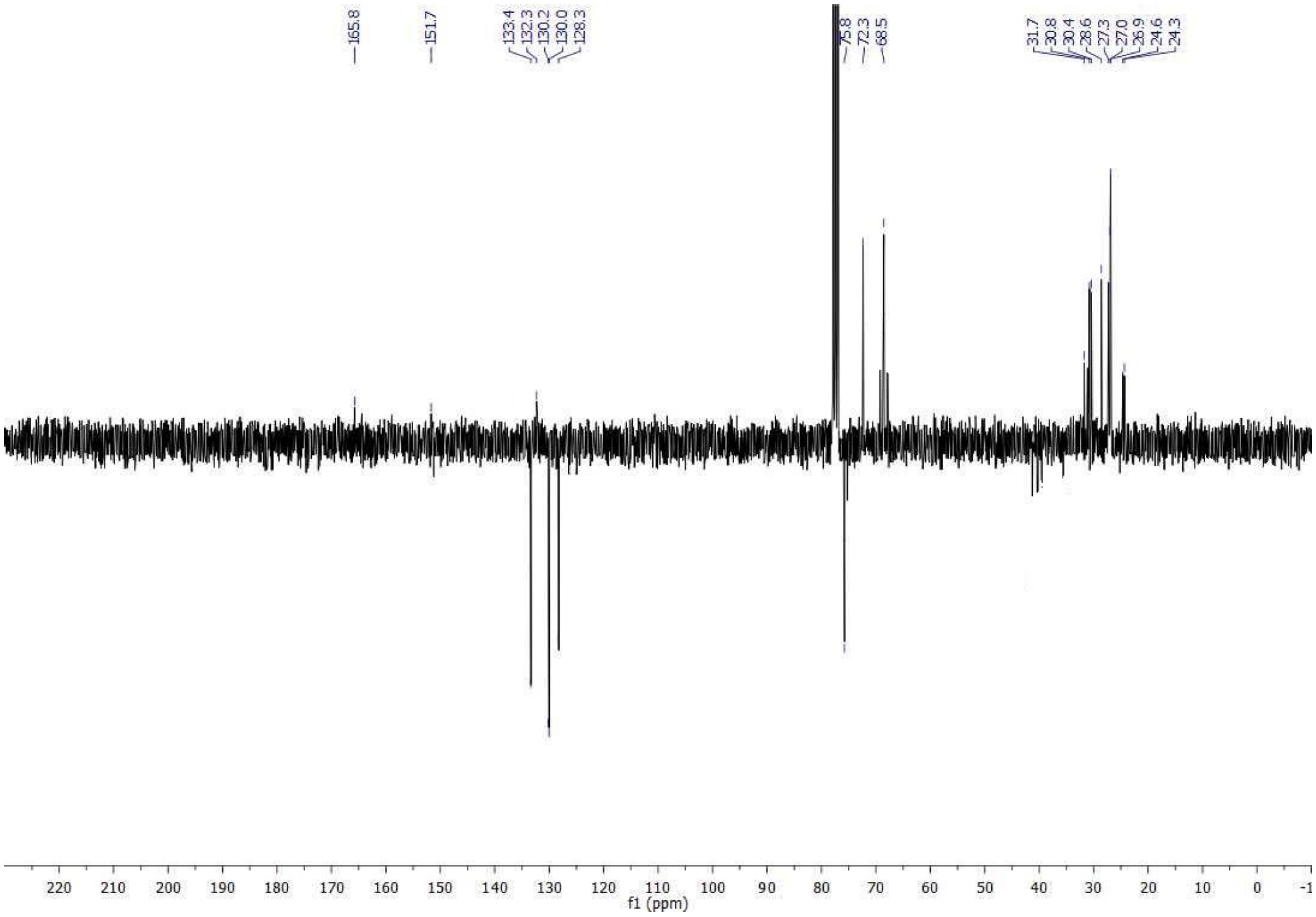
165.8
151.7
133.4
132.3
130.2
130.0
128.3
75.8
72.3
68.5
31.7
30.8
30.4
28.6
27.3
27.0
26.9
24.6
24.3
220 210 200 190 180 170 160 150 140 130 120 110 100 90 80 70 60 50 40 30 20 10 0 -1
f1 (ppm)

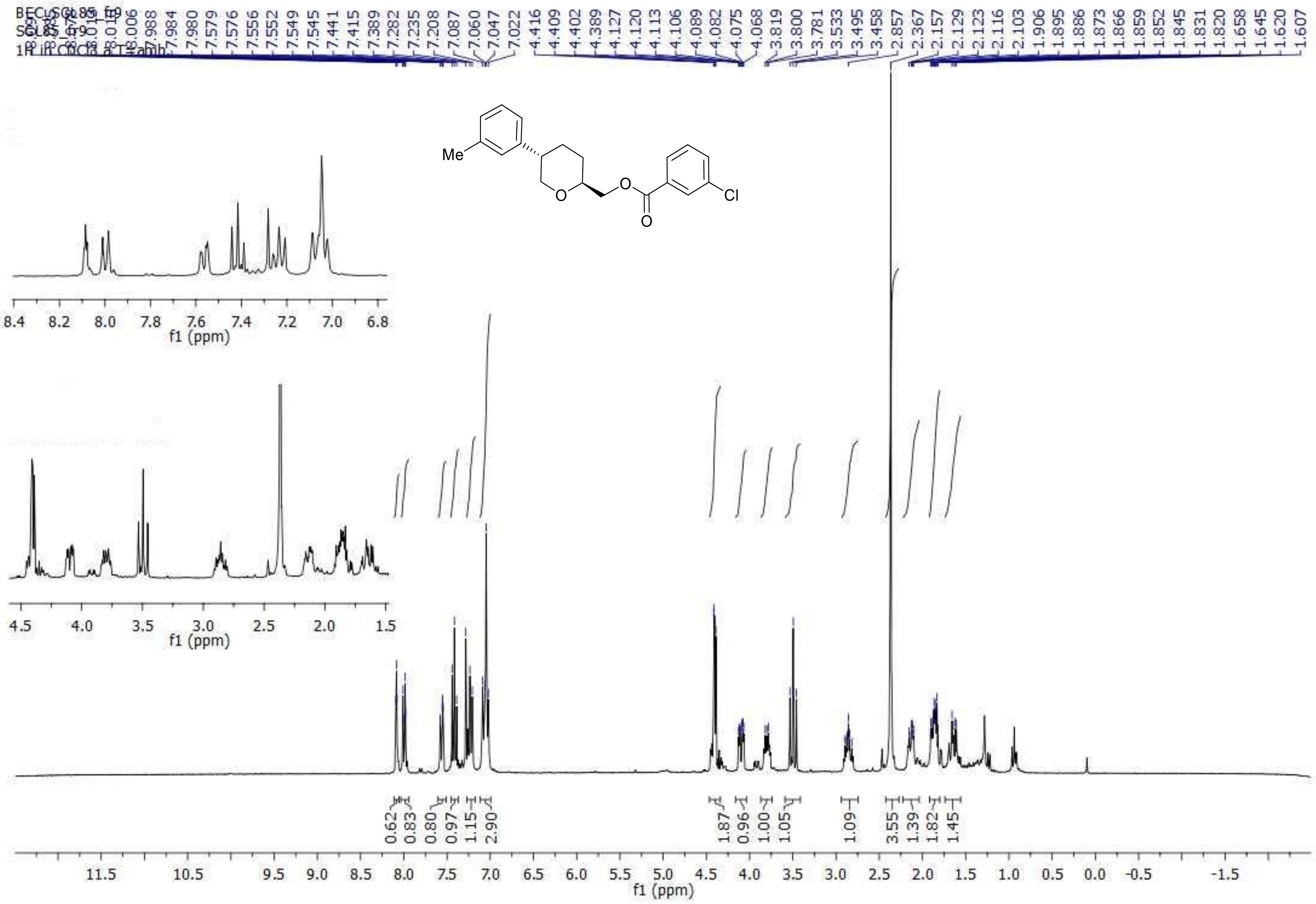

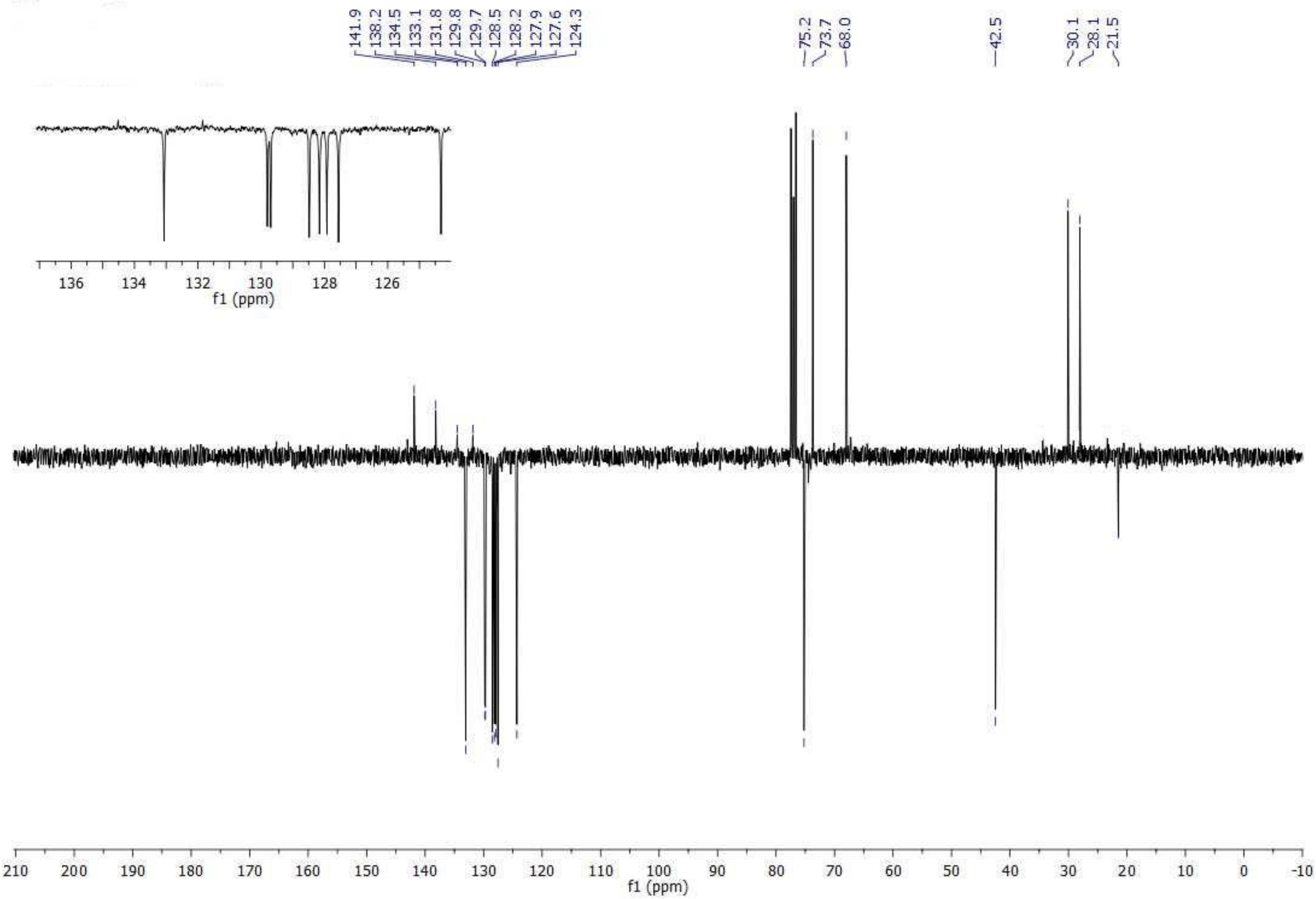
141.9
138.2
134.5
133.1
131.8
129.8
129.7
128.5
128.2
127.9
127.6
124.3
75.2
73.7
68.0
42.5
30.1
28.1
21.5
136 134 132 130 128 126
f1 (ppm)
210 200 190 180 170 160 150 140 130 120 110 100 90 80 70 60 50 40 30 20 10 0 -10
f1 (ppm)

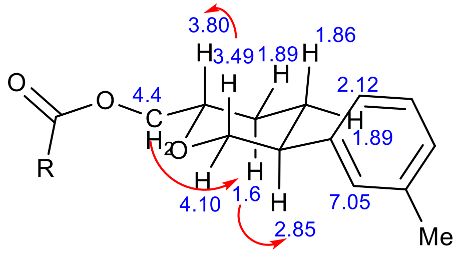
3.80
3.49
1.89
1.86
2.12
4.4
1.89
4.10
1.6
7.05
2.85
Me
R

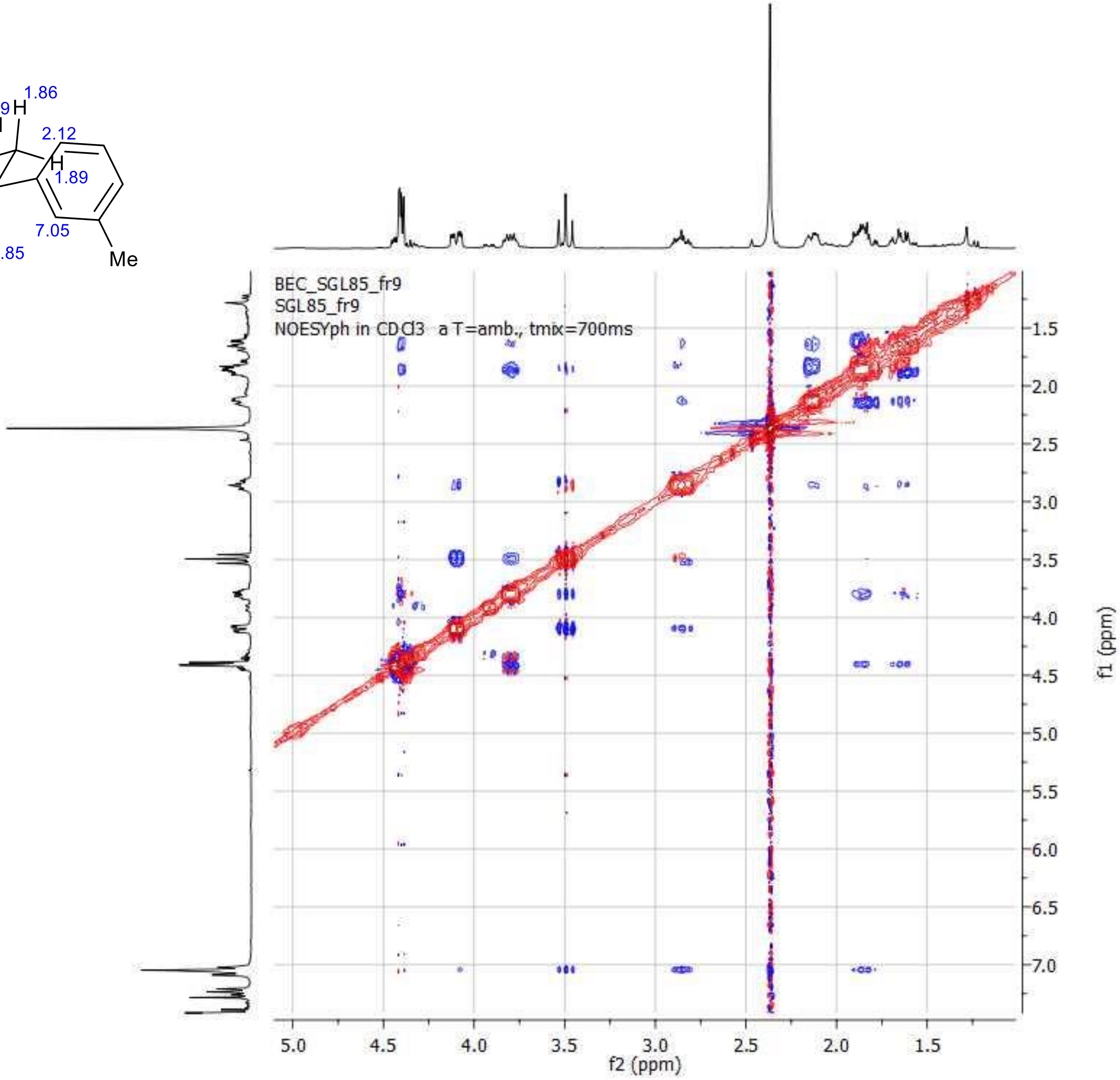
BEC_SGL85_fr9
SGL85_fr9
NOESYph in CDCl3 a T=amb., tmix=700ms
f2 (ppm)
f1 (ppm)

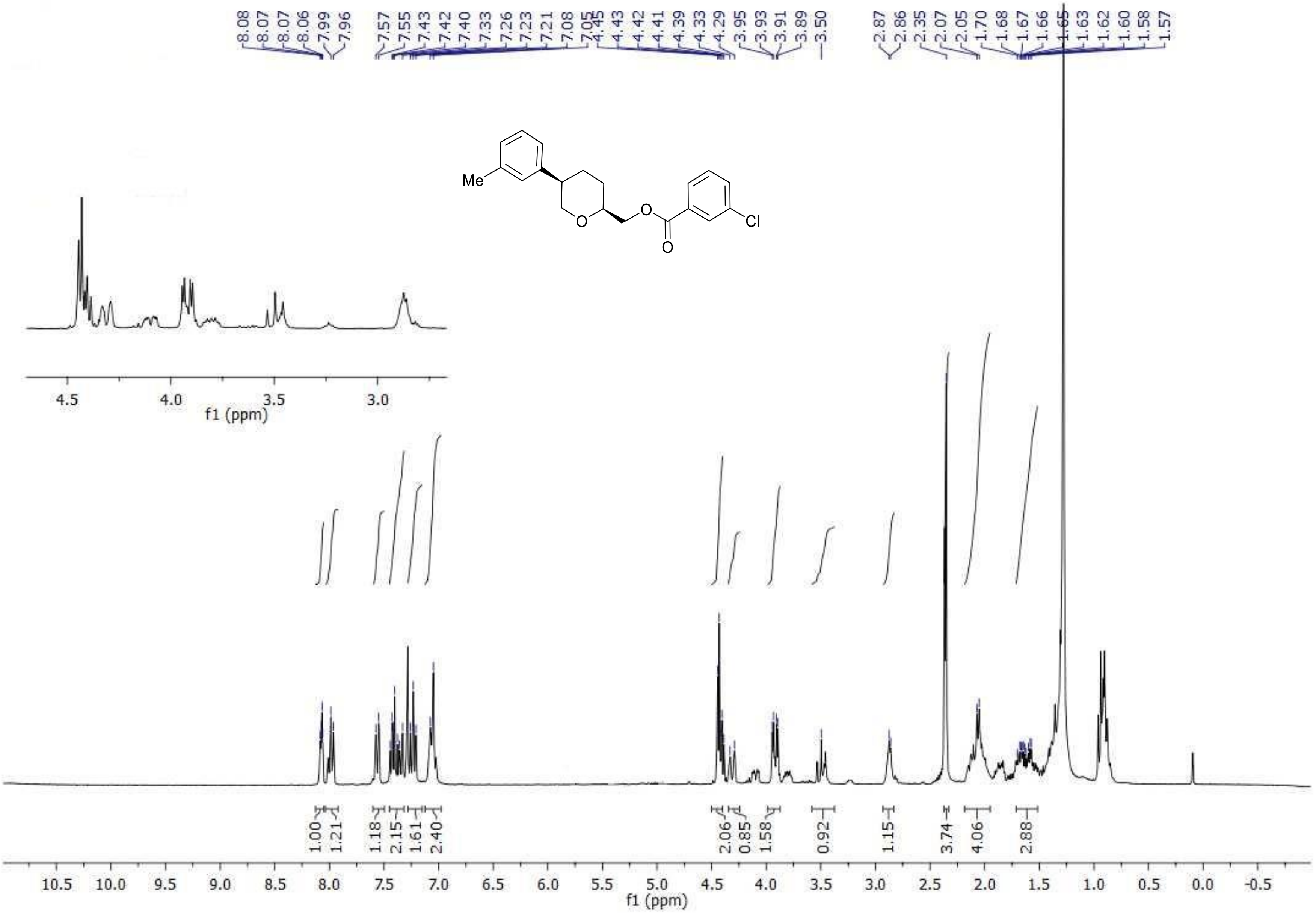

8.08
8.07
8.07
8.06
7.99
7.96
7.57
7.55
7.43
7.42
7.40
7.33
7.26
7.23
7.21
7.08
7.05
4.45
4.43
4.42
4.41
4.39
4.33
4.29
3.95
3.93
3.91
3.89
3.50
2.87
2.86
2.35
2.07
2.05
1.70
1.68
1.67
1.66
1.65
1.63
1.62
1.60
1.58
1.57
Me
O
O
O
Cl
1.00
1.21
1.18
2.15
1.61
2.40
2.06
0.85
1.58
0.92
1.15
3.74
4.06
2.88
f1 (ppm)

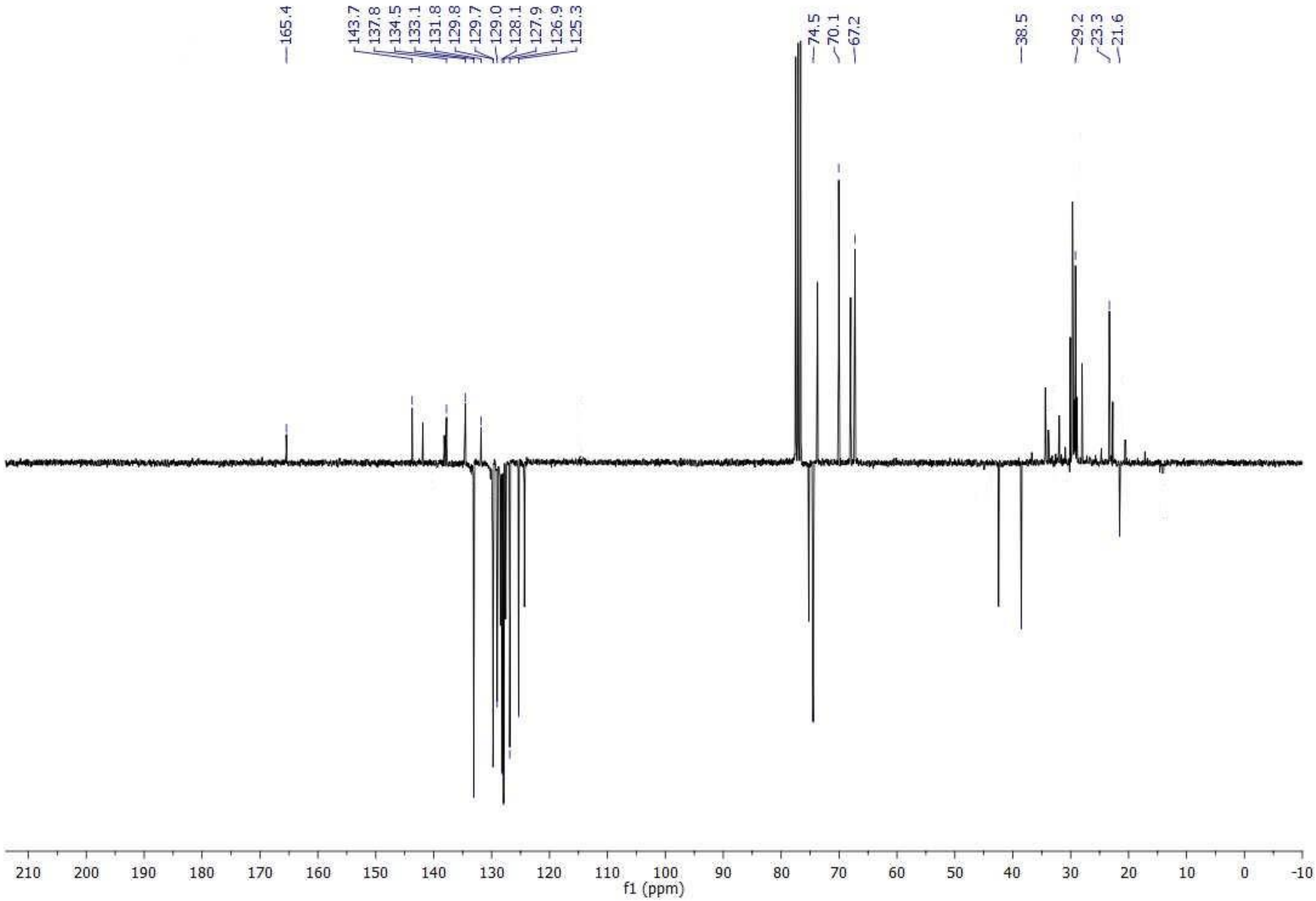
165.4
143.7
137.8
134.5
133.1
131.8
129.8
129.7
129.0
128.1
127.9
126.9
125.3
74.5
70.1
67.2
38.5
29.2
23.3
21.6
210 200 190 180 170 160 150 140 130 120 110 100 90 80 70 60 50 40 30 20 10 0 -10
f1 (ppm)

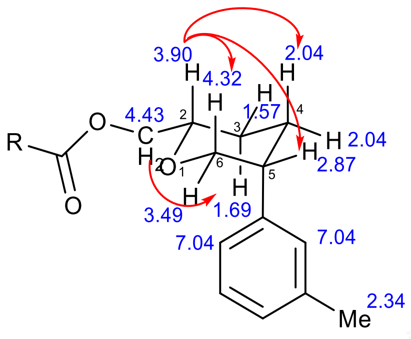

3.90
4.32
2.04
4.43
1.57
2.04
2.87
3.49
1.69
7.04
7.04
2.34
R
O
O
Me


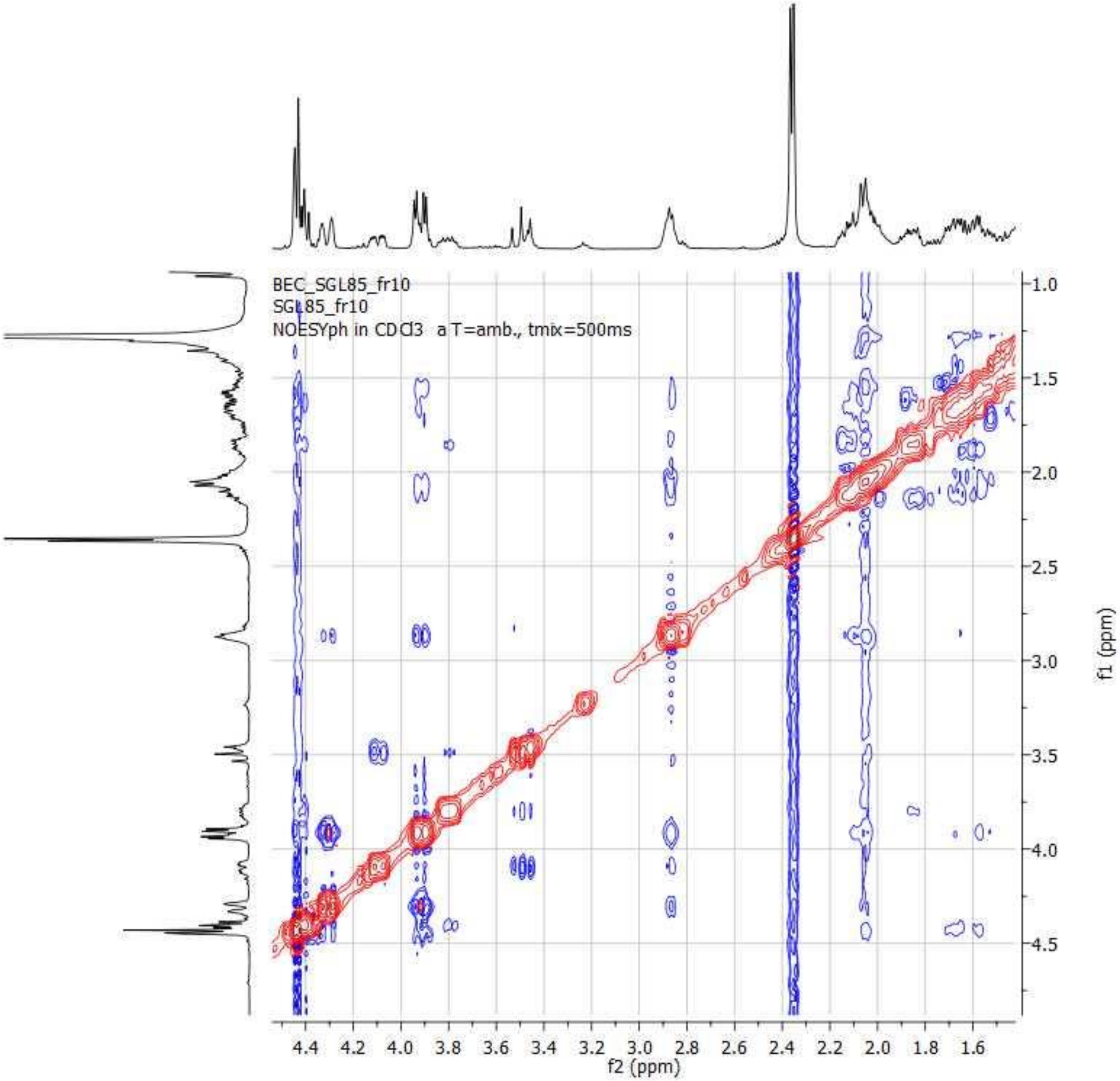

BEC_SGL85_fr10
SGL85_fr10
NOESYph in CDCl3 a T=amb., tmix=500ms
f2 (ppm)
f1 (ppm)

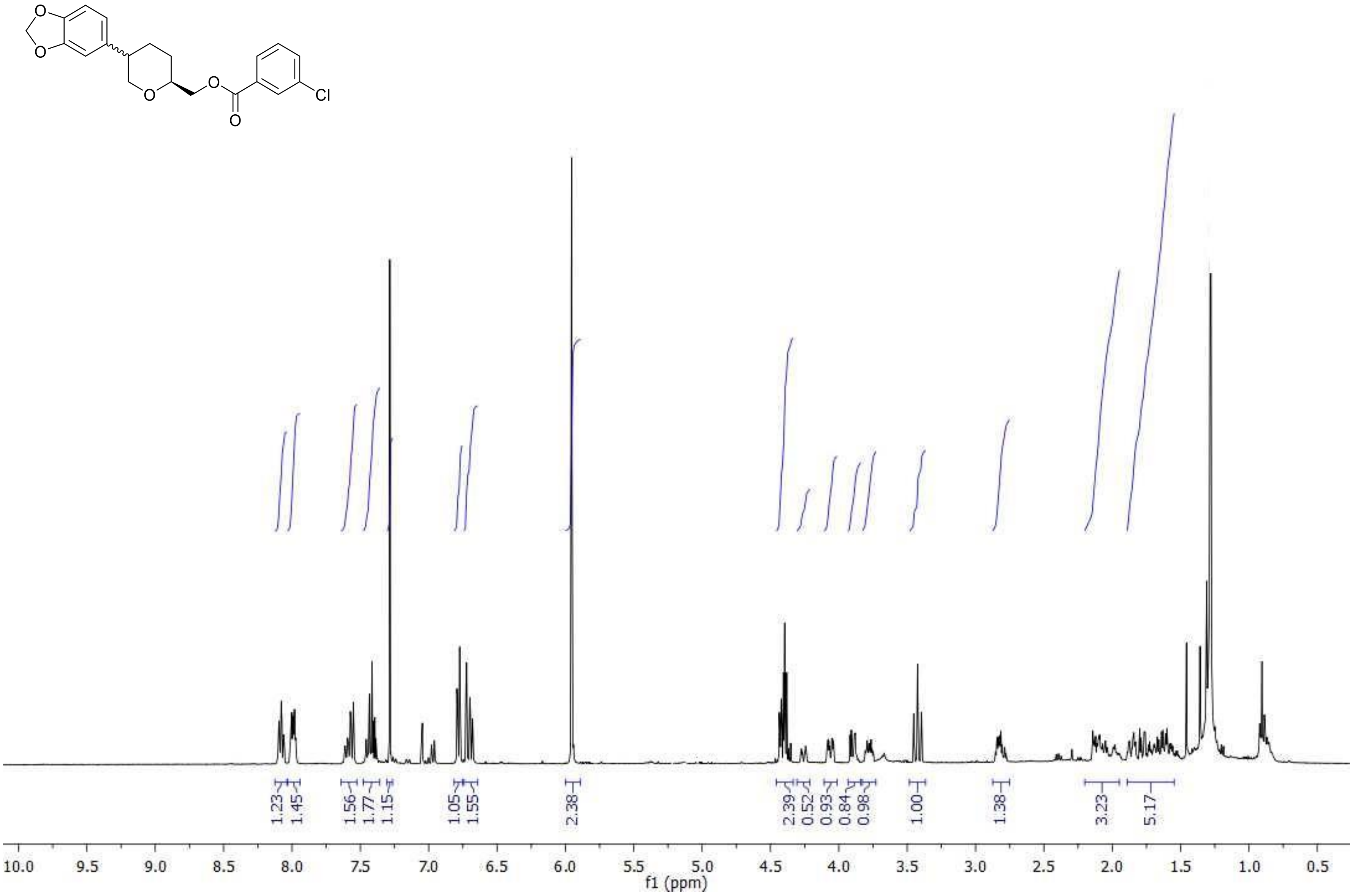
O
O
O
O
O
Cl
1.23
1.45
1.56
1.77
1.15
1.05
1.55
2.38
2.39
0.52
0.93
0.84
0.98
1.00
1.38
3.23
5.17
10.0
9.5
9.0
8.5
8.0
7.5
7.0
6.5
6.0
5.5
5.0
4.5
4.0
3.5
3.0
2.5
2.0
1.5
1.0
0.5
f1 (ppm)

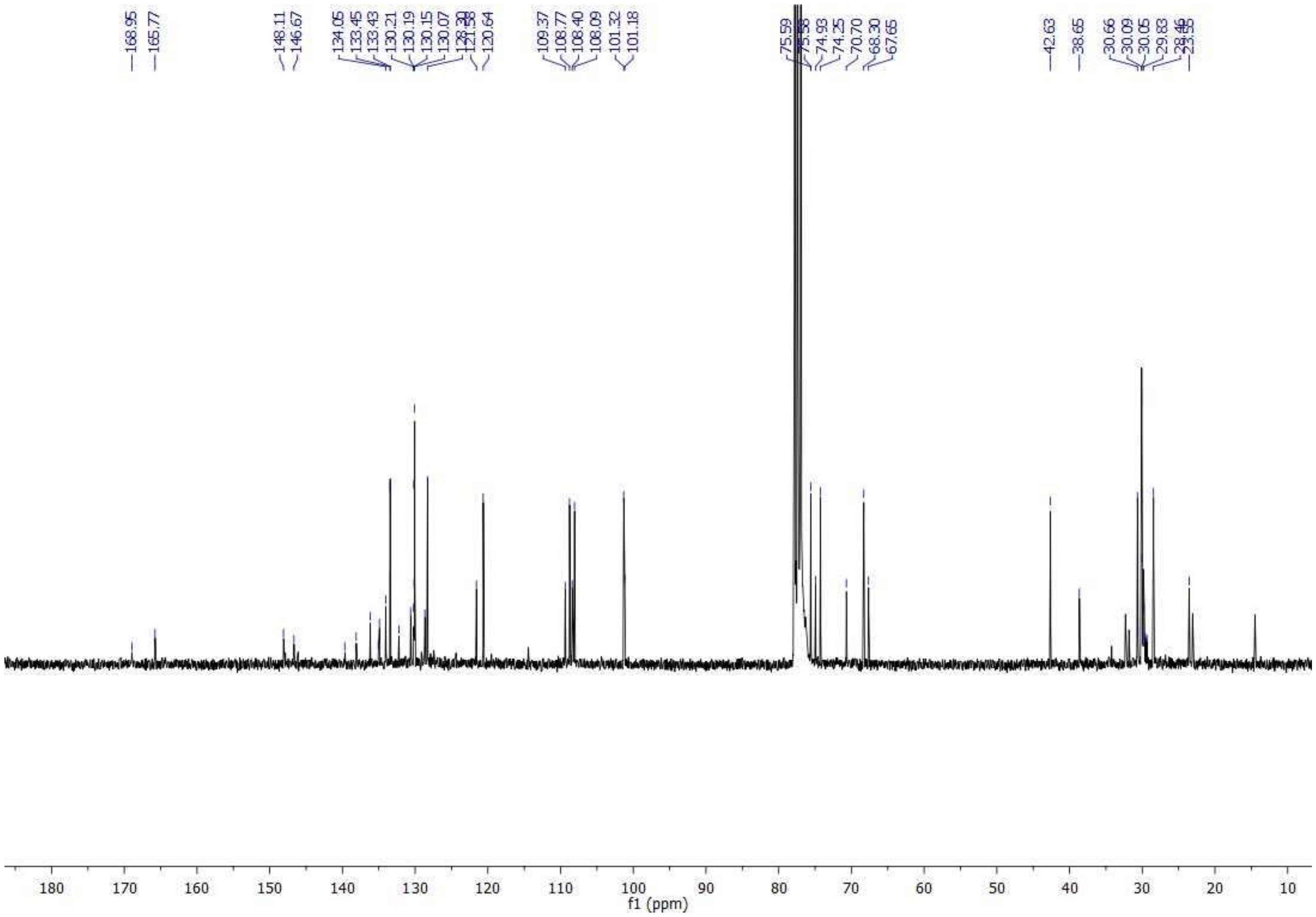

168.95
165.77
148.11
146.67
134.05
133.45
133.43
130.21
130.19
130.15
130.07
128.30
121.58
120.64
109.37
108.77
108.40
108.09
101.32
101.18
75.59
74.93
74.25
70.70
68.30
67.65
42.63
38.65
30.66
30.09
30.05
29.83
23.55
180
170
160
150
140
130
120
110
100
90
80
70
60
50
40
30
20
10
f1 (ppm)

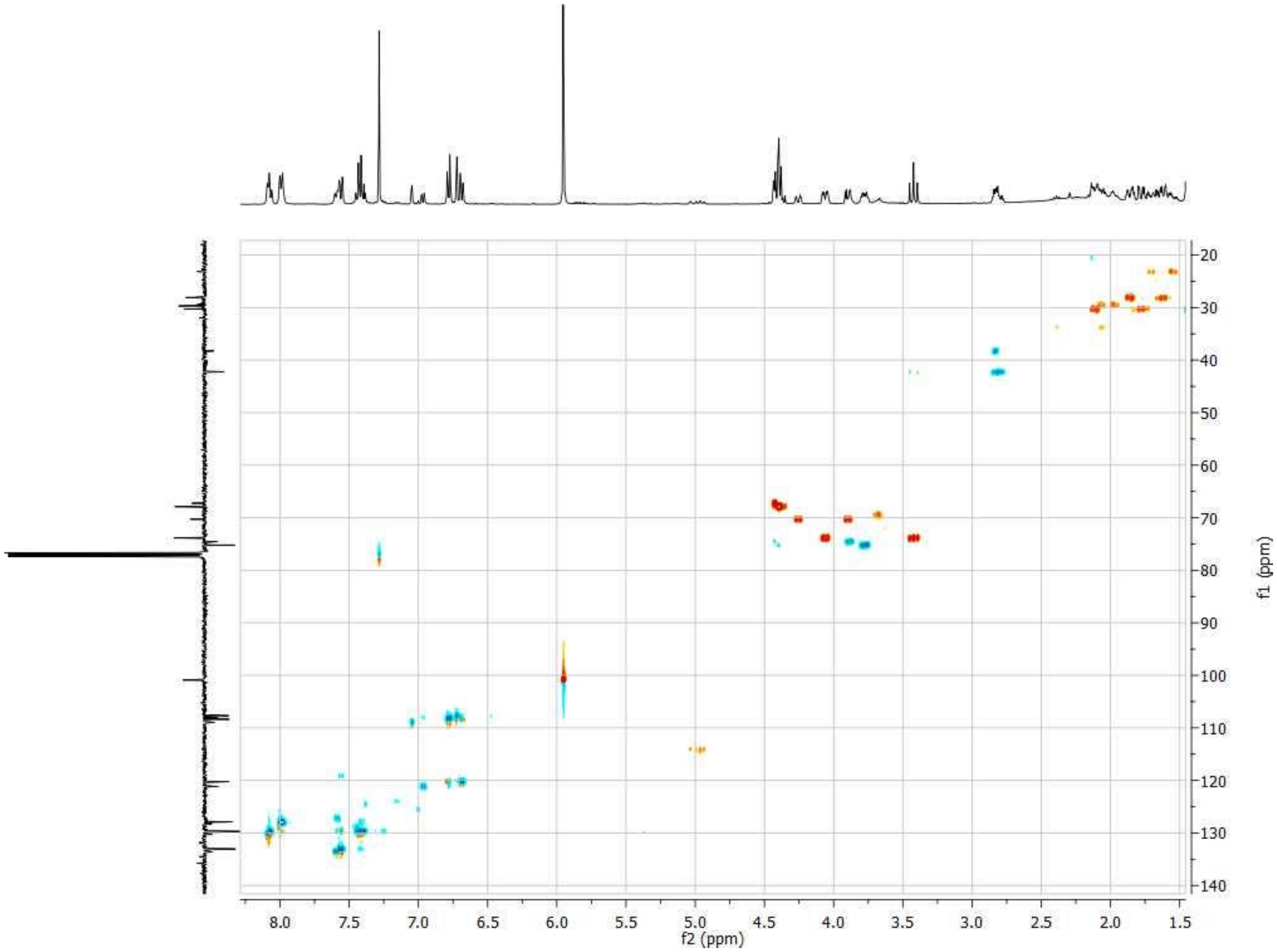
f2 (ppm)
f1 (ppm)

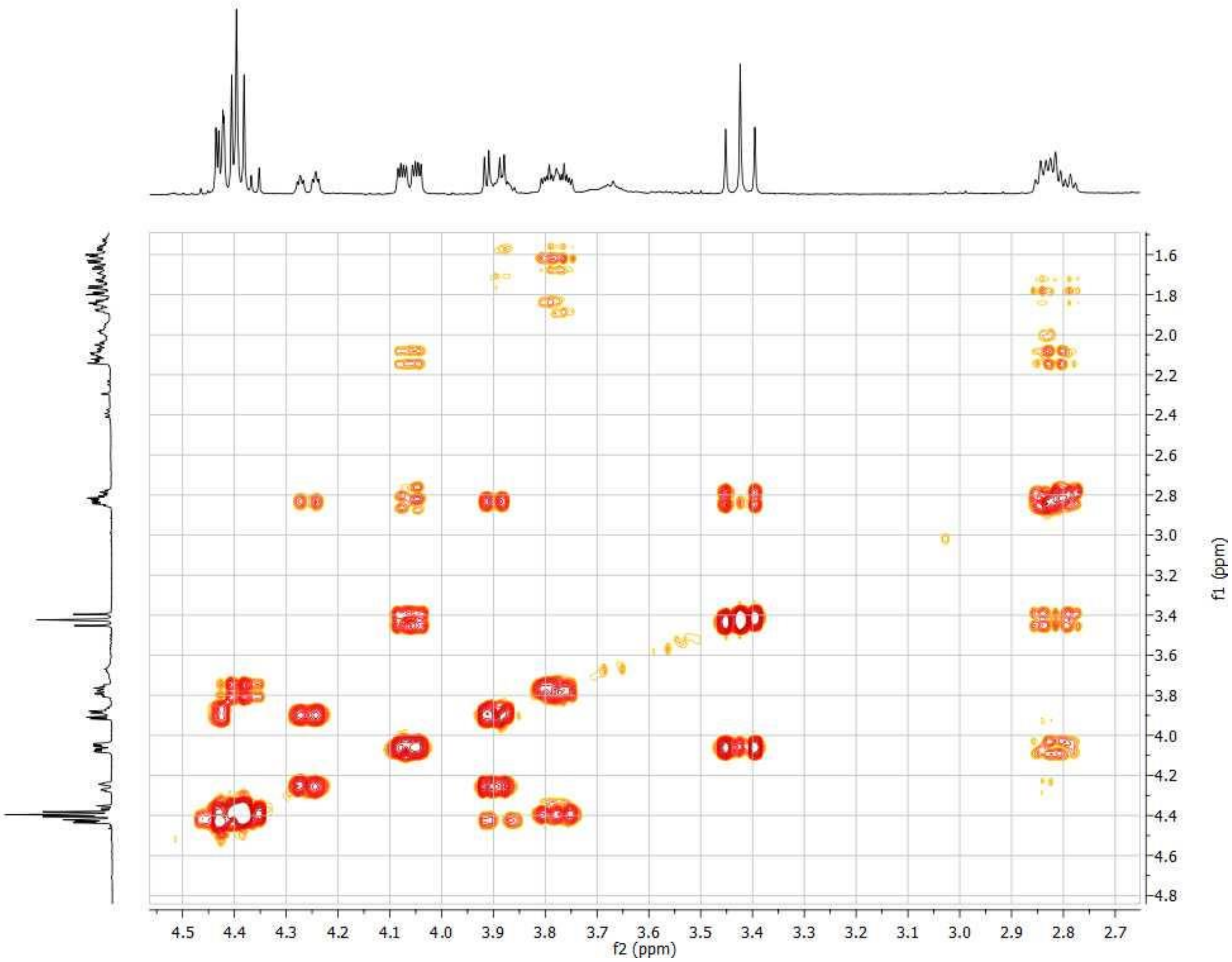

f2 (ppm)
f1 (ppm)
4.5
4.4
4.3
4.2
4.1
4.0
3.9
3.8
3.7
3.6
3.5
3.4
3.3
3.2
3.1
3.0
2.9
2.8
2.7
1.6
1.8
2.0
2.2
2.4
2.6
2.8
3.0
3.2
3.4
3.6
3.8
4.0
4.2
4.4
4.6
4.8

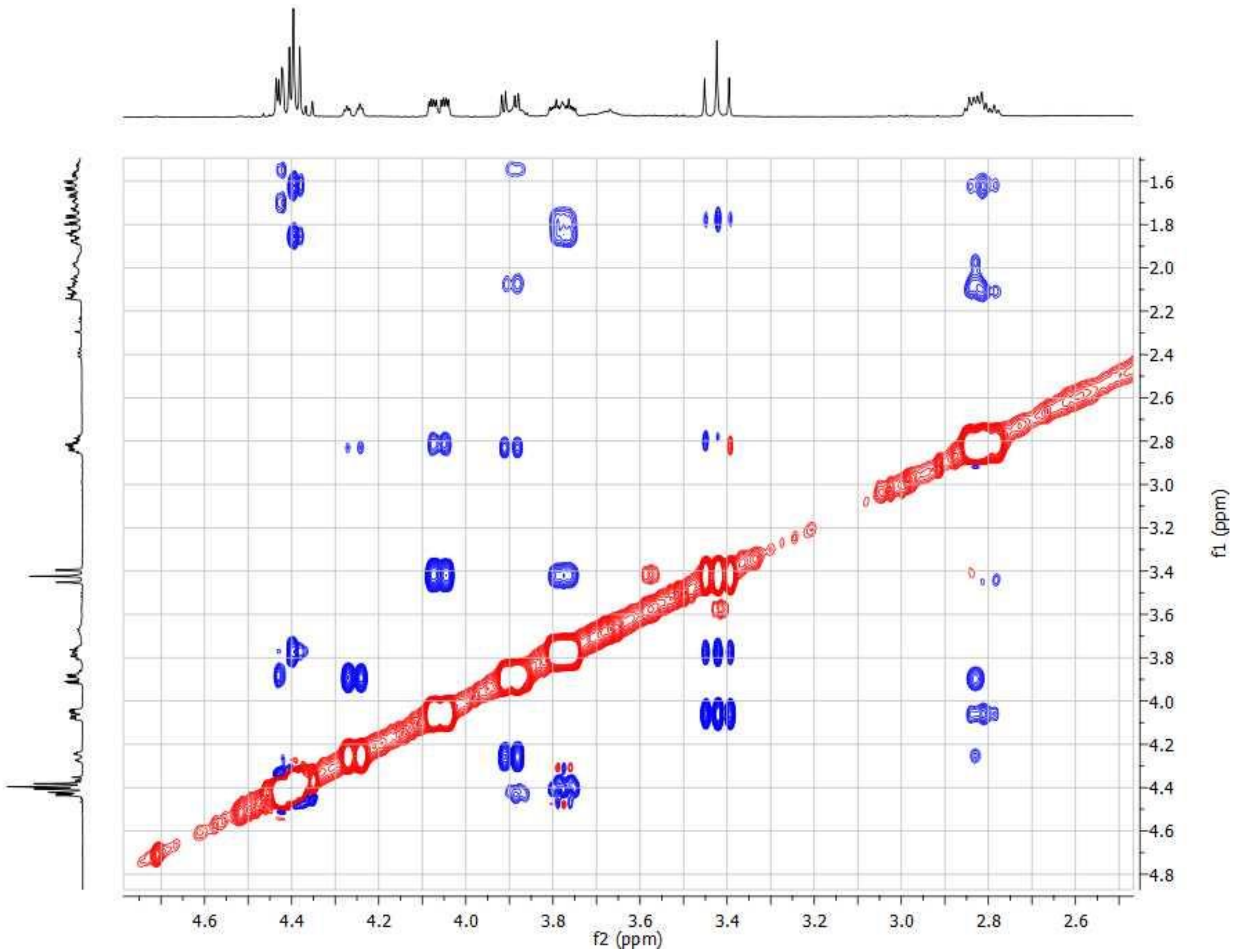

4.6
4.4
4.2
4.0
3.8
3.6
3.4
3.2
3.0
2.8
2.6
f2 (ppm)
1.6
1.8
2.0
2.2
2.4
2.6
2.8
3.0
3.2
3.4
3.6
3.8
4.0
4.2
4.4
4.6
4.8
f1 (ppm)

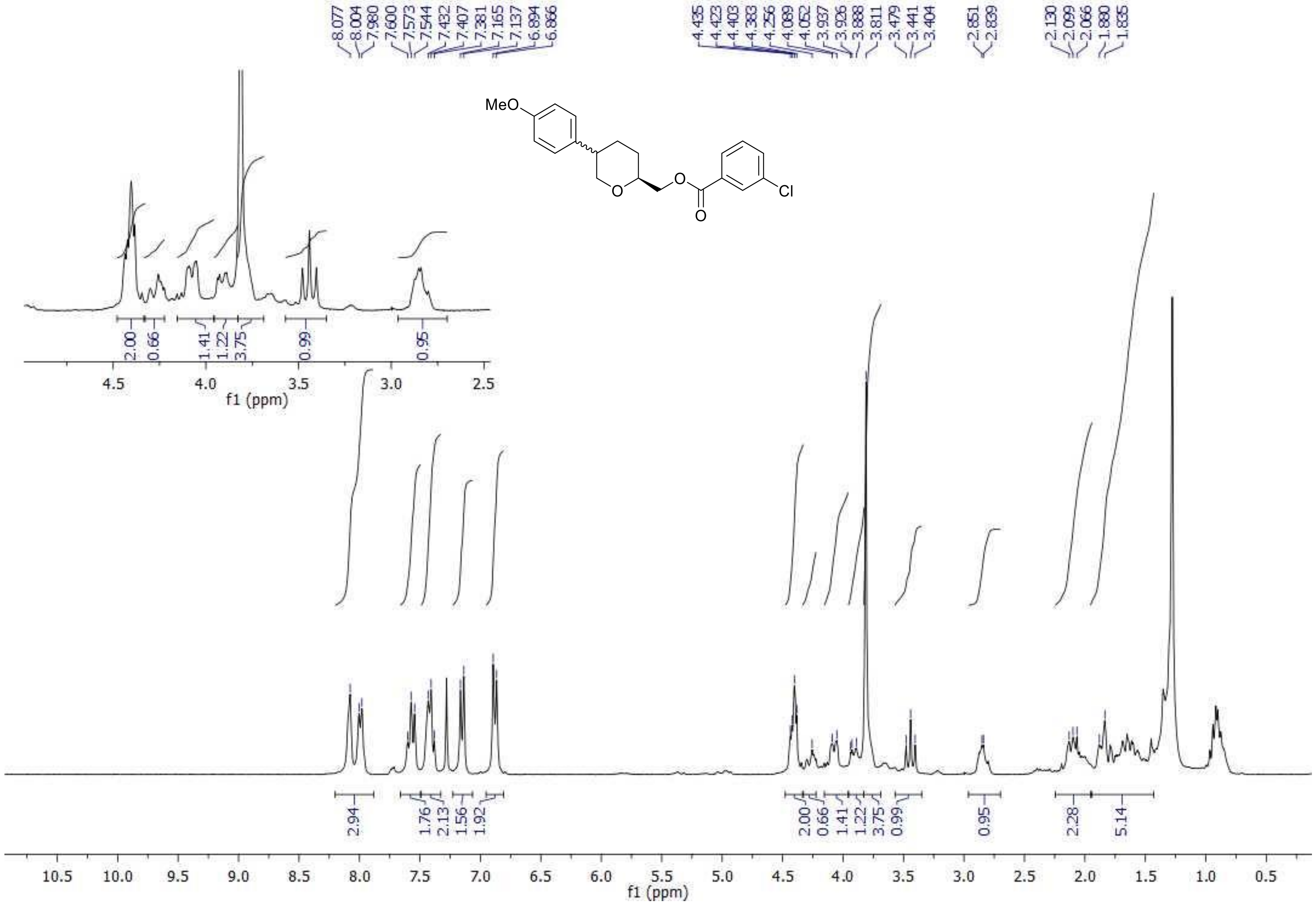

MeO
O
O
O
Cl
f1 (ppm)

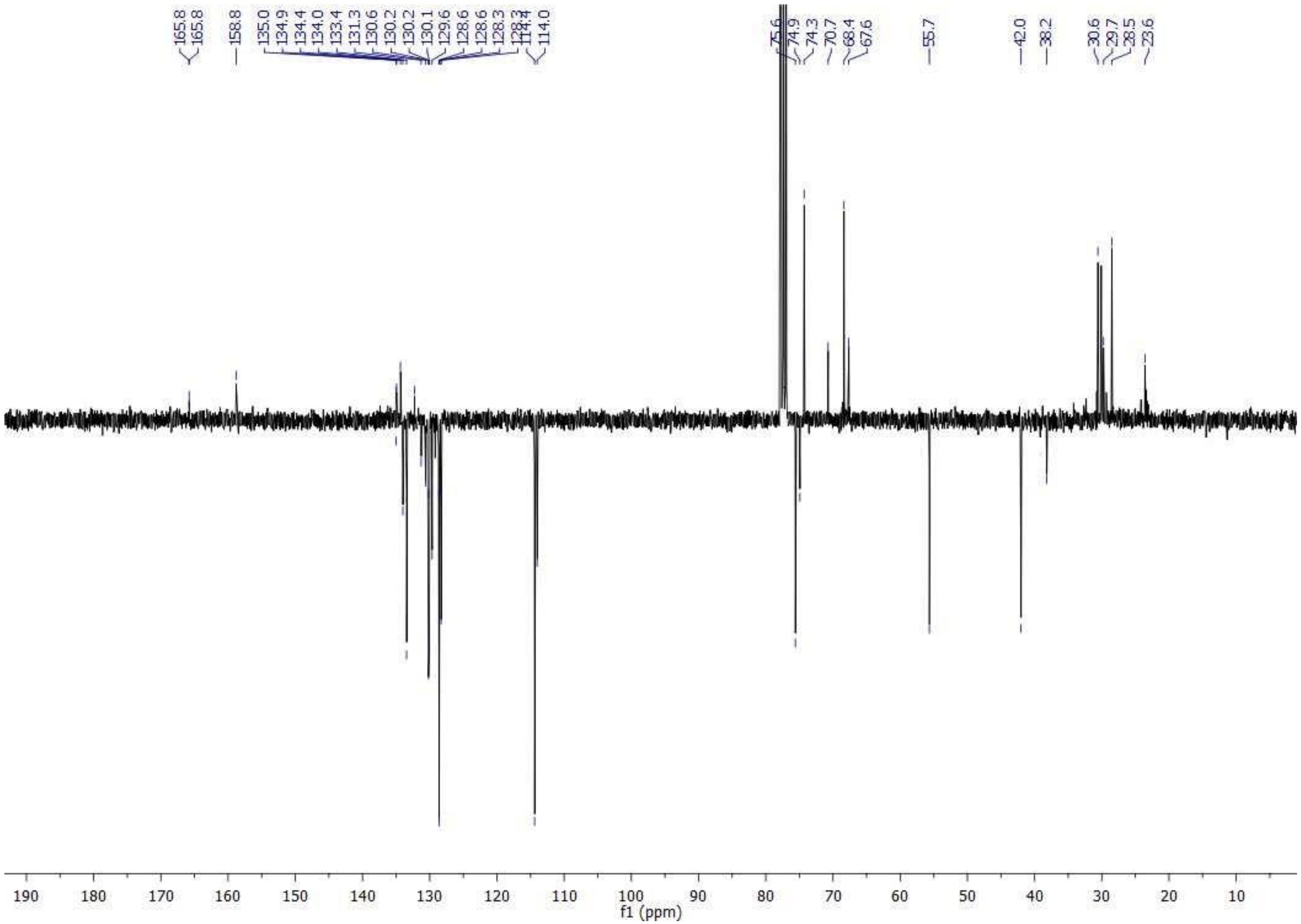
165.8
165.8
158.8
135.0
134.9
134.4
134.0
133.4
131.3
130.6
130.2
130.2
130.1
129.6
128.6
128.6
128.3
128.3
114.4
114.0
75.6
74.9
74.3
70.7
68.4
67.6
55.7
42.0
38.2
30.6
29.7
28.5
23.6
190 180 170 160 150 140 130 120 110 100 90 80 70 60 50 40 30 20 10
f1 (ppm)

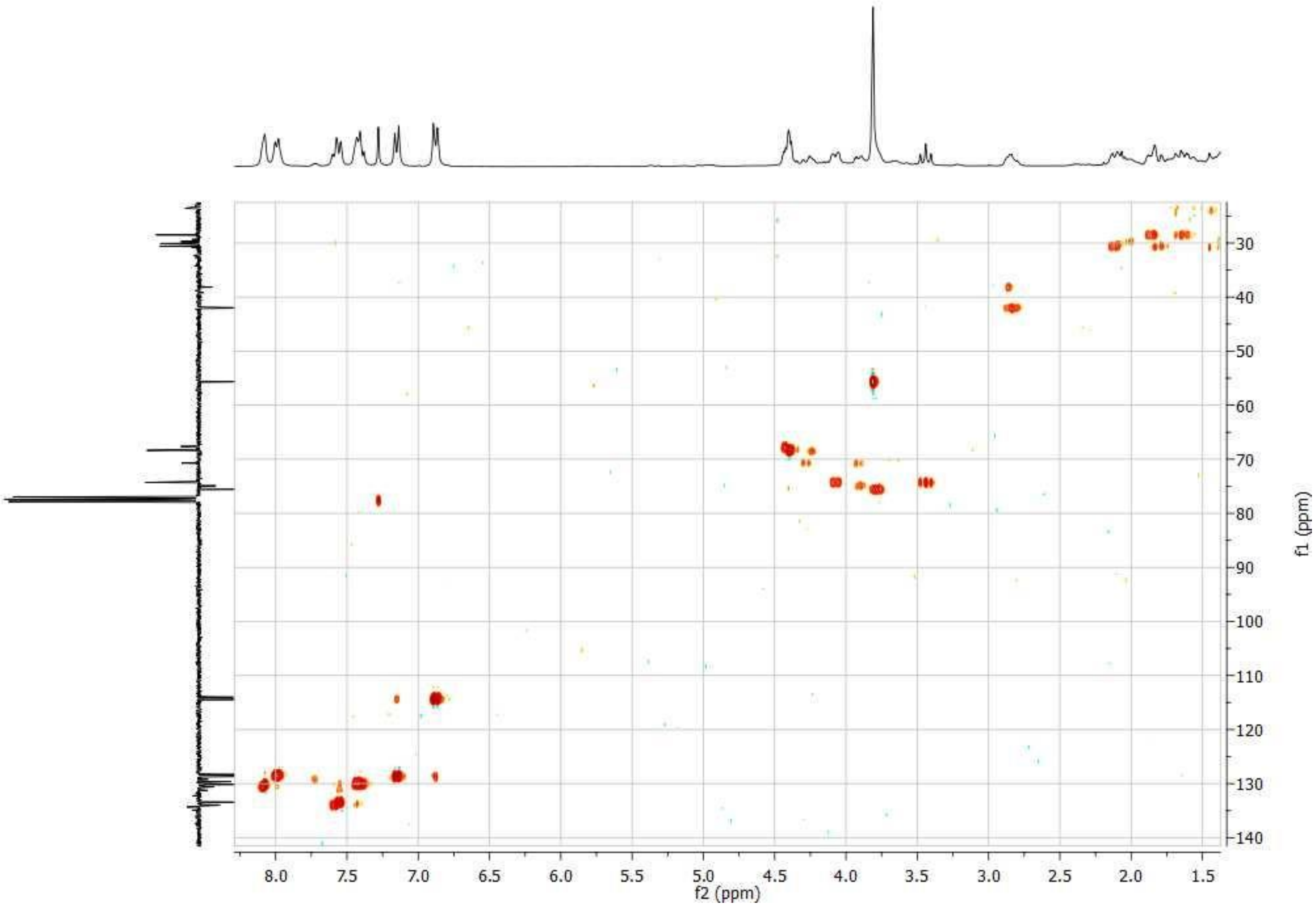

f2 (ppm)
f1 (ppm)
8.0
7.5
7.0
6.5
6.0
5.5
5.0
4.5
4.0
3.5
3.0
2.5
2.0
1.5
30
40
50
60
70
80
90
100
110
120
130
140

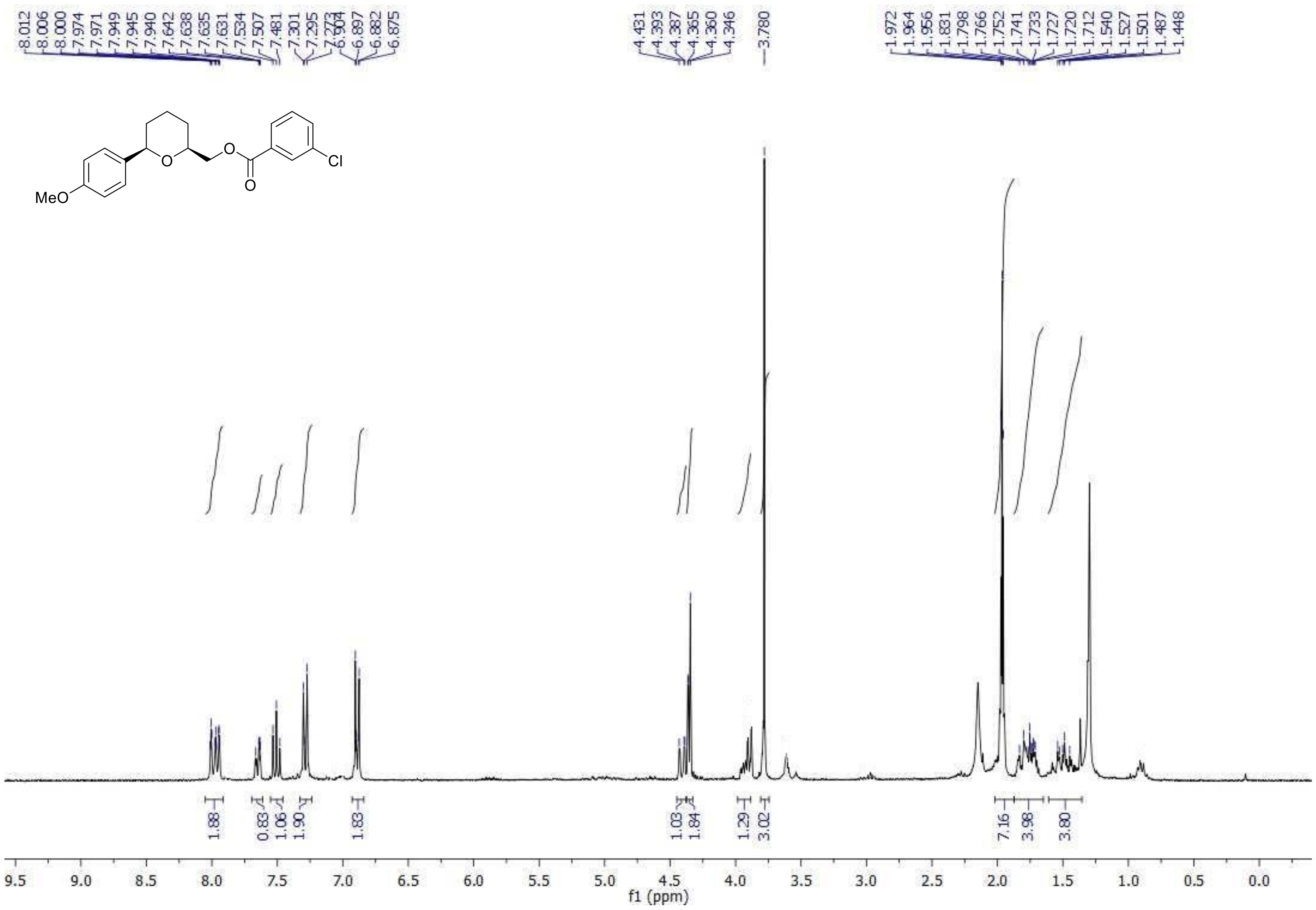

8.012 8.006 8.000 7.974 7.971 7.949 7.945 7.940 7.642 7.638 7.635 7.631 7.534 7.507 7.481 7.301 7.295 7.273 6.904 6.897 6.882 6.875
4.431 4.393 4.387 4.365 4.360 4.346
3.780
1.972 1.964 1.956 1.831 1.798 1.766 1.752 1.741 1.733 1.727 1.720 1.712 1.540 1.527 1.501 1.487 1.448
MeO
O
O
O
Cl
1.88
0.83
1.06
1.90
1.83
1.03
1.84
1.29
3.02
7.16
3.98
3.80
9.5 9.0 8.5 8.0 7.5 7.0 6.5 6.0 5.5 5.0 4.5 4.0 3.5 3.0 2.5 2.0 1.5 1.0 0.5 0.0
f1 (ppm)

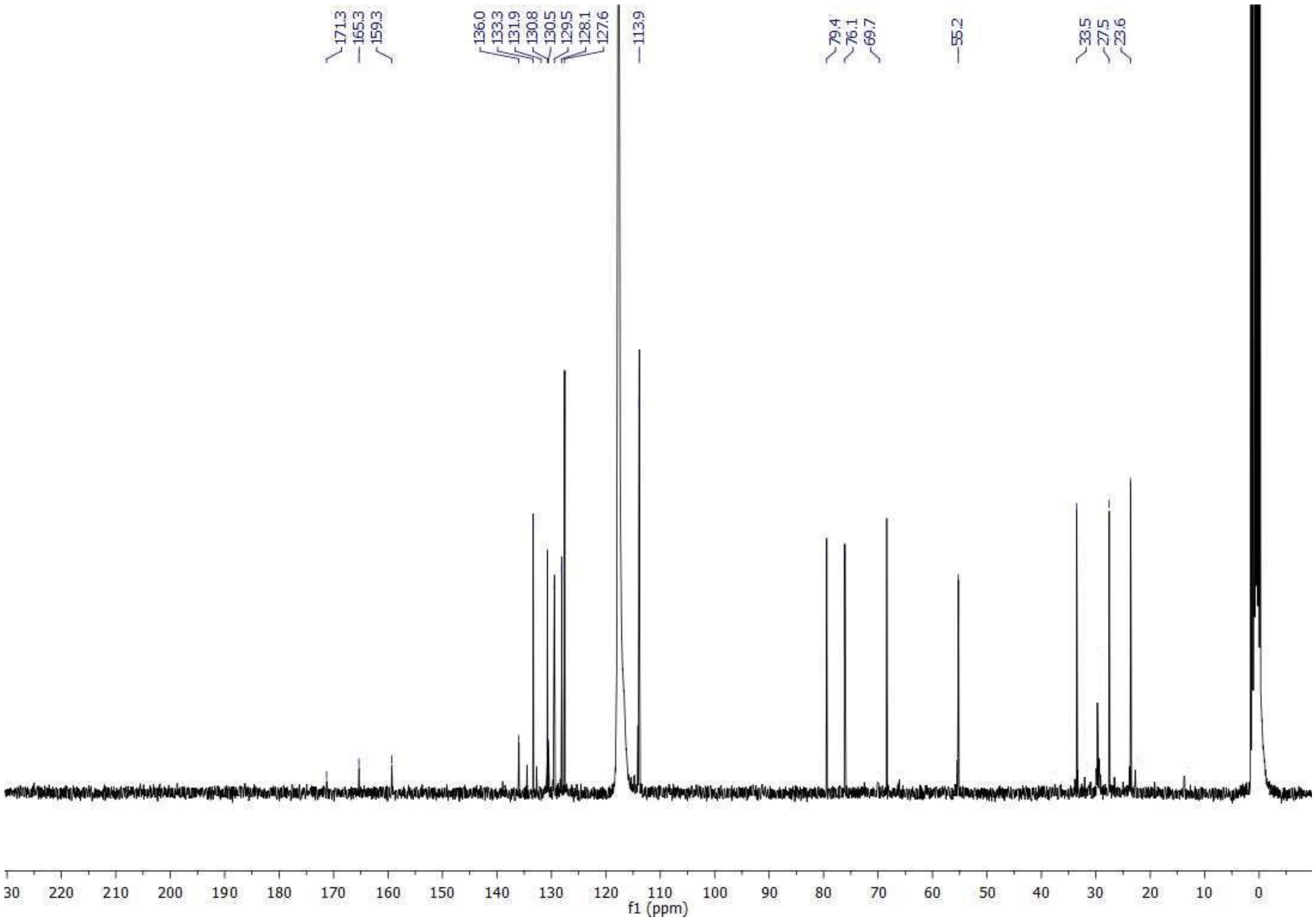
171.3
165.3
159.3
136.0
133.3
131.9
130.8
130.5
129.5
128.1
127.6
113.9
79.4
76.1
69.7
55.2
33.5
27.5
23.6
30
220
210
200
190
180
170
160
150
140
130
120
110
100
90
80
70
60
50
40
30
20
10
0
f1 (ppm)

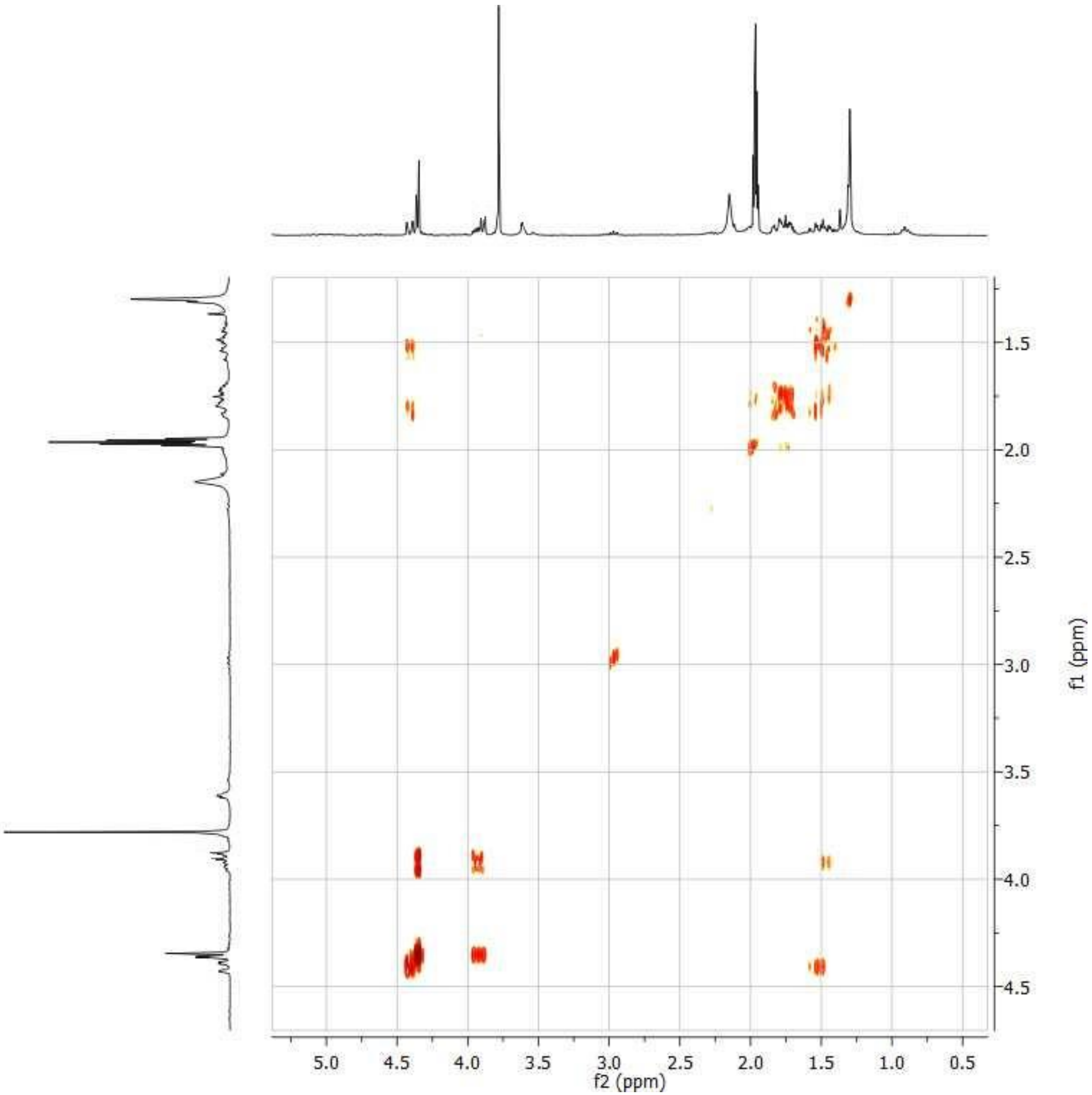

1.5
2.0
2.5
3.0
3.5
4.0
4.5
f1 (ppm)
5.0
4.5
4.0
3.5
3.0
2.5
2.0
1.5
1.0
0.5
f2 (ppm)

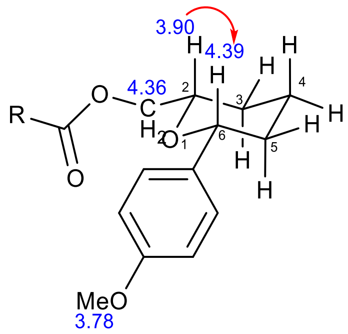
3.90
4.39
4.36
R
O
C
H2
O
1
2
3
4
5
6
H
MeO
3.78

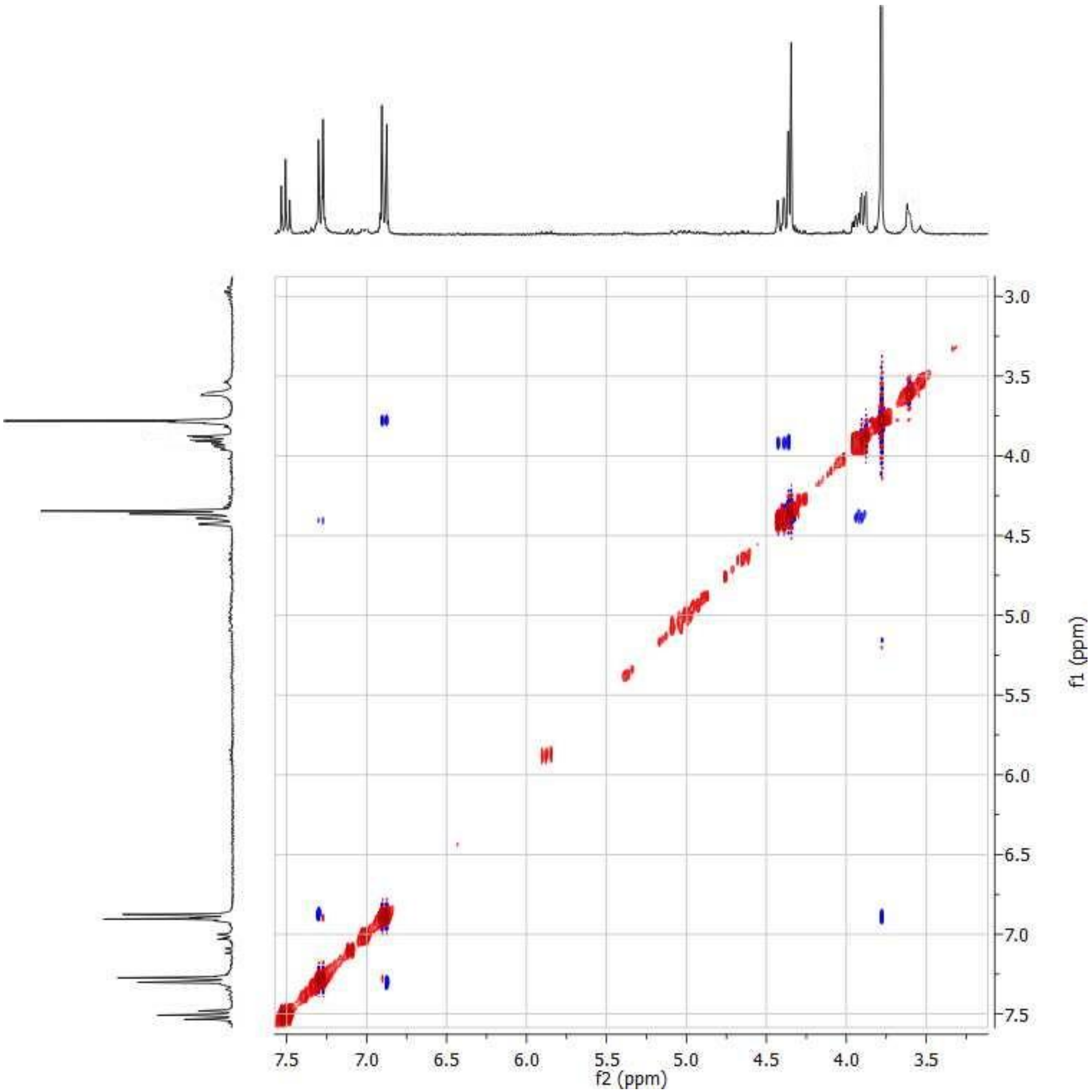
f2 (ppm)
f1 (ppm)
7.5
7.0
6.5
6.0
5.5
5.0
4.5
4.0
3.5
3.0

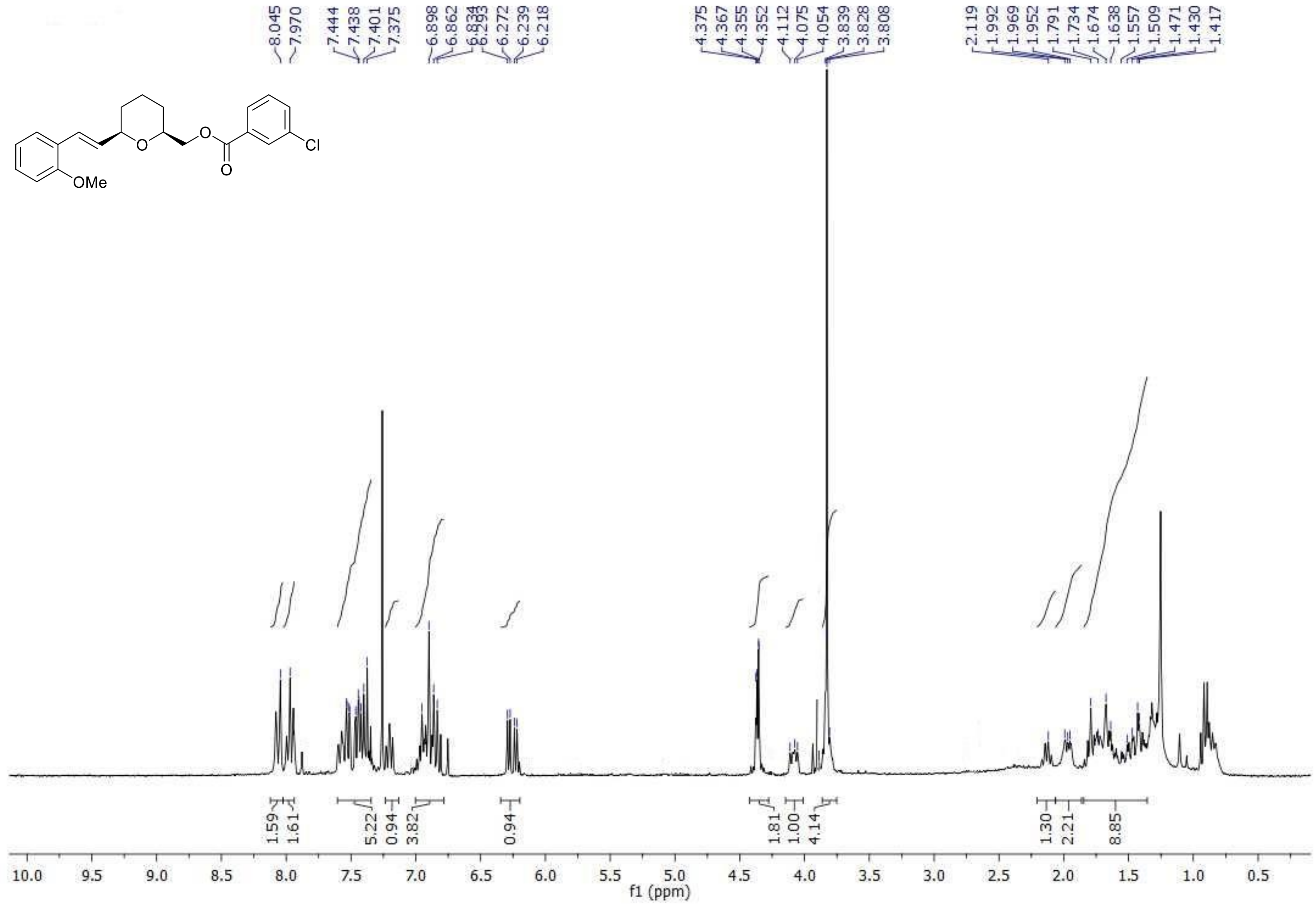

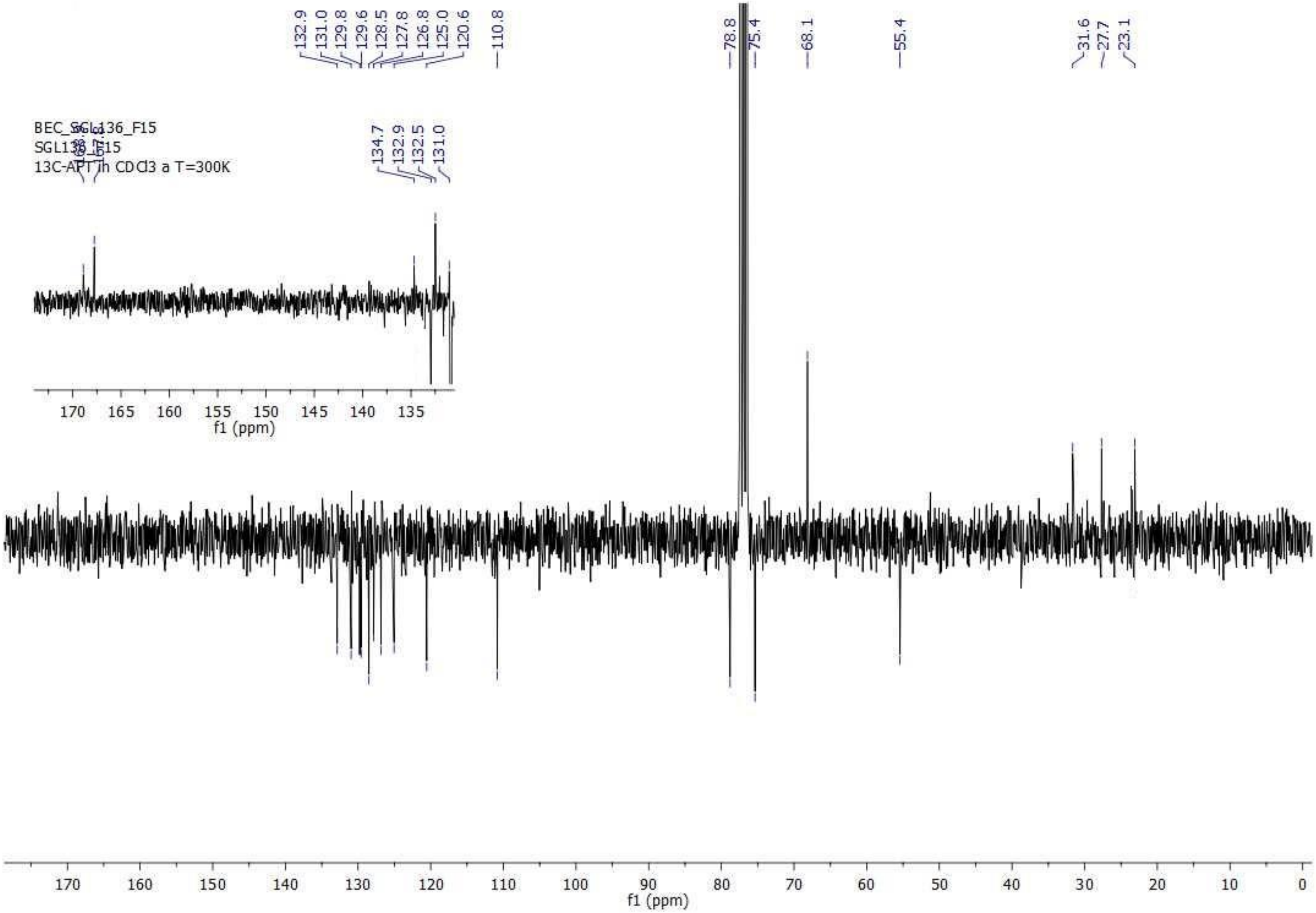

BEC_SGL136_F15
SGL136_F15
13C-APT in CDCl3 a T=300K
132.9
131.0
129.8
129.6
128.5
127.8
126.8
125.0
120.6
110.8
78.8
75.4
68.1
55.4
31.6
27.7
23.1
134.7
132.9
132.5
131.0
170 165 160 155 150 145 140 135
f1 (ppm)
170 160 150 140 130 120 110 100 90 80 70 60 50 40 30 20 10 0
f1 (ppm)

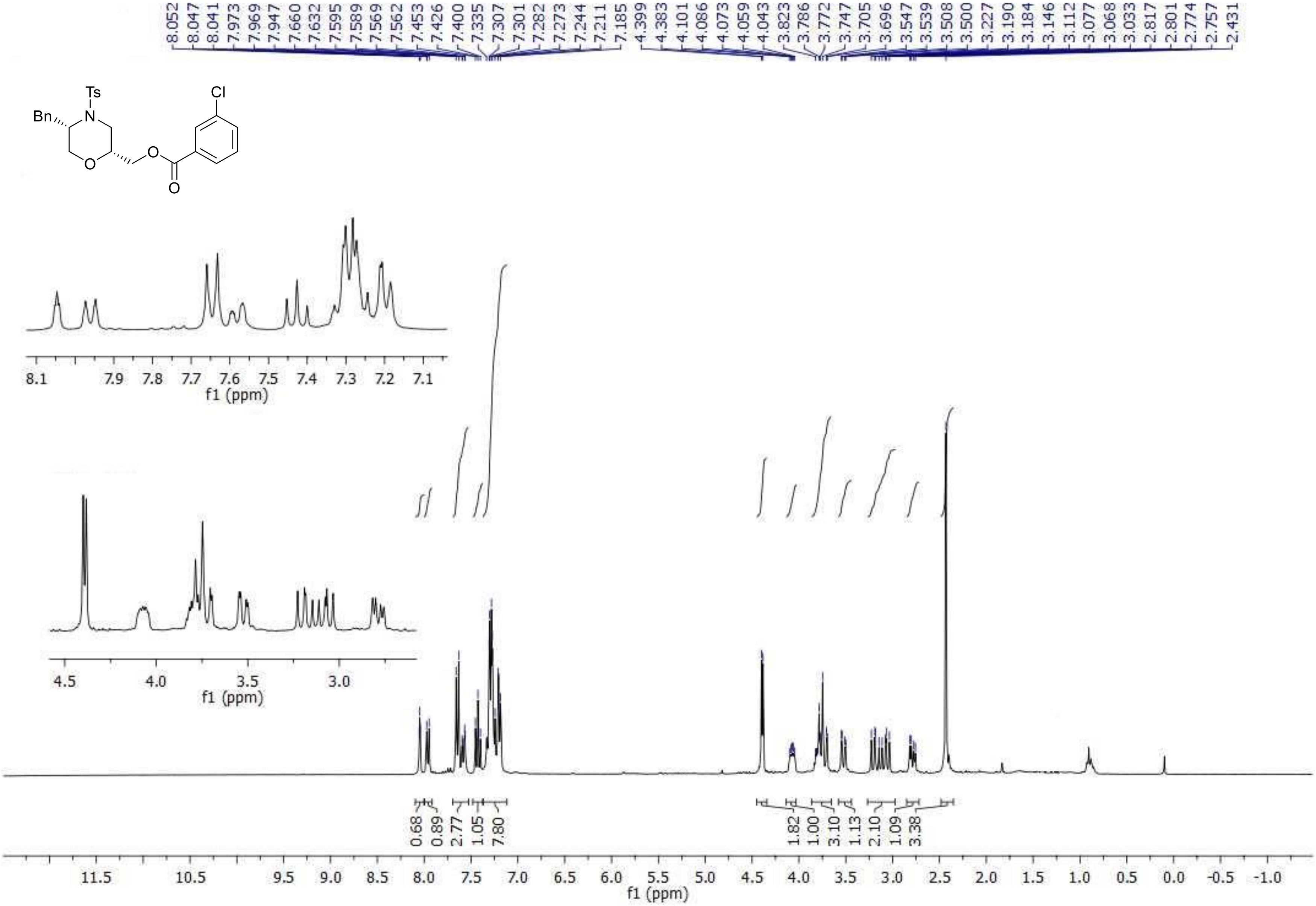

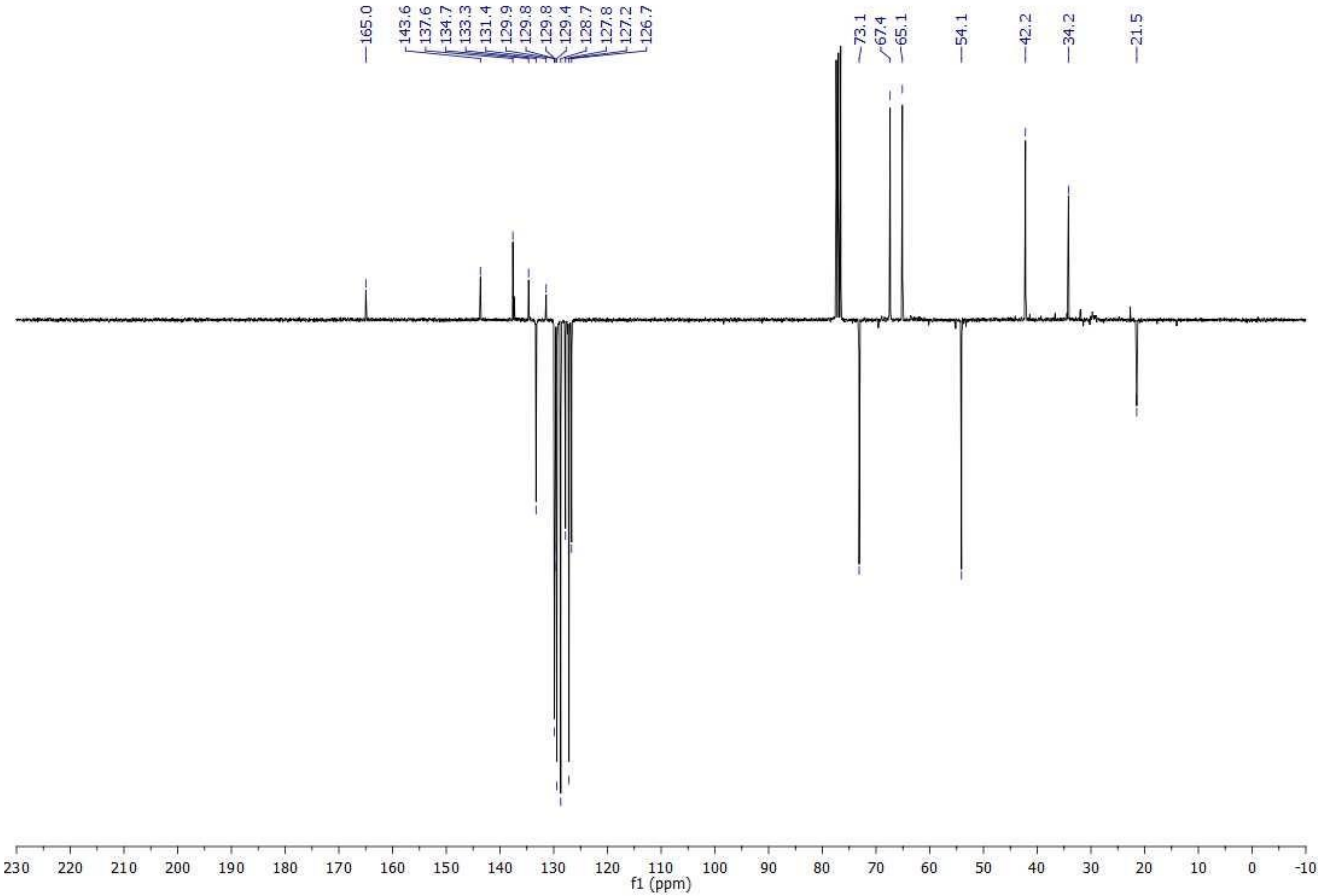
165.0
143.6
137.6
134.7
133.3
131.4
129.9
129.8
129.8
129.4
128.7
127.8
127.2
126.7
73.1
67.4
65.1
54.1
42.2
34.2
21.5
230 220 210 200 190 180 170 160 150 140 130 120 110 100 90 80 70 60 50 40 30 20 10 0 -10
f1 (ppm)

**COSY NMR**

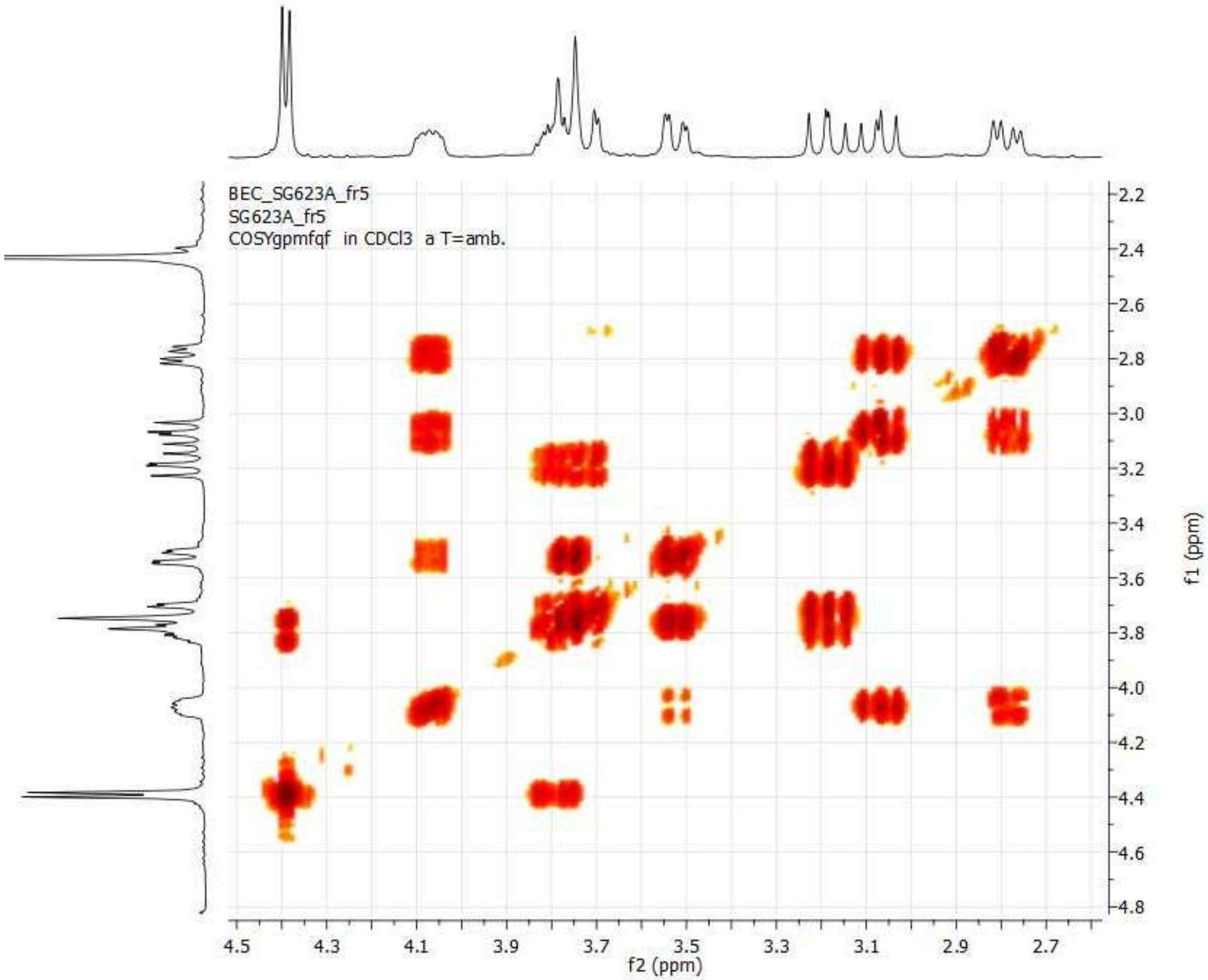

BEC_SG623A_fr5
SG623A_fr5
COSYgpmfqf in CDCl3 a T=amb.
f2 (ppm)
f1 (ppm)

**NOESY NMR**

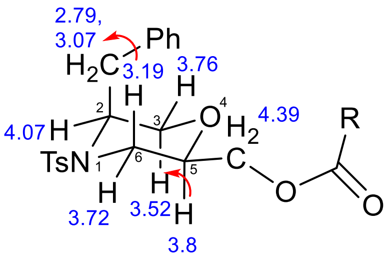
2.79,
3.07
Ph
$H_2C$
3.19
3.76
H
H
O
4
4.39
$H_2$
R
4.07 H
2
3
TsN
1
6
5
C
O
O
H
H
H
3.72
3.52
3.8

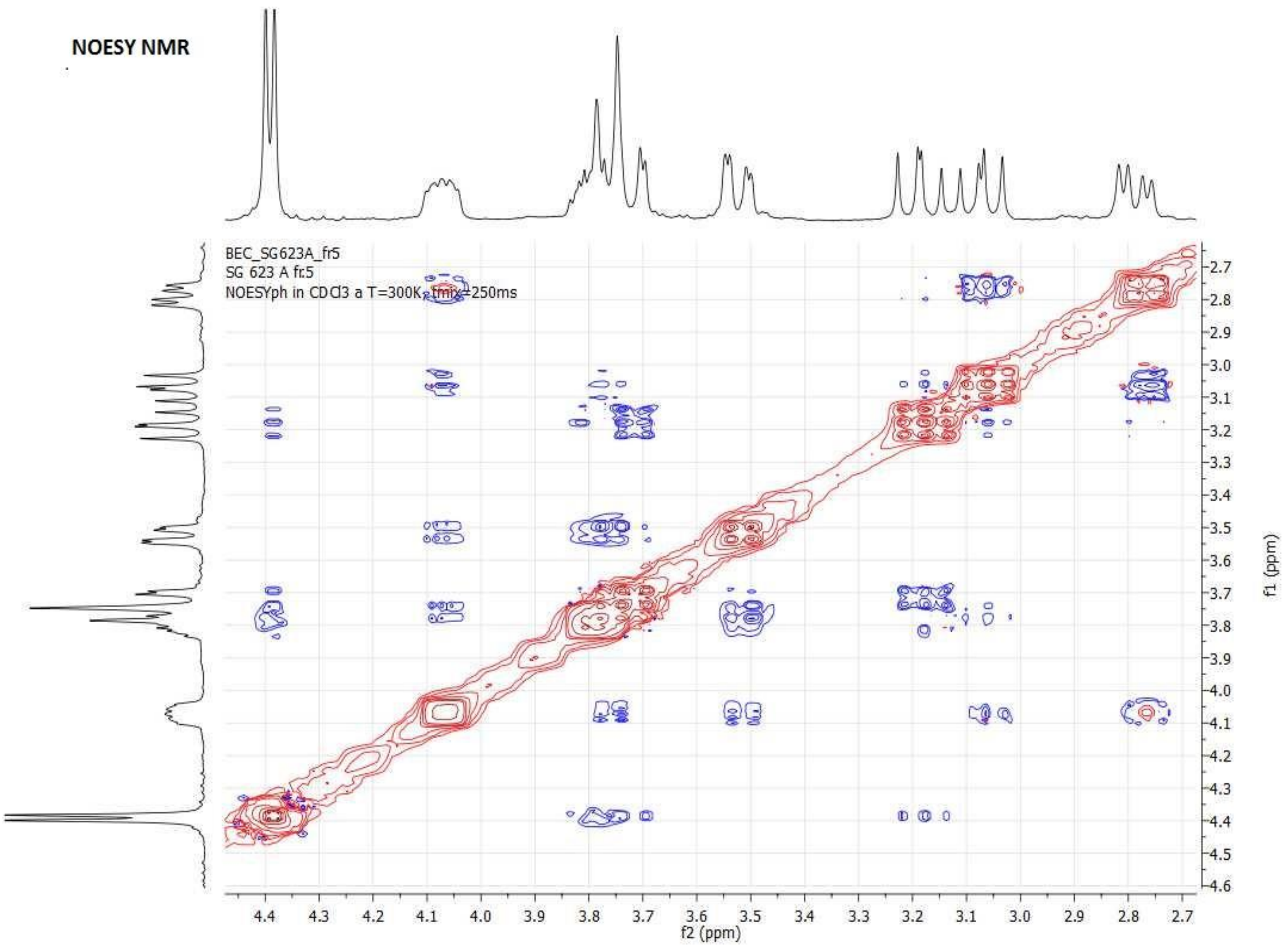
BEC_SG623A_fr5
SG 623 A fr.5
NOESYph in CDCl3 a T=300K, tmix=250ms
f2 (ppm)
f1 (ppm)

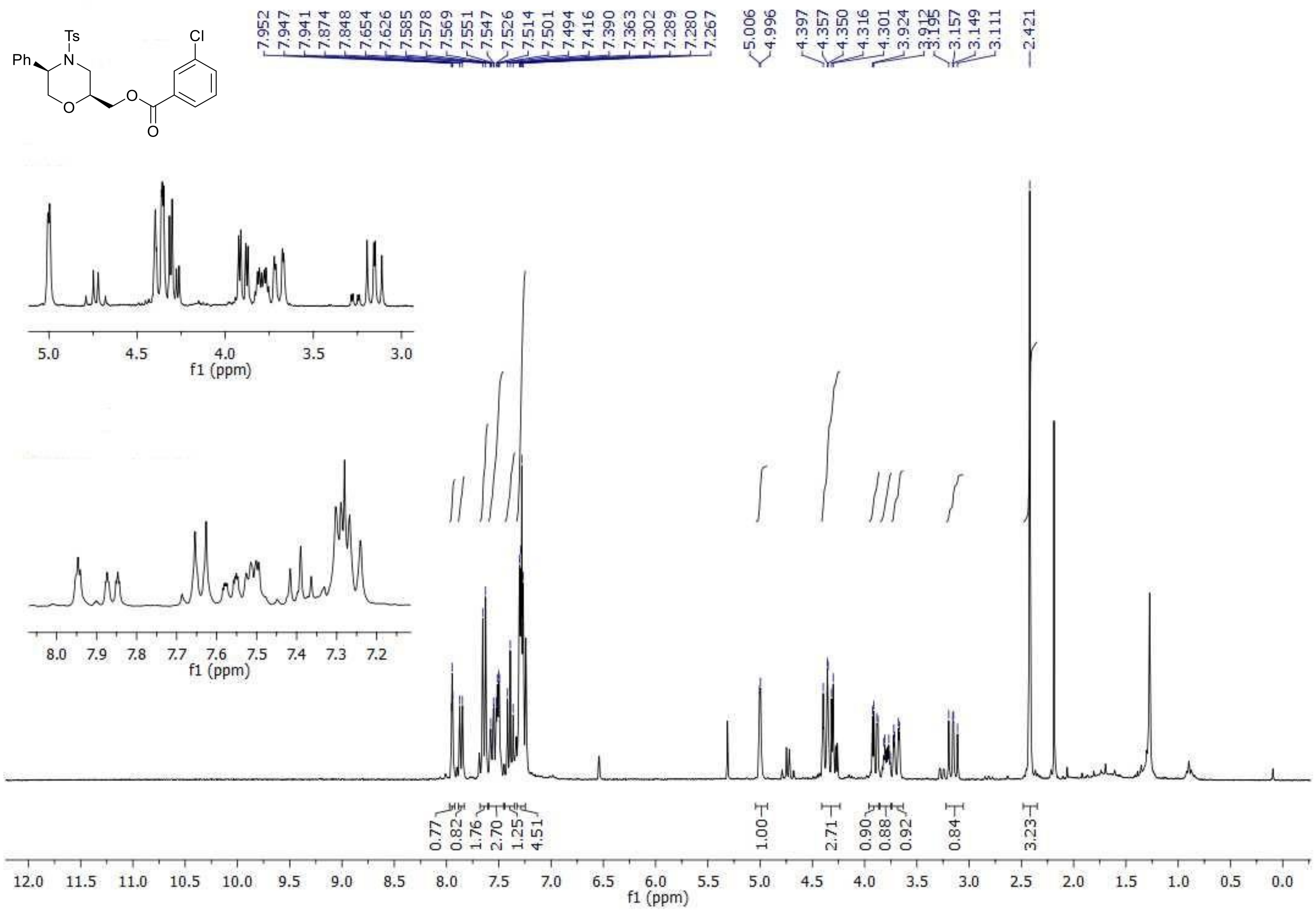

Ph
Ts
N
O
O
O
Cl
7.952 7.947 7.941 7.874 7.848 7.654 7.626 7.585 7.578 7.569 7.551 7.547 7.526 7.514 7.501 7.494 7.416 7.390 7.363 7.302 7.289 7.280 7.267
5.006 4.996
4.397 4.357 4.350 4.316 4.301 3.924 3.912 3.195 3.157 3.149 3.111
2.421
0.77 0.82 1.76 2.70 1.25 4.51 1.00 2.71 0.90 0.88 0.92 0.84 3.23
f1 (ppm)

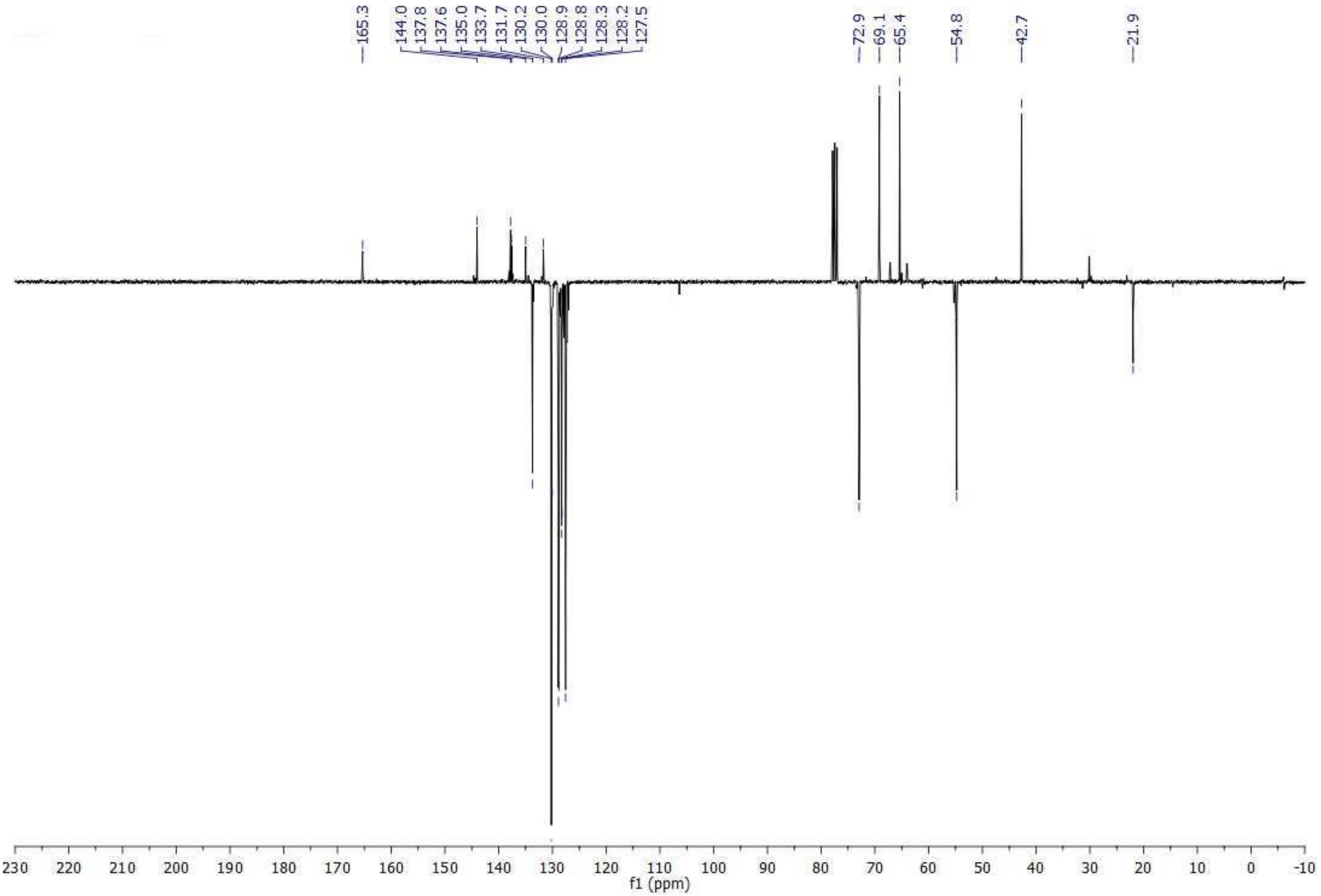

165.3
144.0
137.8
137.6
135.0
133.7
131.7
130.2
130.0
128.9
128.8
128.3
128.2
127.5
72.9
69.1
65.4
54.8
42.7
21.9
230 220 210 200 190 180 170 160 150 140 130 120 110 100 90 80 70 60 50 40 30 20 10 0 -10
f1 (ppm)

NMR COSY

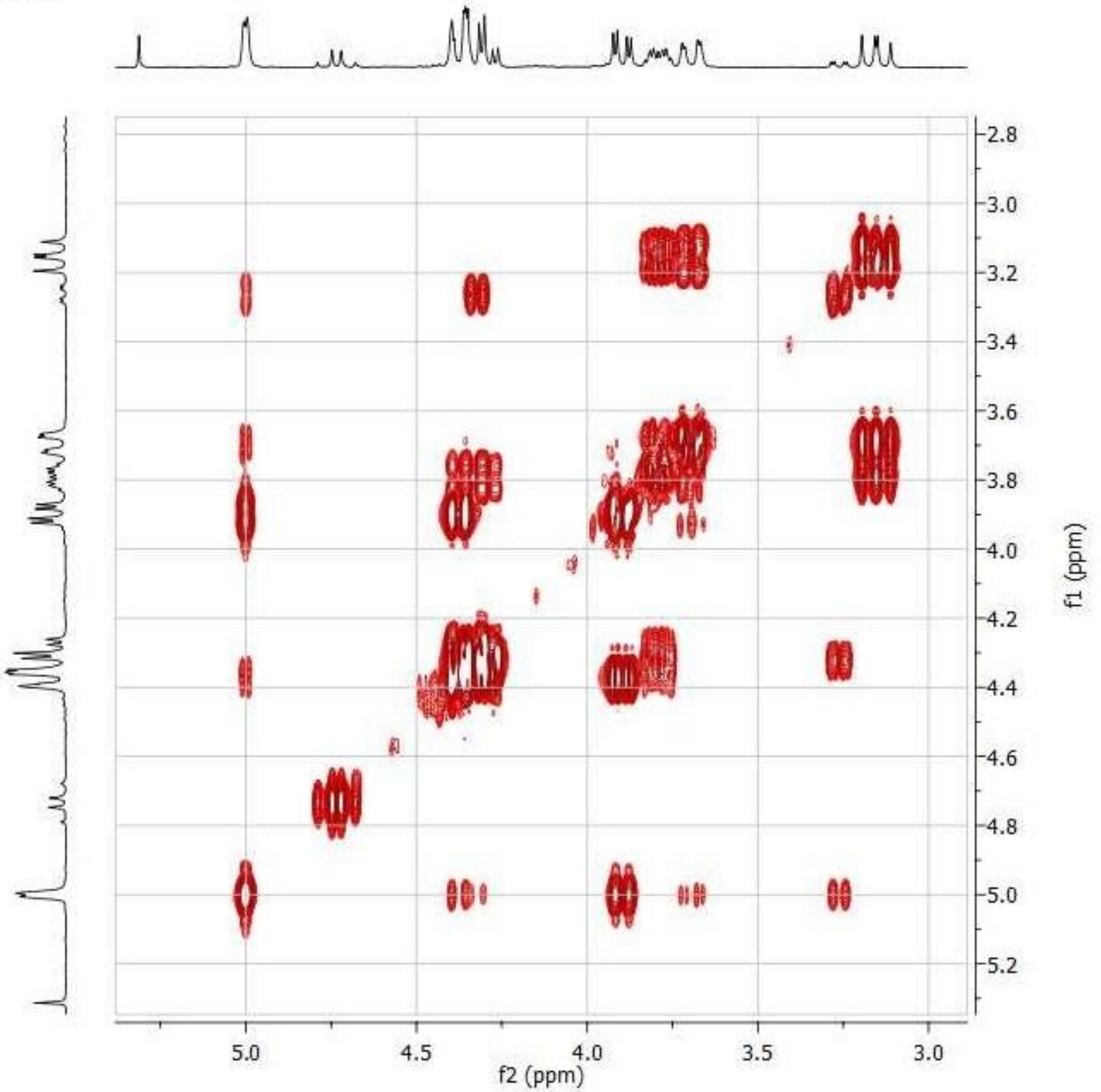

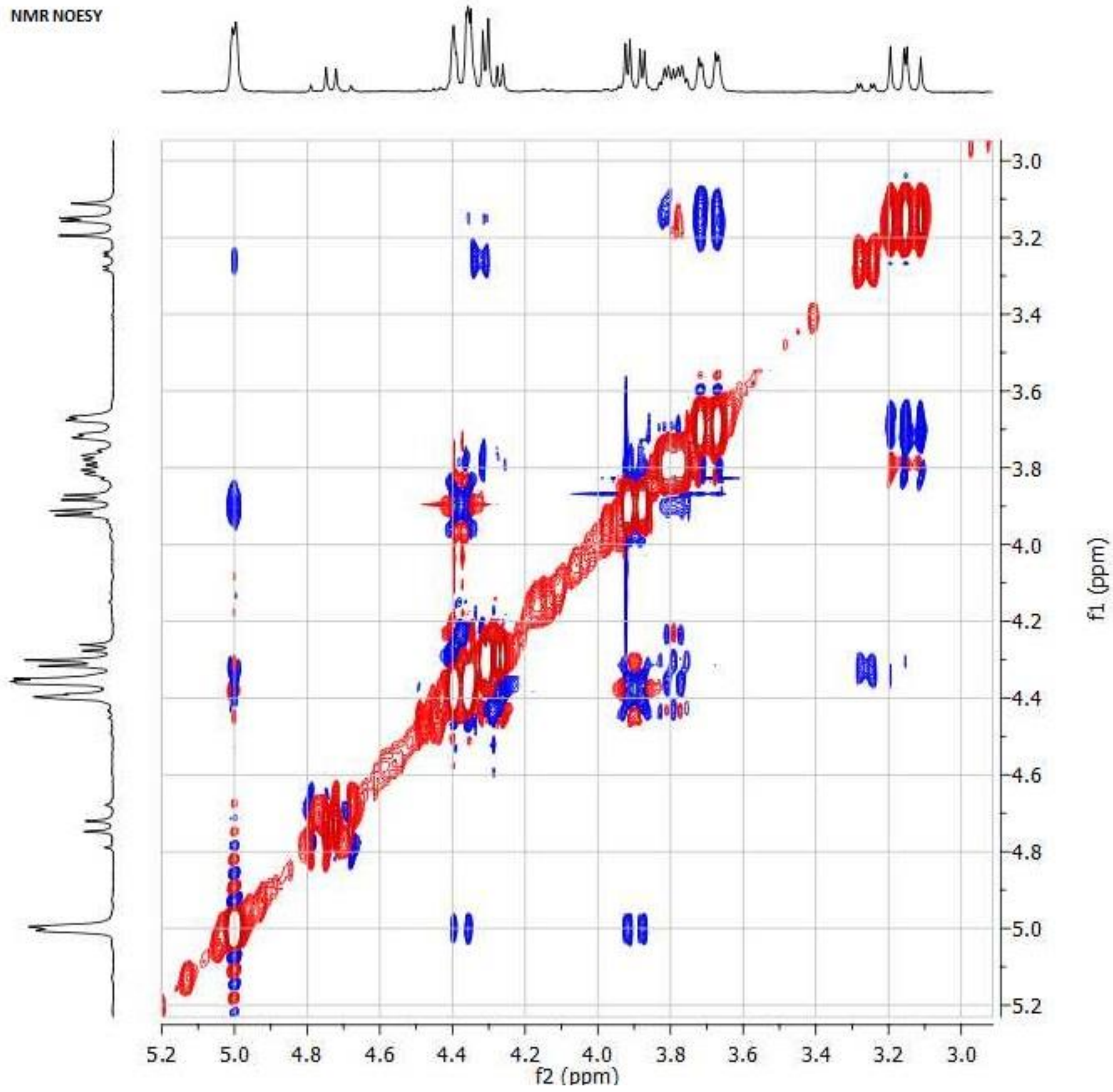
NMR NOESY
f1 (ppm)
f2 (ppm)

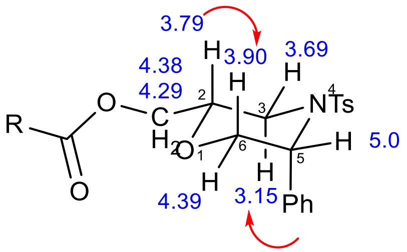
3.79
3.90
3.69
4.38
4.29
5.0
4.39
3.15
R
O
NTs
Ph

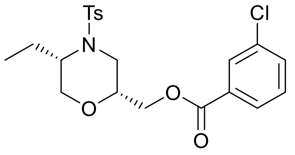
Ts
N
Cl
O
O
O

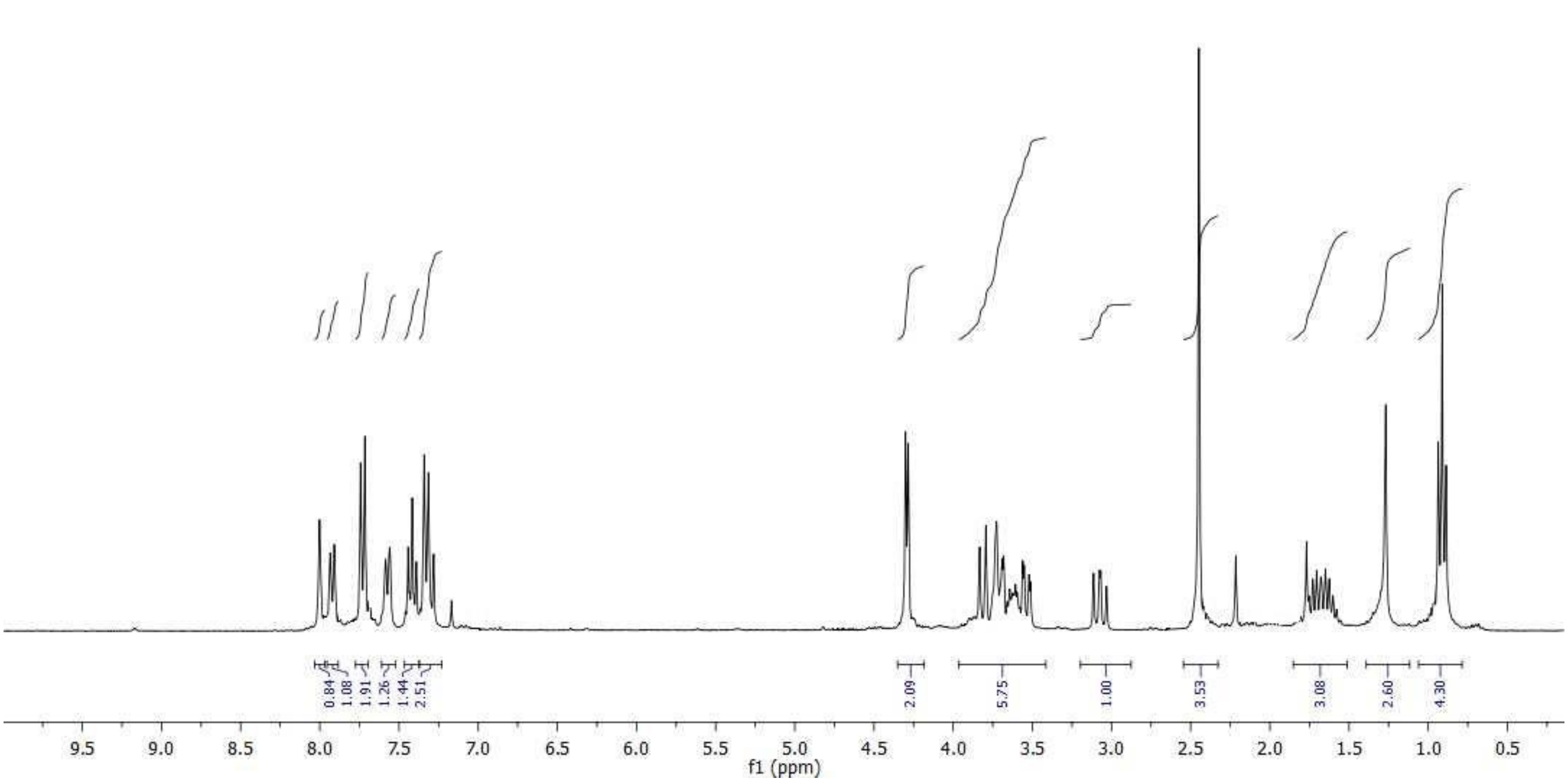
8.008
8.002
7.995
7.939
7.935
7.929
7.914
7.909
7.904
7.743
7.716
7.590
7.586
7.580
7.563
7.560
7.556
7.552
7.442
7.416
7.390
7.341
7.314
7.281
4.301
4.285
3.833
3.794
3.726
3.692
3.681
3.561
3.076
3.068
3.031
2.449
2.216
1.769
1.731
1.705
1.680
1.650
1.269
0.965
0.937
0.912
0.887
0.84
1.08
1.91
1.26
1.44
2.51
2.09
5.75
1.00
3.53
3.08
2.60
4.30
9.5
9.0
8.5
8.0
7.5
7.0
6.5
6.0
5.5
5.0
4.5
4.0
3.5
3.0
2.5
2.0
1.5
1.0
0.5
f1 (ppm)

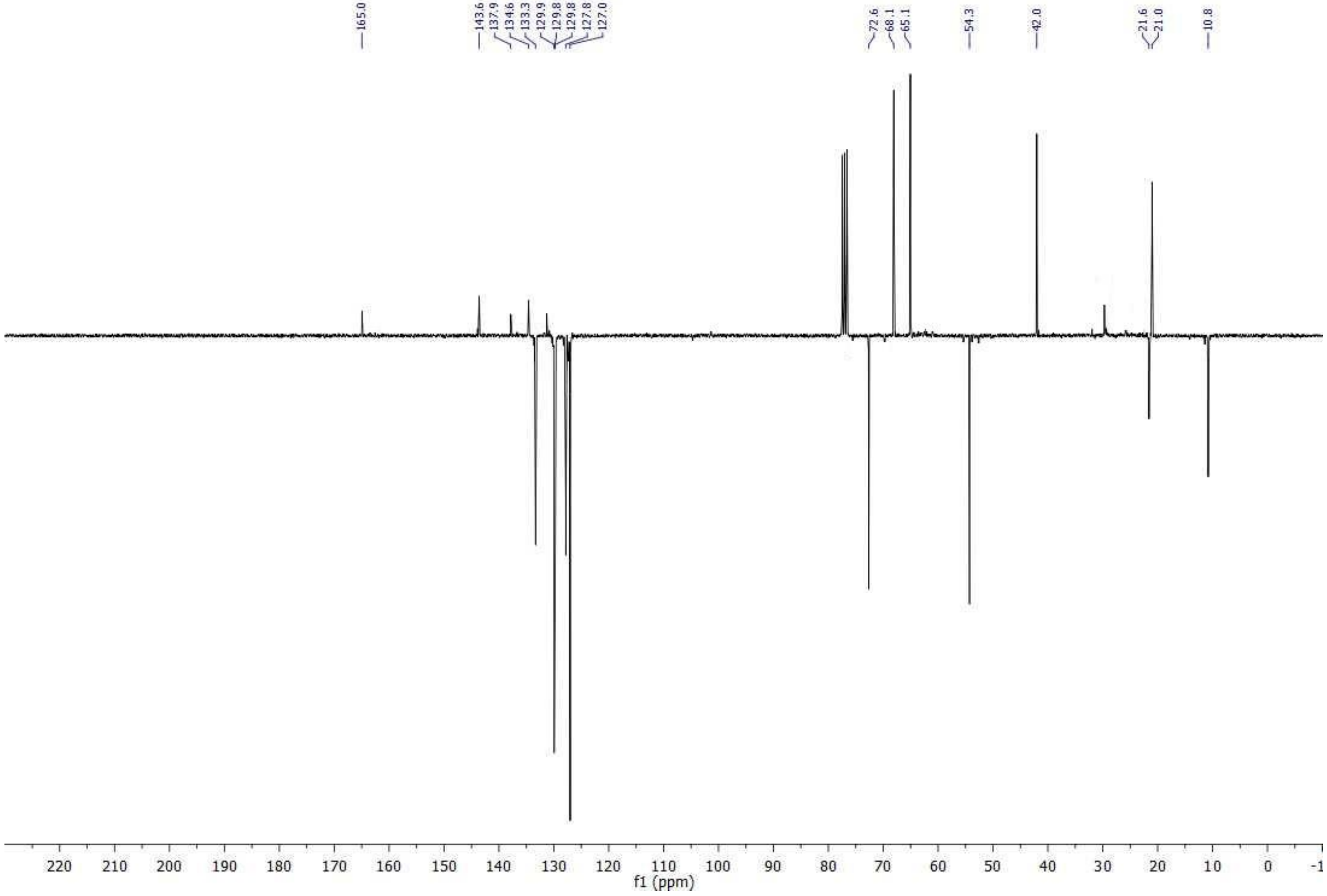
165.0
143.6
137.9
134.6
133.3
129.9
129.8
129.8
127.8
127.0
72.6
68.1
65.1
54.3
42.0
21.6
21.0
10.8
220 210 200 190 180 170 160 150 140 130 120 110 100 90 80 70 60 50 40 30 20 10 0 -1
f1 (ppm)

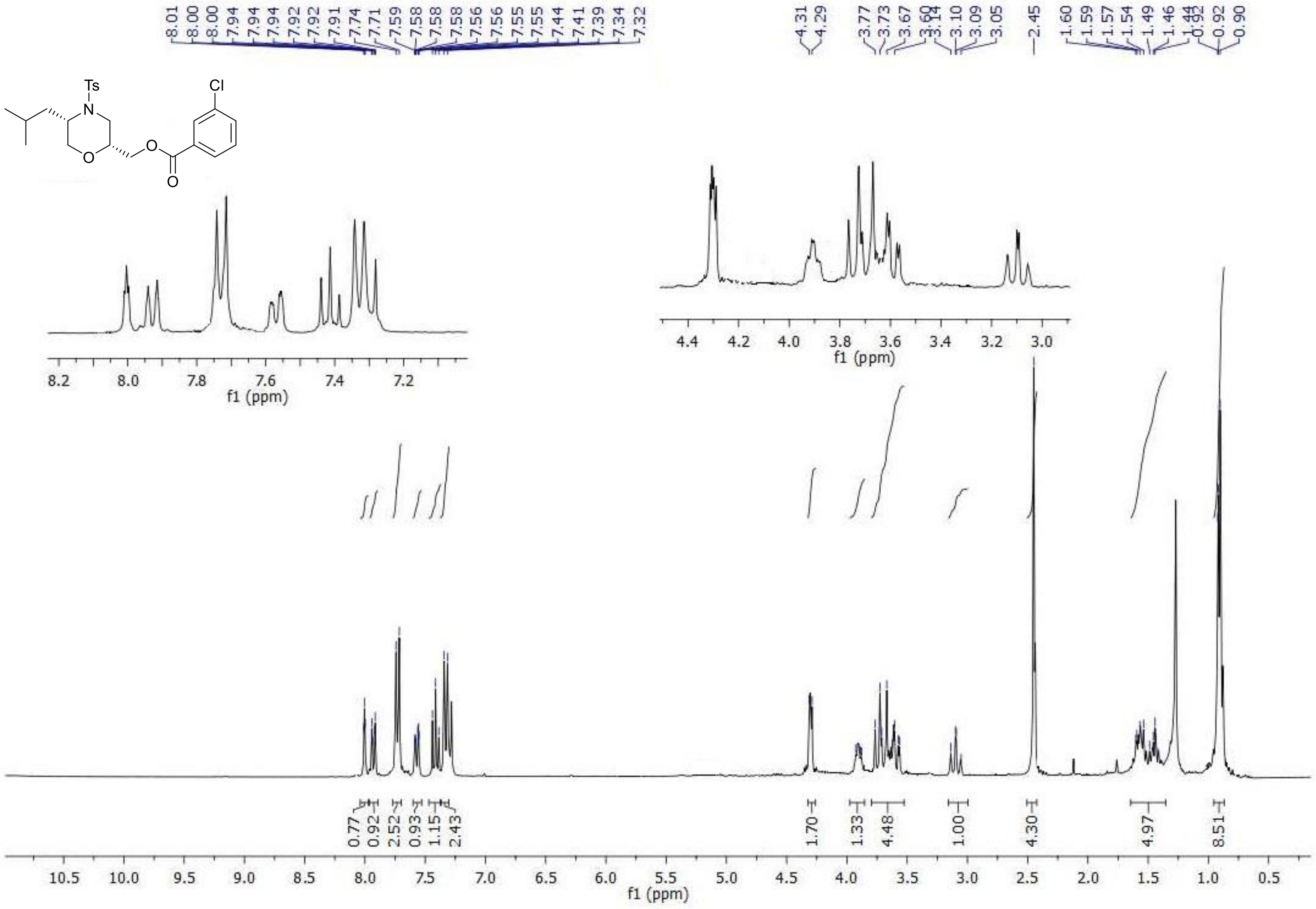
8.01 8.00 8.00 7.94 7.94 7.94 7.92 7.92 7.91 7.74 7.71 7.59 7.58 7.58 7.58 7.56 7.56 7.55 7.55 7.44 7.41 7.39 7.34 7.32
4.31 4.29 3.77 3.73 3.67 3.60 3.14 3.10 3.09 3.05 2.45 1.60 1.59 1.57 1.54 1.49 1.46 1.44 0.92 0.92 0.90
Ts
N
O
Cl
O
O
f1 (ppm)
0.77 0.92 2.52 0.93 1.15 2.43 1.70 1.33 4.48 1.00 4.30 4.97 8.51

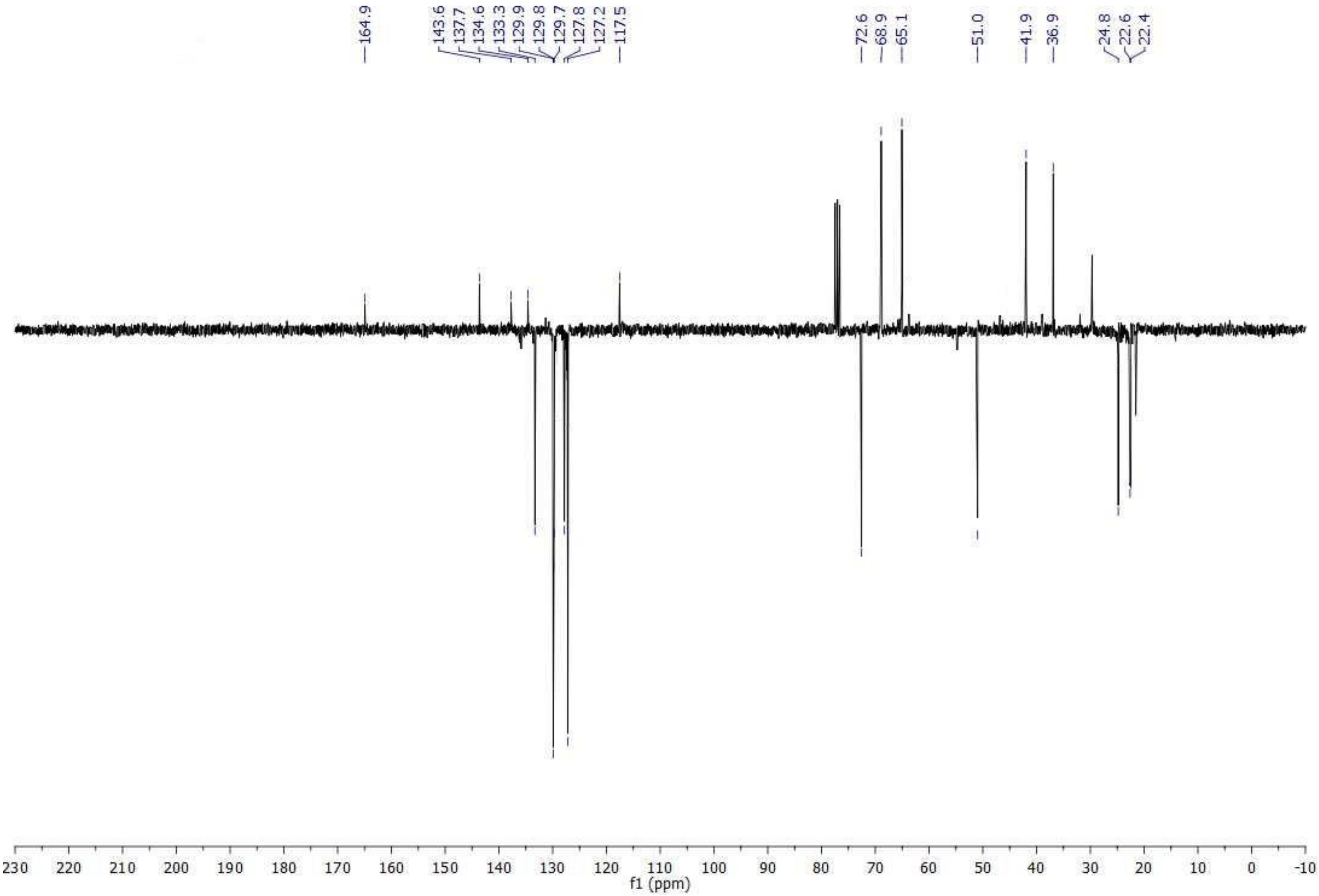

164.9
143.6
137.7
134.6
133.3
129.9
129.8
129.7
127.8
127.2
117.5
72.6
68.9
65.1
51.0
41.9
36.9
24.8
22.6
22.4
230 220 210 200 190 180 170 160 150 140 130 120 110 100 90 80 70 60 50 40 30 20 10 0 -10
f1 (ppm)

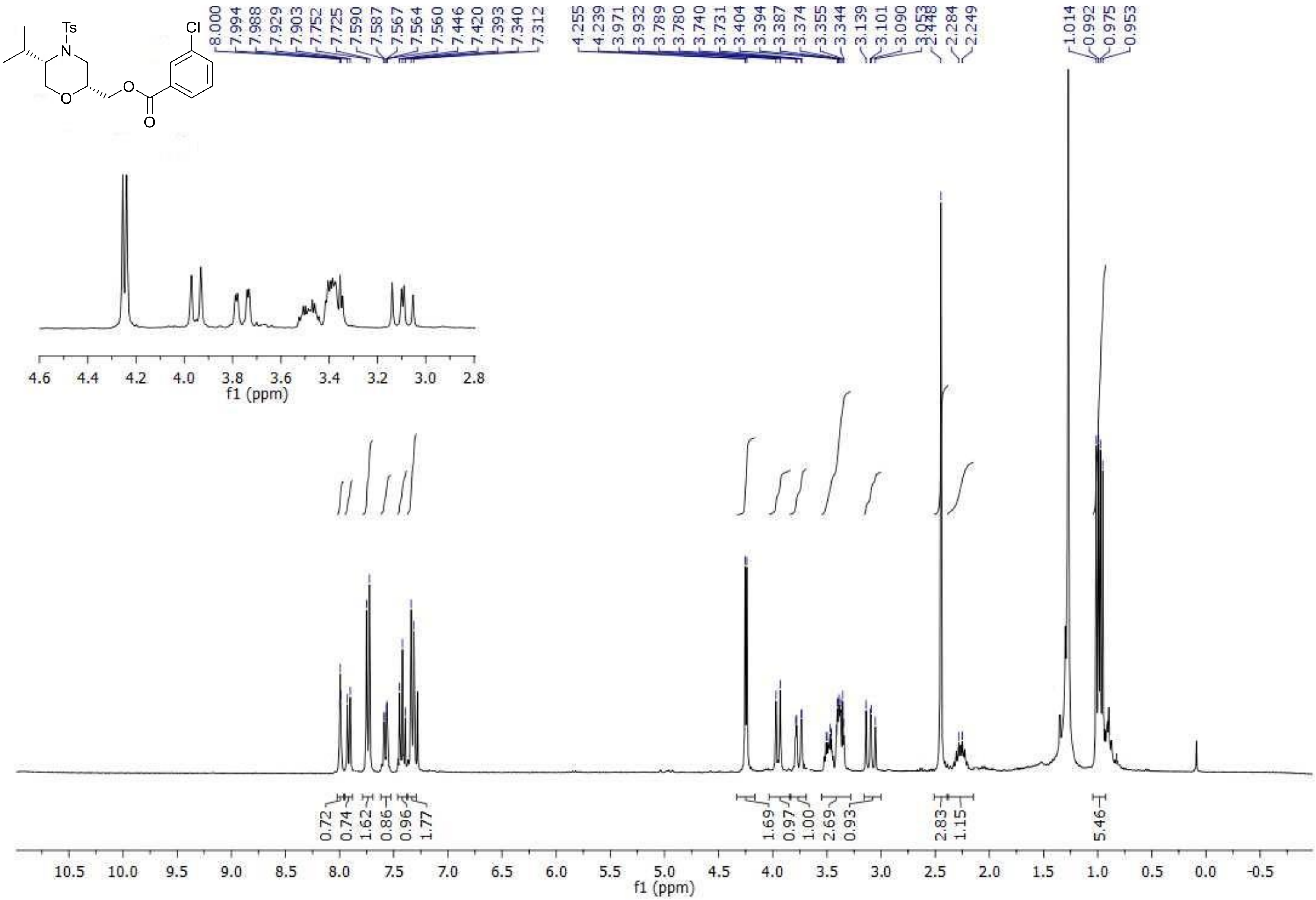

Ts
N
O
O
O
Cl
8.000
7.994
7.988
7.929
7.903
7.752
7.725
7.590
7.587
7.567
7.564
7.560
7.446
7.420
7.393
7.340
7.312
4.255
4.239
3.971
3.932
3.789
3.780
3.740
3.731
3.404
3.394
3.387
3.374
3.355
3.344
3.139
3.101
3.090
3.053
2.448
2.284
2.249
1.014
0.992
0.975
0.953
4.6 4.4 4.2 4.0 3.8 3.6 3.4 3.2 3.0 2.8
f1 (ppm)
0.72
0.74
1.62
0.86
0.96
1.77
1.69
0.97
1.00
2.69
0.93
2.83
1.15
5.46
10.5 10.0 9.5 9.0 8.5 8.0 7.5 7.0 6.5 6.0 5.5 5.0 4.5 4.0 3.5 3.0 2.5 2.0 1.5 1.0 0.5 0.0 -0.5
f1 (ppm)

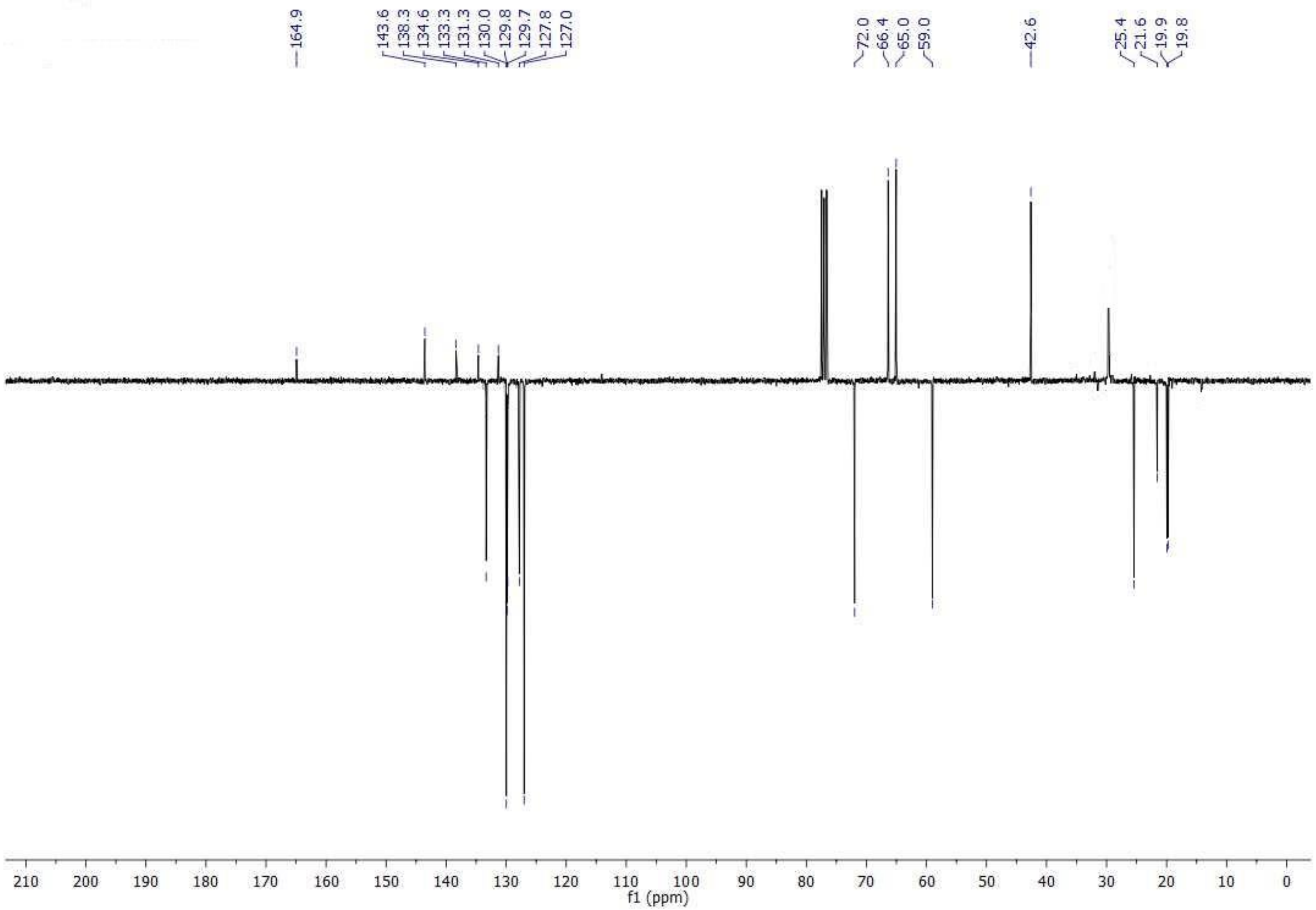
164.9
143.6
138.3
134.6
133.3
131.3
130.0
129.8
129.7
127.8
127.0
72.0
66.4
65.0
59.0
42.6
25.4
21.6
19.9
19.8
210 200 190 180 170 160 150 140 130 120 110 100 90 80 70 60 50 40 30 20 10 0
f1 (ppm)

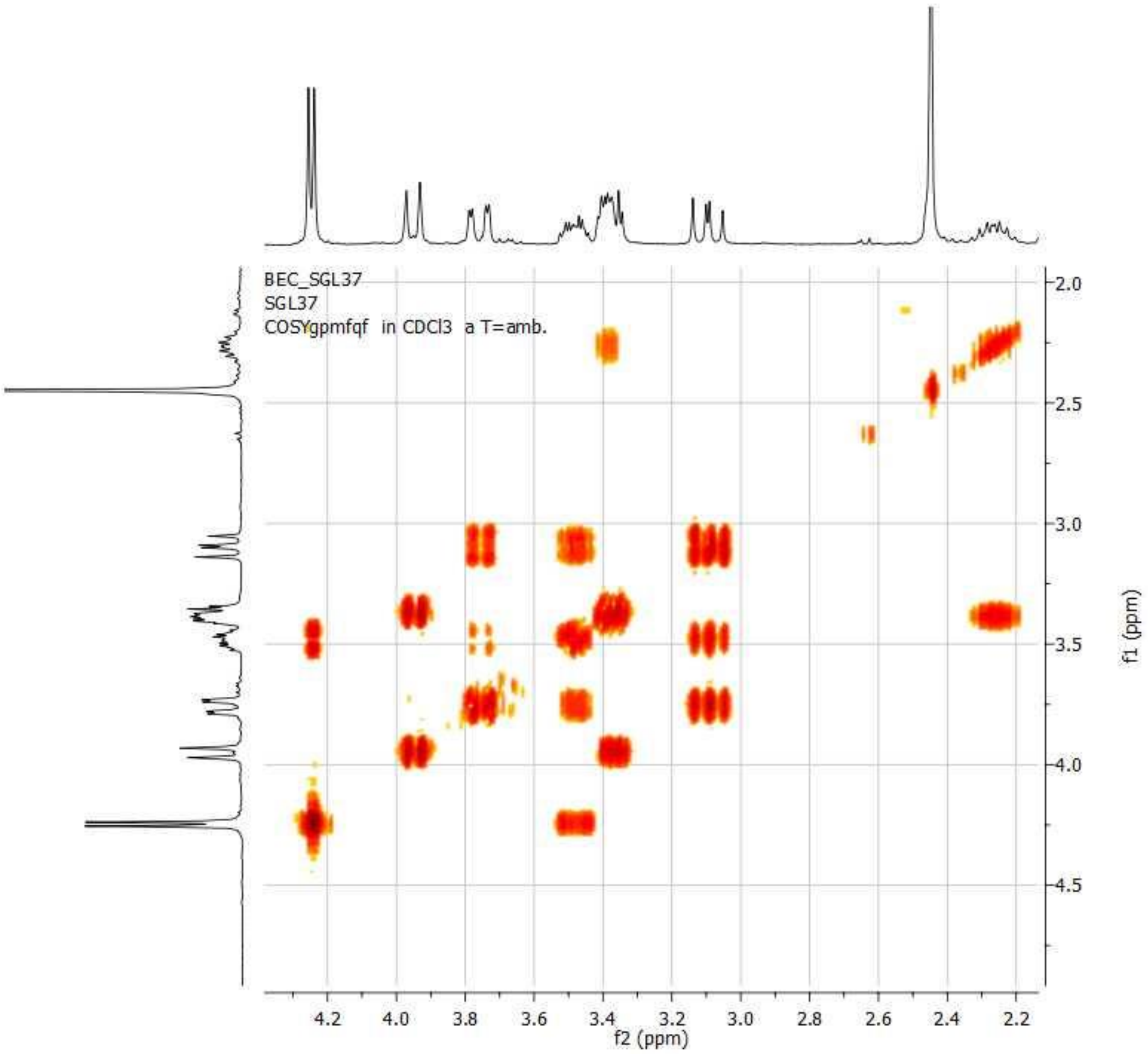
BEC_SGL37
SGL37
COSYgpmfqf in CDCl3 a T=amb.
f2 (ppm)
f1 (ppm)
4.2 4.0 3.8 3.6 3.4 3.2 3.0 2.8 2.6 2.4 2.2
2.0 2.5 3.0 3.5 4.0 4.5

ù

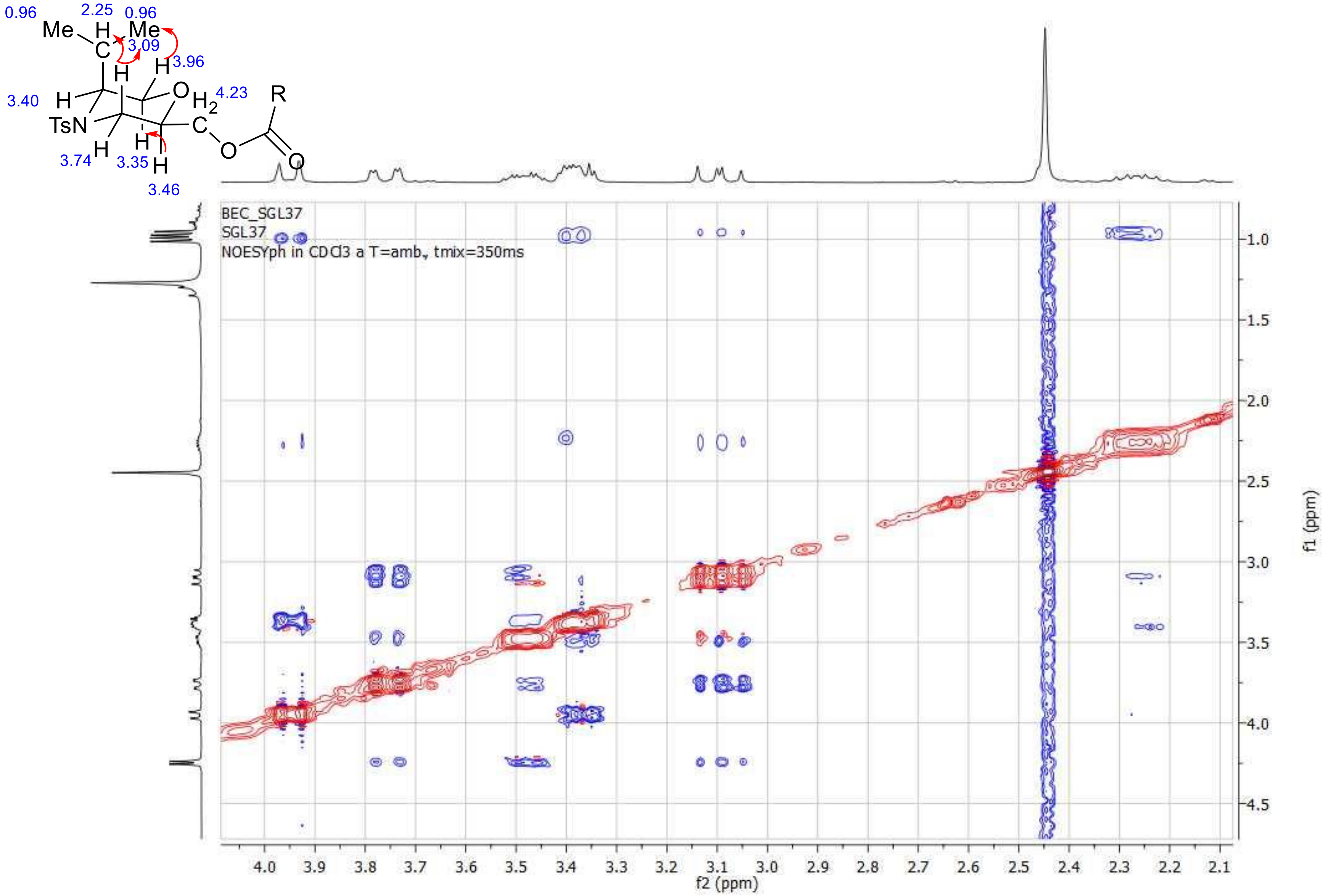

BEC_SGL37
SGL37
NOESYph in CDCl3 a T=amb., tmix=350ms
f2 (ppm)
f1 (ppm)
Me
Me
TsN
O
R
0.96
2.25
0.96
3.09
3.96
3.40
4.23
3.74
3.35
3.46

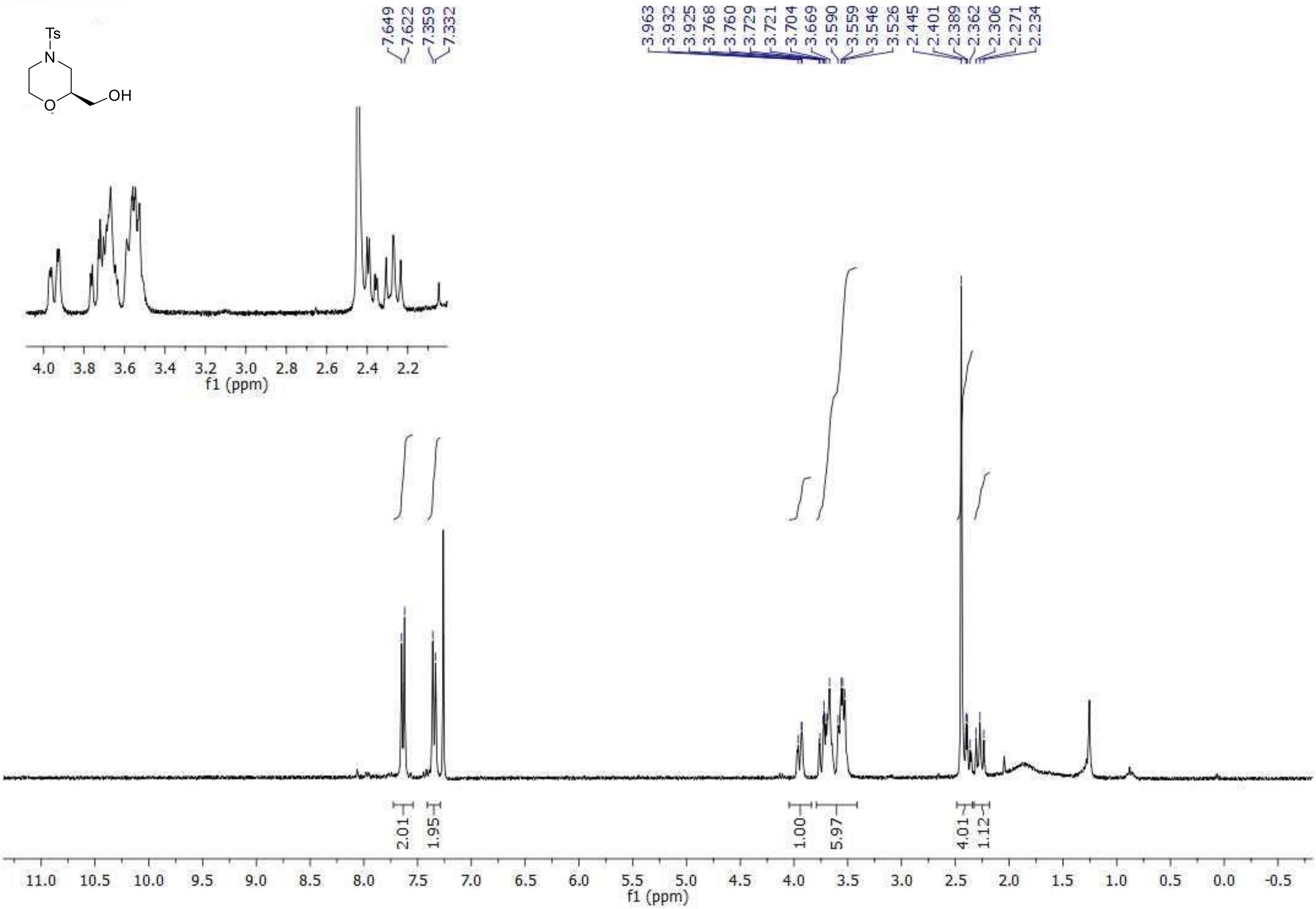
Ts
N
O
OH
7.649
7.622
7.359
7.332
3.963
3.932
3.925
3.768
3.760
3.729
3.721
3.704
3.669
3.590
3.559
3.546
3.526
2.445
2.401
2.389
2.362
2.306
2.271
2.234
4.0 3.8 3.6 3.4 3.2 3.0 2.8 2.6 2.4 2.2
f1 (ppm)
2.01
1.95
1.00
5.97
4.01
1.12
11.0 10.5 10.0 9.5 9.0 8.5 8.0 7.5 7.0 6.5 6.0 5.5 5.0 4.5 4.0 3.5 3.0 2.5 2.0 1.5 1.0 0.5 0.0 -0.5
f1 (ppm)

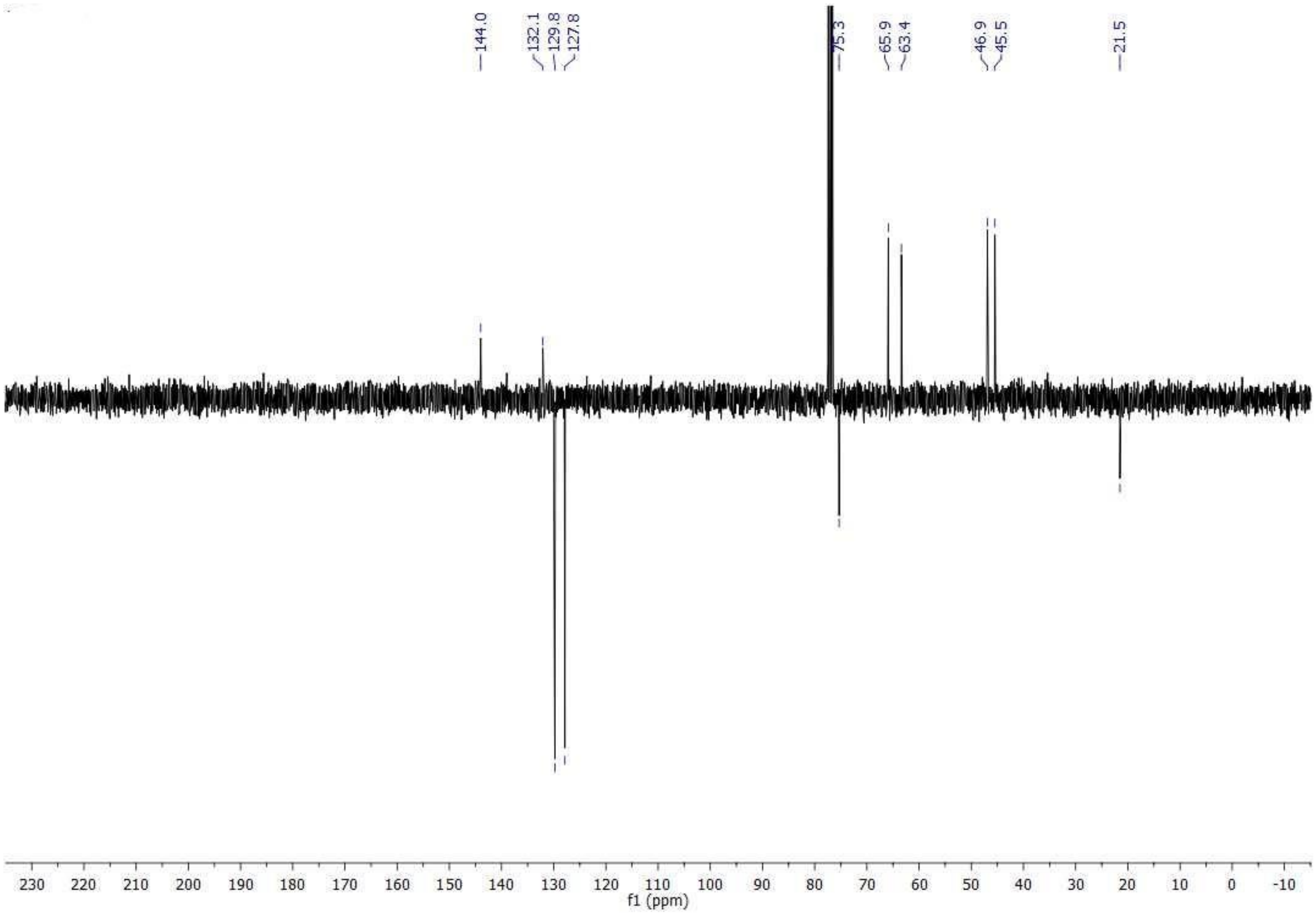
144.0
132.1
129.8
127.8
75.3
65.9
63.4
46.9
45.5
21.5
230
220
210
200
190
180
170
160
150
140
130
120
110
100
90
80
70
60
50
40
30
20
10
0
-10
f1 (ppm)

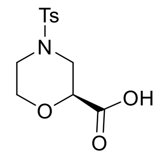

Ts
N
O
OH
O


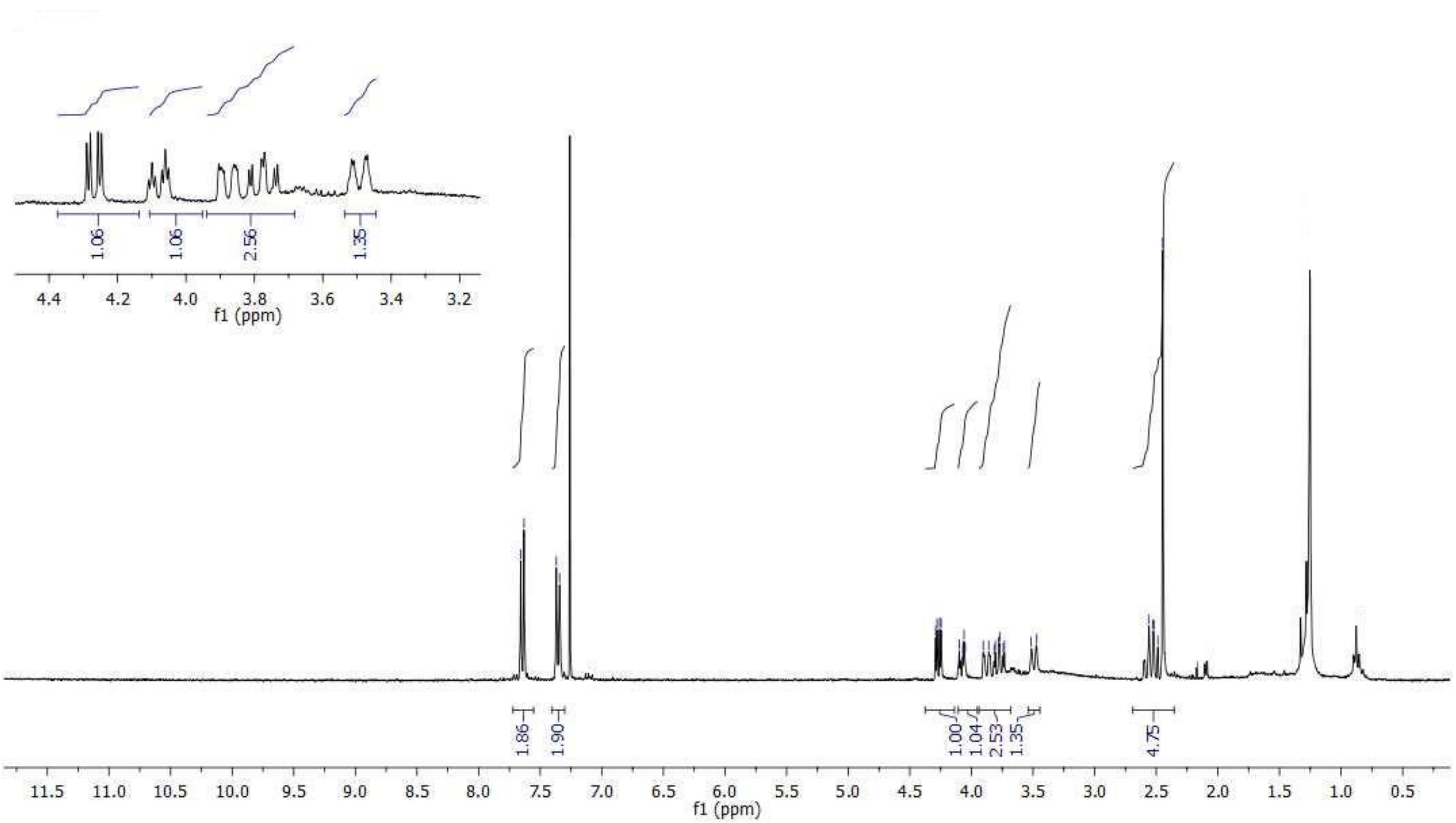

7.659
7.631
7.369
7.342
4.290
4.279
4.257
4.247
4.099
4.070
4.060
4.051
3.904
3.860
3.815
3.806
3.780
3.771
3.742
3.732
3.515
3.471
2.558
2.526
2.519
2.487
2.447
1.06
1.06
2.56
1.35
4.4 4.2 4.0 3.8 3.6 3.4 3.2
f1 (ppm)
1.86
1.90
1.00
1.04
2.53
1.35
4.75
11.5 11.0 10.5 10.0 9.5 9.0 8.5 8.0 7.5 7.0 6.5 6.0 5.5 5.0 4.5 4.0 3.5 3.0 2.5 2.0 1.5 1.0 0.5
f1 (ppm)

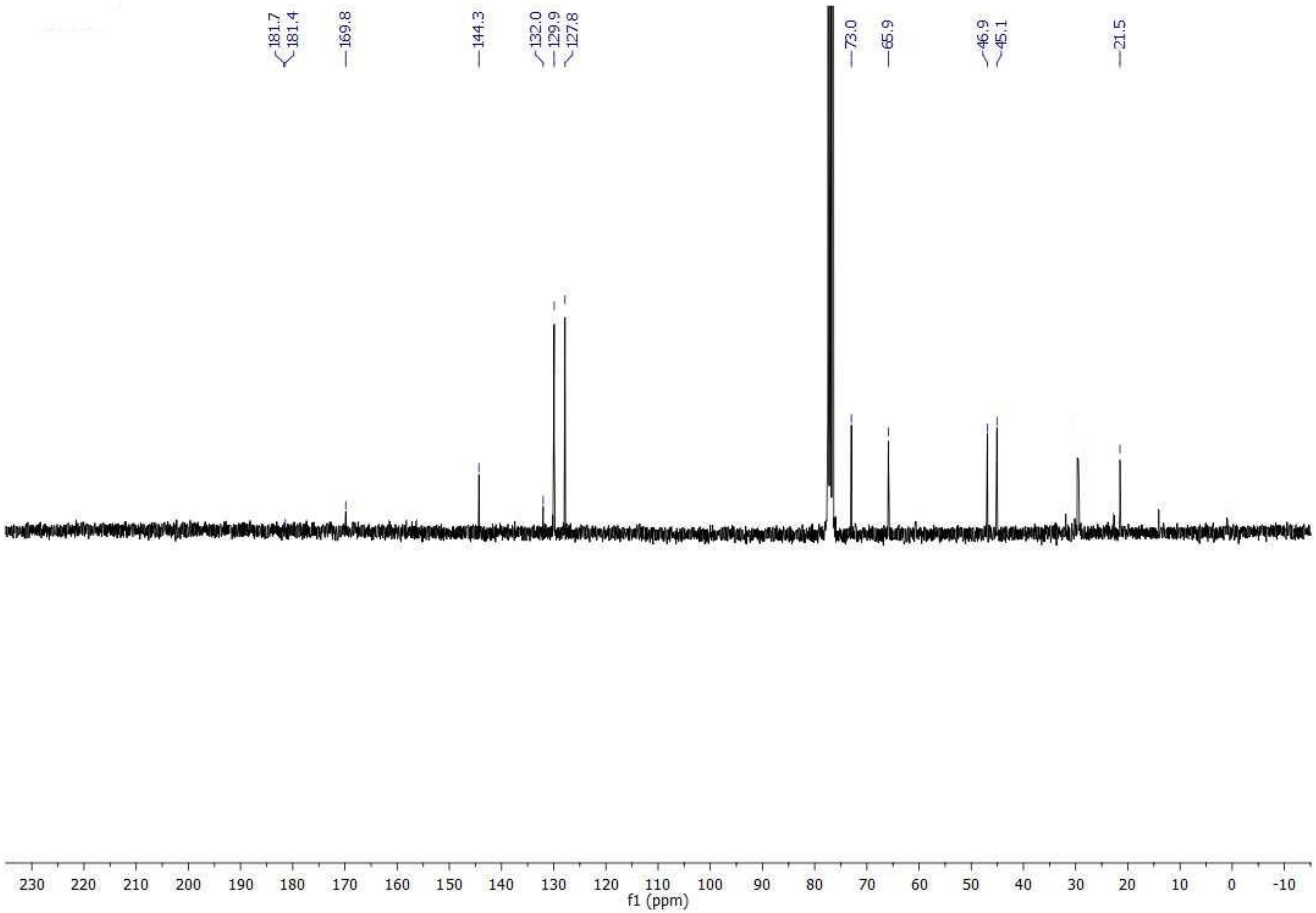
181.7
181.4
169.8
144.3
132.0
129.9
127.8
73.0
65.9
46.9
45.1
21.5
230 220 210 200 190 180 170 160 150 140 130 120 110 100 90 80 70 60 50 40 30 20 10 0 -10
f1 (ppm)

# HPLC data

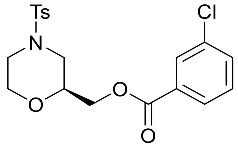


```
Injection Date  : 21/10/20 13.44.59
Sample Name     : SGmrac_amy2_5u30                    Vial :   1
Acq. Operator   : sabrina
Acq. Method     : C:\HPCHEM\1\METHODS\CECE.M
Last changed    : 21/10/20 13.48.29  by sabrina
                  (modified after loading)
Analysis Method : C:\HPCHEM\1\METHODS\CECE.M
Last changed    : 17/02/21 16.12.04  by sabrina
                  (modified after loading)
```

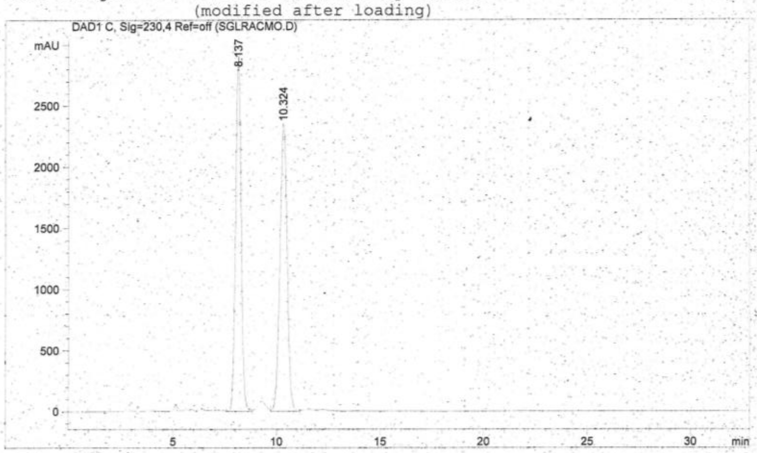


Area Percent Report

```
Sorted By          :      Signal
Multiplier         :      1.0000
Dilution           :      1.0000

Signal 1: DAD1 C, Sig=230,4 Ref=off
```

| Peak # | RetTime [min] | Type | Width [min] | Area [mAU*s] | Height [mAU] | Area % |
|---|---|---|---|---|---|---|
| 1 | 8.137 | VV | 0.2645 | 5.55765e4 | 2888.30518 | 49.2846 |
| 2 | 10.324 | VV | 0.3804 | 5.71900e4 | 2346.03271 | 50.7154 |

Totals : 1.12767e5 5234.33789

Results obtained with enhanced integrator!

```
Injection Date  : 17/02/21 16.33.13
Sample Name     : SG636zz_amy2_30                     Vial :   1
Acq. Operator   : sabrina
Method          : C:\HPCHEM\1\METHODS\CECE.M
Last changed    : 17/02/21 16.12.04  by sabrina
                  (modified after loading)
```

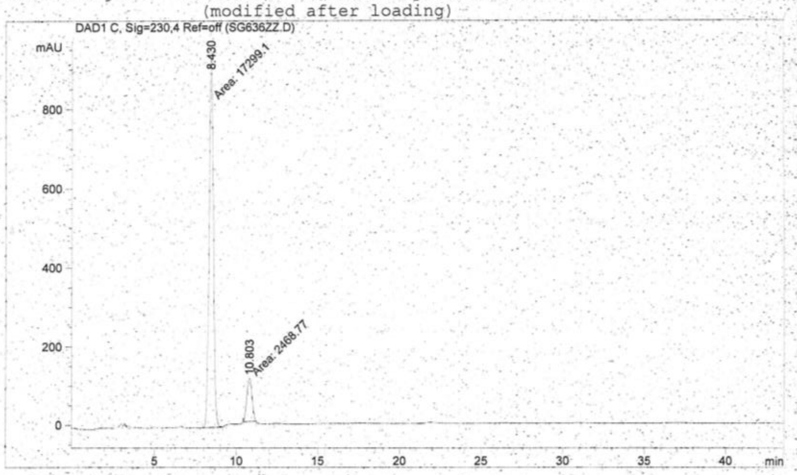


Area Percent Report

```
Sorted By          :      Signal
Multiplier         :      1.0000
Dilution           :      1.0000

Signal 1: DAD1 C, Sig=230,4 Ref=off
```

| Peak # | RetTime [min] | Type | Width [min] | Area [mAU*s] | Height [mAU] | Area % |
|---|---|---|---|---|---|---|
| 1 | 8.430 | MM | 0.3089 | 1.72991e4 | 933.50159 | 87.5112 |
| 2 | 10.803 | MM | 0.3734 | 2468.77124 | 110.19308 | 12.4888 |

Totals : 1.97679e4 1043.69467

Results obtained with enhanced integrator!

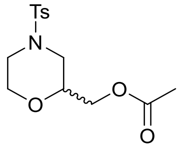


```
Injection Date   : 20/09/19 10.10.20
Sample Name      : sg 651r amy2 30i                    Vial :   1
Acq. Operator    : 1
Acq. Method      : C:\HPCHEM\1\METHODS\CECE.M
Last changed     : 18/09/19 13.01.00  by 1
                   (modified after loading)
Analysis Method : C:\HPCHEM\1\METHODS\CECE.M
Last changed     : 20/09/19 10.44.33  by 1
                   (modified after loading)
```

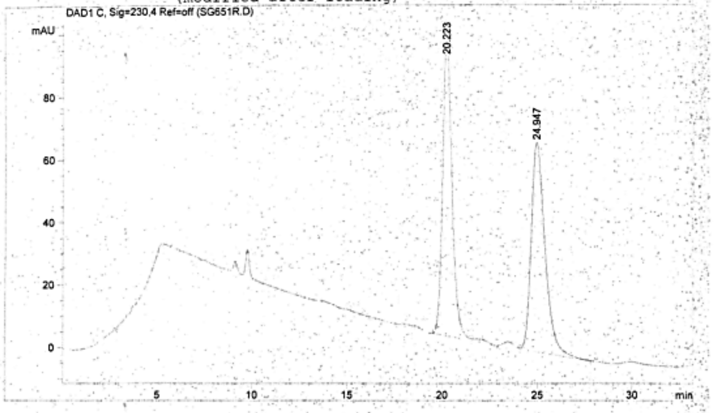


Area Percent Report

```
Sorted By              :      Signal
Multiplier             :      1.0000
Dilution               :      1.0000

Signal 1: DAD1 C, Sig=230,4 Ref=off
```

| Peak # | RetTime [min] | Type | Width [min] | Area [mAU*s] | Height [mAU] | Area % |
|---|---|---|---|---|---|---|
| 1 | 20.223 | PB | 0.5232 | 3262.40112 | 94.40825 | 49.5868 |
| 2 | 24.947 | VP | 0.7412 | 3316.77100 | 66.58188 | 50.4132 |
| Totals : | | | | 6579.17212 | 160.99013 | |

Results obtained with enhanced integrator!

```
Injection Date   : 20/09/19 10.46.58
Sample Name      : sg 651n amy2 30i                    Vial :   1
Acq. Operator    : 1
Method           : C:\HPCHEM\1\METHODS\CECE.M
Last changed     : 20/09/19 10.44.33  by 1
                   (modified after loading)
```

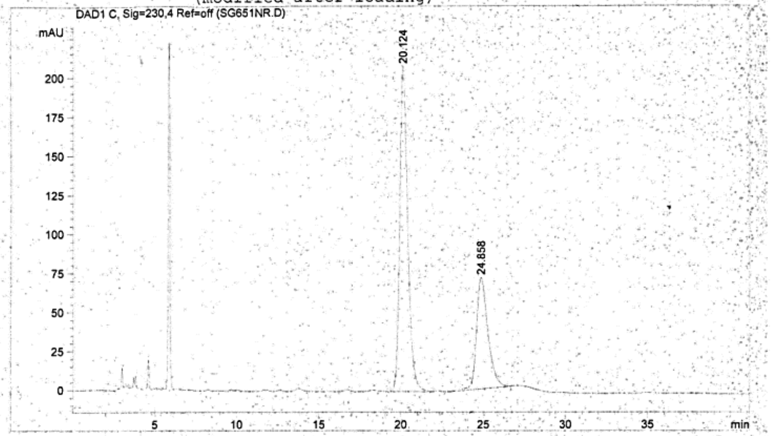


Area Percent Report

```
Sorted By              :      Signal
Multiplier             :      1.0000
Dilution               :      1.0000

Signal 1: DAD1 C, Sig=230,4 Ref=off
```

| Peak # | RetTime [min] | Type | Width [min] | Area [mAU*s] | Height [mAU] | Area % |
|---|---|---|---|---|---|---|
| 1 | 20.124 | PB | 0.5102 | 7080.59424 | 207.44821 | 66.9534 |
| 2 | 24.858 | VB | 0.7339 | 3494.81396 | 71.04444 | 33.0466 |
| Totals : | | | | 1.05754e4 | 278.49265 | |

Results obtained with enhanced integrator!

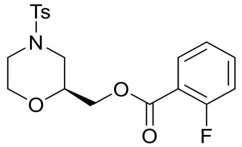


```
Injection Date  : 18/12/20 11.28.51
Sample Name     : SGL62r_amy2-5_30                    Vial :   1
Acq. Operator   : sabrina
Method          : C:\HPCHEM\1\METHODS\CECE.M
Last changed    : 14/02/20 15.40.47  by 1
```

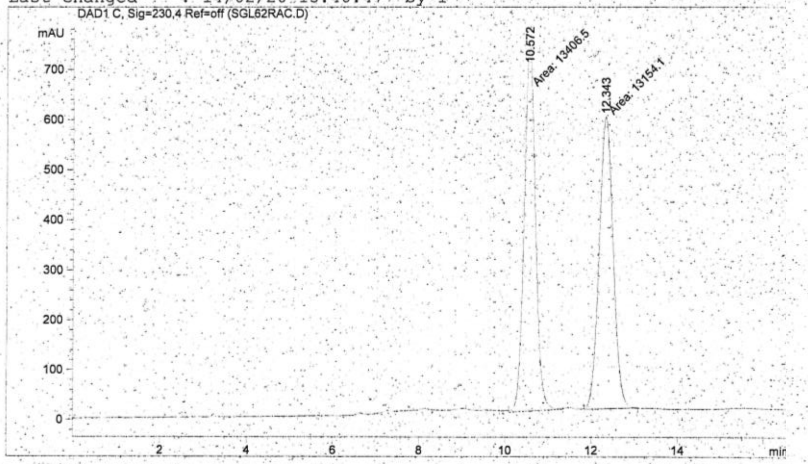


Area Percent Report

```
Sorted By          :      Signal
Multiplier         :      1.0000
Dilution           :      1.0000
```

Signal 1: DAD1 C, Sig=230,4 Ref=off

| Peak # | RetTime [min] | Type | Width [min] | Area [mAU*s] | Height [mAU] | Area % |
|---|---|---|---|---|---|---|
| 1 | 10.572 | MM | 0.3057 | 1.34065e4 | 730.81757 | 50.4752 |
| 2 | 12.343 | MM | 0.3761 | 1.31541e4 | 582.95972 | 49.5248 |
| Totals : | | | | 2.65605e4 | 1313.77728 | |

Results obtained with enhanced integrator!

```
Injection Date  : 18/12/20 11.46.33
Sample Name     : SGL62f_amy2-5_30                    Vial :   1
Acq. Operator   : sabrina
Method          : C:\HPCHEM\1\METHODS\CECE.M
Last changed    : 14/02/20 15.40.47  by 1
```

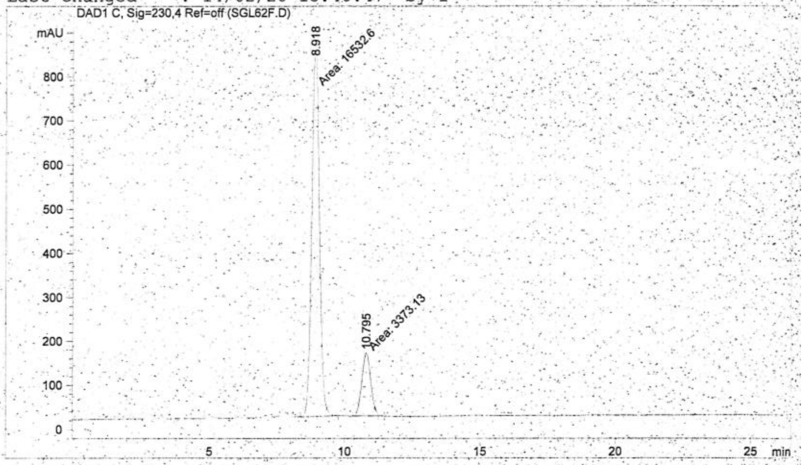


Area Percent Report

```
Sorted By          :      Signal
Multiplier         :      1.0000
Dilution           :      1.0000
```

Signal 1: DAD1 C, Sig=230,4 Ref=off

| Peak # | RetTime [min] | Type | Width [min] | Area [mAU*s] | Height [mAU] | Area % |
|---|---|---|---|---|---|---|
| 1 | 8.918 | MM | 0.3267 | 1.65326e4 | 843.48077 | 83.0545 |
| 2 | 10.795 | MM | 0.3929 | 3373.12915 | 143.08032 | 16.9455 |
| Totals : | | | | 1.99057e4 | 986.56110 | |

Results obtained with enhanced integrator!

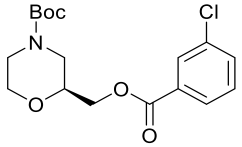


```
Injection Date  : 15/12/20 12.14.47
Sample Name     : SG499X_odh_10                          Vial :   1
Acq. Operator   : sabrina
Method          : C:\HPCHEM\1\METHODS\CECE.M
Last changed    : 15/12/20 12.02.13  by sabrina
                  (modified after loading)
```

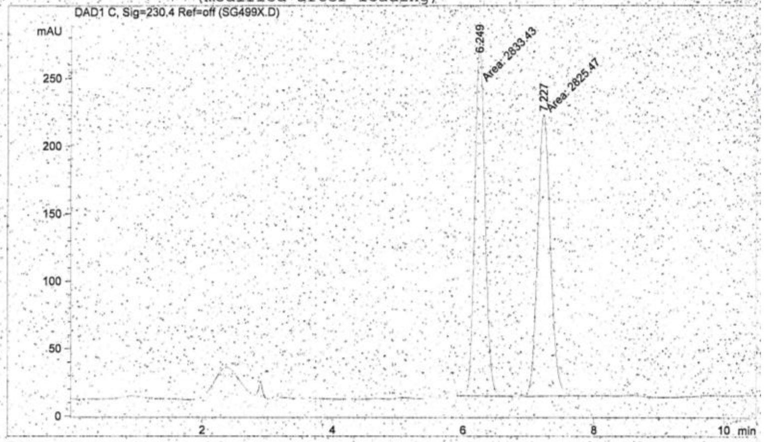


```
Area Percent Report

Sorted By             :      Signal
Multiplier            :      1.0000
Dilution              :      1.0000

Signal 1: DAD1 C, Sig=230,4 Ref=off

Peak RetTime Type  Width     Area      Height     Area
  #   [min]        [min]   [mAU*s]     [mAU]        %
----|-------|----|-------|----------|----------|--------|
   1   6.249 MM    0.1804 2833.43042  261.77948  50.0703
   2   7.227 MM    0.2264 2825.47095  208.02473  49.9297

Totals :                  5658.90137  469.80421

Results obtained with enhanced integrator!
```

```
Injection Date  : 15/12/20 12.02.40
Sample Name     : SGL61X_odh_10                          Vial :   1
Acq. Operator   : sabrina
Method          : C:\HPCHEM\1\METHODS\CECE.M
Last changed    : 15/12/20 12.02.13  by sabrina
                  (modified after loading)
```

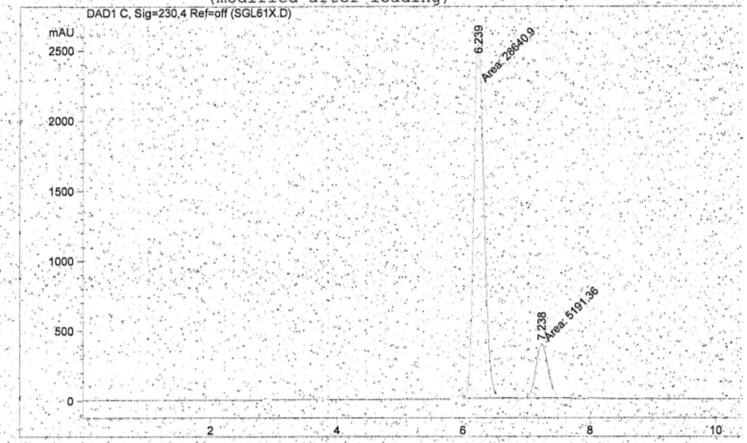


```
Area Percent Report

Sorted By             :      Signal
Multiplier            :      1.0000
Dilution              :      1.0000

Signal 1: DAD1 C, Sig=230,4 Ref=off

Peak RetTime Type  Width     Area      Height     Area
  #   [min]        [min]   [mAU*s]     [mAU]        %
----|-------|----|-------|----------|----------|--------|
   1   6.239 MM    0.1880 2.86409e4  2539.72974  84.6556
   2   7.238 MM    0.2232 5191.36035  387.72241  15.3444

Totals :                  3.38323e4  2927.45215

Results obtained with enhanced integrator!
```

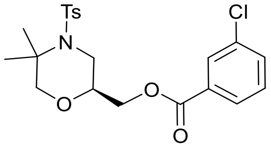


```
Injection Date  : 22/10/20 16.25.23
Sample Name     : SGL35_amy_3u30                         Vial :   1
Acq. Operator   : sabrina
Acq. Method     : C:\HPCHEM\1\METHODS\CECE.M
Last changed    : 22/10/20 16.33.17  by sabrina
                  (modified after loading)
Analysis Method : C:\HPCHEM\1\METHODS\CECE.M
Last changed    : 22/10/20 17.04.24  by sabrina
                  (modified after loading)
```

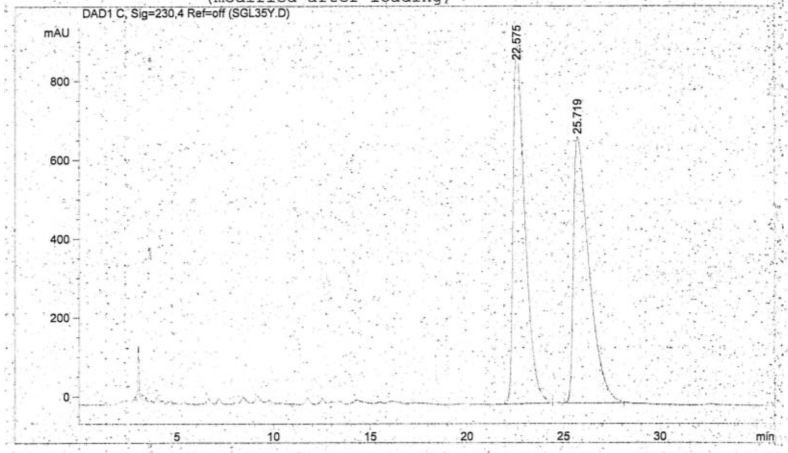


Area Percent Report

```
Sorted By          :     Signal
Multiplier         :     1.0000
Dilution           :     1.0000

Signal 1: DAD1 C, Sig=230,4 Ref=off
```

| Peak # | RetTime [min] | Type | Width [min] | Area [mAU*s] | Height [mAU] | Area % |
|---|---|---|---|---|---|---|
| 1 | 22.575 | BB | 0.6097 | 3.84972e4 | 913.38953 | 50.4172 |
| 2 | 25.719 | PB | 0.7885 | 3.78601e4 | 672.83215 | 49.5828 |

Totals : 7.63573e4 1586.22168

Results obtained with enhanced integrator!

```
Injection Date  : 18/12/20 15.22.27
Sample Name     : SGL41G_AMY3_30                         Vial :   1
Acq. Operator   : sabrina
Acq. Method     : C:\HPCHEM\1\METHODS\CECE.M
Last changed    : 14/02/20 15.40.47  by 1
Analysis Method : C:\HPCHEM\1\METHODS\CECE.M
Last changed    : 18/12/20 15.56.45  by sabrina
                  (modified after loading)
```

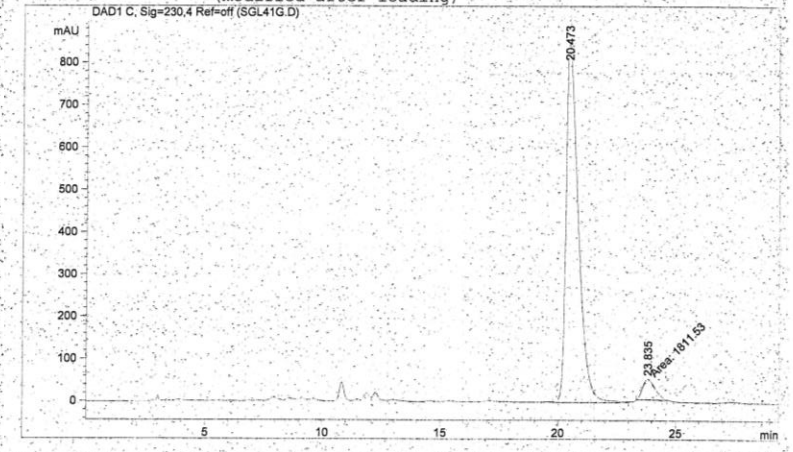


Area Percent Report

```
Sorted By          :     Signal
Multiplier         :     1.0000
Dilution           :     1.0000

Signal 1: DAD1 C, Sig=230,4 Ref=off
```

| Peak # | RetTime [min] | Type | Width [min] | Area [mAU*s] | Height [mAU] | Area % |
|---|---|---|---|---|---|---|
| 1 | 20.473 | VV | 0.5332 | 3.05758e4 | 846.93811 | 94.4067 |
| 2 | 23.835 | MM | 0.6216 | 1811.52527 | 48.57106 | 5.5933 |

Totals : 3.23874e4 895.50917

Results obtained with enhanced integrator!

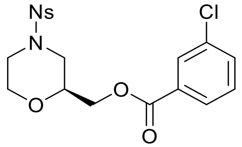


```
Injection Date  : 23/10/20 16.14.06
Sample Name     : SGL10_amy_5u30                  Vial :   1
Acq. Operator   : sabrina
Acq. Method     : C:\HPCHEM\1\METHODS\CECE.M
Last changed    : 23/10/20 16.17.19  by sabrina
                  (modified after loading)
Analysis Method : C:\HPCHEM\1\METHODS\CECE.M
Last changed    : 23/10/20 17.05.41  by sabrina
                  (modified after loading)
```

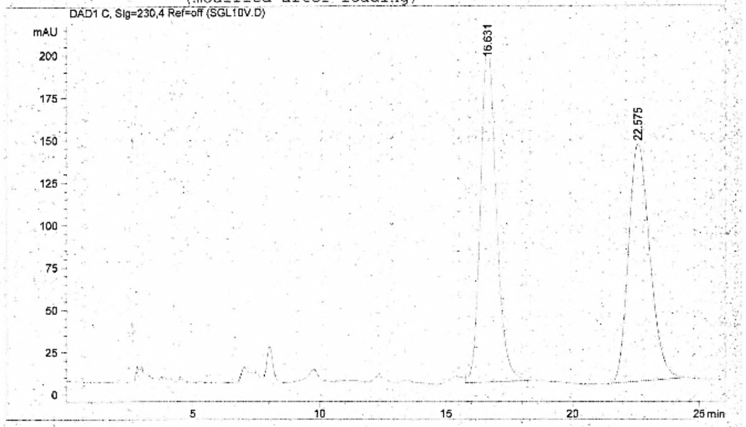


Area Percent Report

```
Sorted By          :    Signal
Multiplier         :    1.0000
Dilution           :    1.0000
```

Signal 1: DAD1 C, Sig=230,4 Ref=off

| Peak # | RetTime [min] | Type | Width [min] | Area [mAU*s] | Height [mAU] | Area % |
|---|---|---|---|---|---|---|
| 1 | 16.631 | VB | 0.6514 | 8609.37305 | 200.30495 | 51.5701 |
| 2 | 22.575 | BB | 0.8498 | 8085.13232 | 139.17250 | 48.4299 |

Totals : 1.66945e4 339.47745

Results obtained with enhanced integrator!

```
Injection Date  : 22/01/21 15.28.40
Sample Name     : SG60_2_AMY5_30                  Vial :   1
Acq. Operator   : sabrina
Method          : C:\HPCHEM\1\METHODS\CECE.M
Last changed    : 14/02/20 15.40.47  by 1
```

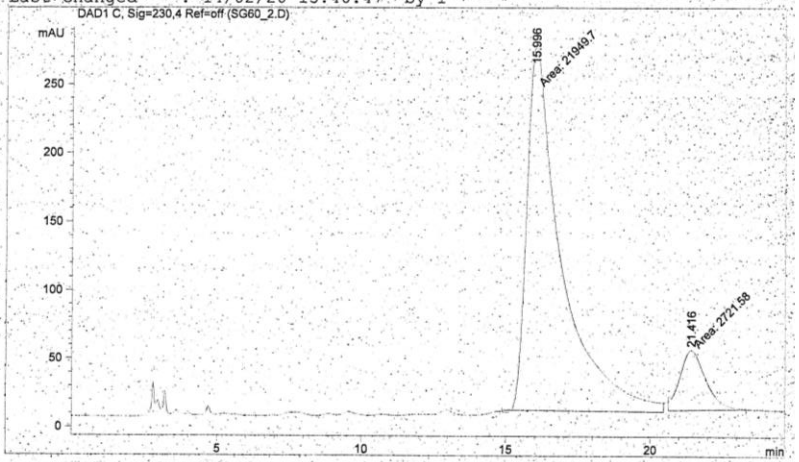


Area Percent Report

```
Sorted By          :    Signal
Multiplier         :    1.0000
Dilution           :    1.0000
```

Signal 1: DAD1 C, Sig=230,4 Ref=off

| Peak # | RetTime [min] | Type | Width [min] | Area [mAU*s] | Height [mAU] | Area % |
|---|---|---|---|---|---|---|
| 1 | 15.996 | MM | 1.3778 | 2.19497e4 | 265.51746 | 88.9686 |
| 2 | 21.416 | MM | 1.0403 | 2721.58325 | 43.60351 | 11.0314 |

Totals : 2.46713e4 309.12096

Results obtained with enhanced integrator!

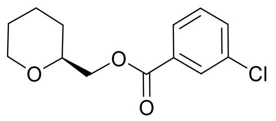


```
Injection Date  : 23/10/20 15.00.21
Sample Name     : SG704_amy_3u30                          Vial :   1
Acq. Operator   : sabrina
Method          : C:\HPCHEM\1\METHODS\CECE.M
Last changed    : 23/10/20 15.02.52  by sabrina
                  (modified after loading)
```

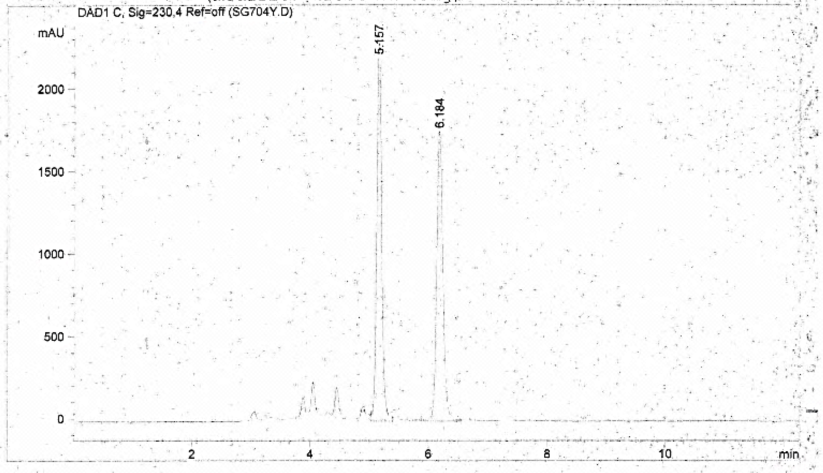


Area Percent Report

| | | |
|---|---|---|
| Sorted By | : | Signal |
| Multiplier | : | 1.0000 |
| Dilution | : | 1.0000 |

Signal 1: DAD1 C, Sig=230,4 Ref=off

| Peak # | RetTime [min] | Type | Width [min] | Area [mAU*s] | Height [mAU] | Area % |
|---|---|---|---|---|---|---|
| 1 | 5.157 | VV | 0.0901 | 1.33414e4 | 2286.28320 | 50.4321 |
| 2 | 6.184 | VB | 0.1131 | 1.31128e4 | 1755.53186 | 49.5679 |

Totals : 2.64543e4 4041.81506

Results obtained with enhanced integrator!

```
Injection Date  : 22/01/21 16.10.17
Sample Name     : SG68_AMY3_30                            Vial :   1
Acq. Operator   : sabrina
Method          : C:\HPCHEM\1\METHODS\CECE.M
Last changed    : 14/02/20 15.40.47  by 1
```

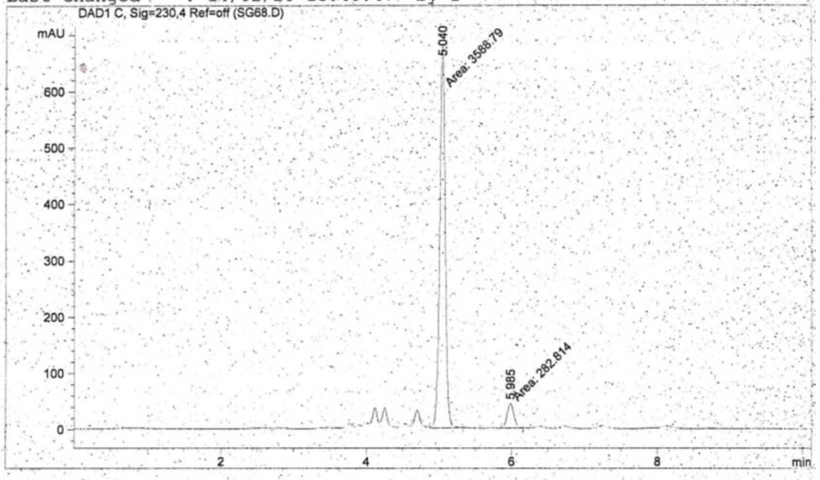


Area Percent Report

| | | |
|---|---|---|
| Sorted By | : | Signal |
| Multiplier | : | 1.0000 |
| Dilution | : | 1.0000 |

Signal 1: DAD1 C, Sig=230,4 Ref=off

| Peak # | RetTime [min] | Type | Width [min] | Area [mAU*s] | Height [mAU] | Area % |
|---|---|---|---|---|---|---|
| 1 | 5.040 | MM | 0.0875 | 3588.78735 | 683.91736 | 92.6952 |
| 2 | 5.985 | MM | 0.1077 | 282.81400 | 43.77470 | 7.3048 |

Totals : 3871.60135 727.69206

Results obtained with enhanced integrator!

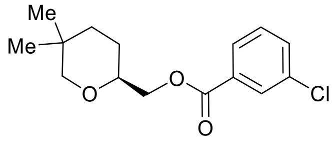


```
==================================================================
Injection Date  : 22/12/20 15.17.03
Sample Name     : SG64RAC_AMY3_10                    Vial :   1
Acq. Operator   : sabrina
Method          : C:\HPCHEM\1\METHODS\CECE.M
Last changed    : 22/12/20 15.08.19  by sabrina
                  (modified after loading)
```

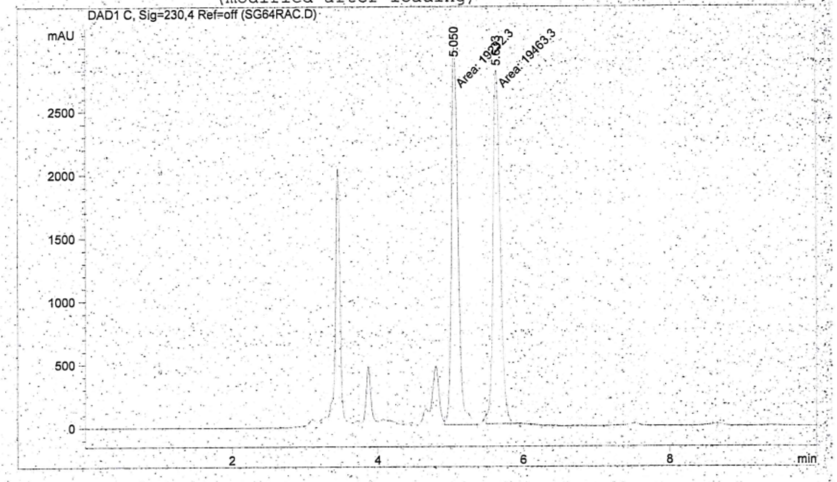


```
==================================================================
                    Area Percent Report
==================================================================

Sorted By            :      Signal
Multiplier           :      1.0000
Dilution             :      1.0000

Signal 1: DAD1 C, Sig=230,4 Ref=off
```

| Peak # | RetTime [min] | Type | Width [min] | Area [mAU*s] | Height [mAU] | Area % |
|---|---|---|---|---|---|---|
| 1 | 5.050 | MM | 0.1068 | 1.92323e4 | 3002.00293 | 49.7015 |
| 2 | 5.633 | MM | 0.1160 | 1.94633e4 | 2795.52417 | 50.2985 |

Totals : 3.86956e4 5797.52710

Results obtained with enhanced integrator!

```
==================================================================
Injection Date  : 22/12/20 15.27.50
Sample Name     : SG64F7_AMY3_10                     Vial :   1
Acq. Operator   : sabrina
Acq. Method     : C:\HPCHEM\1\METHODS\CECE.M
Last changed    : 22/12/20 15.08.19  by sabrina
                  (modified after loading)
Analysis Method : C:\HPCHEM\1\METHODS\CECE.M
Last changed    : 22/12/20 15.47.06  by sabrina
                  (modified after loading)
```

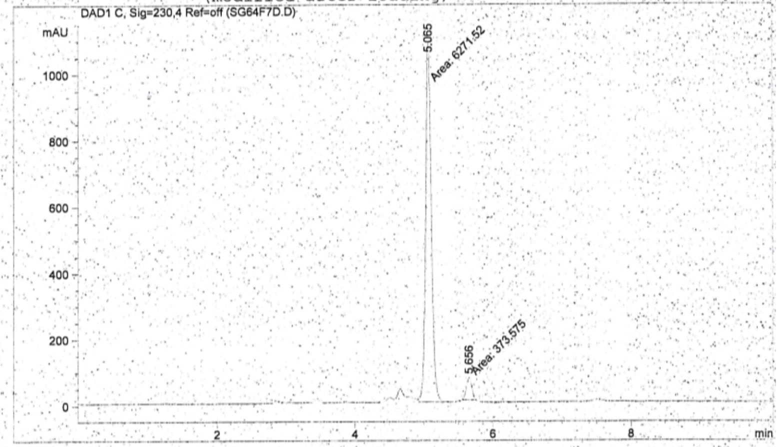


```
==================================================================
                    Area Percent Report
==================================================================

Sorted By            :      Signal
Multiplier           :      1.0000
Dilution             :      1.0000

Signal 1: DAD1 C, Sig=230,4 Ref=off
```

| Peak # | RetTime [min] | Type | Width [min] | Area [mAU*s] | Height [mAU] | Area % |
|---|---|---|---|---|---|---|
| 1 | 5.065 | MM | 0.0956 | 6271.52002 | 1093.80457 | 94.3782 |
| 2 | 5.656 | MM | 0.0927 | 373.57532 | 67.15026 | 5.6218 |

Totals : 6645.09534 1160.95483

Results obtained with enhanced integrator!

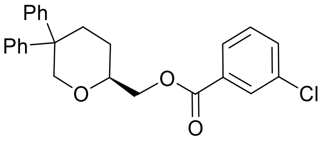


```
Injection Date  : 27/01/21 16.48.22
Sample Name     : SG80rx_odh_5                          Vial :   1
Acq. Operator   : sabrina
Method          : C:\HPCHEM\1\METHODS\CECE.M
Last changed    : 14/02/20 15.40.47  by 1
```

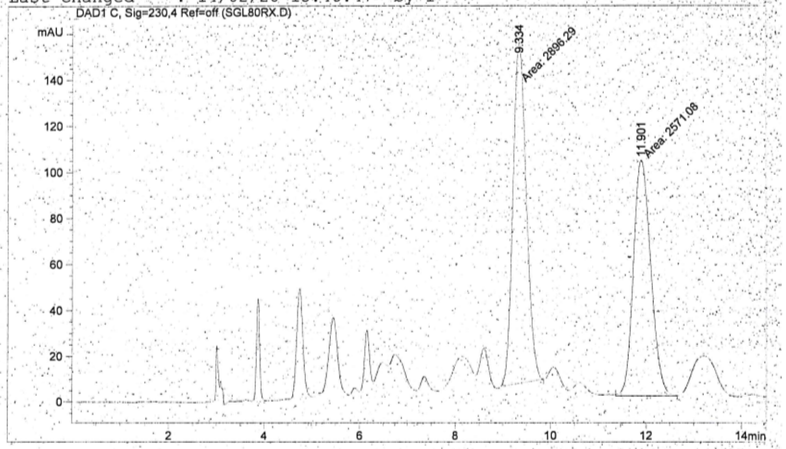


Area Percent Report

```
Sorted By          :      Signal
Multiplier         :      1.0000
Dilution           :      1.0000
```

Signal 1: DAD1 C, Sig=230,4 Ref=off

| Peak # | RetTime [min] | Type | Width [min] | Area [mAU*s] | Height [mAU] | Area % |
|---|---|---|---|---|---|---|
| 1 | 9.334 | MM | 0.3261 | 2896.29419 | 148.04005 | 52.9742 |
| 2 | 11.901 | MM | 0.4183 | 2571.07666 | 102.43639 | 47.0258 |

Totals : 5467.37085 250.47644

Results obtained with enhanced integrator!

```
Injection Date  : 27/01/21 17.05.13
Sample Name     : SG80ex_odh_5                          Vial :   1
Acq. Operator   : sabrina
Method          : C:\HPCHEM\1\METHODS\CECE.M
Last changed    : 14/02/20 15.40.47  by 1
```

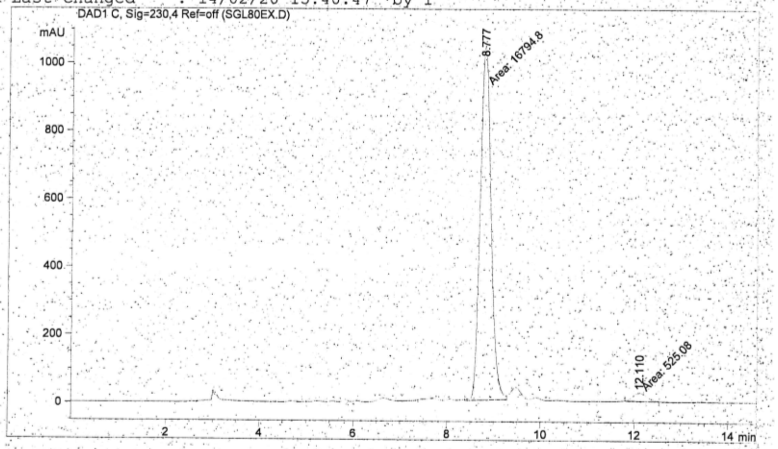


Area Percent Report

```
Sorted By          :      Signal
Multiplier         :      1.0000
Dilution           :      1.0000
```

Signal 1: DAD1 C, Sig=230,4 Ref=off

| Peak # | RetTime [min] | Type | Width [min] | Area [mAU*s] | Height [mAU] | Area % |
|---|---|---|---|---|---|---|
| 1 | 8.777 | MM | 0.2687 | 1.67948e4 | 1041.71460 | 96.9683 |
| 2 | 12.110 | MM | 0.3589 | 525.08014 | 24.38369 | 3.0317 |

Totals : 1.73199e4 1066.09829

Results obtained with enhanced integrator!

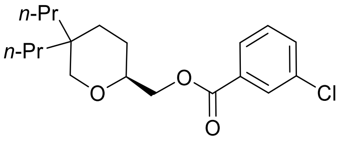


Injection Date : 19/03/21 10.15.48
Sample Name : SGl98rac_amy3_5 Vial : 1
Acq. Operator : sabrina
Method : C:\HPCHEM\1\METHODS\CECE.M
Last changed : 14/02/20 15.40.47 by 1

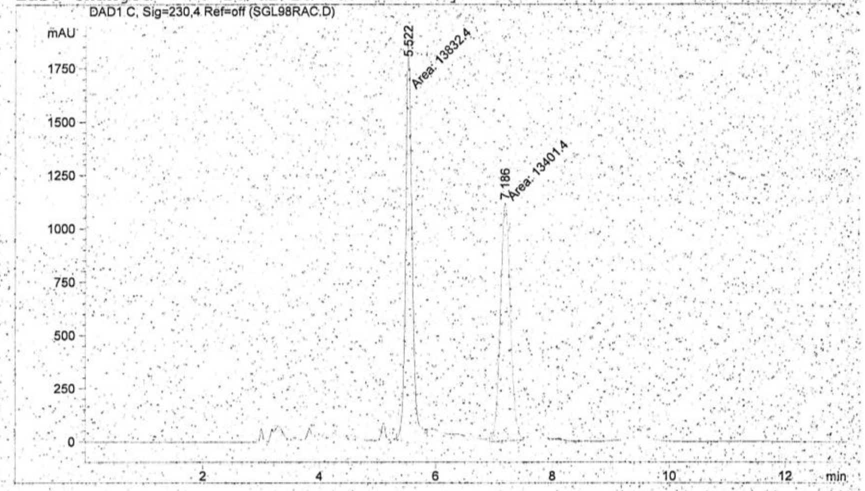


Area Percent Report

Sorted By : Signal
Multiplier : 1.0000
Dilution : 1.0000

Signal 1: DAD1 C, Sig=230,4 Ref=off

| Peak # | RetTime [min] | Type | Width [min] | Area [mAU*s] | Height [mAU] | Area % |
|---|---|---|---|---|---|---|
| 1 | 5.522 | MM | 0.1255 | 1.38324e4 | 1836.96594 | 50.7912 |
| 2 | 7.186 | MM | 0.2006 | 1.34014e4 | 1113.65112 | 49.2088 |

Totals : 2.72338e4 2950.61707

Results obtained with enhanced integrator!

Injection Date : 19/03/21 10.41.11
Sample Name : SGl98ee_amy3_5 Vial : 1
Acq. Operator : sabrina
Acq. Method : C:\HPCHEM\1\METHODS\CECE.M
Last changed : 14/02/20 15.40.47 by 1
Analysis Method : C:\HPCHEM\1\METHODS\CECE.M
Last changed : 19/03/21 10.58.55 by sabrina
(modified after loading)

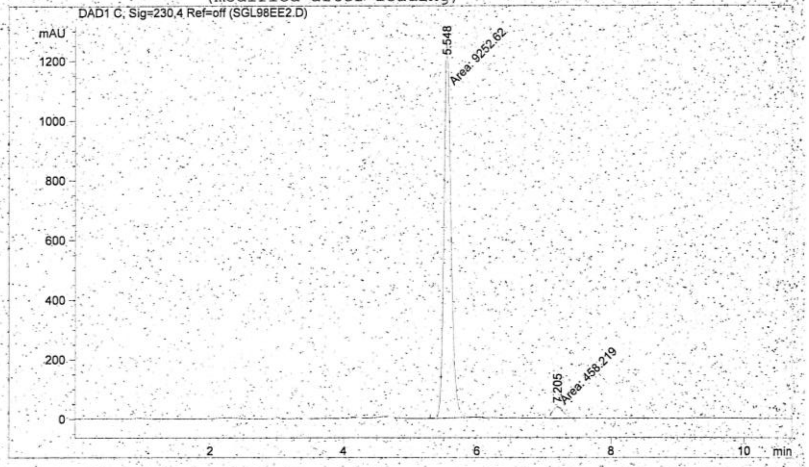


Area Percent Report

Sorted By : Signal
Multiplier : 1.0000
Dilution : 1.0000

Signal 1: DAD1 C, Sig=230,4 Ref=off

| Peak # | RetTime [min] | Type | Width [min] | Area [mAU*s] | Height [mAU] | Area % |
|---|---|---|---|---|---|---|
| 1 | 5.548 | MM | 0.1231 | 9252.61719 | 1252.94312 | 95.2814 |
| 2 | 7.205 | MM | 0.1979 | 458.21924 | 38.59211 | 4.7186 |

Totals : 9710.83643 1291.53523

Results obtained with enhanced integrator!

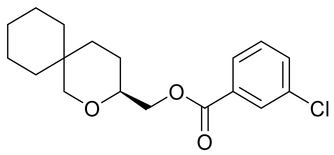


```
Injection Date  : 30/06/21 14.17.53
Sample Name     : SGL111r OD 10i                    Vial :   1
Acq. Operator   : sabrina
Method          : C:\HPCHEM\1\METHODS\CECE.M
Last changed    : 16/06/21 15.16.00  by vale p
```

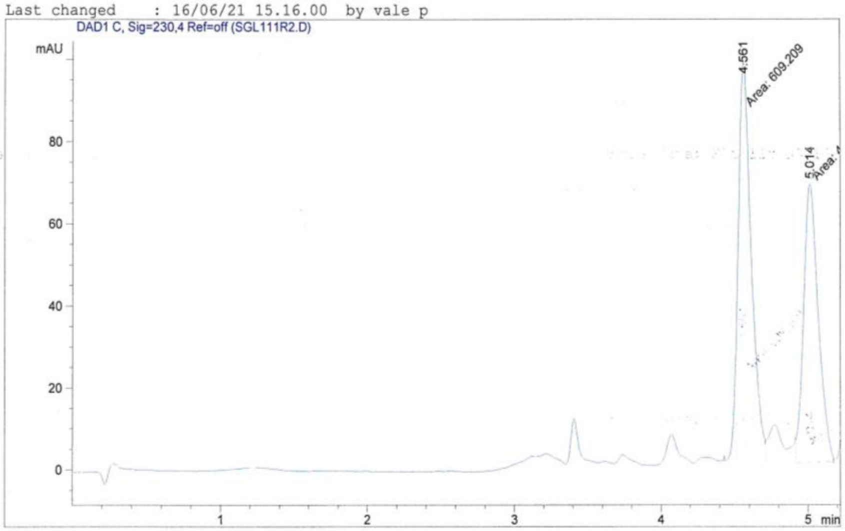


Area Percent Report

| | | |
|---|---|---|
| Sorted By | : | Signal |
| Multiplier | : | 1.0000 |
| Dilution | : | 1.0000 |

Signal 1: DAD1 C, Sig=230,4 Ref=off

| Peak # | RetTime [min] | Type | Width [min] | Area [mAU*s] | Height [mAU] | Area % |
|---|---|---|---|---|---|---|
| 1 | 4.561 | MM | 0.1041 | 609.20886 | 97.50368 | 56.6167 |
| 2 | 5.014 | MM | 0.1141 | 466.81396 | 68.17485 | 43.3833 |

Totals : 1076.02283 165.67853

Results obtained with enhanced integrator!

*** End of Report ***

```
Injection Date  : 30/06/21 14.32.06
Sample Name     : SGL111e3 OD 10i                   Vial :   1
Acq. Operator   : sabrina
Method          : C:\HPCHEM\1\METHODS\CECE.M
Last changed    : 16/06/21 15.16.00  by vale p
```

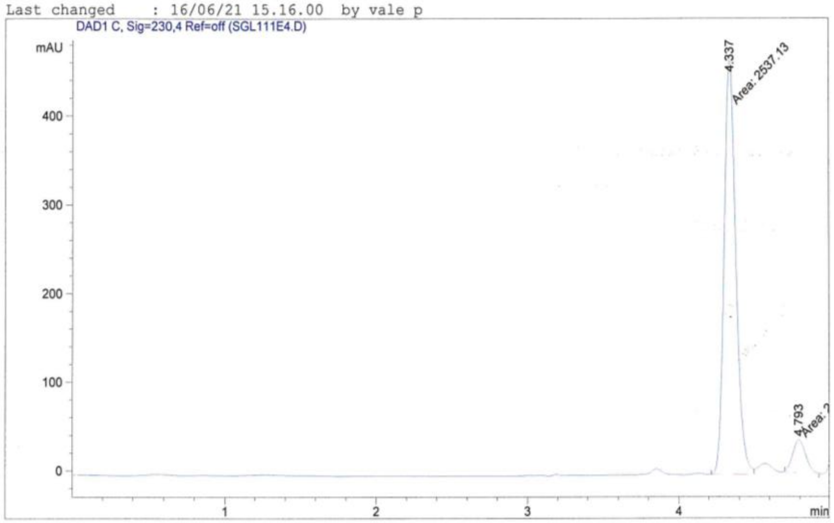


Area Percent Report

| | | |
|---|---|---|
| Sorted By | : | Signal |
| Multiplier | : | 1.0000 |
| Dilution | : | 1.0000 |

Signal 1: DAD1 C, Sig=230,4 Ref=off

| Peak # | RetTime [min] | Type | Width [min] | Area [mAU*s] | Height [mAU] | Area % |
|---|---|---|---|---|---|---|
| 1 | 4.337 | MM | 0.0899 | 2537.12891 | 470.33481 | 92.2039 |
| 2 | 4.793 | MM | 0.0966 | 214.52083 | 37.01746 | 7.7961 |

Totals : 2751.64973 507.35226

Results obtained with enhanced integrator!

*** End of Report ***

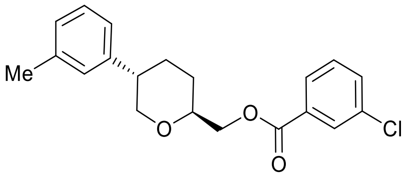


```
Injection Date  : 17/02/21 14.53.23
Sample Name     : SGL85F15_odh_5                    Vial :   1
Acq. Operator   : sabrina
Method          : C:\HPCHEM\1\METHODS\CECE.M
Last changed    : 14/02/20 15.40.47  by 1
```

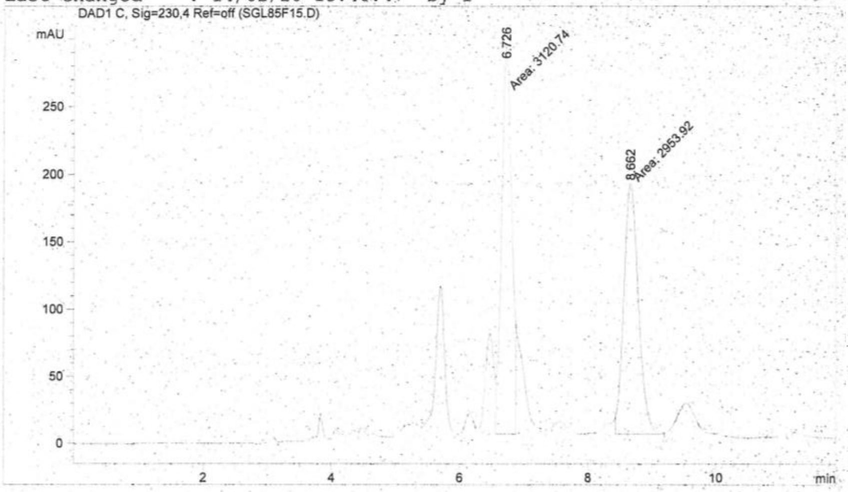


Area Percent Report

```
Sorted By          :     Signal
Multiplier         :     1.0000
Dilution           :     1.0000
```

Signal 1: DAD1 C, Sig=230,4 Ref=off

| Peak # | RetTime [min] | Type | Width [min] | Area [mAU*s] | Height [mAU] | Area % |
|---|---|---|---|---|---|---|
| 1 | 6.726 | MM | 0.1809 | 3120.74390 | 287.56183 | 51.3731 |
| 2 | 8.662 | MM | 0.2652 | 2953.92236 | 185.66353 | 48.6269 |

Totals : 6074.66626 473.22536

Results obtained with enhanced integrator!

```
Injection Date  : 17/02/21 15.18.02
Sample Name     : SGL85F9eb_odh_5                   Vial :   1
Acq. Operator   : sabrina
Method          : C:\HPCHEM\1\METHODS\CECE.M
Last changed    : 14/02/20 15.40.47  by 1
```

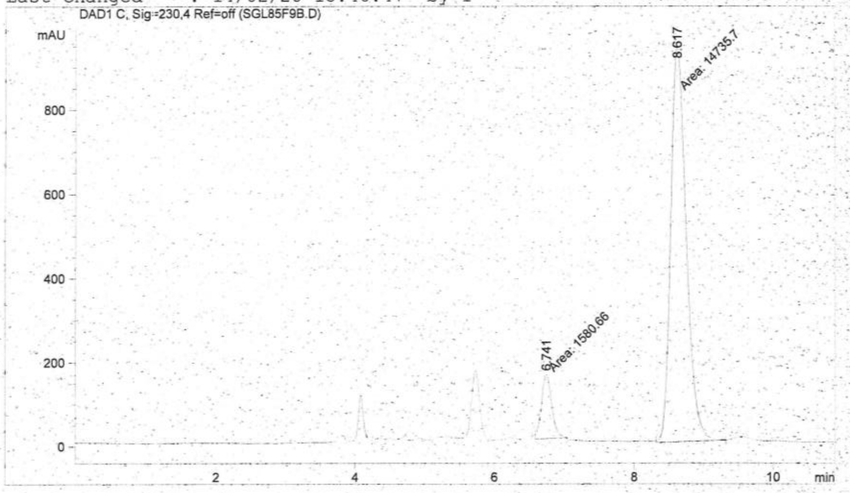


Area Percent Report

```
Sorted By          :     Signal
Multiplier         :     1.0000
Dilution           :     1.0000
```

Signal 1: DAD1 C, Sig=230,4 Ref=off

| Peak # | RetTime [min] | Type | Width [min] | Area [mAU*s] | Height [mAU] | Area % |
|---|---|---|---|---|---|---|
| 1 | 6.741 | MM | 0.1722 | 1580.66406 | 153.01257 | 9.6876 |
| 2 | 8.617 | MM | 0.2619 | 1.47357e4 | 937.86469 | 90.3124 |

Totals : 1.63163e4 1090.87726

Results obtained with enhanced integrator!

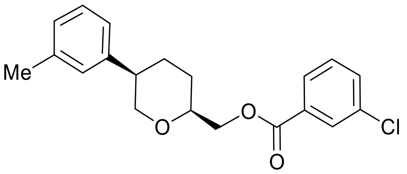


```
==================================================================
Injection Date   : 17/02/21 15.55.56
Sample Name      : SGL85F16_odh_5                    Vial :   1
Acq. Operator    : sabrina
Acq. Method      : C:\HPCHEM\1\METHODS\CECE.M
Last changed     : 14/02/20 15.40.47  by 1
Analysis Method  : C:\HPCHEM\1\METHODS\CECE.M
Last changed     : 17/02/21 16.12.04  by sabrina
                   (modified after loading)
```

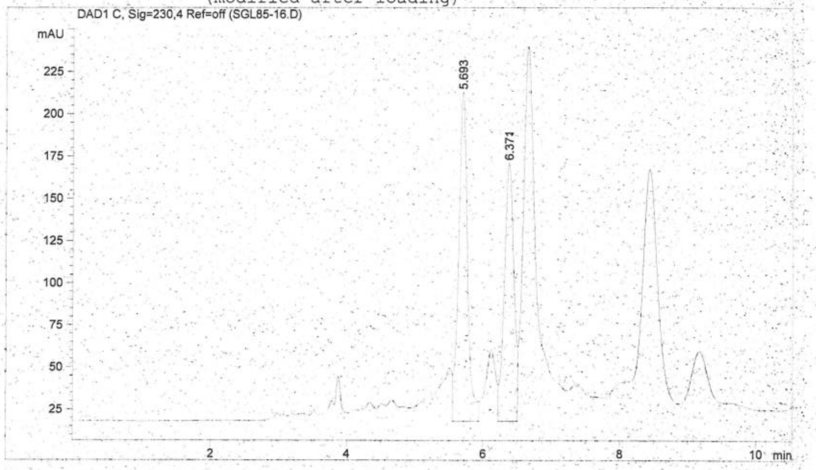


```
==================================================================
                      Area Percent Report
==================================================================

Sorted By            :      Signal
Multiplier           :      1.0000
Dilution             :      1.0000


Signal 1: DAD1 C, Sig=230,4 Ref=off

Peak RetTime Type  Width     Area      Height     Area
  #   [min]        [min]   [mAU*s]     [mAU]        %
----|-------|----|-------|----------|----------|--------|
   1   5.693 VV    0.1381 1767.28186  194.39514  54.1701
   2   6.371 VV    0.1503 1495.18359  152.56760  45.8299

Totals :                  3262.46545  346.96274

 Results obtained with enhanced integrator!
==================================================================
```

```
==================================================================
Injection Date   : 17/02/21 15.42.36
Sample Name      : SGL85F10e_odh_5                    Vial :   1
Acq. Operator    : sabrina
Method           : C:\HPCHEM\1\METHODS\CECE.M
Last changed     : 14/02/20 15.40.47  by 1
```

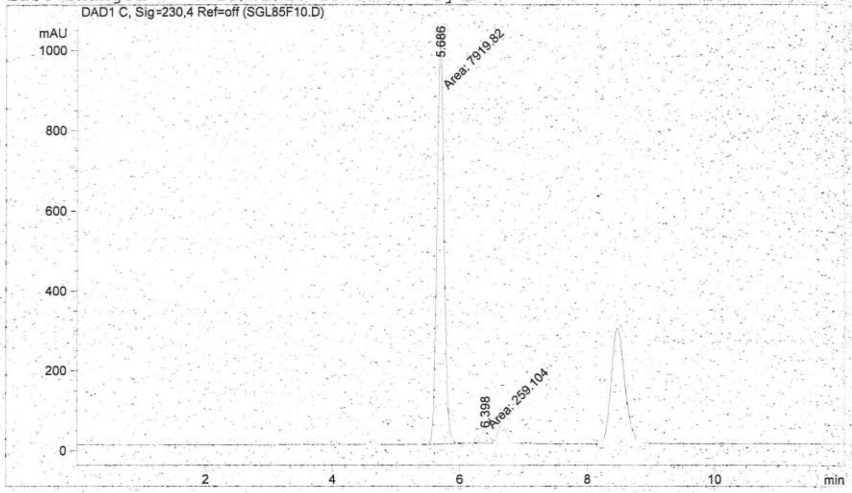


```
==================================================================
                      Area Percent Report
==================================================================

Sorted By            :      Signal
Multiplier           :      1.0000
Dilution             :      1.0000


Signal 1: DAD1 C, Sig=230,4 Ref=off

Peak RetTime Type  Width     Area      Height     Area
  #   [min]        [min]   [mAU*s]     [mAU]        %
----|-------|----|-------|----------|----------|--------|
   1   5.686 MM    0.1324 7919.81885  997.20337  96.8321
   2   6.398 MM    0.1486  259.10385   29.05127   3.1679

Totals :                  8178.92270 1026.25464

 Results obtained with enhanced integrator!
==================================================================
```

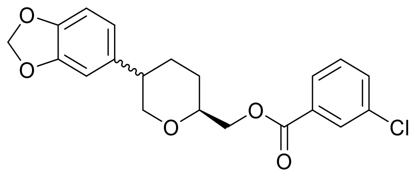

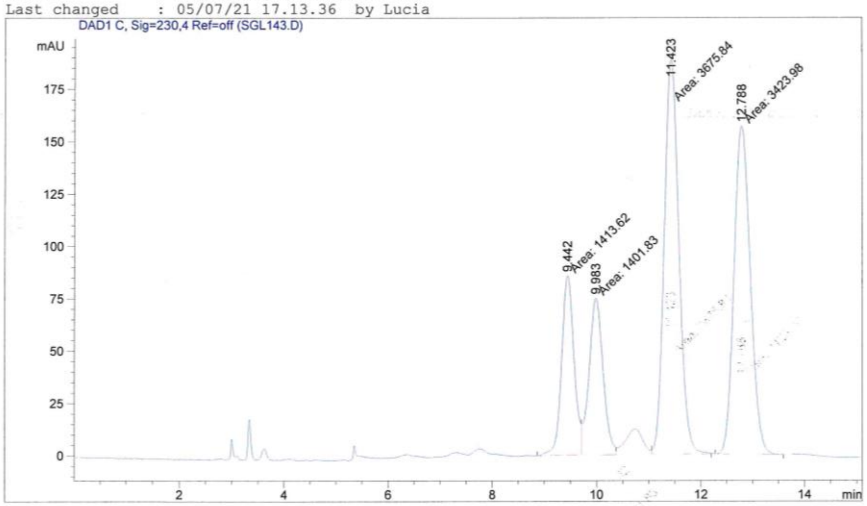


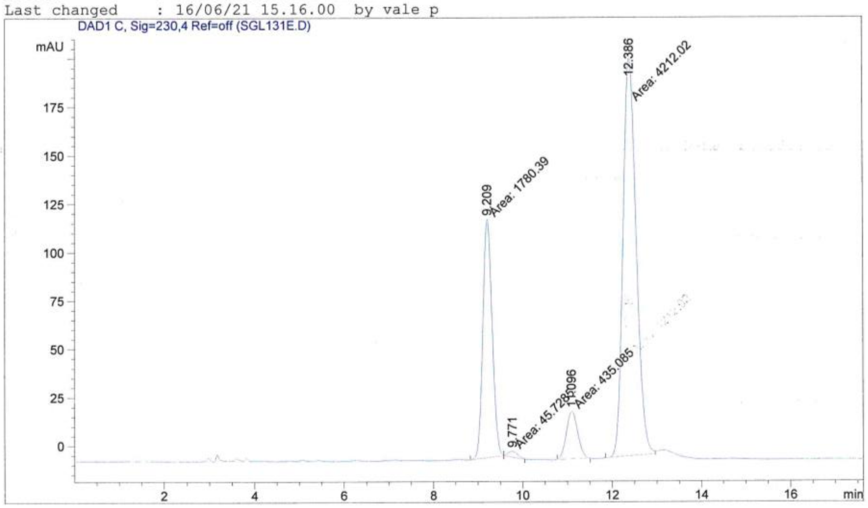


Injection Date : 09/07/21 13.38.23
Sample Name : SGL143 Vial : 1
Acq. Operator : Saria
Method : C:\HPCHEM\1\METHODS\CECE.M
Last changed : 05/07/21 17.13.36 by Lucia

Area Percent Report

Sorted By : Signal
Multiplier : 1.0000
Dilution : 1.0000

Signal 1: DAD1 C, Sig=230,4 Ref=off

| Peak # | RetTime [min] | Type | Width [min] | Area [mAU*s] | Height [mAU] | Area % |
|---|---|---|---|---|---|---|
| 1 | 9.442 | MM | 0.2747 | 1413.61560 | 85.77908 | 14.2570 |
| 2 | 9.983 | MM | 0.3120 | 1401.82849 | 74.88804 | 14.1381 |
| 3 | 11.423 | MM | 0.3244 | 3675.84033 | 188.87381 | 37.0726 |
| 4 | 12.788 | MM | 0.3637 | 3423.97607 | 156.90059 | 34.5324 |

Totals : 9915.26050 506.44151

Results obtained with enhanced integrator!

*** End of Report ***

Injection Date : 30/06/21 14.58.37
Sample Name : SGL131e OD 5i Vial : 1
Acq. Operator : sabrina
Method : C:\HPCHEM\1\METHODS\CECE.M
Last changed : 16/06/21 15.16.00 by vale p

Area Percent Report

Sorted By : Signal
Multiplier : 1.0000
Dilution : 1.0000

Signal 1: DAD1 C, Sig=230,4 Ref=off

| Peak # | RetTime [min] | Type | Width [min] | Area [mAU*s] | Height [mAU] | Area % |
|---|---|---|---|---|---|---|
| 1 | 9.209 | MM | 0.2408 | 1780.38977 | 123.24638 | 27.5039 |
| 2 | 9.771 | MM | 0.2455 | 45.72850 | 3.10420 | 0.7064 |
| 3 | 11.096 | MM | 0.2933 | 435.08496 | 24.72416 | 6.7213 |
| 4 | 12.386 | MM | 0.3419 | 4212.01611 | 205.30264 | 65.0683 |

Totals : 6473.21935 356.37738

Results obtained with enhanced integrator!

*** End of Report ***

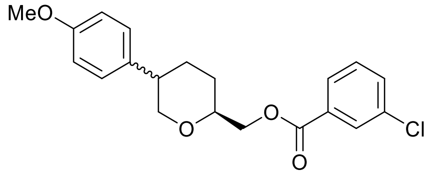


```
Injection Date  : 09/07/21 14.11.22
Sample Name     : SGL144                                Vial :   1
Acq. Operator   : Saria
Method          : C:\HPCHEM\1\METHODS\CECE.M
Last changed    : 05/07/21 17.13.36  by Lucia
```

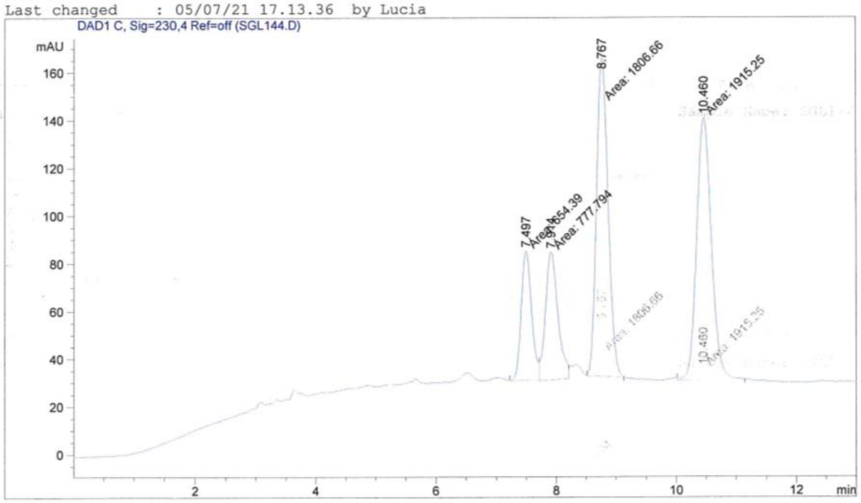


Area Percent Report

```
Sorted By          :      Signal
Multiplier         :      1.0000
Dilution           :      1.0000
```

Signal 1: DAD1 C, Sig=230,4 Ref=off

| Peak # | RetTime [min] | Type | Width [min] | Area [mAU*s] | Height [mAU] | Area % |
|---|---|---|---|---|---|---|
| 1 | 7.497 | MM | 0.2009 | 654.39038 | 54.27993 | 12.6965 |
| 2 | 7.914 | MM | 0.2412 | 777.79437 | 53.74920 | 15.0908 |
| 3 | 8.767 | MM | 0.2255 | 1806.66077 | 133.55495 | 35.0529 |
| 4 | 10.460 | MM | 0.2905 | 1915.24731 | 109.89252 | 37.1597 |

Totals : 5154.09283 351.47660

Results obtained with enhanced integrator!

*** End of Report ***

```
Injection Date  : 09/07/21 13.55.23
Sample Name     : SGL142                                Vial :   1
Acq. Operator   : Saria
Method          : C:\HPCHEM\1\METHODS\CECE.M
Last changed    : 05/07/21 17.13.36  by Lucia
```

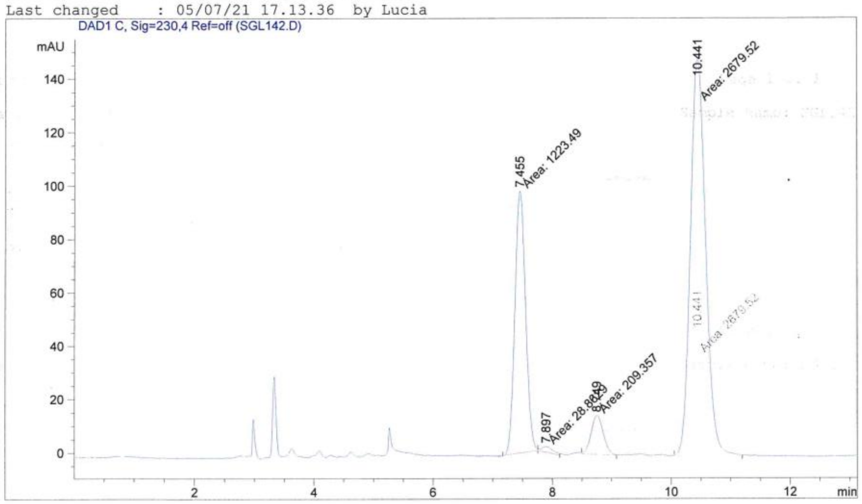


Area Percent Report

```
Sorted By          :      Signal
Multiplier         :      1.0000
Dilution           :      1.0000
```

Signal 1: DAD1 C, Sig=230,4 Ref=off

| Peak # | RetTime [min] | Type | Width [min] | Area [mAU*s] | Height [mAU] | Area % |
|---|---|---|---|---|---|---|
| 1 | 7.455 | MM | 0.2075 | 1223.48560 | 98.26891 | 29.5440 |
| 2 | 7.897 | MM | 0.2229 | 28.86286 | 2.15788 | 0.6970 |
| 3 | 8.749 | MM | 0.2392 | 209.35725 | 14.59026 | 5.0554 |
| 4 | 10.441 | MM | 0.2996 | 2679.52026 | 149.06940 | 64.7036 |

Totals : 4141.22598 264.08644

Results obtained with enhanced integrator!

*** End of Report ***

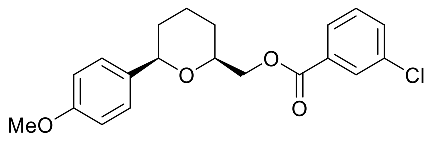


Injection Date : 30/06/21 12.10.37
Sample Name : SGL123r OD 10i    Vial : 1
Acq. Operator :
Acq. Method : C:\HPCHEM\1\METHODS\CECE.M
Last changed : 16/06/21 15.16.00 by vale p
Analysis Method : C:\HPCHEM\1\METHODS\CECE.M
Last changed : 30/06/21 12.30.35 by sabrina
(modified after loading)

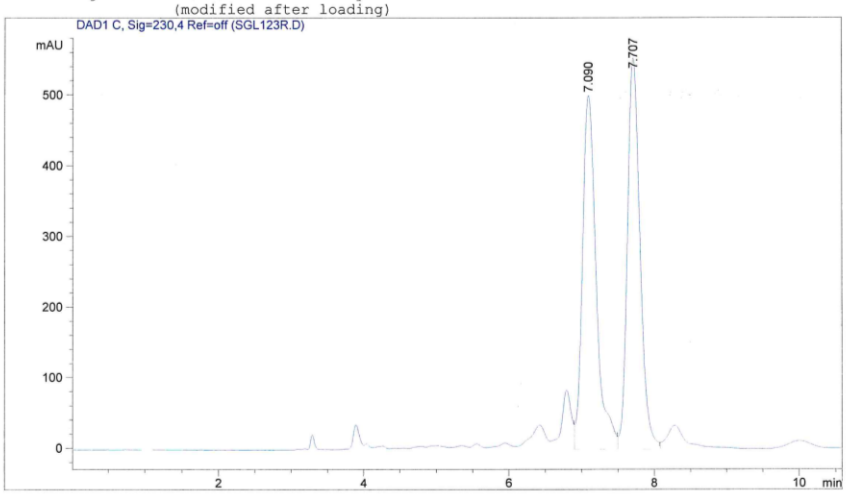


Area Percent Report

Sorted By : Signal
Multiplier : 1.0000
Dilution : 1.0000

Signal 1: DAD1 C, Sig=230,4 Ref=off

| Peak # | RetTime [min] | Type | Width [min] | Area [mAU*s] | Height [mAU] | Area % |
|---|---|---|---|---|---|---|
| 1 | 7.090 | VV | 0.2016 | 6595.24561 | 500.77893 | 49.5515 |
| 2 | 7.707 | VV | 0.1894 | 6714.64307 | 554.05182 | 50.4485 |

Totals : 1.33099e4 1054.83075

Results obtained with enhanced integrator!

*** End of Report ***

Injection Date : 30/06/21 13.40.20
Sample Name : SGL110e OD 10i    Vial : 1
Acq. Operator : sabrina
Acq. Method : C:\HPCHEM\1\METHODS\CECE.M
Last changed : 30/06/21 12.30.35 by sabrina
(modified after loading)
Analysis Method : C:\HPCHEM\1\METHODS\CECE.M
Last changed : 16/06/21 15.16.00 by vale p

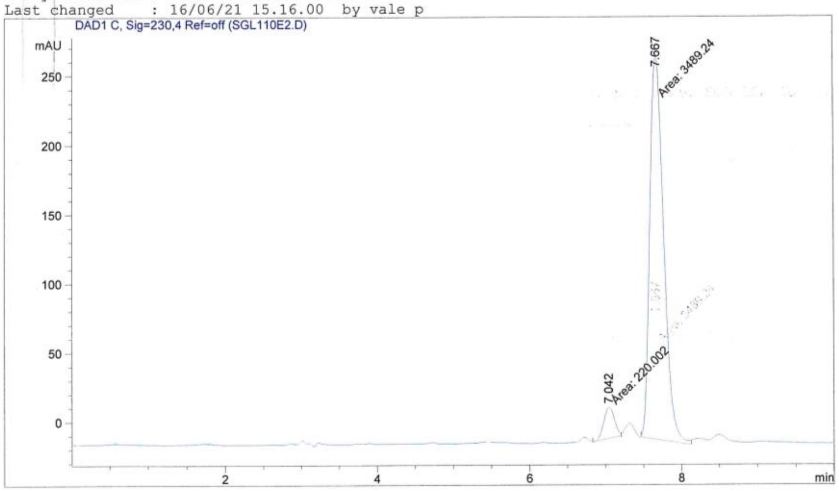


Area Percent Report

Sorted By : Signal
Multiplier : 1.0000
Dilution : 1.0000

Signal 1: DAD1 C, Sig=230,4 Ref=off

| Peak # | RetTime [min] | Type | Width [min] | Area [mAU*s] | Height [mAU] | Area % |
|---|---|---|---|---|---|---|
| 1 | 7.042 | MM | 0.1617 | 220.00212 | 22.67938 | 5.9312 |
| 2 | 7.667 | MM | 0.2100 | 3489.23999 | 276.92654 | 94.0688 |

Totals : 3709.24211 299.60592

Results obtained with enhanced integrator!

*** End of Report ***

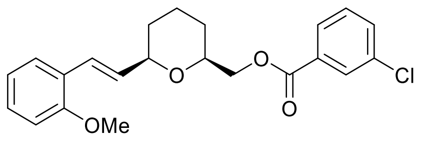


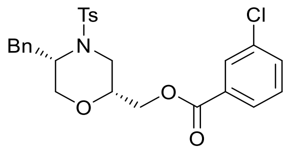


Injection Date : 30/06/21 15.43.07
Sample Name : SGL141e OD 5i    Vial : 1
Acq. Operator : sabrina
Acq. Method : C:\HPCHEM\1\METHODS\CECE.M
Last changed : 30/06/21 15.48.23 by sabrina
(modified after loading)
Analysis Method : C:\HPCHEM\1\METHODS\CECE.M
Last changed : 30/06/21 17.09.21 by sabrina
(modified after loading)

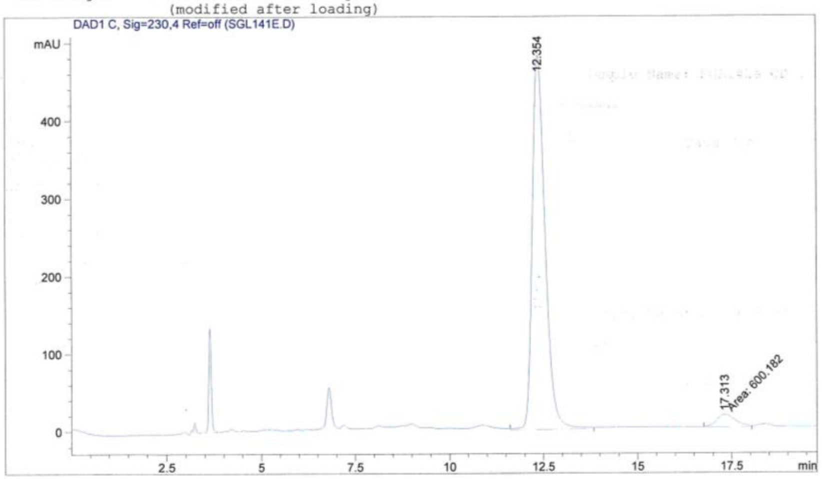


Area Percent Report

Sorted By : Signal
Multiplier : 1.0000
Dilution : 1.0000

Signal 1: DAD1 C, Sig=230,4 Ref=off

| Peak # | RetTime [min] | Type | Width [min] | Area [mAU*s] | Height [mAU] | Area % |
|---|---|---|---|---|---|---|
| 1 | 12.354 | VV | 0.3654 | 1.14549e4 | 481.75293 | 95.0213 |
| 2 | 17.313 | MM | 0.5947 | 600.18195 | 16.81946 | 4.9787 |

Totals : 1.20551e4 498.57239

Results obtained with enhanced integrator!

*** End of Report ***

Injection Date : 17/07/19 11.25.31
Sample Name : sg623af5 ce2 10i    Vial : 1
Acq. Operator : 1
Method : C:\HPCHEM\1\METHODS\CECE.M
Last changed : 09/07/19 15.32.49 by 1
(modified after loading)

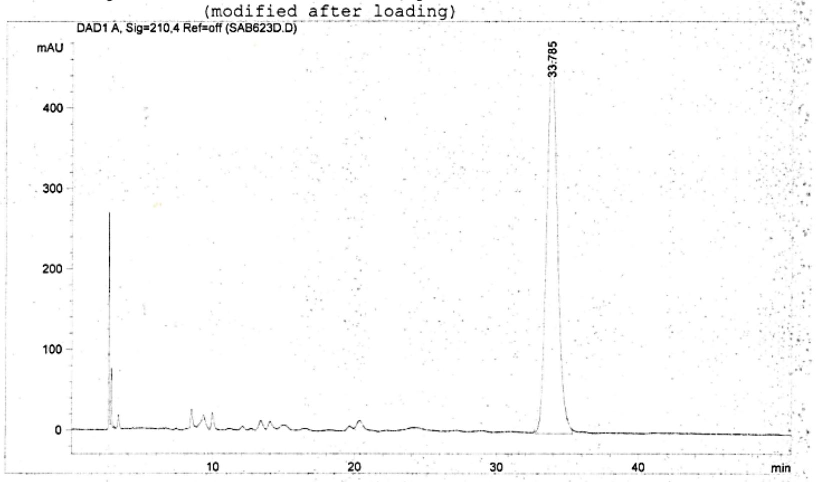


Area Percent Report

Sorted By : Signal
Multiplier : 1.0000
Dilution : 1.0000

Signal 1: DAD1 A, Sig=210,4 Ref=off

| Peak # | RetTime [min] | Type | Width [min] | Area [mAU*s] | Height [mAU] | Area % |
|---|---|---|---|---|---|---|
| 1 | 33.785 | VV | 0.6527 | 2.55215e4 | 463.27847 | 100.0000 |

Totals : 2.55215e4 463.27847

Results obtained with enhanced integrator!

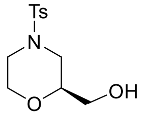


```
Injection Date  : 01/12/20 15.52.52
Sample Name     : SG705ra_amy3u_30                    Vial :   1
Acq. Operator   : sabrina
Method          : C:\HPCHEM\1\METHODS\CECE.M
Last changed    : 14/02/20 15.40.47  by 1
```

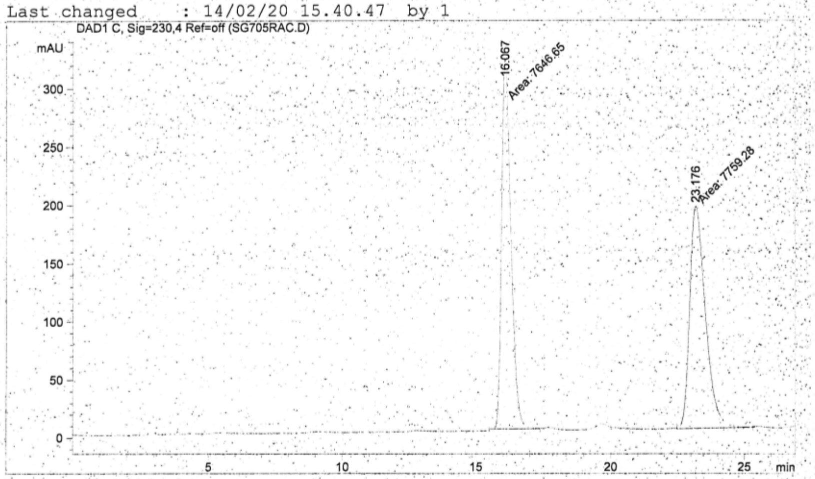


Area Percent Report

```
Sorted By        :     Signal
Multiplier       :     1.0000
Dilution         :     1.0000
```

Signal 1: DAD1 C, Sig=230,4 Ref=off

| Peak # | RetTime [min] | Type | Width [min] | Area [mAU*s] | Height [mAU] | Area % |
|---|---|---|---|---|---|---|
| 1 | 16.067 | MM | 0.4016 | 7646.65430 | 317.37805 | 49.6345 |
| 2 | 23.176 | MM | 0.6784 | 7759.28174 | 190.62187 | 50.3655 |

Totals : 1.54059e4 507.99992

Results obtained with enhanced integrator!

```
Injection Date  : 17/02/21 17.29.24
Sample Name     : SG705-3z_amy3_30                    Vial :   1
Acq. Operator   : sabrina
Method          : C:\HPCHEM\1\METHODS\CECE.M
Last changed    : 14/02/20 15.40.47  by 1
```

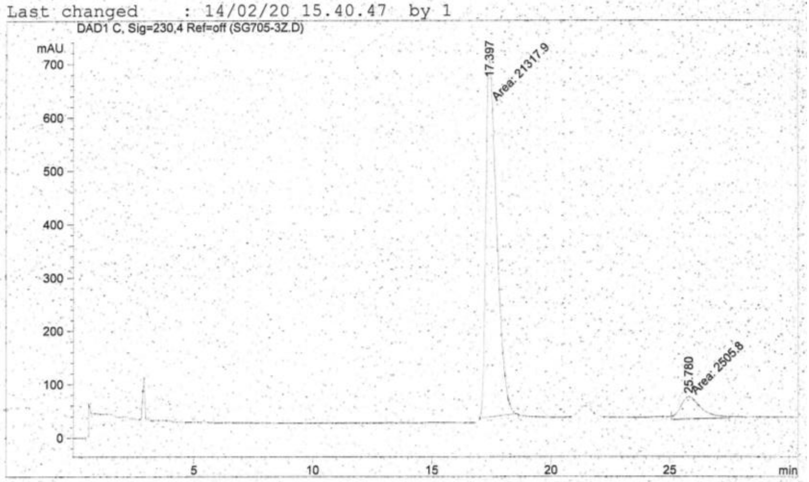


Area Percent Report

```
Sorted By        :     Signal
Multiplier       :     1.0000
Dilution         :     1.0000
```

Signal 1: DAD1 C, Sig=230,4 Ref=off

| Peak # | RetTime [min] | Type | Width [min] | Area [mAU*s] | Height [mAU] | Area % |
|---|---|---|---|---|---|---|
| 1 | 17.397 | MM | 0.5311 | 2.13179e4 | 669.02948 | 89.4819 |
| 2 | 25.780 | MM | 1.0136 | 2505.79980 | 41.20261 | 10.5181 |

Totals : 2.38237e4 710.23209

Results obtained with enhanced integrator!

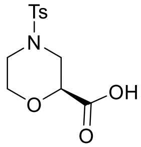


Injection Date : 30/04/21 12.45.08
Sample Name : SG199r_ADH_20et Vial : 1
Acq. Operator : sabrina
Acq. Method : C:\HPCHEM\1\METHODS\CECE.M
Last changed : 14/02/20 15.40.47 by 1
Analysis Method : C:\HPCHEM\1\METHODS\CECE.M
Last changed : 30/04/21 14.21.48 by sabrina
(modified after loading)

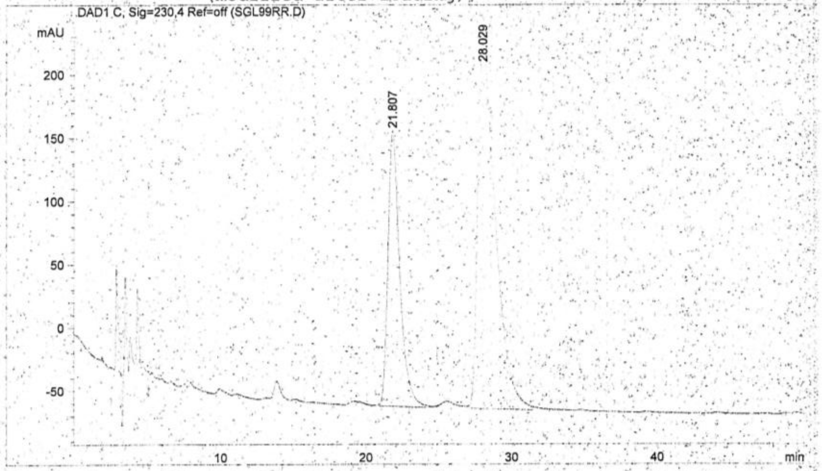


Area Percent Report

Sorted By : Signal
Multiplier : 1.0000
Dilution : 1.0000

Signal 1: DAD1 C, Sig=230,4 Ref=off

| Peak # | RetTime [min] | Type | Width [min] | Area [mAU*s] | Height [mAU] | Area % |
|---|---|---|---|---|---|---|
| 1 | 21.807 | VV | 0.7780 | 1.22592e4 | 217.35796 | 33.0990 |
| 2 | 28.029 | VV | 1.0347 | 2.47788e4 | 288.32028 | 66.9010 |

Totals : 3.70380e4 505.67824

Results obtained with enhanced integrator!

*** End of Report ***

Injection Date : 30/04/21 13.36.53
Sample Name : SG199e_ADH_20et Vial : 1
Acq. Operator : sabrina
Method : C:\HPCHEM\1\METHODS\CECE.M
Last changed : 14/02/20 15.40.47 by 1

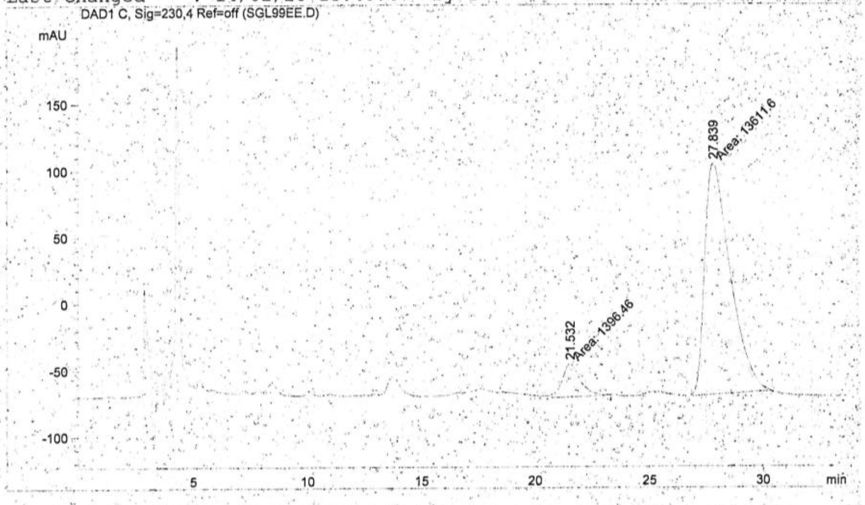


Area Percent Report

Sorted By : Signal
Multiplier : 1.0000
Dilution : 1.0000

Signal 1: DAD1 C, Sig=230,4 Ref=off

| Peak # | RetTime [min] | Type | Width [min] | Area [mAU*s] | Height [mAU] | Area % |
|---|---|---|---|---|---|---|
| 1 | 21.532 | MM | 0.9247 | 1396.45972 | 25.17068 | 9.3047 |
| 2 | 27.839 | MM | 1.3068 | 1.36116e4 | 173.60303 | 90.6953 |

Totals : 1.50081e4 198.77371

Results obtained with enhanced integrator!

*** End of Report ***

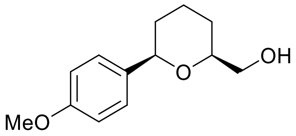


Injection Date : 30/06/21 16.53.31
Sample Name : SGL125r amy3 10i Vial : 1
Acq. Operator : sabrina
Method : C:\HPCHEM\1\METHODS\CECE.M
Last changed : 30/06/21 17.09.21 by sabrina
(modified after loading)

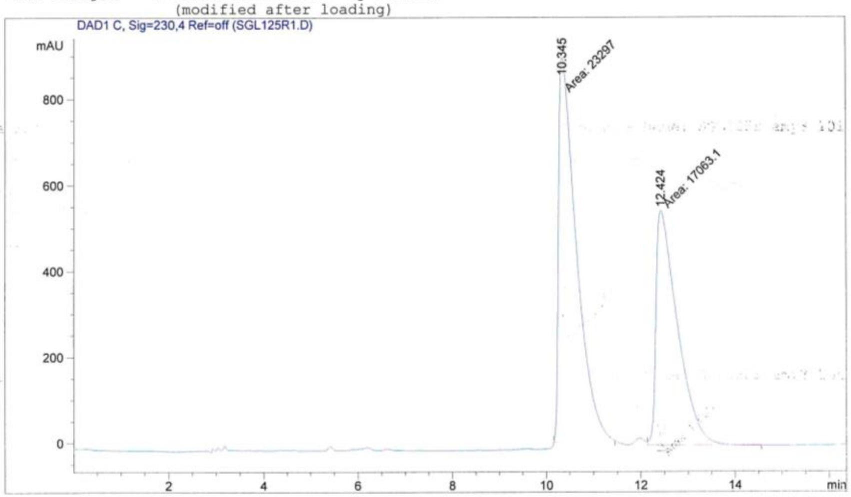


Area Percent Report

Sorted By : Signal
Multiplier : 1.0000
Dilution : 1.0000

Signal 1: DAD1 C, Sig=230,4 Ref=off

| Peak # | RetTime [min] | Type | Width [min] | Area [mAU*s] | Height [mAU] | Area % |
|---|---|---|---|---|---|---|
| 1 | 10.345 | MM | 0.4338 | 2.32970e4 | 895.15320 | 57.7228 |
| 2 | 12.424 | MM | 0.5207 | 1.70631e4 | 546.14844 | 42.2772 |

Totals : 4.03602e4 1441.30164

Results obtained with enhanced integrator!

*** End of Report ***

Injection Date : 09/07/21 15.16.33
Sample Name : SGL145 Vial : 1
Acq. Operator : Saria
Method : C:\HPCHEM\1\METHODS\CECE.M
Last changed : 05/07/21 17.13.36 by Lucia

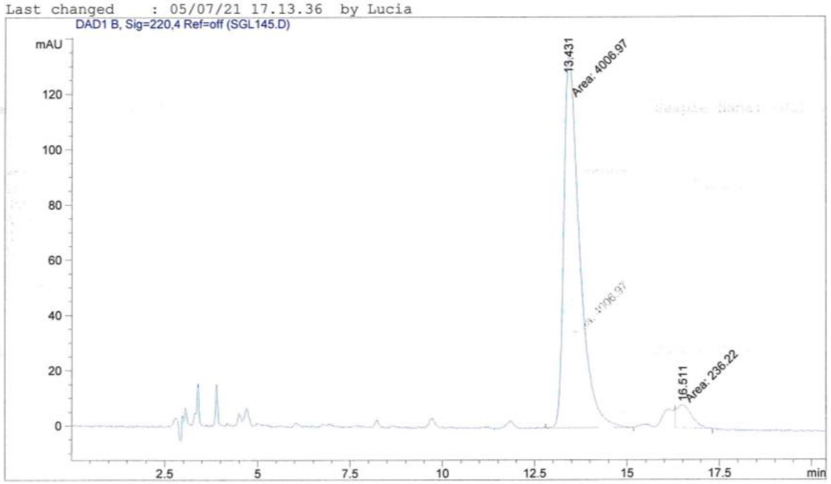


Area Percent Report

Sorted By : Signal
Multiplier : 1.0000
Dilution : 1.0000

Signal 1: DAD1 B, Sig=220,4 Ref=off

| Peak # | RetTime [min] | Type | Width [min] | Area [mAU*s] | Height [mAU] | Area % |
|---|---|---|---|---|---|---|
| 1 | 13.431 | MM | 0.4961 | 4006.96704 | 134.62198 | 94.4330 |
| 2 | 16.511 | MM | 0.4718 | 236.21999 | 8.34436 | 5.5670 |

Totals : 4243.18703 142.96634

Results obtained with enhanced integrator!

*** End of Report ***
